# Design, Synthesis and Fungicidal Activity of Ester Derivatives of 4-(3,4-Dichloroisothiazole) 7-Hydroxy Coumarin

**DOI:** 10.3390/molecules28135205

**Published:** 2023-07-04

**Authors:** Kun Li, Yue Zhang, Zeyu Hong, Zhenwu Yu, Xiaoyu Liu, Zhihong Duan, Wei Gao, Liangfu Tang, You Lv, Zhijin Fan

**Affiliations:** 1 State Key Laboratory of Elemento-Organic Chemistry, College of Chemistry, Nankai University, Tianjin 300071, China; 2 Frontiers Science Center for New Organic Matter, College of Chemistry, Nankai University, Tianjin 300071, China; 3 College of Agricultural and Biological Engineering, Heze University, Heze 274015, China

**Keywords:** natural product, carboxylic acid ester, fungicidal activity, density functional theory calculations, 3D-QSAR

## Abstract

The development of new fungicides is vital for safeguarding crops and ensuring sustainable agriculture. Building on our previous finding that 4-(3,4-dichloroisothiazole)-7-hydroxy coumarins can be used as fungicidal leads, 44 novel coumarin ester derivatives were designed and synthesized to evaluate whether esterification could enhance their fungicidal activity. In vitro fungicidal bioassays indicated that compound **2ai** displayed good activity against *Alternaria solani*, *Botrytis cinereal*, *Cercospora arachidicola*, *Physalospora piricola* and *Sclerotinia sclerotiorum*, with an EC_50_ value ranging from 2.90 to 5.56 μg/mL, comparable to the lead compound **1a**, with its EC_50_ value ranging from 1.92 to 9.37 μg/mL. In vivo bioassays demonstrated that compounds **1a**, **2ar** and **2bg** showed comparable, excellent efficacy against *Pseudoperonospora cubensis* at a dose of 25 µg/mL. Our research shows that the esterification of 4-(3,4-dichloroisothiazole) 7-hydroxycoumarins results in a fungicidal activity equivalent to that of its lead compounds. Furthermore, our density functional theory (DFT) calculations and 3D-QSAR modeling provide a rational explanation of the structure–activity relationship and offer valuable insights to guide further molecular design.

## 1. Introduction

Agrochemicals are important strategical materials for crop protection and food safety production [1,2]. However, the unreasonable and unscientific use of some pesticides has also resulted in a series of risks [3,4], including increasing pest resistance [5], toxicity against non-target organisms and potential environmental pollution [6]. Therefore, the development of green and high-activity agricultural chemicals with good environmental behavior is always crucial for plant protection.

**Figure 1:**
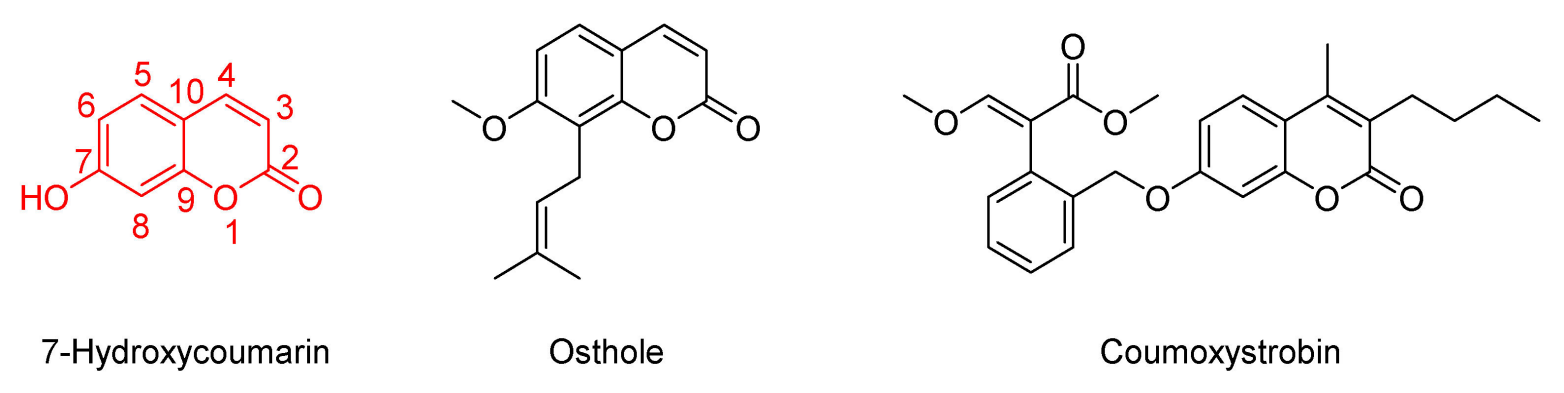
Representatives of coumarin-containing fungicides.

Natural products are usually metabolites or secondary metabolites of plants, microorganisms and animals, which have the advantages of structural diversity, biocompatibility, low resistance risk, a unique mode of action and a specific target [7,8,9,10,11]. They are widely used in the development of novel medicines and agrochemicals [12,13]. Coumarins are a highly significant class of natural products with bactericidal [14], anticoagulant [15], anti-inflammatory [16], antiviral [17], anti-HIV [18] and antitumor activity [19,20]; they are considered as phytoalexins for plant defense against pathogen attacks [1,21]. Several coumarin derivatives, such as osthole and coumoxystrobin, have been reported as fungicides [22,23] (Figure 1).

**Figure 2:**
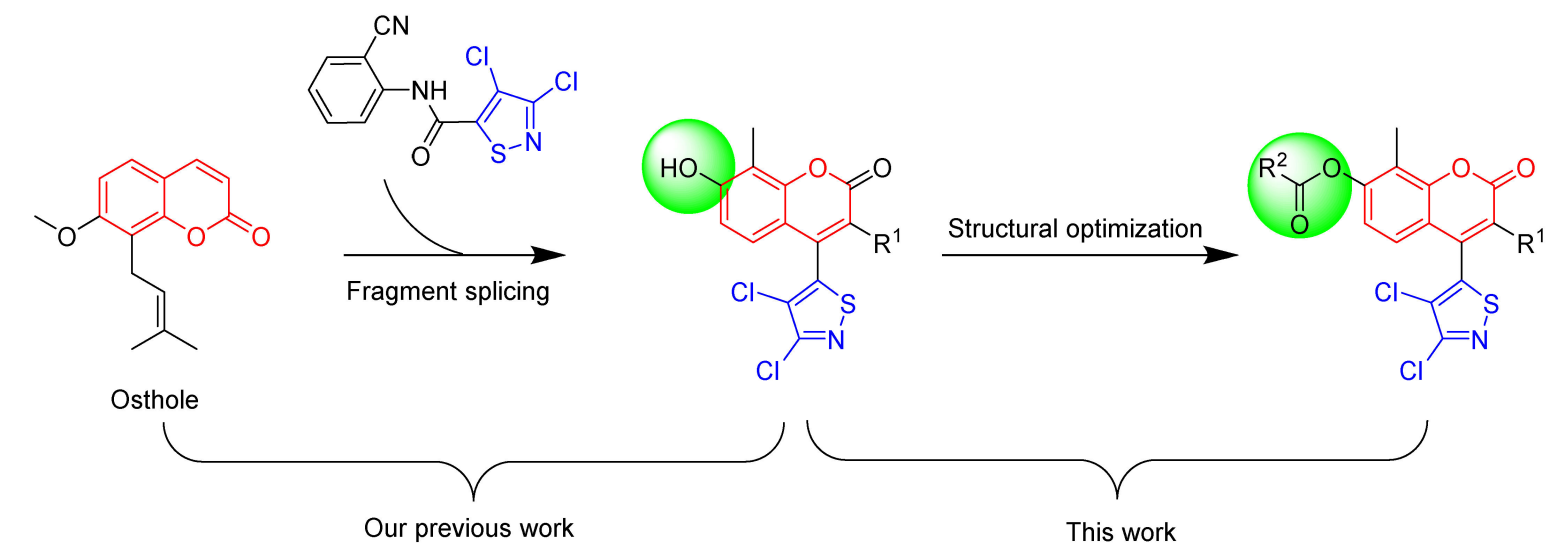
Molecular design of the target compounds.

Lead optimization is an effective approach for the development of novel pesticides [24,25]. Among the different methods for novel pesticide development, intermediate derivatization is effective and widely adopted [26,27]. Previously, our group discovered 4-(3,4-dichloroisothiazole) 7-hydroxy coumarins as fungicidal leads by combining the naturally occurring coumarin and a substructure (i.e., 3,4-dichloroisothiazole) of the compounds with systemic acquired resistance. Although compounds **1a** and **1b** displayed favorable in vitro activity, their in vivo performance was suboptimal [1]. Subsequently, a series of 3,4-dichloroisothiazolocoumarin-containing strobilurins were developed and found to have poor solubility [2]. To continue the exploration of fungicidal leads and improve the physicochemical properties of a weak acidic moiety of 7-hydroxy group on the coumarins, the esterification of 7-hydroxy coumarins was carried out (Figure 2), the in vitro and in vivo fungicidal activity of the target compounds were simultaneously evaluated, and notably, compounds **2ai** and **2ar** were found to show good activity.

## 2. Results and Discussion

### 2.1. Chemical Synthesis

**Scheme 1:**
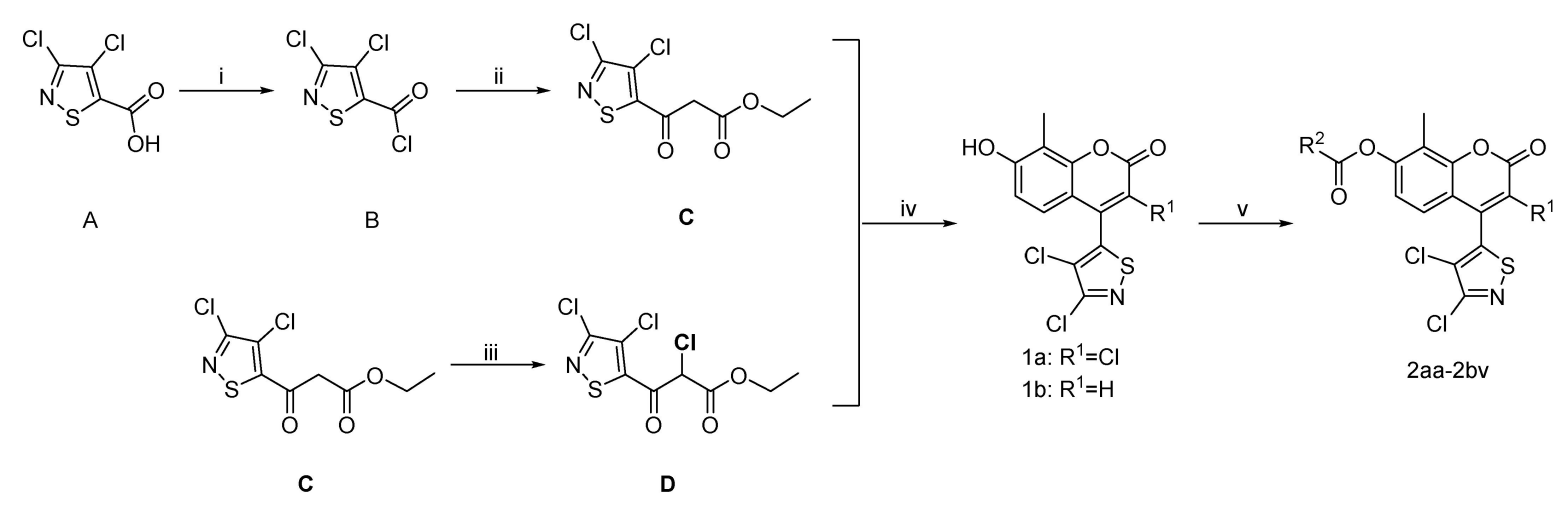
General synthesis procedure for compounds **2aa**–**2bv**. Reagents and conditions: (i) SOCl_2_, 80 °C, reflux, 4 h. (ii) Ethyl potassium malonate, MgCl_2_, TEA, THF, 0 °C to r.t., overnight; HCl, H_2_O, r.t., 2 h. (iii) SO_2_Cl_2_, CH_2_Cl_2_, 0 °C to r.t., overnight. (iv) 2-Methylresorcinol, CF_3_COOH, reflux, 4–8 h. (v) Acyl chloride,4-DMAP, Et_3_N, CH_2_Cl_2_, r.t., overnight.

The synthetic route for the target compounds **2aa**–**2bv** is outlined in Scheme 1. The target compounds **2aa**–**2bv** were prepared via esterification using compound **1** with the corresponding acyl chloride. The specific structures of the target compounds are shown in Table 1.

### 2.2. X-ray Diffraction of Compound ***2be***

**Figure 3:**
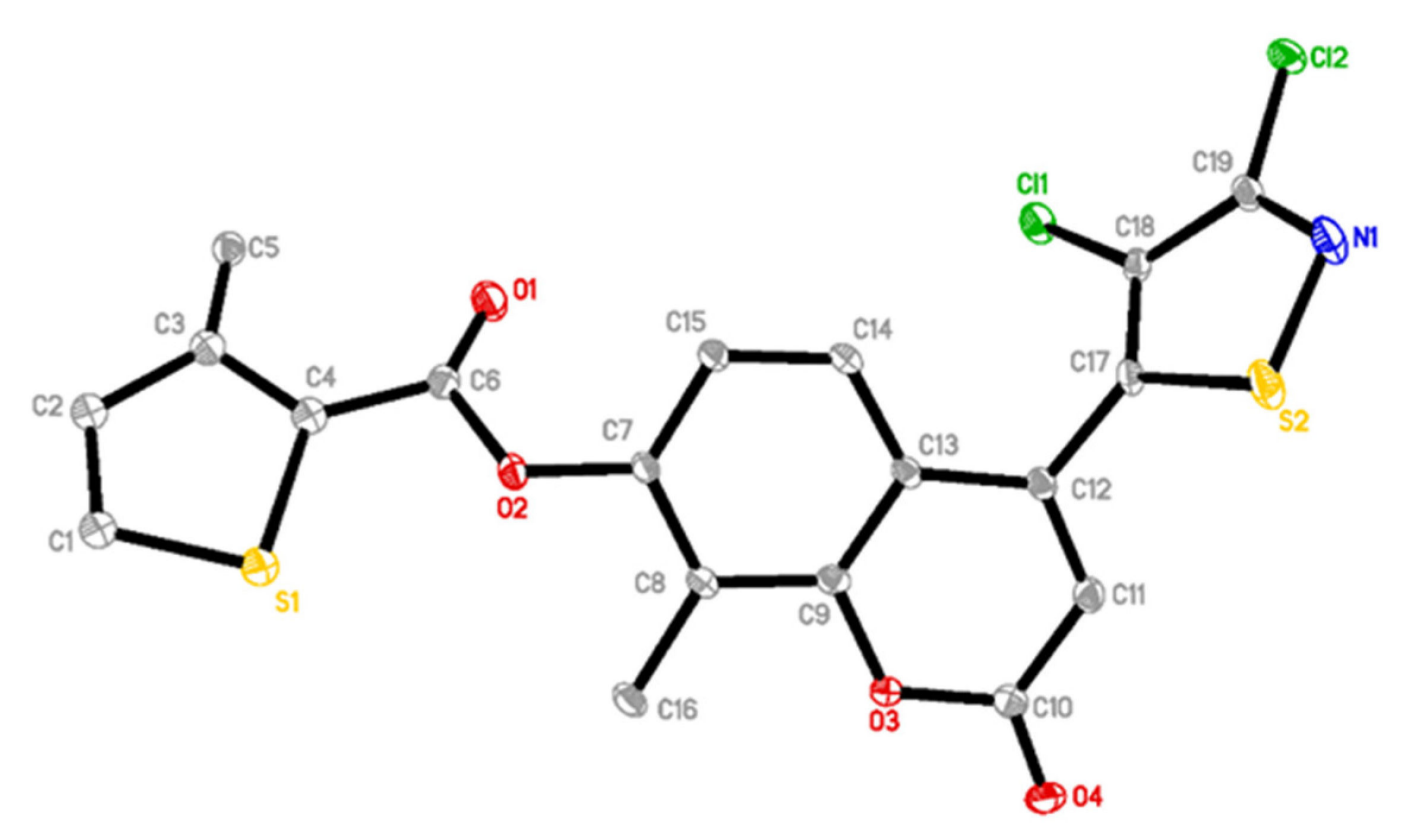
X-ray crystal structure of **2be** (CCDC: 2256560).

The crystal structure cell stacking diagram of **2be**, shown in Figure 3, was identified via X-ray diffraction analysis for structure validation. The crystal belongs to the monoclinic system with the space group P2_1/c_, and each unit cell contains four molecules. The unit cell parameters are a = 16.9633(6) Å, b = 7.2717(3) Å, c = 14.8314(6) Å; α = 90, β = 90.164(4), γ = 90; and Z = 4, V = 1829.48(12) Å^3^. In addition, the torsional angles (all near 180° or 0°) and the measured dihedral angles between the benzene and pyranone rings indicate that the entire coumarin ring was coplanar. Each molecule has a coumarin ring and an isothiazole moiety with a measured dihedral angle of 55.1° between these two moieties. There are no hydrogen bonds to be found in the crystal structure. The additional data for **2be** were alternatively obtained from the Cambridge Crystallographic Data Center (www.ccdc.cam.ac.uk, accessed on 1 June 2023), denoted as CCDC 2256560.

### 2.3. In Vitro Fungicidal Activity

The in vitro fungicidal activity of target compounds **2aa–2bv** against seven representative plant pathogens at 50 μg/mL are shown in Table 2. The osthole, isotianil and compounds **1a** and **1b** were used as positive controls. Compound **2ai** showed more than 75% inhibition against *Alternaria solani*, *Botrytis cinerea*, *Cercospora arachidicola* and *Sclerotinia sclerotiorum*, being similar to the positive control compound **1a**. Meanwhile, compound **2aj** showed more than 75% inhibition against *C. arachidicola* (76%) and *Physalospora piricola* (78%), and compound **2ar** showed more than 75% inhibition against *C. arachidicola* (77%), *P. piricola* (77%) and *Rhizoctonia solani* (76%).

In order to further explore the fungicidal activity of the compounds, their median effective concentration (EC_50_) values were examined. As shown in Table 3, compound **2ai** displayed higher fungicidal activity against *A. solani*, with an EC_50_ value of 3.20 μg/mL, being more potent than compound **1a** (EC_50_ = 6.24 μg/mL). For *B. cinerea*, compound **2ai** showed slightly weaker activity than compound **1a**. Compound **2ai** was more potent against *C. arachidicola* (EC_50_ = 3.82 μg/mL) than compound **2aj** (EC_50_ = 5.08 μg/mL) and the positive control compound **1a** (EC_50_ = 5.18 μg/mL). For *P. piricola*, compound **2ai** (with an EC_50_ value of 3.65 μg/mL) showed 2.5 times more potency than compound **1a** (with an EC_50_ value of 9.37 μg/mL). For *S. sclerotiorum*, compound **2bg** (EC_50_ = 5.71 μg/mL) displayed higher fungicidal activity than compound **1b** (EC_50_ = 9.78 μg/mL); therefore, some of the esters could exhibit better fungicidal activity towards certain pathogens than the original hydroxyl derivatives.

### 2.4. In Vivo Fungicidal Activity

The in vivo fungicidal activity of the target compounds was tested against *Pseudoperonospora cubensis*. The results are shown in Table 4. Compounds **2ai**, **2aj**, **2ar** and **2bg** showed excellent activity against *P. cubensis* at 200 and 100 μg/mL, results which were consistent with compounds **1a** and **1b**. When the concentration was reduced to 50 μg/mL and 25 μg/mL, the compounds **2ar** maintained 80% and 75% inhibition rates, and compound **2bg** maintained 88% and 65% inhibition rates, being equivalent to the positive control compound **1a**. In short, **2ar** and **2bg** showed excellent in vivo activity against *P. cubensis,* and they could be potential candidates for further research.

### 2.5. 3D-QSAR Analysis

**Figure 4:**
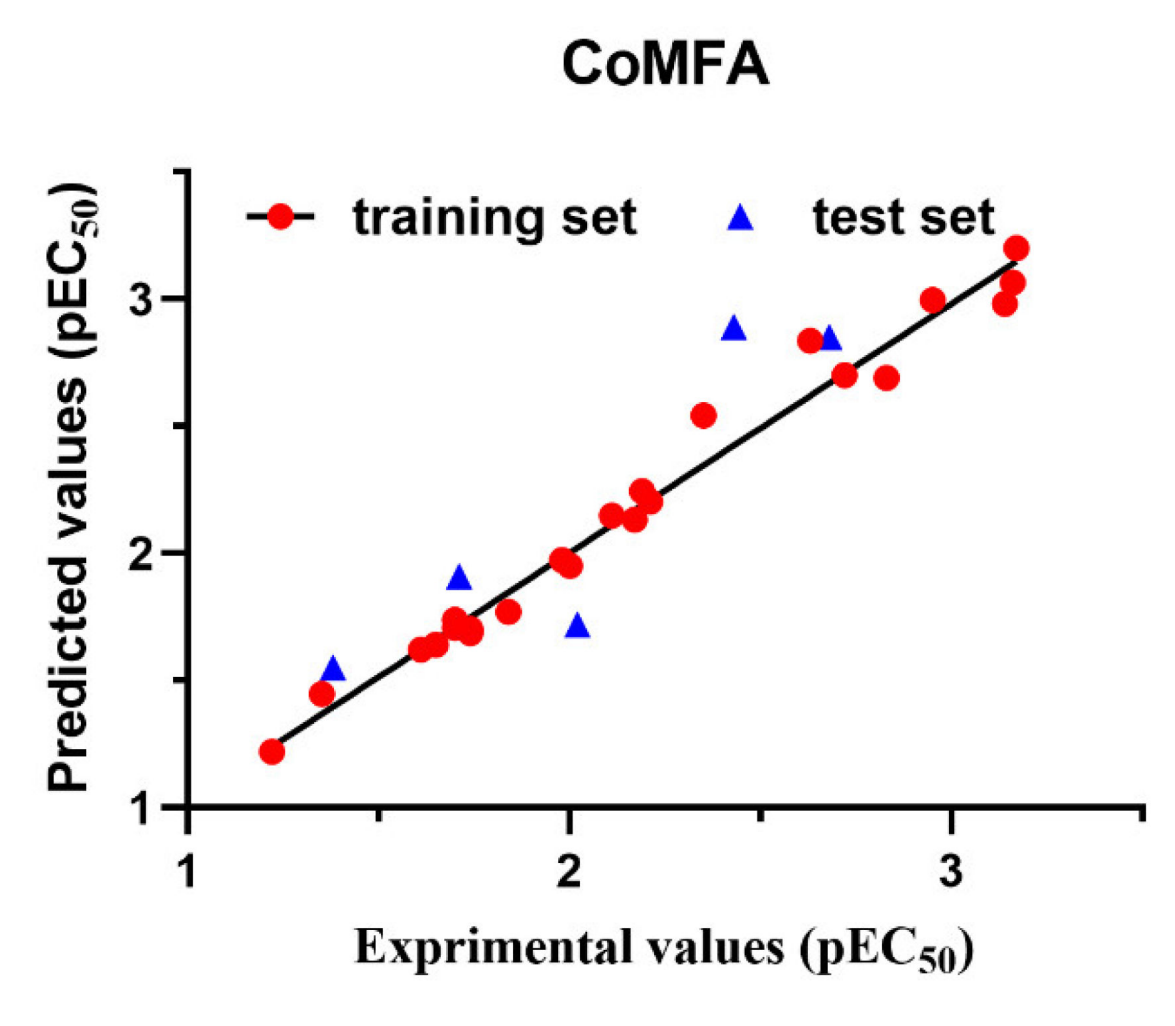
Linear relationship diagram of CoMFA.

The analysis of the statistical parameters of the established 3D-QSAR model was carried out on the basis of the optimal molecular conformation of fungicidal activity, and the statistical results are shown in Table 5. The CoMFA model includes the effects of the steric field and electrostatic field. When the optimum component was five, the parameters (Q^2^ = 0.797, R^2^ = 0.979, SEE = 0.096, F = 166.514) were obtained. For CoMFA models, when Q^2^ is greater than 0.5 and R^2^ is greater than 0.8, this suggests that the models have high reliability and a good prediction ability. The predicted versus experimental values (Figure 4) obtained using this 3D-QSAR model indicate that the predictive capability of this model is robust.

**Figure 5:**
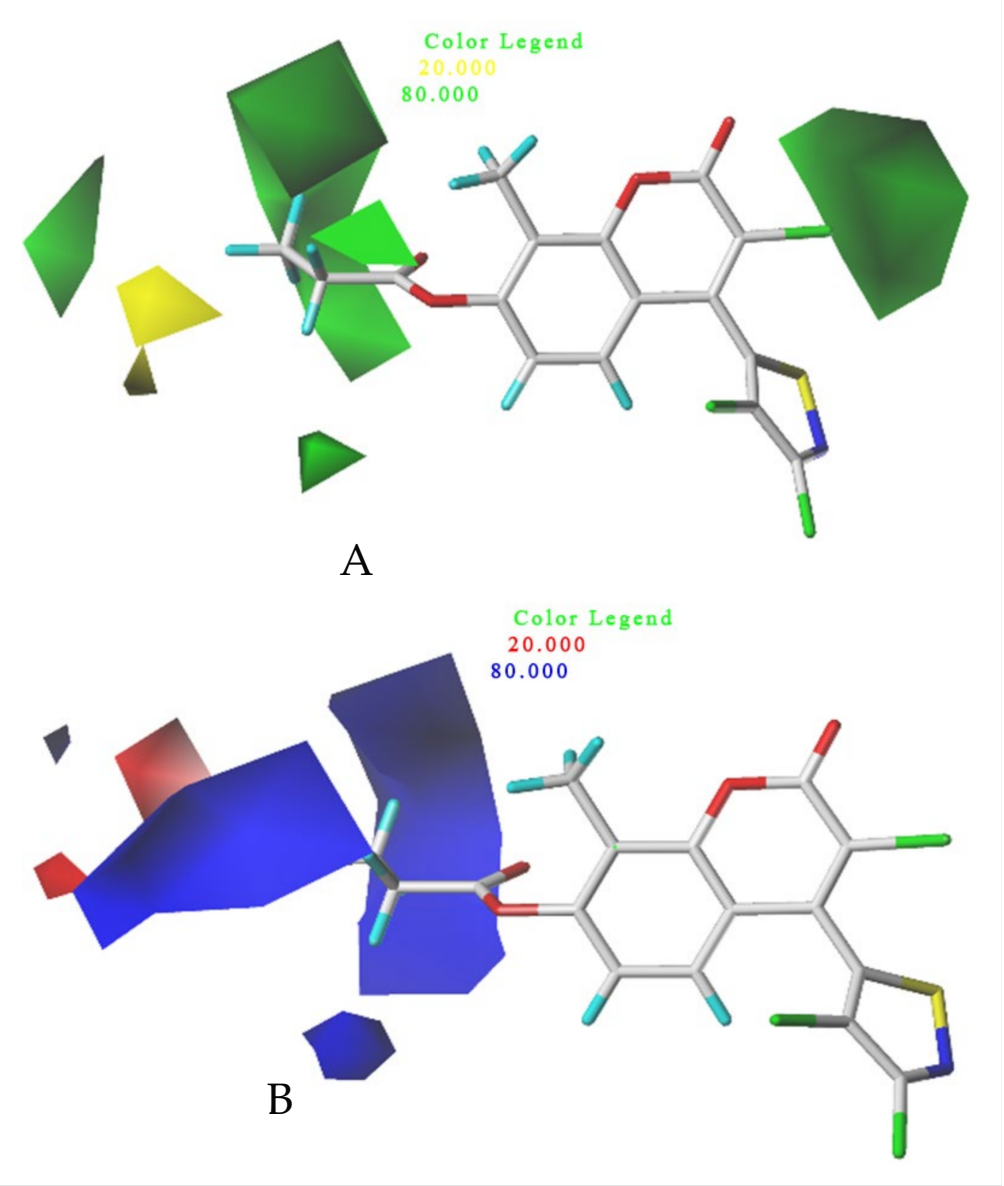
Contour maps of the steric field (**A**) and electrostatic field (**B**) of the CoMFA model.

In the steric field equipotential diagram of the CoMFA model, the green area indicates that the large-volume group is beneficial for improving the molecular activity, and the yellow area indicates that the large-volume group is not beneficial for improving the molecular activity. Figure 5A indicates that increasing the group volume of the 3-position and 7-position on the coumarin ring is conducive to improving the inhibitory activity of the target compounds against *P. piricola*. In the electrostatic field equipotential diagram of the CoMFA model, the blue area indicates that the positively charged group is beneficial for improving the molecular activity, and the red area indicates that the negatively charged group is beneficial for improving the molecular activity. Figure 5B indicates that increasing the positively charged group in the 7-position on the coumarin ring is conducive to improving the inhibitory activity of the target compounds against *P. piricola*.

As mentioned above, a larger group and positively charged group at the 7-position of the coumarin ring and a larger group at the 3-position of the coumarin ring are beneficial for improving fungicidal activity against *P. piricola*.

### 2.6. DFT Calculations of ***2ai***, ***2aq*** and ***1a***

The molecular total energy (MTE), frontier orbital energy (FMO), energy gaps between the HOMO and LUMO of compounds with high (**2ai**) or low (**2aq**) fungicidal potency and the positive control **1a** are listed in Table 6. The energy gaps between the HOMO and LUMO were 4.17, 3.83 and 4.00 eV, respectively (Table 6).

**Figure 6:**
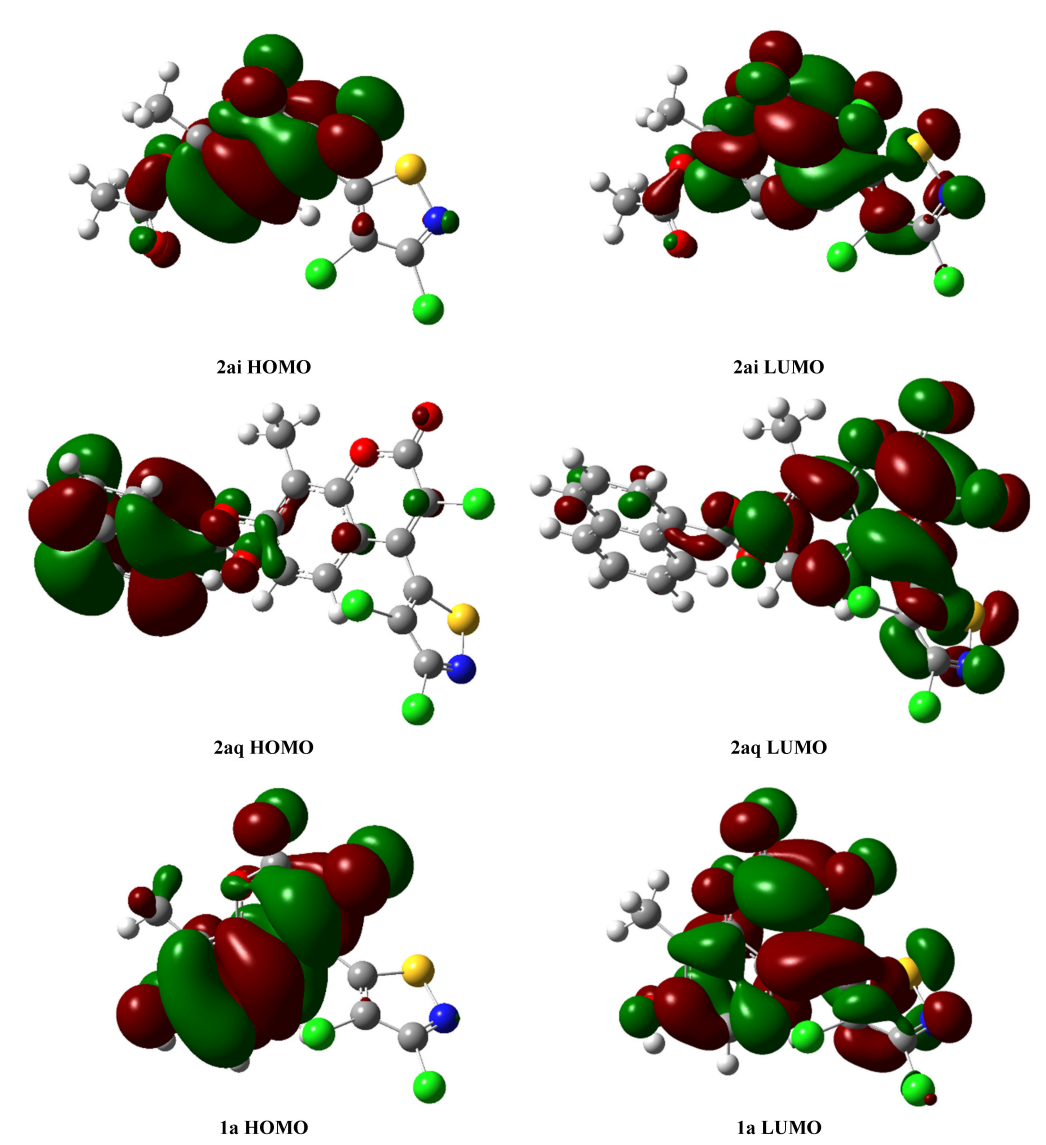
HOMO and LUMO of **2ai**, **2aq** and **1a**.

LUMO and HOMO affect electron transition by accepting and providing electrons, respectively, according to the FMO theory. In Figure 6, the HOMO of **2ai** and **1a** is mainly located on the benzopyranone ring, while the LUMO is mainly located on the benzopyranone ring and the isothiazole ring, suggesting that the electrons of both compounds were transferred from the benzopyranone ring to the isothiazole ring. However, the HOMO of **2aq** with low fungicidal potency is primarily distributed in the naphthalene ring, and the LUMO is distributed in the ester bond and benzopyranone and isothiazole rings, indicating that the direction of electron transition is different from that of **2ai** with high fungicidal potency and the positive control **1a**.

The aforementioned results regarding the energy and electron transition of **2ai** are in approximate agreement with those of **1a**, whereas that for **2aq** was different, explaining their difference in fungicidal potency on the basis of their chemical structures.

## 3. Materials and Methods

### 3.1. Instruments and Materials

All solvents and reagents used were obtained from commercial sources and were not further purified. The melting points were measured using an X-4 digital display micro-melting point apparatus (Gongyi, China) and were not corrected. ^1^H NMR (400 MHz), ^13^C NMR (101 MHz) or ^19^F NMR (376 MHz) spectra were measured using TMS as the internal standard with a Bruker AV 400 spectrometer in CDCl_3_. High-resolution mass spectrometry (HRMS) data were measured using a Varian QFT-ESI instrument. Crystal structures were determined using a Rigaku 007 Saturn 70 diffractometer.

### 3.2. General Procedures for Preparation of the Target Compounds ***2aa***–***2bv***

According to the reported method, intermediates **1a** and **1b** were synthesized [1]. The compounds **2aa**–**2bv** were synthesized according to the reported method with minor modifications [28].

To a mixture of compound **1** (0.25 mmol), 4-DMAP (0.50 mmol) in dichloromethane (15 mL) and triethylamine (0.50 mmol), acyl chloride (0.75 mmol) was slowly added under stirring, and the reaction mixture was then stirred overnight at room temperature. After the reaction was complete, the mixture was extracted with dichloromethane (3 × 15 mL). The organic phase was sequentially washed with saturated NaHCO_3_ (20 mL) and brine (20 mL) and dried over anhydrous Na_2_SO_4_. The solution was then filtered and concentrated under reduced pressure. The crude product was purified using column chromatography with a mobile phase of dichloromethane/methanol (500:1–200:1, *v*/*v*) to yield compounds **2aa**–**2bv**.

**2aa**. 3-Chloro-4-(3,4-dichloroisothiazol-5-yl)-8-methyl-2-oxo-2*H*-chromen-7-yl benzoate, white solid; yield: 95%; m.p.: 186–188 °C; ^1^H NMR (400 MHz, Chloroform-*d*) δ 8.21 (d, *J* = 7.2 Hz, 2H), 7.68 (t, *J* = 7.5 Hz, 1H), 7.54 (t, *J* = 7.8 Hz, 2H), 7.18 (d, *J* = 8.7 Hz, 1H), 7.03 (d, *J* = 8.7 Hz, 1H), 2.39 (s, 3H). ^13^C NMR (101 MHz, Chloroform-*d*) δ 164.23, 155.84, 152.77, 151.95, 150.91, 149.55, 139.00, 134.32, 130.34, 128.87, 128.35, 123.79, 123.27, 123.07, 120.53, 119.89, 115.74, 9.44. HRMS (ESI) *m*/*z* calcd for C_20_H_11_Cl_3_NO_4_S^+^ (M+H)^+^: 465.9474, found: 465.9467.

**2ab**. 3-Chloro-4-(3,4-dichloroisothiazol-5-yl)-8-methyl-2-oxo-2*H*-chromen-7-yl 2-phenylacetate, pale yellow solid; yield: 90%; m.p.: 55–56 °C; ^1^H NMR (400 MHz, Chloroform-*d*) δ 7.42–7.31 (m, 5H), 7.01 (d, *J* = 8.7 Hz, 1H), 6.93 (d, *J* = 8.7 Hz, 1H), 3.92 (s, 2H), 2.18 (s, 3H). ^13^C NMR (101 MHz, Chloroform-*d*) δ 169.06, 155.78, 152.41, 151.87, 150.81, 149.59, 138.89, 132.71, 129.31, 128.92, 127.75, 123.62, 123.26, 123.12, 120.33, 119.58, 115.68, 41.30, 9.04. HRMS (ESI) *m*/*z* calcd for C_21_H_13_Cl_3_NO_4_S^+^ (M+H)^+^: 479.9631, found: 479.9623. 

**2ac**. 3-Chloro-4-(3,4-dichloroisothiazol-5-yl)-8-methyl-2-oxo-2*H*-chromen-7-yl 4-methoxybenzoate, white solid; yield: 54%; m.p.: 196–198 °C; ^1^H NMR (400 MHz, Chloroform-*d*) δ 8.16 (d, *J* = 8.8 Hz, 2H), 7.16 (d, *J* = 8.7 Hz, 1H), 7.01 (d, *J* = 8.7 Hz, 3H), 3.91 (s, 3H), 2.39 (s, 3H). ^13^C NMR (101 MHz, Chloroform-*d*) δ 164.45, 163.97, 155.97, 152.97, 151.96, 150.91, 149.60, 139.05, 132.61, 123.70, 123.29, 122.96, 120.61, 120.50, 120.03, 115.61, 114.14, 55.65, 9.44. HRMS (ESI) *m*/*z* calcd for C_21_H_13_Cl_3_NO_5_S^+^ (M+H)^+^: 495.9580, found: 495.9571. 

**2ad**. 3-Chloro-4-(3,4-dichloroisothiazol-5-yl)-8-methyl-2-oxo-2*H*-chromen-7-yl 2,4-difluorobenzoate, pale yellow solid; yield: 92%; m.p.: 192–194 °C; ^1^H NMR (400 MHz, Chloroform-*d*) δ 8.19 (q, *J* = 7.9 Hz, 1H), 7.21 (d, *J* = 8.7 Hz, 1H), 7.11–6.97 (m, 3H), 2.43 (s, 3H). ^13^C NMR (101 MHz, Chloroform-*d*) δ 166.68 (dd, *J* = 259.1, 12.2 Hz), 163.44 (dd, *J* = 265.3, 12.8 Hz), 161.08 (d, *J* = 4.6 Hz), 155.85, 152.21, 151.86, 150.90, 149.61, 138.96, 134.66 (t, *J* = 8.0 Hz), 123.81, 123.30, 123.28, 120.45, 119.73, 115.89, 113.43 (dd, *J* = 9.6, 3.6 Hz), 112.31 (dd, *J* = 21.6, 3.7 Hz), 105.82 (t, *J* = 25.3 Hz), 9.45. ^19^F NMR (376 MHz, Chloroform-*d*) δ −98.74 (d, *J* = 13.3 Hz), −101.46 (d, *J* = 14.3 Hz). HRMS (ESI) *m*/*z* calcd for C_20_H_9_Cl_3_F_2_NO_4_S^+^ (M+H)^+^: 501.9286, found: 501.9277. 

**2ae**. 3-Chloro-4-(3,4-dichloroisothiazol-5-yl)-8-methyl-2-oxo-2*H*-chromen-7-yl 3-bromobenzoate, white solid; yield: 86%; m.p.: 191–193 °C; ^1^H NMR (400 MHz, Chloroform-*d*) δ 8.35 (s, 1H), 8.15 (d, *J* = 7.8 Hz, 1H), 7.82 (d, *J* = 8.0 Hz, 1H), 7.44 (t, *J* = 7.9 Hz, 1H), 7.16 (d, *J* = 8.7 Hz, 1H), 7.03 (d, *J* = 8.7 Hz, 1H), 2.39 (s, 3H). ^13^C NMR (101 MHz, Chloroform-*d*) δ 163.00, 155.80, 152.42, 151.85, 150.91, 149.65, 138.94, 137.27, 133.24, 130.43, 130.26, 128.90, 123.86, 123.32, 122.93, 120.52, 119.69, 115.93, 9.48. HRMS (ESI) *m*/*z* calcd for C_20_H_10_BrCl_3_NO_4_S^+^ (M+H)^+^: 543.8579, found: 543.8569. 

**2af**. 3-Chloro-4-(3,4-dichloroisothiazol-5-yl)-8-methyl-2-oxo-2*H*-chromen-7-yl 4-chlorobenzoate, white solid; yield: 98%; m.p.: 184–186 °C; ^1^H NMR (400 MHz, Chloroform-*d*) δ 8.17 (d, *J* = 8.6 Hz, 2H), 7.55 (d, *J* = 8.6 Hz, 2H), 7.19 (d, *J* = 8.7 Hz, 1H), 7.05 (d, *J* = 8.7 Hz, 1H), 2.41 (s, 3H). ^13^C NMR (101 MHz, Chloroform-*d*) δ 163.42, 155.79, 152.50, 151.87, 150.91, 149.61, 140.99, 138.94, 131.69, 129.27, 126.78, 123.82, 123.30, 123.24, 120.48, 119.74, 115.85, 9.44. HRMS (ESI) *m*/*z* calcd for C_20_H_10_Cl_4_NO_4_S^+^ (M+H)^+^: 499.9084, found: 499.9076.

**2ag**. 3-Chloro-4-(3,4-dichloroisothiazol-5-yl)-8-methyl-2-oxo-2*H*-chromen-7-yl 4-fluorobenzoate, white solid; yield: 96%; m.p.: 168–169 °C; ^1^H NMR (400 MHz, Chloroform-*d*) δ 8.31–8.23 (m, 2H), 7.27–7.17 (m, 3H), 7.05 (d, *J* = 8.7 Hz, 1H), 2.41 (s, 3H). ^13^C NMR (101 MHz, Chloroform-*d*) δ 166.56 (d, *J* = 256.6 Hz), 163.31, 155.88, 152.57, 151.87, 150.91, 149.64, 138.98, 133.07 (d, *J* = 9.7 Hz), 124.57 (d, *J* = 3.0 Hz), 123.82, 123.26 (d, *J* = 10.5 Hz), 120.55, 119.82, 116.30, 116.08, 115.83, 9.45. ^19^F NMR (376 MHz, Chloroform-*d*) δ −102.78. HRMS (ESI) *m*/*z* calcd for C_20_H_10_Cl_3_FNO_4_S^+^ (M+H)^+^: 483.9380, found: 483.9377. 

**2ah**. 3-Chloro-4-(3,4-dichloroisothiazol-5-yl)-8-methyl-2-oxo-2*H*-chromen-7-yl 4-(trifluoromethyl)benzoate, white solid; yield: 60%; m.p.: 179–180 °C; ^1^H NMR (400 MHz, Chloroform-*d*) δ 8.34 (d, *J* = 8.1 Hz, 2H), 7.82 (d, *J* = 8.3 Hz, 2H), 7.19 (d, *J* = 8.7 Hz, 1H), 7.05 (d, *J* = 8.7 Hz, 1H), 2.39 (s, 3H). ^13^C NMR (101 MHz, Chloroform-*d*) δ 163.08, 155.72, 152.31, 151.83, 150.92, 149.61, 138.90, 135.68 (d, *J* = 32.8 Hz), 131.62, 130.76, 125.91 (q, *J* = 3.3 Hz), 123.91, 123.41 (q, *J* = 273.1 Hz), 123.39, 123.31, 120.43, 119.62, 116.00, 9.43. ^19^F NMR (376 MHz, Chloroform-*d*) δ −63.24. HRMS (ESI) *m*/*z* calcd for C_21_H_10_Cl_3_F_3_NO_4_S^+^ (M+H)^+^: 533.9348, found: 533.9346.

**2ai**. 3-Chloro-4-(3,4-dichloroisothiazol-5-yl)-8-methyl-2-oxo-2*H*-chromen-7-yl acetate, white solid; yield: 97%; m.p.: 140–142 °C; ^1^H NMR (400 MHz, Chloroform-*d*) δ 7.05 (d, *J* = 8.7 Hz, 1H), 6.98 (d, *J* = 8.7 Hz, 1H), 2.39 (s, 3H), 2.33 (s, 3H). ^13^C NMR (101 MHz, Chloroform-*d*) δ 168.45, 155.80, 152.44, 151.90, 150.82, 149.49, 138.95, 123.73, 123.22, 123.02, 120.23, 119.71, 115.68, 20.76, 9.30. HRMS (ESI) *m*/*z* calcd for C_15_H_9_Cl_3_NO_4_S^+^ (M+H)^+^: 403.9318, found: 403.9308. 

**2aj**. 3-Chloro-4-(3,4-dichloroisothiazol-5-yl)-8-methyl-2-oxo-2*H*-chromen-7-yl propionate, white solid; yield: 80%; m.p.: 125–127 °C; ^1^H NMR (400 MHz, Chloroform-*d*) δ 7.05 (d, *J* = 8.7 Hz, 1H), 6.98 (d, *J* = 8.7 Hz, 1H), 2.69 (q, *J* = 7.5 Hz, 2H), 2.34 (s, 3H), 1.32 (t, *J* = 7.5 Hz, 3H). ^13^C NMR (101 MHz, Chloroform-*d*) δ 171.98, 155.83, 152.56, 151.93, 150.85, 149.52, 138.96, 123.68, 123.23, 122.97, 120.23, 119.72, 115.60, 27.55, 9.26, 9.08. HRMS (ESI) *m*/*z* calcd for C_16_H_11_Cl_3_NO_4_S^+^ (M+H)^+^: 417.9474, found: 417.9464.

**2ak**. 3-Chloro-4-(3,4-dichloroisothiazol-5-yl)-8-methyl-2-oxo-2*H*-chromen-7-yl cyclopropanecarboxylate, white solid; yield: 94%; m.p.: 138–139 °C; ^1^H NMR (400 MHz, Chloroform-*d*) δ 7.05 (d, *J* = 8.7 Hz, 1H), 6.97 (d, *J* = 8.7 Hz, 1H), 2.34 (s, 3H), 1.98–1.85 (m, 1H), 1.24–1.19 (m, 2H), 1.14–1.08 (m, 2H). ^13^C NMR (101 MHz, Chloroform-*d*) δ 172.53, 155.85, 152.58, 151.95, 150.81, 149.48, 138.97, 123.65, 123.20, 122.90, 120.32, 119.75, 115.57, 12.80, 9.70, 9.24. HRMS (ESI) *m*/*z* calcd for C_17_H_11_Cl_3_NO_4_S^+^ (M+H)^+^: 429.9474, found: 429.9465. 

**2al**. 3-Chloro-4-(3,4-dichloroisothiazol-5-yl)-8-methyl-2-oxo-2*H*-chromen-7-yl isobutyrate, white solid; yield: 91%; m.p.: 155–157 °C; ^1^H NMR (400 MHz, Chloroform-*d*) δ 7.00 (d, *J* = 8.7 Hz, 1H), 6.95 (d, *J* = 8.7 Hz, 1H), 2.88 (hept, *J* = 7.0 Hz, 1H), 2.31 (s, 3H), 1.35 (d, *J* = 7.0 Hz, 6H). ^13^C NMR (101 MHz, Chloroform-*d*) δ 174.59, 155.84, 152.65, 151.94, 150.83, 149.50, 138.97, 123.67, 123.22, 122.93, 120.23, 119.68, 115.57, 34.20, 18.95, 9.18. HRMS (ESI) *m*/*z* calcd for C_17_H_13_Cl_3_NO_4_S^+^ (M+H)^+^: 431.9631, found: 431.9624.

**2am**. 3-Chloro-4-(3,4-dichloroisothiazol-5-yl)-8-methyl-2-oxo-2*H*-chromen-7-yl pivalate, white solid; yield: 88%; m.p.:166–168 °C; ^1^H NMR (400 MHz, Chloroform-*d*) δ 6.98 (d, *J* = 8.8 Hz, 1H), 6.94 (d, *J* = 8.8 Hz, 1H), 2.30 (s, 3H), 1.39 (s, 9H). ^13^C NMR (101 MHz, Chloroform-*d*) δ 176.10, 155.86, 152.89, 151.95, 150.82, 149.49, 139.00, 123.66, 123.21, 122.88, 120.25, 119.65, 115.51, 39.41, 27.14, 9.13. HRMS (ESI) *m*/*z* calcd for C_18_H_15_Cl_3_NO_4_S^+^ (M+H)^+^: 445.9787, found: 445.9778.

**2an**. 3-Chloro-4-(3,4-dichloroisothiazol-5-yl)-8-methyl-2-oxo-2*H*-chromen-7-yl thiophene-2-carboxylate, white solid; yield: 77%; m.p.: 210–211 °C; ^1^H NMR (400 MHz, Chloroform-*d*) δ 8.03 (d, *J* = 3.8 Hz, 1H), 7.74 (d, *J* = 5.0 Hz, 1H), 7.25–7.16 (m, 2H), 7.01 (d, *J* = 8.7 Hz, 1H), 2.41 (s, 3H). ^13^C NMR (101 MHz, Chloroform-*d*) δ 159.59, 155.90, 152.25, 151.88, 150.88, 149.63, 138.99, 135.64, 134.64, 131.40, 128.44, 123.75, 123.31, 123.18, 120.61, 119.84, 115.80, 9.45. HRMS (ESI) *m*/*z* calcd for C_18_H_9_Cl_3_NO_4_S_2_^+^ (M+H)^+^: 471.9038, found: 471.9032.

**2ao**. 3-Chloro-4-(3,4-dichloroisothiazol-5-yl)-8-methyl-2-oxo-2*H*-chromen-7-yl furan-2-carboxylate, white solid; yield: 71%; m.p.: 203–204 °C; ^1^H NMR (400 MHz, Chloroform-*d*) δ 7.72 (s, 1H), 7.46 (d, *J* = 3.5 Hz, 1H), 7.17 (d, *J* = 8.7 Hz, 1H), 7.01 (d, *J* = 8.7 Hz, 1H), 6.64 (dd, *J* = 3.5, 1.7 Hz, 1H), 2.39 (s, 3H). ^13^C NMR (101 MHz, Chloroform-*d*) δ 155.81, 151.85, 150.88, 149.59, 147.95, 142.93, 138.94, 123.78, 123.29, 123.23, 120.59, 119.73, 115.87, 112.54, 9.40. HRMS (ESI) *m*/*z* calcd for C_18_H_9_Cl_3_NO_5_S^+^ (M+H)^+^: 455.9267, found: 455.9258.

**2ap**. 3-Chloro-4-(3,4-dichloroisothiazol-5-yl)-8-methyl-2-oxo-2*H*-chromen-7-yl cinnamate, white solid; yield: 70%; m.p.: 168–169 °C; ^1^H NMR (400 MHz, Chloroform-*d*) δ 7.72 (d, *J* = 16.0 Hz, 1H), 7.43–7.36 (m, 2H), 7.26–7.19 (m, 3H), 6.92 (d, *J* = 8.7 Hz, 1H), 6.79 (d, *J* = 8.7 Hz, 1H), 6.46 (d, *J* = 16.0 Hz, 1H), 2.17 (s, 3H). ^13^C NMR (101 MHz, Chloroform-*d*) δ 164.43, 155.87, 152.66, 151.95, 150.90, 149.57, 148.16, 138.98, 133.74, 131.24, 129.12, 128.50, 123.71, 123.27, 122.99, 120.47, 119.82, 115.84, 115.65, 29.71, 9.43. HRMS (ESI) *m*/*z* calcd for C_22_H_13_Cl_3_NO_4_S^+^ (M+H)^+^: 491.9631, found: 491.9622.

**2aq**. 3-Chloro-4-(3,4-dichloroisothiazol-5-yl)-8-methyl-2-oxo-2*H*-chromen-7-yl 1-naphthoate, pale yellow solid; yield: 78%; m.p.: 213–215 °C; ^1^H NMR (400 MHz, Chloroform-*d*) δ 9.02 (d, *J* = 8.6 Hz, 1H), 8.56 (d, *J* = 7.3 Hz, 1H), 8.17 (d, *J* = 8.0 Hz, 1H), 7.96 (d, *J* = 8.1 Hz, 1H), 7.72–7.65 (m, 1H), 7.65–7.56 (m, 2H), 7.24 (d, *J* = 8.7 Hz, 1H), 7.05 (d, *J* = 8.7 Hz, 1H), 2.46 (s, 3H). ^13^C NMR (101 MHz, DMSO) δ 164.34, 155.67, 153.01, 151.92, 150.19, 147.36, 139.83, 134.92, 133.51, 131.60, 130.76, 129.00, 128.51, 126.70, 125.03, 124.80, 124.64, 124.22, 121.78, 121.76, 120.04, 119.11, 116.27, 9.06. HRMS (ESI) *m*/*z* calcd for C_24_H_13_Cl_3_NO_4_S^+^ (M+H)^+^: 515.9631, found: 515.9625.

**2ar**. 3-Chloro-4-(3,4-dichloroisothiazol-5-yl)-8-methyl-2-oxo-2*H*-chromen-7-yl acrylate, white solid; yield: 54%; m.p.: 118–119 °C; ^1^H NMR (400 MHz, Chloroform-*d*) δ 7.08 (d, *J* = 8.7 Hz, 1H), 6.98 (d, *J* = 8.7 Hz, 1H), 6.67 (d, *J* = 17.3 Hz, 1H), 6.36 (dd, *J* = 17.3, 10.5 Hz, 1H), 6.12 (d, *J* = 10.5 Hz, 1H), 2.33 (s, 3H). ^13^C NMR (101 MHz, Chloroform-*d*) δ 163.50, 155.82, 152.33, 151.89, 150.86, 149.56, 138.95, 134.17, 126.83, 123.73, 123.26, 123.09, 120.37, 119.67, 115.73, 9.32. HRMS (ESI) *m*/*z* calcd for C_16_H_9_Cl_3_NO_4_S^+^ (M+H)^+^: 415.9318, found: 415.9310.

**2as**. 3-Chloro-4-(3,4-dichloroisothiazol-5-yl)-8-methyl-2-oxo-2*H*-chromen-7-yl 3-(difluoromethyl)-1-methyl-1*H*-pyrazole-4-carboxylate, white solid; yield: 69%; m.p.: 202–204 °C; ^1^H NMR (400 MHz, Chloroform-*d*) δ 8.17 (s, 1H), 7.15 (d, *J* = 8.7 Hz, 1H), 7.02 (d, *J* = 8.7 Hz, 1H), 4.06 (s, 3H), 2.38 (s, 3H). ^13^C NMR (101 MHz, Chloroform-*d*) δ 159.05, 155.79, 151.85, 150.86, 149.56, 146.95 (t, *J* = 25.6 Hz), 138.95, 136.23, 123.77, 123.26, 123.19, 120.55, 119.80, 115.86, 111.04 (t, *J* = 2.2 Hz), 109.23 (t, *J* = 237.4 Hz), 39.97, 9.33. ^19^F NMR (376 MHz, Chloroform-*d*) δ -115.63. ^19^F NMR (376 MHz, Chloroform-*d*) δ −115.63. HRMS (ESI) *m*/*z* calcd for C_19_H_11_Cl_3_F_2_N_3_O_4_S^+^ (M+H)^+^: 519.9504, found: 519.9496.

**2at**. 3-Chloro-4-(3,4-dichloroisothiazol-5-yl)-8-methyl-2-oxo-2*H*-chromen-7-yl-1-methyl-3-(trifluoromethyl)-1*H*-pyrazole-4-carboxylate, white solid; yield: 77%; m.p.: 220–221 °C; ^1^H NMR (400 MHz, Chloroform-*d*) δ 8.23 (s, 1H), 7.14 (d, *J* = 8.7 Hz, 1H), 7.01 (d, *J* = 8.7 Hz, 1H), 4.07 (s, 3H), 2.37 (s, 3H). ^13^C NMR (101 MHz, Chloroform-*d*) δ 158.03, 155.79, 151.85, 150.85, 149.54, 142.21 (q, *J* = 38.7 Hz), 138.97, 137.44, 123.78, 123.25, 123.17, 120.51, 120.12 (q, *J* = 269.6 Hz), 119.73, 115.86, 111.09, 40.11, 9.26. ^19^F NMR (376 MHz, Chloroform-*d*) δ −62.06. HRMS (ESI) *m*/*z* calcd for C_19_H_10_Cl_3_F_3_N_3_O_4_S^+^ (M+H)^+^: 537.9409, found: 537.9401.

**2au**. 3-Chloro-4-(3,4-dichloroisothiazol-5-yl)-8-methyl-2-oxo-2*H*-chromen-7-yl 2-methylfuran-3-carboxylate, yellow solid; yield: 67%; m.p.: 159–160 °C; ^1^H NMR (400 MHz, Chloroform-*d*) δ 7.33 (d, *J* = 1.8 Hz, 1H), 7.14 (d, *J* = 8.7 Hz, 1H), 7.00 (d, *J* = 8.7 Hz, 1H), 6.79 (d, *J* = 1.7 Hz, 1H), 2.64 (s, 3H), 2.36 (s, 3H). ^13^C NMR (101 MHz, Chloroform-*d*) δ 161.70, 161.35, 155.90, 152.39, 151.95, 150.90, 149.54, 139.02, 123.71, 123.25, 122.98, 120.54, 120.06, 115.64, 111.90, 110.67, 110.59, 14.09, 9.44. HRMS (ESI) *m*/*z* calcd for C_19_H_11_Cl_3_NO_5_S^+^ (M+H)^+^: 469.9423, found: 469.9416.

**2av**. 3-Chloro-4-(3,4-dichloroisothiazol-5-yl)-8-methyl-2-oxo-2*H*-chromen-7-yl 3-methylthiophene-2-carboxylate, white solid; yield: 91%; m.p.: 180–182 °C; ^1^H NMR (400 MHz, Chloroform-*d*) δ 7.59–7.50 (m, 1H), 7.19 (d, *J* = 8.7 Hz, 1H), 7.07–6.94 (m, 2H), 2.59 (s, 3H), 2.39 (s, 3H). ^13^C NMR (101 MHz, Chloroform-*d*) δ 159.95, 155.89, 152.30, 151.96, 150.88, 149.52, 149.33, 139.04, 132.29, 132.20, 124.19, 123.70, 123.25, 122.99, 120.59, 120.06, 115.67, 16.22, 9.49. HRMS (ESI) *m*/*z* calcd for C_19_H_11_Cl_3_NO_4_S_2_^+^ (M+H)^+^: 485.9195, found: 485.9188.

**2ba**. 4-(3,4-Dichloroisothiazol-5-yl)-8-methyl-2-oxo-2*H*-chromen-7-yl 2-chloronicotinate, pale yellow solid; yield 97%; m.p. 197–198 °C. ^1^H NMR (400 MHz, DMSO-*d*_6_) δ 8.71 (dd, *J* = 4.7, 1.8 Hz, 1H), 8.65 (dd, *J* = 7.7, 1.8 Hz, 1H), 7.70 (dd, *J* = 7.7, 4.8 Hz, 1H), 7.44 (d, *J* = 8.7 Hz, 1H), 7.39 (d, *J* = 8.7 Hz, 1H), 6.83 (s, 1H), 2.32 (s, 3H). ^13^C NMR (101 MHz, DMSO-*d*_6_) δ 161.83, 158.42, 154.60, 153.27, 152.12, 151.41, 148.67, 147.12, 142.72, 141.53, 125.03, 124.75, 123.39, 121.42, 119.04, 118.89, 118.05, 115.23, 8.99. HRMS (ESI) *m*/*z* calcd for C_19_H_10_Cl_3_N_2_O_4_S^+^ (M+H)^+^: 466.9427, found: 466.9421.

**2bb**. 4-(3,4-Dichloroisothiazol-5-yl)-8-methyl-2-oxo-2*H*-chromen-7-yl 3-(difluoromethyl)-1-methyl-1*H*-pyrazole-4-carboxylate, pale yellow solid; yield 89%; m.p. 196–198 °C. ^1^H NMR (400 MHz, Chloroform-*d*) δ 8.16 (s, 1H), 7.29–7.23 (m, 1H), 7.15 (d, *J* = 8.7 Hz, 1H), 7.09 (t, *J* = 53.8 Hz, 1H), 6.52 (s, 1H), 4.07 (s, 3H), 2.39 (s, 3H). ^13^C NMR (101 MHz, Chloroform-*d*) δ 159.12, 158.84, 153.76, 153.05, 152.06, 149.57, 147.01 (t, *J* = 25.5 Hz), 142.78, 136.13, 123.99, 122.68, 120.65, 119.06, 117.67, 114.69, 111.21 (t, *J* = 2.4 Hz), 109.20 (t, *J* = 237.4 Hz), 39.98, 9.35. ^19^F NMR (376 MHz, Chloroform-*d*) δ −115.64, −115.78. ^19^F NMR (376 MHz, Chloroform-*d*) δ −115.71. HRMS (ESI) *m*/*z* calcd for C_19_H_12_Cl_2_F_2_N_3_O_4_S^+^ (M+H)^+^: 485.9893, found: 485.9885.

**2bc**. 4-(3,4-Dichloroisothiazol-5-yl)-8-methyl-2-oxo-2*H*-chromen-7-yl 1-methyl-3-(trifluoromethyl)-1*H*-pyrazole-4-carboxylate, white solid; yield 58%; m.p. 163–164 °C. ^1^H NMR (400 MHz, Chloroform-*d*) δ 8.20 (s, 1H), 7.23 (d, *J* = 8.7 Hz, 1H), 7.13 (d, *J* = 8.7 Hz, 1H), 6.50 (s, 1H), 4.06 (s, 3H), 2.37 (s, 3H).^13^C NMR (101 MHz, Chloroform-*d*) δ 158.84, 158.11, 153.77, 153.06, 152.07, 149.57, 142.78, 142.31 (q, *J* = 38.8 Hz), 137.33, 123.98, 122.68, 120.63, 120.12 (q, *J* = 269.7 Hz), 118.97, 117.67, 114.70, 111.32. ^19^F NMR (376 MHz, Chloroform-*d*) δ −62.06. HRMS (ESI) *m*/*z* calcd for C_19_H_11_Cl_2_F_3_N_3_O_4_S^+^ (M+H)^+^: 503.9799, found: 503.9791.

**2bd**. 4-(3,4-Dichloroisothiazol-5-yl)-8-methyl-2-oxo-2*H*-chromen-7-yl 2-methylfuran-3-carboxylate, brown red solid; yield 82%; m.p. 186–188 °C. ^1^H NMR (400 MHz, Chloroform-*d*) δ 7.37 (d, *J* = 1.9 Hz, 1H), 7.25 (d, *J* = 8.7 Hz, 1H), 7.15 (d, *J* = 8.7 Hz, 1H), 6.83 (d, *J* = 1.9 Hz, 1H), 6.51 (s, 1H), 2.68 (s, 3H), 2.40 (s, 3H). ^13^C NMR (101 MHz, Chloroform-*d*) δ 161.60, 161.39, 158.93, 153.88, 153.09, 152.62, 149.54, 142.83, 141.02, 123.86, 122.65, 120.62, 119.28, 117.46, 114.46, 112.00, 110.65, 14.02, 9.40. HRMS (ESI) *m*/*z* calcd for C_19_H_12_Cl_2_NO_5_S^+^ (M+H)^+^: 435.9813, found: 435.9804.

**2be**. 4-(3,4-Dichloroisothiazol-5-yl)-8-methyl-2-oxo-2*H*-chromen-7-yl 3-methylthiophene-2-carboxylate, pale yellow solid; yield 90%; m.p. 188–189 °C. ^1^H NMR (400 MHz, Chloroform-*d*) δ 7.56 (d, *J* = 5.0 Hz, 1H), 7.23 (d, *J* = 8.7 Hz, 1H), 7.18 (d, *J* = 8.7 Hz, 1H), 7.03 (d, *J* = 5.0 Hz, 1H), 6.49 (s, 1H), 2.62 (s, 3H), 2.40 (s, 3H). ^13^C NMR (101 MHz, Chloroform-*d*) δ 160.03, 158.93, 153.88, 153.07, 152.53, 149.55, 149.20, 142.83, 132.22, 132.05, 124.37, 123.83, 122.66, 120.69, 119.28, 117.49, 114.48, 16.19, 9.44. HRMS (ESI) *m*/*z* calcd for C_19_H_12_Cl_2_NO_4_S_2_^+^ (M+H)^+^: 451.9585, found: 451.9577.

**2bf**. 4-(3,4-Dichloroisothiazol-5-yl)-8-methyl-2-oxo-2*H*-chromen-7-yl benzoate, white solid; yield 93%; m.p. 172–173 °C. ^1^H NMR (400 MHz, Chloroform-*d*) δ 8.25 (d, *J* = 7.5 Hz, 2H), 7.72 (t, *J* = 7.3 Hz, 1H), 7.58 (t, *J* = 7.5 Hz, 2H), 7.28 (d, *J* = 7.5 Hz, 1H), 7.20 (d, *J* = 8.6 Hz, 1H), 6.52 (s, 1H), 2.42 (s, 3H). ^13^C NMR (101 MHz, Chloroform-*d*) δ 164.29, 158.89, 153.87, 153.10, 153.01, 149.56, 142.83, 134.24, 130.34, 128.84, 128.48, 123.97, 122.67, 120.62, 119.13, 117.53, 114.56, 9.41. HRMS (ESI) *m*/*z* calcd for C_20_H_12_Cl_2_NO_4_S^+^ (M+H)^+^: 431.9864, found: 431.9854.

**2bg**. 4-(3,4-Dichloroisothiazol-5-yl)-8-methyl-2-oxo-2*H*-chromen-7-yl propionate, white solid; yield 98%; m.p. 111–112 °C. ^1^H NMR (400 MHz, Chloroform-*d*) δ 7.19 (d, *J* = 8.7 Hz, 1H), 7.02 (d, *J* = 8.7 Hz, 1H), 6.47 (s, 1H), 2.68 (q, *J* = 7.5 Hz, 2H), 2.32 (s, 3H), 1.31 (t, *J* = 7.5 Hz, 3H). ^13^C NMR (101 MHz, Chloroform-*d*) δ 172.07, 158.93, 153.85, 153.03, 152.79, 149.54, 142.81, 123.89, 122.65, 120.35, 119.00, 117.45, 114.44, 27.59, 9.27, 9.11. HRMS (ESI) *m*/*z* calcd for C_16_H_12_Cl_2_NO_4_S^+^ (M+H)^+^: 383.9864, found: 383.9854.

**2bh**. 4-(3,4-Dichloroisothiazol-5-yl)-8-methyl-2-oxo-2*H*-chromen-7-yl cyclopropane carboxylate, yellow solid; yield 94%; m.p. 102–104 °C. ^1^H NMR (400 MHz, Chloroform-*d*) δ 7.17 (d, *J* = 8.7 Hz, 1H), 7.01 (d, *J* = 8.7 Hz, 1H), 6.43 (s, 1H), 2.30 (s, 3H), 1.93–1.85 (m, 1H), 1.21–1.15 (m, 2H), 1.11–1.04 (m, 2H). ^13^C NMR (101 MHz, Chloroform-*d*) δ 172.51, 158.84, 153.90, 152.97, 152.79, 149.37, 142.75, 123.84, 122.55, 120.35, 118.99, 117.39, 114.38, 12.81, 9.62, 9.22. HRMS (ESI) *m*/*z* calcd for C_17_H_12_Cl_2_NO_4_S^+^ (M+H)^+^: 395.9864, found: 395.9854.

**2bi**. 4-(3,4-Dichloroisothiazol-5-yl)-8-methyl-2-oxo-2*H*-chromen-7-yl furan-2-carboxylate, white solid; yield 75%; m.p. 195–197 °C. ^1^H NMR (400 MHz, Chloroform-*d*) δ 7.75–7.70 (m, 1H), 7.46 (d, *J* = 3.5 Hz, 1H), 7.24 (d, *J* = 8.8 Hz, 1H), 7.17 (d, *J* = 8.7 Hz, 1H), 6.64 (dd, *J* = 3.5, 1.7 Hz, 1H), 6.50 (s, 1H), 2.39 (s, 3H). ^13^C NMR (101 MHz, Chloroform-*d*) δ 158.83, 155.87, 153.79, 153.07, 152.09, 149.56, 147.85, 143.06, 142.78, 123.98, 122.68, 120.65, 120.45, 118.98, 117.65, 114.70, 112.49, 9.38. HRMS (ESI) *m*/*z* calcd for C_18_H_10_Cl_2_NO_5_S^+^ (M+H)^+^: 421.9656, found: 421.9647.

**2bj**. 4-(3,4-Dichloroisothiazol-5-yl)-8-methyl-2-oxo-2*H*-chromen-7-yl thiophene-2-carboxylate, white solid; yield 84%; m.p. 178–179 °C. ^1^H NMR (400 MHz, Chloroform-*d*) δ 8.04 (dd, *J* = 3.8, 1.1 Hz, 1H), 7.74 (dd, *J* = 5.0, 1.1 Hz, 1H), 7.27–7.22 (m, 2H), 7.19 (d, *J* = 8.7 Hz, 1H), 6.50 (s, 1H), 2.40 (s, 3H). ^13^C NMR (101 MHz, Chloroform-*d*) δ 159.66, 158.95, 153.82, 153.05, 152.48, 149.58, 142.84, 135.50, 134.47, 131.54, 128.39, 123.97, 122.69, 120.67, 119.11, 117.59, 114.62, 9.44. HRMS (ESI) *m*/*z* calcd for C_18_H_10_Cl_2_NO_4_S_2_^+^ (M+H)^+^: 437.9428, found: 437.9426.

**2bk**. 4-(3,4-Dichloroisothiazol-5-yl)-8-methyl-2-oxo-2*H*-chromen-7-yl 2-phenylacetate, yellow solid; yield 59%; m.p. 82–83 °C. ^1^H NMR (400 MHz, Chloroform-*d*) δ 7.42–7.30 (m, 5H), 7.17 (d, *J* = 8.7 Hz, 1H), 7.00 (d, *J* = 8.7 Hz, 1H), 6.44 (s, 1H), 3.93 (s, 2H), 2.18 (s, 3H). ^13^C NMR (101 MHz, Chloroform-*d*) δ 169.12, 158.81, 153.85, 152.97, 152.64, 149.45, 142.71, 132.84, 129.35, 128.90, 127.70, 123.88, 122.60, 120.35, 118.87, 117.53, 114.51, 41.28, 9.06. HRMS (ESI) *m*/*z* calcd for C_21_H_14_Cl_2_NO_4_S^+^ (M+H)^+^: 446.0020, found: 446.0013.

**2bl**. 4-(3,4-Dichloroisothiazol-5-yl)-8-methyl-2-oxo-2*H*-chromen-7-yl 4-methoxybenzoate, yellow solid; yield 53%; m.p. 192–194 °C. ^1^H NMR (400 MHz, Chloroform-*d*) δ 8.17 (d, *J* = 8.9 Hz, 2H), 7.24 (d, *J* = 8.7 Hz, 1H), 7.16 (d, *J* = 8.7 Hz, 1H), 7.01 (d, *J* = 8.9 Hz, 2H), 6.49 (s, 1H), 3.91 (s, 3H), 2.38 (s, 3H). ^13^C NMR (101 MHz, Chloroform-*d*) δ 164.41, 163.99, 158.97, 153.91, 153.19, 153.09, 149.54, 142.86, 132.83, 132.52, 123.88, 122.65, 120.66, 119.26, 117.40, 114.42, 114.12, 55.62, 9.39. HRMS (ESI) *m*/*z* calcd for C_21_H_14_Cl_2_NO_5_S^+^ (M+H)^+^: 461.9969, found: 461.9959.

**2bm**. 4-(3,4-Dichloroisothiazol-5-yl)-8-methyl-2-oxo-2*H*-chromen-7-yl isobutyrate, white solid; yield 75%; m.p. 127–129 °C. ^1^H NMR (400 MHz, Chloroform-*d*) δ 7.19 (d, *J* = 8.7 Hz, 1H), 7.00 (d, *J* = 8.7 Hz, 1H), 6.47 (s, 1H), 2.89 (hept, *J* = 7.0 Hz, 1H), 2.32 (s, 3H), 1.37 (d, *J* = 7.0 Hz, 6H). ^13^C NMR (101 MHz, Chloroform-*d*) δ 174.66, 158.92, 153.86, 153.02, 152.89, 149.53, 142.81, 123.86, 122.63, 120.34, 118.95, 117.41, 114.39, 34.22, 18.96, 9.17. HRMS (ESI) *m*/*z* calcd for C_17_H_14_Cl_2_NO_4_S^+^ (M+H)^+^: 398.0020, found: 398.0010.

**2bn**. 4-(3,4-Dichloroisothiazol-5-yl)-8-methyl-2-oxo-2*H*-chromen-7-yl 4-fluorobenzoate, yellow solid; yield 82%; m.p. 173–174 °C. ^1^H NMR (400 MHz, Chloroform-*d*) δ 8.31–8.24 (m, 2H), 7.30–7.23 (m, 3H), 7.18 (d, *J* = 8.7 Hz, 1H), 6.53 (s, 1H), 2.41 (s, 3H). ^13^C NMR (101 MHz, Chloroform-*d*) δ 166.52 (d, *J* = 256.6 Hz), 163.32, 158.87, 153.81, 153.09, 152.82, 149.58, 142.81, 133.02 (d, *J* = 9.5 Hz), 124.71 (d, *J* = 2.6 Hz), 124.01, 122.68, 120.58, 119.05, 117.60, 116.14 (d, *J* = 22.1 Hz), 114.64, 9.41. ^19^F NMR (376 MHz, Chloroform-*d*) δ −102.94. HRMS (ESI) *m*/*z* calcd for C_20_H_11_Cl_2_FNO_4_S^+^ (M+H)^+^: 449.9770, found: 449.9763.

**2bo**. 4-(3,4-Dichloroisothiazol-5-yl)-8-methyl-2-oxo-2*H*-chromen-7-yl 4-(trifluoromethyl)-benzoate, yellow solid; yield: 98%; m.p. 175–177 °C. ^1^H NMR (400 MHz, Chloroform-*d*) δ 8.39 (d, *J* = 8.1 Hz, 2H), 7.86 (d, *J* = 8.2 Hz, 2H), 7.31 (d, *J* = 9.0 Hz, 1H), 7.21 (d, *J* = 8.7 Hz, 1H), 6.55 (s, 1H), 2.42 (s, 3H). ^13^C NMR (101 MHz, Chloroform-*d*) δ 163.15, 158.78, 153.75, 153.10, 152.55, 149.61, 142.78, 135.66 (q, *J* = 32.8 Hz), 131.71, 130.76, 125.91 (q, *J* = 4.0 Hz), 123.42 (q, *J* = 271.3 Hz), 124.13, 122.72, 120.52, 118.89, 117.78, 114.82, 9.45. ^19^F NMR (376 MHz, Chloroform-*d*) δ −63.23. HRMS (ESI) *m*/*z* calcd for C_21_H_11_Cl_2_F_3_NO_4_S^+^ (M+H)^+^: 499.9738, found: 499.9729.

**2bp**. 4-(3,4-Dichloroisothiazol-5-yl)-8-methyl-2-oxo-2*H*-chromen-7-yl pivalate, yellow solid; yield 89%; m.p. 156–158 °C. ^1^H NMR (400 MHz, Chloroform-*d*) δ 7.19 (d, *J* = 8.7 Hz, 1H), 6.98 (d, *J* = 8.7 Hz, 1H), 6.47 (s, 1H), 2.32 (s, 3H), 1.42 (s, 9H). ^13^C NMR (101 MHz, Chloroform-*d*) δ 176.16, 158.94, 153.88, 153.14, 153.01, 149.54, 142.82, 123.83, 122.63, 120.36, 118.91, 117.37, 114.32, 39.42, 27.16, 9.11. HRMS (ESI) *m*/*z* calcd for C_18_H_16_Cl_2_NO_4_S^+^ (M+H)^+^: 412.0177, found: 412.0170.

**2bq**. 4-(3,4-Dichloroisothiazol-5-yl)-8-methyl-2-oxo-2*H*-chromen-7-yl 3-bromobenzoate, yellow solid; yield 84%; m.p. 203–204 °C. ^1^H NMR (400 MHz, Chloroform-*d*) δ 8.34 (s, 1H), 8.14 (d, *J* = 7.8 Hz, 1H), 7.80 (d, *J* = 8.0 Hz, 1H), 7.43 (t, *J* = 7.9 Hz, 1H), 7.24 (d, *J* = 3.2 Hz, 1H), 7.14 (d, *J* = 8.7 Hz, 1H), 6.50 (s, 1H), 2.37 (s, 3H). ^13^C NMR (101 MHz, Chloroform-*d*) δ 163.06, 158.87, 153.77, 153.08, 152.64, 149.59, 142.81, 137.22, 133.24, 130.43, 130.35, 128.91, 124.10, 122.91, 122.71, 120.59, 118.97, 117.70, 114.75, 9.48. HRMS (ESI) *m*/*z* calcd for C_20_H_11_BrCl_2_NO_4_S^+^ (M+H)^+^: 509.8969, found: 509.8962.

**2br**. 4-(3,4-Dichloroisothiazol-5-yl)-8-methyl-2-oxo-2*H*-chromen-7-yl 2,4-difluorobenzoate, yellow solid; yield 84%; m.p. 200–202 °C. ^1^H NMR (400 MHz, Chloroform-*d*) δ 8.20 (q, *J* = 8.4 Hz, 1H), 7.28 (d, *J* = 7.9 Hz, 1H), 7.20 (d, *J* = 8.7 Hz, 1H), 7.12–6.97 (m, 2H), 6.53 (s, 1H), 2.42 (s, 3H). ^13^C NMR (101 MHz, Chloroform-*d*) δ 166.64 (dd, *J* = 259.0, 12.0 Hz), 163.43 (dd, *J* = 265.2, 12.8 Hz), 161.10 (d, *J* = 4.4 Hz), 158.83, 153.80, 153.09, 152.46, 149.57, 142.78, 134.60 (d, *J* = 10.7 Hz), 123.99, 122.68, 120.50, 118.96, 117.68, 114.71, 113.57 (dd, *J* = 9.7, 3.6 Hz), 112.24 (dd, *J* = 21.7, 3.8 Hz), 105.77 (t, *J* = 25.6 Hz), 9.41. ^19^F NMR (376 MHz, Chloroform-*d*) δ −98.91 (d, *J* = 12.9 Hz), −101.54 (d, *J* = 13.6 Hz). HRMS (ESI) *m*/*z* calcd for C_20_H_10_Cl_2_F_2_NO_4_S^+^ (M+H)^+^: 467.9675, found: 467.9667.

**2bs**. 4-(3,4-Dichloroisothiazol-5-yl)-8-methyl-2-oxo-2*H*-chromen-7-yl 1-naphthoate, yellow solid; yield 77%; m.p. 201–203 °C. ^1^H NMR (400 MHz, Chloroform-*d*) δ 9.03 (d, *J* = 8.6 Hz, 1H), 8.56 (d, *J* = 7.3 Hz, 1H), 8.16 (d, *J* = 8.1 Hz, 1H), 7.95 (d, *J* = 8.1 Hz, 1H), 7.63 (dq, *J* = 20.9, 6.7, 6.1 Hz, 3H), 7.33–7.14 (m, 2H), 6.50 (s, 1H), 2.45 (s, 3H). ^13^C NMR (101 MHz, Chloroform-*d*) δ 164.68, 158.92, 153.89, 153.16, 153.11, 149.57, 142.84, 135.16, 134.00, 131.81, 131.67, 128.88, 128.61, 126.68, 125.50, 124.55, 124.49, 124.02, 122.69, 120.81, 119.32, 117.53, 114.59, 9.56. HRMS (ESI) *m*/*z* calcd for C_24_H_14_Cl_2_NO_4_S^+^ (M+H)^+^: 482.0020, found: 482.0011.

**2bt**. 4-(3,4-Dichloroisothiazol-5-yl)-8-methyl-2-oxo-2*H*-chromen-7-yl cinnamate, yellow solid; yield 58%; m.p. 174–175 °C. ^1^H NMR (400 MHz, Chloroform-*d*) δ 7.93 (d, *J* = 16.0 Hz, 1H), 7.68–7.56 (m, 2H), 7.50–7.38 (m, 3H), 7.23 (d, *J* = 8.7 Hz, 1H), 7.12 (d, *J* = 8.7 Hz, 1H), 6.68 (d, *J* = 16.0 Hz, 1H), 6.48 (s, 1H), 2.38 (s, 3H). ^13^C NMR (101 MHz, Chloroform-*d*) δ 164.51, 158.95, 153.88, 153.09, 152.89, 149.55, 148.03, 142.83, 133.80, 131.19, 129.12, 128.48, 123.92, 122.67, 120.57, 119.09, 117.46, 115.97, 114.50, 9.41. HRMS (ESI) *m*/*z* calcd for C_22_H_14_Cl_2_NO_4_S^+^ (M+H)^+^: 458.0020, found: 458.0010.

**2bu**. 4-(3,4-Dichloroisothiazol-5-yl)-8-methyl-2-oxo-2*H*-chromen-7-yl 4-chlorobenzoate, yellow solid; yield 77%; m.p. 185–187 °C. ^1^H NMR (400 MHz, Chloroform-*d*) δ 8.19 (d, *J* = 8.6 Hz, 2H), 7.55 (d, *J* = 8.6 Hz, 2H), 7.28 (d, *J* = 5.8 Hz, 1H), 7.18 (d, *J* = 8.7 Hz, 1H), 6.53 (s, 1H), 2.40 (s, 3H). ^13^C NMR (101 MHz, Chloroform-*d*) δ 163.48, 158.84, 153.80, 153.09, 152.74, 149.58, 142.80, 140.92, 131.68, 129.25, 126.89, 124.03, 122.69, 120.56, 119.00, 117.64, 114.68, 9.42. HRMS (ESI) *m*/*z* calcd for C_20_H_11_Cl_3_NO_4_S^+^ (M+H)^+^: 465.9474, found: 465.9468.

**2bv**. 4-(3,4-Dichloroisothiazol-5-yl)-8-methyl-2-oxo-2*H*-chromen-7-yl acrylate, white solid; yield 68%; m.p. 142–144 °C. ^1^H NMR (400 MHz, Chloroform-*d*) δ 7.21 (d, *J* = 8.7 Hz, 1H), 7.07 (d, *J* = 8.7 Hz, 1H), 6.67 (d, *J* = 18.0 Hz, 1H), 6.47 (s, 1H), 6.36 (dd, *J* = 17.3, 10.5 Hz, 1H), 6.11 (d, *J* = 11.3 Hz, 1H), 2.32 (s, 3H). ^13^C NMR (101 MHz, Chloroform-*d*) δ 163.56, 158.85, 153.83, 153.03, 152.56, 149.49, 142.78, 134.00, 126.93, 123.94, 122.63, 120.43, 118.92, 117.53, 114.55, 9.30. HRMS (ESI) *m*/*z* calcd for C_16_H_10_Cl_2_NO_4_S^+^ (M+H)^+^: 381.9707, found: 381.9697.

### 3.3. X-ray Diffraction

The crystal structure of compound **2be** was cultured using the fusion method. Using a hot stage to heat the compound until it melted, the temperature was then meticulously controlled just below the melting point during the slow crystal growth process. The crystal structure was determined using a Rigaku Saturn 724 CCD diffractometer equipped with graphite-monochromatic Mo Kα radiation (λ = 0.71073 Å) at 113 K. The atomic coordinates for **2be** were deposited in the Cambridge Crystallographic Data Centre (CCDC).

### 3.4. In Vitro Fungicidal Activity

The fungicidal activity of compounds **2** against *A. solani* (A. s), *B. cinerea* (B. c), *C. arachidicola* (C. a), *Fusarium graminearum* (F. g), *P. piricola* (P. p), *R. solani* (R. s) and *S. sclerotiorum* (S. s) were evaluated in vitro at a concentration of 50 μg/mL according to the mycelium growth inhibition method. Pure drugs of osthole, isotianil and compounds **1a** and **1b** were chosen as positive controls. The median effective concentration (EC_50_) of the active target compounds was also assessed according to the mycelium growth inhibition method. To prepare the test solution, a highly concentrated mother solution of the test substance was carefully created at a concentration of 25,000 μg/mL using *N, N*-dimethylformamide as the solvent. A precise amount of the mother solution was added to sterile water containing 0.1% Tween 80 to create a test solution with a concentration of 500 μg/mL. This solution was then mixed with potato dextrose agar (PDA) to create a culture with a controlled concentration of 50 μg/mL of the test compound. A blank control was also included using pure DMF without the target compound. To ensure accuracy, three replicates were included for each treatment. The EC_50_ was determined using seven concentration gradients ranging from 1.56 to 100 μg/mL, and advanced DPS software was used to process the data.

### 3.5. In Vivo Fungicidal Activity

The in vivo activity of compounds **2** against *Pseudoperonospora cubensis* on cucumber was evaluated at Shenyang Sinochem Agrochemicals R&D Co., Ltd., Shenyang, China. Pure osthole and cyzofamid were used as a positive control. The compounds with an inhibition rate of 100% were chosen for further tests with a concentration gradient. To prepare the test solution, 10 mg of the compound to be tested was accurately weighed and dissolved in 0.5 mL of *N*, *N*-dimethylformamide. The resulting solution was then diluted with distilled water containing 1% Tween 80 to create a stock concentration of 500 μg/mL. Potted plants with uniform growth were carefully selected as test plants, and the stock solution was further diluted with distilled water to the desired test concentration. The test solution was then sprayed onto the surface of the plant and allowed to dry for 2 h to allow for solvent evaporation. After 24 h, the treated test colonies were properly inoculated on the leaves of the plant for three replicates. A blank control was also included, which involved spraying the plants with plain distilled water containing no test solution. The treated plants were then cultured normally in the greenhouse, and the antifungal efficacy was assessed after 7 d.

### 3.6. 3D-QSAR Molecular Modelling

Next, 3D-QSAR studies were carried out with 29 target compounds (listed in Table 7) with fungicidal activity against *P. piricola*. The construction of the 3D-QSAR model was completed in the CoMFA modules in SYBYL X 2.1.1. All the structures were constructed using the molecular fragment library of the SYBYL software. The Powell method was used in the optimization and conformation search process. The gradients were limited to 0.005 kJ·mol^−1^, and the maximum number of iterations was 1000. The selected small-molecular force field was the Tripos force field, and the charge carried by the molecule was the Gasteiger–Hückel charge. The potent compound **2aj** was chosen as a template molecule. The biological activity was expressed in terms of D (Table 7) using the following formula [29]:

$$D=\log_{10}\left[\frac{a}{1-a}\right]+\log_{10}M_{w}$$

where *a* is the percentage inhibition against *P. piricola* at 50 μg/mL in vitro, *M_w_* is the molecular weight of the target compounds, and *D* is the biological activity index.

### 3.7. Density Functional Theory (DFT) Calculation

Based on their fungicidal activity, the highly active compound **2ai**, the slightly active compound **2aq** and the lead compound **1a** were selected for molecular orbital calculations using Gaussian 09W and drawn in Gaussview 6.0. The structures were preprocessed and geometrically optimized, and frequency calculations were carried out to generate geometrically stable structures using the DFT-B3LYP/6-31G (d) method. Single-point energy calculations were then performed using the DFT-B3LYP/6-31G (d, p) method. 

## 4. Conclusions

A series of new coumarin derivatives were designed and synthesized. The bioassay results indicated that compound **2ai** exhibited broad-spectrum fungicidal in vitro activity, and compounds **2ar** and **2bg** showed good fungicidal activity against *P. cubensis* in vivo. Overall, we successfully adjusted the solubility of this series of compounds via esterification, and the activity was equivalent to that of the lead compound. The 3D-QSAR analysis showed that a larger group and positively charged group at the 7-position of the coumarin ring and a larger group at the 3-position of the coumarin ring were beneficial for improving fungicidal activity against *P. piricola*, the results of this study provide guidance for further coumarin lead based fungicide development.

## Figures and Tables

**Table 1:**
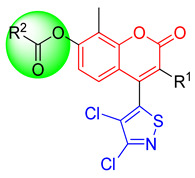
Structures of target compounds **2aa–2bv**.

Compd.	R^1^	R^2^	Compd.	R^1^	R^2^	Compd.	R^1^	R^2^	Compd.	R^1^	R^2^
**2aa**	Cl	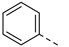	**2al**	Cl	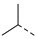	**2ba**	H	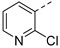	**2bl**	H	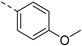
**2ab**	Cl	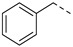	**2am**	Cl	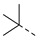	**2bb**	H	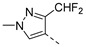	**2bm**	H	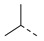
**2ac**	Cl	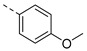	**2an**	Cl	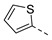	**2bc**	H	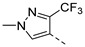	**2bn**	H	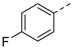
**2ad**	Cl	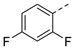	**2ao**	Cl	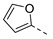	**2bd**	H	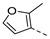	**2bo**	H	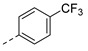
**2ae**	Cl	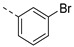	**2ap**	Cl	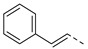	**2be**	H	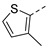	**2bp**	H	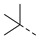
**2af**	Cl	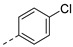	**2aq**	Cl	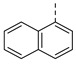	**2bf**	H	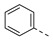	**2bq**	H	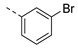
**2ag**	Cl	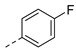	**2ar**	Cl	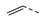	**2bg**	H	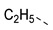	**2br**	H	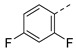
**2ah**	Cl	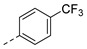	**2as**	Cl	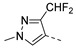	**2bh**	H	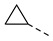	**2bs**	H	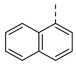
**2ai**	Cl	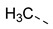	**2at**	Cl	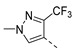	**2bi**	H	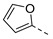	**2bt**	H	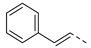
**2aj**	Cl	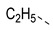	**2au**	Cl	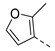	**2bj**	H	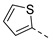	**2bu**	H	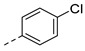
**2ak**	Cl	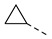	**2av**	Cl	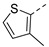	**2bk**	H	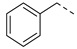	**2bv**	H	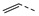

**Table 2:** In vitro fungicidal activity of target compounds **2** against the test phytopathogens.

Compd.	*A. s*	*B. c*	*C. a*	*F. g*	*P. p*	*R. s*	*S. s*	Compd.	*A. s*	*B. c*	*C. a*	*F. g*	*P. p*	*R. s*	*S. s*
**2aa**	4 ± 2	8 ± 1	0	0	39 ± 3	0	7 ± 2	**2bc**	7 ± 1	0	14 ± 3	0	9 ± 2	0	27 ± 2
**2ab**	12 ± 2	16 ± 1	4 ± 2	6 ± 2	18 ± 3	0	16 ± 2	**2bd**	7 ± 1	7 ± 2	13 ± 2	0	23 ± 2	0	11 ± 0
**2ac**	6 ± 1	4 ± 1	0	0	10 ± 3	0	6 ± 2	**2be**	4 ± 1	7 ± 3	0	0	10 ± 0	0	12 ± 2
**2ad**	7 ± 1	5 ± 3	7 ± 0	0	62 ± 3	0	6 ± 2	**2bf**	7 ± 1	8 ± 2	5 ± 2	0	31 ± 2	0	4 ± 2
**2ae**	6 ± 1	0	7 ± 3	0	3 ± 0	0	4 ± 2	**2bg**	63 ± 3	69 ± 2	50 ± 2	23 ± 1	56 ± 0	29 ± 1	63 ± 2
**2af**	3 ± 1	0	4 ± 2	0	10 ± 0	0	12 ± 1	**2bh**	42 ± 3	44 ± 2	35 ± 3	19 ± 3	41 ± 3	25 ± 3	45 ± 3
**2ag**	4 ± 1	0	9 ± 2	0	0	0	11 ± 1	**2bi**	4 ± 1	0	0	0	14 ± 2	0	5 ± 0
**2ah**	5 ± 2	4 ± 2	0	0	4 ± 0	0	8 ± 1	**2bj**	0	16 ± 2	10 ± 3	9 ± 3	23 ± 2	0	10 ± 2
**2ai**	77 ± 2	76 ± 0	86 ± 2	20 ± 2	69 ± 2	46 ± 3	76 ± 0	**2bk**	0	15 ± 2	13 ± 2	9 ± 1	24 ± 0	0	14 ± 3
**2aj**	71 ± 1	68 ± 0	76 ± 2	26 ± 1	78 ± 2	35 ± 3	63 ± 2	**2bl**	0	0	11 ± 2	0	19 ± 2	0	0
**2ak**	38 ± 2	30 ± 0	22 ± 2	0	29 ± 2	0	24 ± 2	**2bm**	26 ± 3	49 ± 2	26 ± 2	18 ± 2	63 ± 2	10 ± 2	52 ± 3
**2al**	23 ± 2	26 ± 1	17 ± 0	15 ± 2	77 ± 0	24 ± 3	27 ± 3	**2bn**	0	10 ± 2	9 ± 2	6 ± 2	28 ± 3	0	10 ± 2
**2am**	0	8 ± 1	11 ± 2	8 ± 2	54 ± 3	0	14 ± 0	**2bo**	0	5 ± 1	0	0	10 ± 0	0	4 ± 2
**2an**	0	7 ± 3	13 ± 2	11 ± 3	10 ± 3	0	11 ± 2	**2bp**	6 ± 2	13 ± 1	14 ± 0	14 ± 0	35 ± 2	0	8 ± 3
**2ao**	44 ± 2	43 ± 1	48 ± 2	23 ± 2	68 ± 0	0	35 ± 3	**2bq**	0	0	5 ± 2	0	8 ± 2	0	4 ± 2
**2ap**	0	0	0	0	24 ± 3	0	0	**2br**	0	0	0	0	8 ± 2	0	4 ± 2
**2aq**	0	0	0	0	16 ± 2	0	9 ± 2	**2bs**	0	4 ± 1	0	0	18 ± 3	0	6 ± 2
**2ar**	66 ± 1	70 ± 0	77 ± 2	28 ± 2	77 ± 2	76 ± 1	62 ± 1	**2bt**	8 ± 3	8 ± 1	5 ± 2	0	12 ± 2	0	8 ± 3
**2as**	25 ± 3	23 ± 1	19 ± 2	0	23 ± 2	0	28 ± 2	**2bu**	0	10 ± 2	6 ± 0	0	5 ± 0	0	11 ± 0
**2at**	17 ± 2	17 ± 1	4 ± 2	12 ± 3	23 ± 3	0	29 ± 3	**2bv**	30 ± 2	17 ± 0	32 ± 3	16 ± 1	53 ± 0	18 ± 3	17 ± 0
**2au**	17 ± 0	12 ± 1	15 ± 2	10 ± 3	24 ± 0	0	18 ± 2	1a *	70 ± 2	85 ± 2	84 ± 3	77 ± 1	93 ± 2	98 ± 1	78 ± 2
**2av**	0	0	5 ± 2	0	17 ± 3	0	14 ± 3	1b *	72 ± 3	83 ± 1	79 ± 3	80 ± 1	91 ± 2	83 ± 2	65 ± 1
**2ba**	5 ± 2	8 ± 3	4 ± 2	8 ± 2	17 ± 2	0	6 ± 2	osthole *	14 ± 1	58 ± 1	10 ± 1	17 ± 1	15 ± 1	70 ± 2	33 ± 1
**2bb**	7 ± 1	16 ± 2	15 ± 2	6 ± 0	18 ± 0	0	12 ± 2	isotianil *	25 ± 2	9 ± 2	20 ± 1	22 ± 1	24 ± 1	45 ± 1	18 ± 2

A. s, Alternaria solani; B. c, Botrytis cinerea; C. a, Cercospora arachidicola; F.g, Fusarium graminearum; P. p, Physalospora piricola; R. s, Rhizoctonia solani; S. s, Sclerotinia sclerotiorum. * Data from [1]. The drugs used for testing are pure drugs.

**Table 3:** The in vitro fungicidal EC_50_ values of the selected compounds.

Compd.	Fungi	Regression Equation	R^2^	EC_50_ (μg/mL)	95% Confidence Interval
**2ai**	*A. s.*	*y* = 4.6354 + 0.7213*x*	0.9671	3.20	2.03~5.06
	*B. c.*	*y* = 4.7569 + 0.5255*x*	0.9738	2.90	1.90~4.43
	*C. a.*	*y* = 4.4115 + 1.0116*x*	0.9528	3.82	2.49~5.85
	*P. p.*	*y* = 4.6117 + 0.6906*x*	0.9799	3.65	2.77~4.81
	*S. s.*	*y* = 4.2996 + 0.9405*x*	0.9704	5.56	4.15~7.44
**2aj**	*A. s.*	*y* = 3.7955 + 0.9777*x*	0.9643	17.06	13.42~21.68
	*B. c.*	*y* = 3.4733 + 1.1522*x*	0.9555	21.14	14.13~31.63
	*C. a.*	*y* = 4.2600 + 1.0479*x*	0.9752	5.08	4.00~6.47
	*P. p.*	*y* = 4.1607 + 0.7510*x*	0.9502	13.11	8.86~19.39
	*S. s.*	*y* = 4.5100 + 0.7458*x*	0.9773	4.54	3.75~5.49
**2ar**	*A. s.*	*y* = 3.6741 + 1.2509*x*	0.9886	11.48	9.54~13.82
	*B. c.*	*y* = 3.3040 + 1.5643*x*	0.9530	12.14	9.29~15.86
	*C. a.*	*y* = 3.9396 + 1.3025*x*	0.9580	6.52	5.07~8.38
	*P. p.*	*y* = 3.6776 + 1.2105*x*	0.9635	12.37	9.76~15.68
	*R. s.*	*y* = 4.3684 + 1.1211*x*	0.9641	3.66	2.78~4.82
	*S. s.*	*y* = 3.8039 + 1.1299*x*	0.9740	11.44	9.42~13.90
**2bg**	*A. s.*	*y* = 4.2795 + 0.6054*x*	0.9624	15.49	12.15~19.76
	*B. c.*	*y* = 4.2014 + 0.7378*x*	0.9866	12.09	10.50~13.92
	*S. s.*	*y* = 4.6013 + 0.5269*x*	0.9827	5.71	4.75~6.87
1a *	*A. s.*	*y* = 4.2794 + 0.9061*x*	0.9696	6.24	4.79~8.12
	*B. c.*	*y* = 4.7981 + 0.7129*x*	0.9716	1.92	1.39~2.65
	*C. a.*	*y* = 4.2191 + 1.0934*x*	0.9676	5.18	3.68~6.95
	*P. p.*	*y* = 4.2839 + 0.7369*x*	0.9691	9.37	5.85~15.03
	*R. s.*	*y* = 4.9015 + 0.8031*x*	0.9718	1.33	0.93~1.90
	*S. s.*	*y* = 4.4350 + 1.0010*x*	0.9586	3.67	2.72~4.95
1b *	*A. s.*	*y* = 3.3158 + 1.7602*x*	0.9841	9.05	7.78~10.53
	*B. c.*	*y* = 4.2055 + 1.0298*x*	0.9734	5.90	4.82~7.24
	*C. a.*	*y* = 3.5992 + 1.5235*x*	0.9555	8.31	6.46~10.68
	*P. p.*	*y* = 3.9657 + 1.3588*x*	0.9018	5.77	3.15~10.58
	*R. s.*	*y* = 3.9626 + 1.3553*x*	0.9932	5.83	5.31~6.39
	*S. s.*	*y* = 3.8143 + 1.1971*x*	0.9499	9.78	7.00~13.67
Osthole *	*B. c.*	*y* = 3.5371 + 1.6848*x*	0.9899	7.38	6.50~8.39
	*P. p.*	*y* = 0.5260 + 2.3891*x*	0.9727	74.59	68.27~85.63
	*R. s.*	*y* = 3.7903 + 1.3147*x*	0.9948	8.32	7.59~9.12
	*S. s.*	*y* = 2.8079 + 1.3590*x*	0.9264	41.03	27.86~64.46

A. s, Alternaria solani; B. c, Botrytis cinerea; C. a, Cercospora arachidicola; F.g, Fusarium graminearum; P. p, Physalospora piricola; R. s, Rhizoctonia solani; S. s, Sclerotinia sclerotiorum. * Data from [1]. The drugs used for testing are pure drugs.

**Table 4:** In vivo fungicidal activity of the target compounds against CDM * (%).

Compd.	200 μg/mL	100 μg/mL	50 μg/mL 2 L	25 μg/mL 10 μg/mL
**2ai**	100	100	30 40	0 nd
**2aj**	100	100	0 75	0 nd
**2ar**	100	100	80 0	75 nd
**2bg**	100	100	88 20	65 nd
1a **	100	100	88 nd	65 nd
1b **	100	100	75 100	20 100
**Osthole**	100	0	// 100	// nd
**Cyazofamid**	100	100	100	100

* CDM: *P. cubensis* (Berk. & Curt.) Rostov. // = not detected. ** Data from [1]. The drugs used for testing are pure drugs.

**Table 5:** Statistical parameters of the CoMFA.

Statistical Parameter	CoMFA
q^2^	0.797
r^2^	0.979
ONC	5
SEE	0.096
F	166.514
Steric	0.754
Electrostatic	0.246

**Table 6:** Frontier orbital energy.

Compd.	*E*_HOMU_ (Hartree)	*E*_LUMU_ (Hartree)	Δ*E* (Hartree/eV)
**2ai**	−0.24774	−0.09462	0.15312/4.17
**2aq**	−0.23359	−0.09296	0.14063/3.83
**1a**	−0.23646	−0.08930	0.14716/4.00

**Table 7:** In vitro biological activity, the biological activity index and experimental and predicted pEC_50_ values of target compounds against *Physalospora piricola*.

Compd.	Inhibition (%)	D	CoMFA	
			Pred. *	Res. **
**2ac**	10	1.74	1.70	0.04
**2ae**	3	1.22	1.22	0.00
**2af**	10	1.74	1.70	0.04
**2ah**	4	1.35	1.45	−0.10
**2ai**	69	2.95	2.99	−0.03
**2aj**	78	3.17	3.20	−0.03
**2al**	77	3.16	3.07	0.09
**2am**	54	2.72	2.70	0.02
**2ar**	77	3.14	2.98	0.16
**2as**	23	2.19	2.24	−0.05
**2at**	23	2.21	2.20	0.01
**2au**	24	2.17	2.13	0.04
**2av**	17	2.00	1.95	0.05
**2ba**	17	1.98	1.97	0.01
**2bc**	9	1.70	1.71	−0.01
**2bd**	23	2.11	2.15	−0.04
**2be**	10	1.70	1.74	−0.04
**2bi**	14	1.84	1.77	0.07
**2bm**	63	2.83	2.69	0.14
**2bo**	10	1.74	1.69	0.05
**2bp**	35	2.35	2.54	−0.19
**2bq**	8	1.65	1.64	0.01
**2br**	8	1.61	1.62	−0.01
**2bv**	53	2.63	2.84	−0.21
2an ***	10	1.71	1.91	−0.20
2bg ***	56	2.68	2.85	−0.17
2bh ***	41	2.43	2.89	−0.46
2bs ***	18	2.02	1.72	0.30
2bu ***	5	1.38	1.55	−0.17

*: Predicted with CoMFA; **: relative error between the D and predicted pEC_50_; ***: test set.

## Data Availability

Data are listed within the article or Supplementary Materials.

## Supplementary Materials

The following supporting information can be downloaded at: https://www.mdpi.com/article/10.3390/molecules28135205/s1. Crystal data and structure refinement for compound **2be**; ^1^H NMR, ^13^C NMR and ^19^F NMR spectral and HRMS of title compounds; In vivo fungicidal activity of the compounds **2ai**, **2aj**, **2ar** and **2bg**.

## Sample Availability

Samples are available on reasonable request from the authors.
