# Supplementary material for: Design, Synthesis and Fungicidal Activity of Ester Derivatives of 4-(3,4-Dichloroisothiazole) 7-Hydroxy Coumarin
Source: Molecules. 2023 Jul 4;28(13):5205. doi: 10.3390/molecules28135205 (PMC10343298; doi:10.3390/molecules28135205)
Supplement: Supplementary file 1 [file molecules-28-05205-s001.zip › molecules-2457223-supplementary.pdf]

Supporting Information for

Design, synthesis and fungicidal activity of ester derivatives of 4-(3,4-dichloroisothiazole) 7-hydroxy coumarin

Kun Li[†,‡], *Yue Zhang*[†,‡], *Zeyu Hong*[†,‡], *Zhenwu Yu*[†,‡], *Xiaoyu Liu*[†,‡], *Zhihong Duan*[†,‡], *Wei Gao*[†,‡], *Liangfu Tang*[†,‡,], *You Lv*[§,]*, *Zhijin Fan*[†,‡]*

[†] *State Key Laboratory of Elemento-Organic Chemistry, College of Chemistry, Nankai University, Tianjin 300071, China*

[§]*College of Agricultural and Biological Engineering, Heze University, Heze, Shandong 274015, China*

[‡] *Frontiers Science Center for New Organic Matter, College of Chemistry, Nankai University, Tianjin 300071, China*

[*] Address correspondence to these authors at State Key Laboratory of Elemento-Organic Chemistry, College of Chemistry, Nankai University, Frontiers Science Center for New Organic Matter, College of Chemistry, No. 94, Weijin Road, Nankai District, Tianjin 300071, P. R. China (Tel: +86-13920714666; Fax: +86 022-23503620; e-mail: fanzj@nankai.edu.cn for Zhijin Fan) or at College of Agricultural and Biological Engineering, Heze University, No. 2269, Daxue Road, Mudan District, Heze, Shandong 274015, P.R. China. (e-mail: lvyou@hezeu.edu.cn)

Table Contents for the Supporting Information

1. Crystal data and structure refinement for compound **2be**.. **3**

2. ^{1}H NMR, ^{13}C NMR and ^{19}F NMR spectral and HRMS of title compounds **4**

3. *In vivo* fungicidal activities of the compounds **2ai**, **2aj**, **2ar** and **2bg**. **75**

Table S1. Crystal data and structure refinement for compound **2be**.

Compounds	**2be**
Empirical formula	$C_{19}H_{11}Cl_2NO_4S_2$
Formula weight	452.31
Temperature/K	113.15
Crystal system	monoclinic
Space group	$P2_1/c$
a/Å	16.9633(6)
b/Å	7.2717(3)
c/Å	14.8314(6)
α/°	90
β/°	90.164(4)
γ/°	90
Volume/Å^3	1829.48(12)
Z	4
ρ_{calc}g/cm^3	1.642
μ/mm^{-1}	0.611
F (000)	920.0
Crystal size/mm^3	0.24 × 0.2 × 0.16
Radiation	MoKα (λ = 0.71073)
2Θ range for data collection/°	4.802 to 65.856
Index ranges	$-25 \leq h \leq 25$, $-10 \leq k \leq 10$, $-22 \leq l \leq 22$
Reflections collected	22211
Independent reflections	6253 [R_{int} = 0.0636, R_{sigma} = 0.0683]
Data/restraints/parameters	6253/268/312
Goodness-of-fit on F^2	1.044
Final *R* indexes [I ≥ 2σ (I)]	R_1 = 0.0690, wR_2 = 0.1642
Final *R* indexes [all data]	R_1 = 0.1030, wR_2 = 0.1967
Largest diff. peak/hole / e Å^{-3}	1.29/−1.02

^{1}H NMR, ^{13}C NMR and ^{19}F NMR spectral and HRMS of title compounds

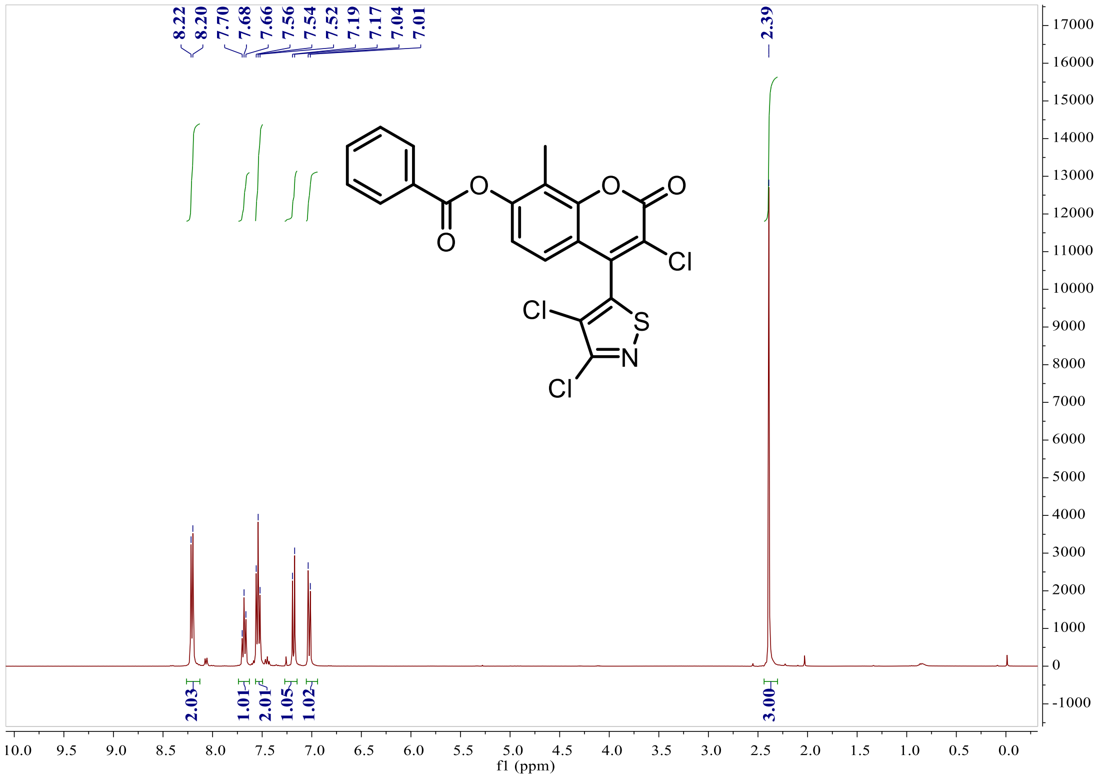

Figure S1. **The ^{1}H NMR (400MHz, Chloroform-*d*) of compound 2aa.**

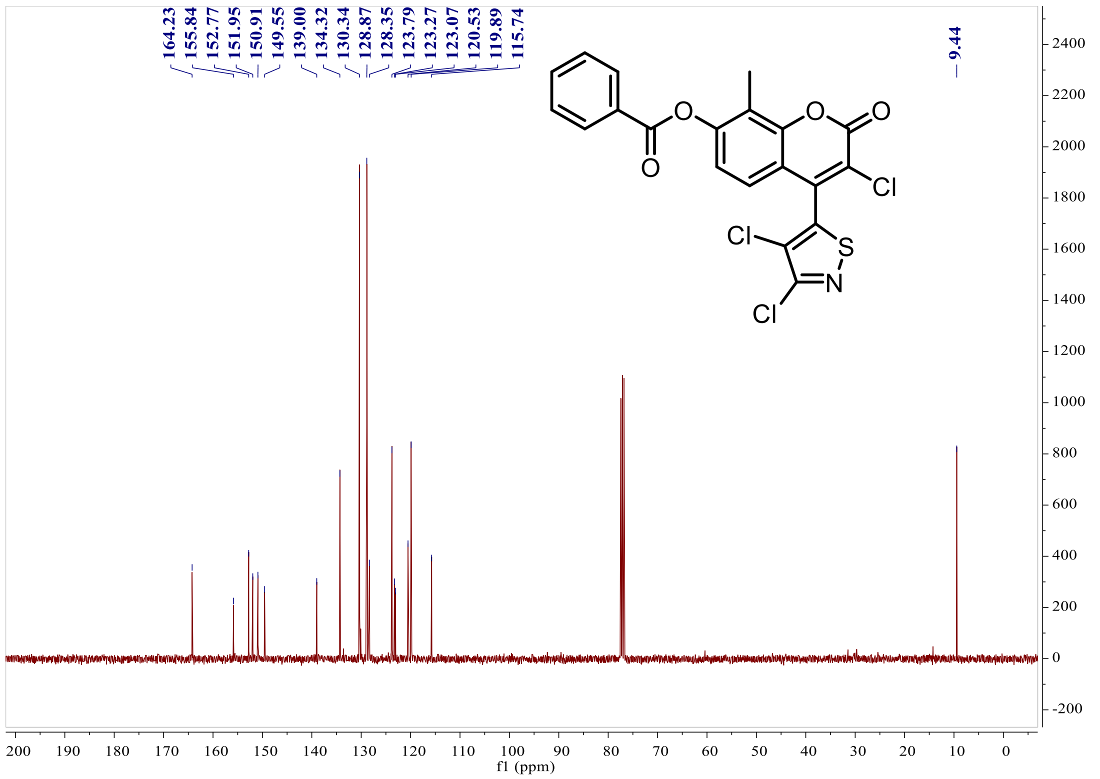

Figure S2. **The ^{13}C NMR (101MHz, Chloroform-*d*) of compound 2aa.**

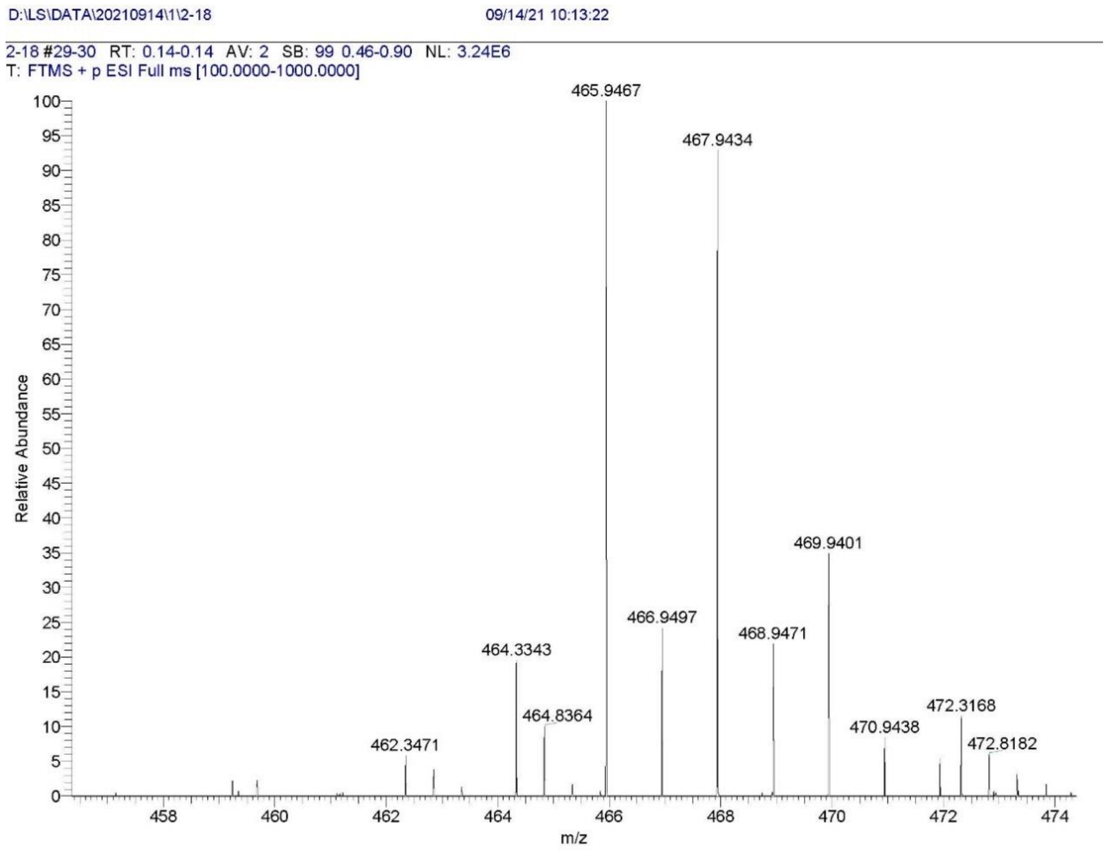

Figure S3. **The HRMS of compound 2aa.**

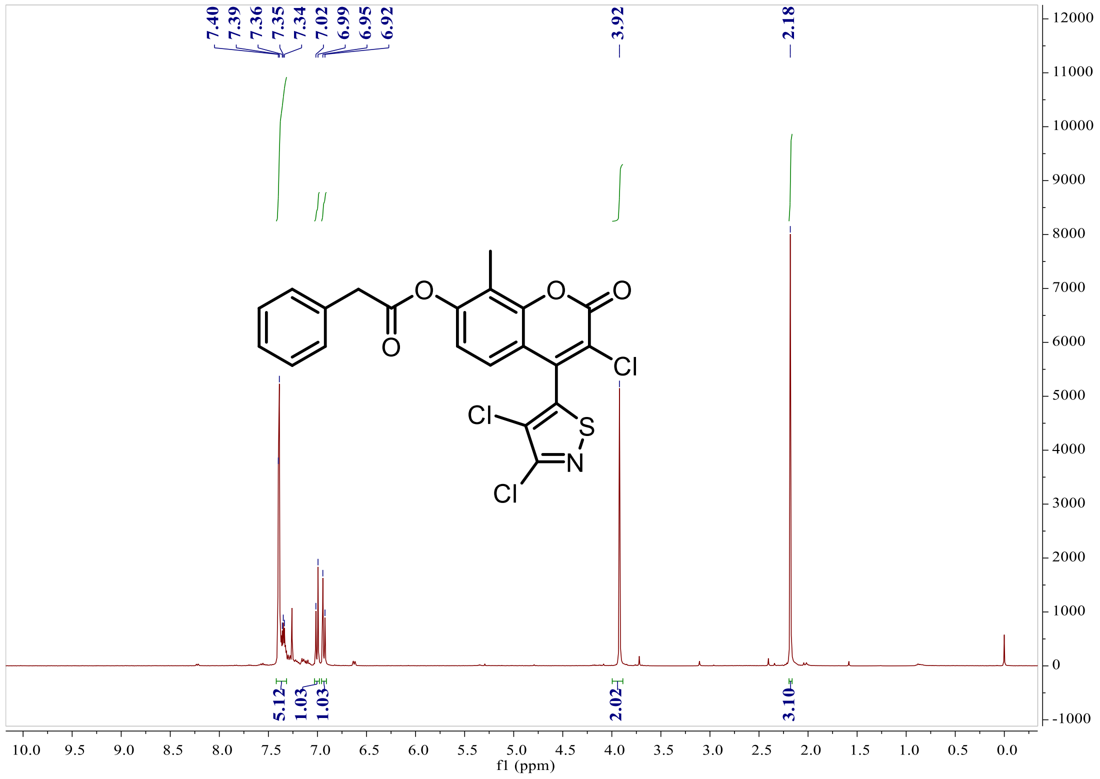

Figure S4. **The** ^{1}**H NMR (400MHz, Chloroform-*d*) of compound 2ab.**

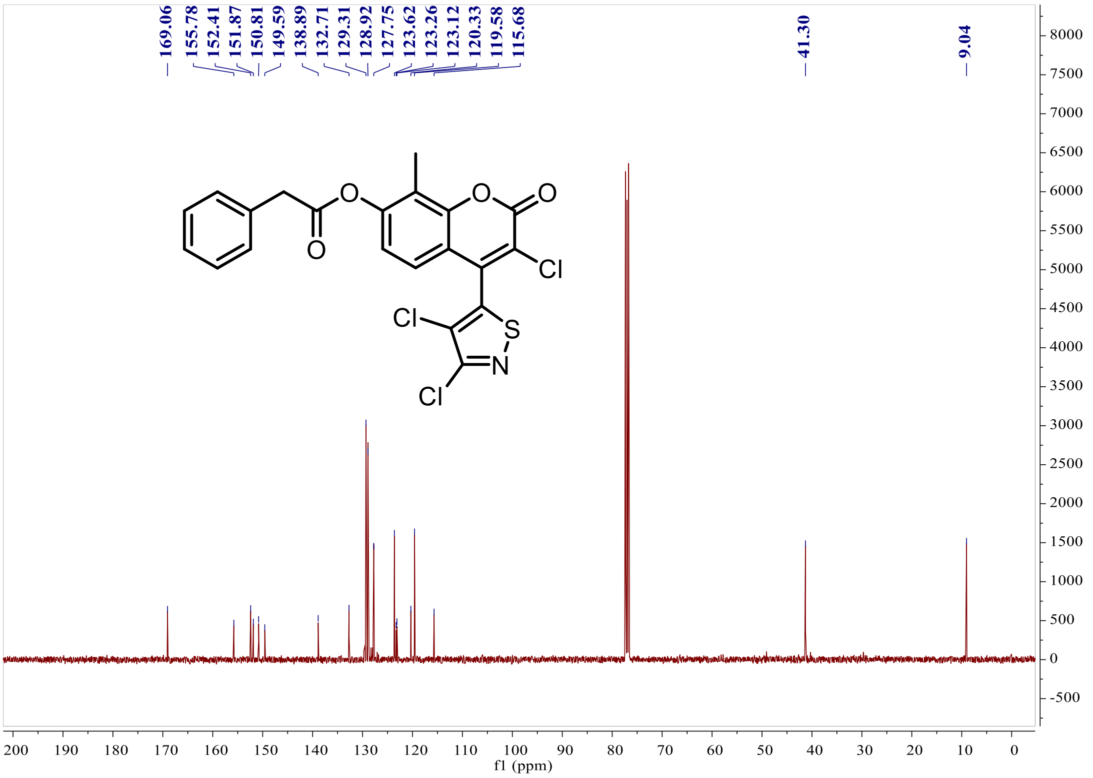

Figure S5. **The ^{13}C NMR (101MHz, Chloroform-*d*) of compound 2ab.**

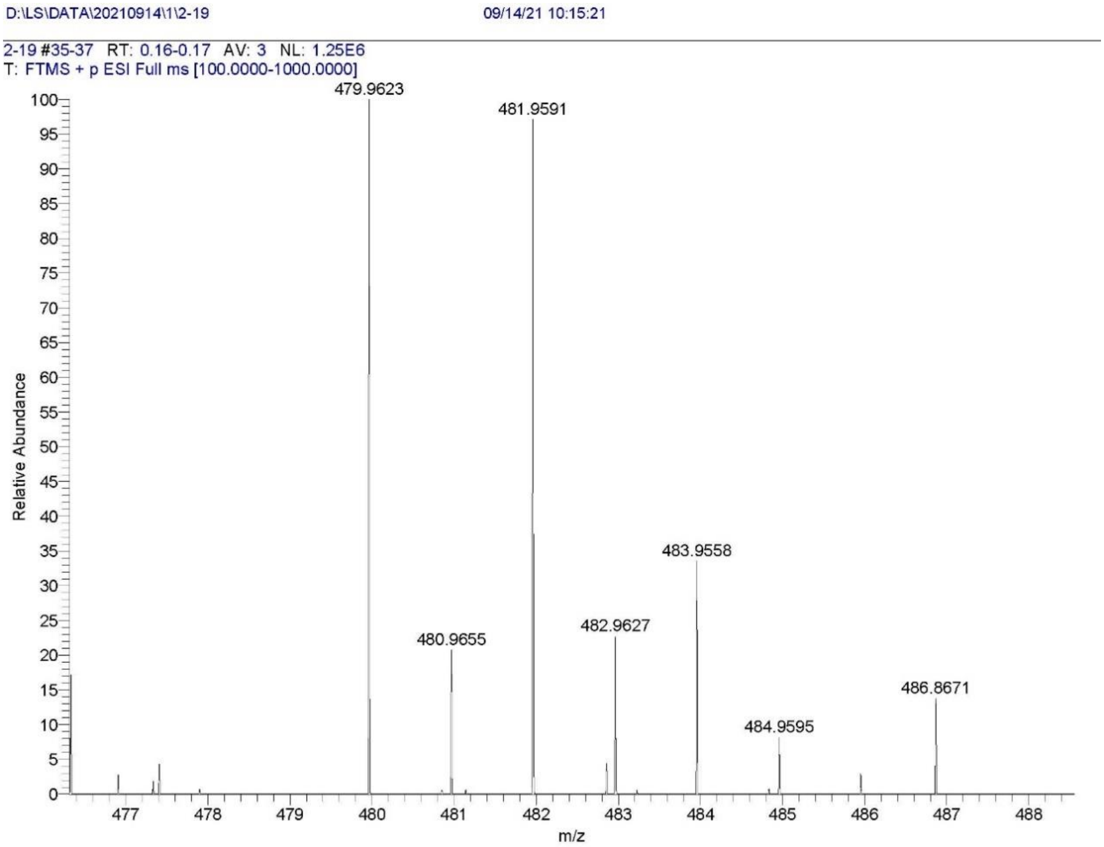

Figure S6. **The HRMS of compound 2ab.**

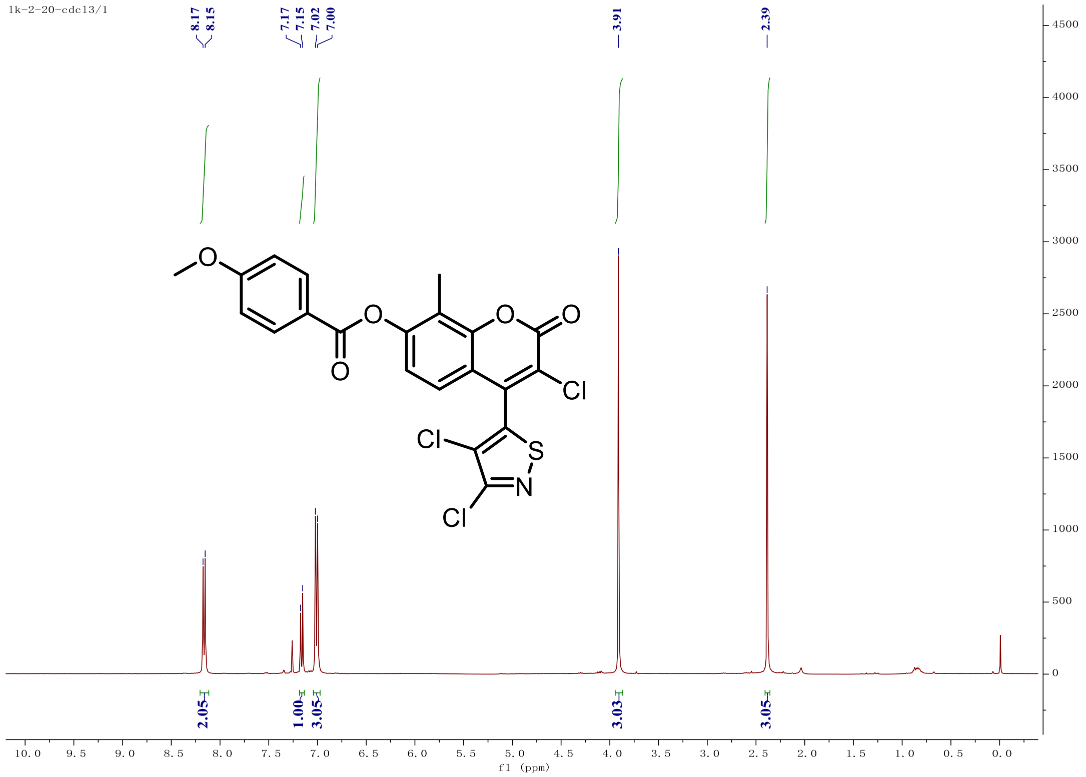

Figure S7. **The ^{1}H NMR (400MHz, Chloroform-*d*) of compound 2ac.**

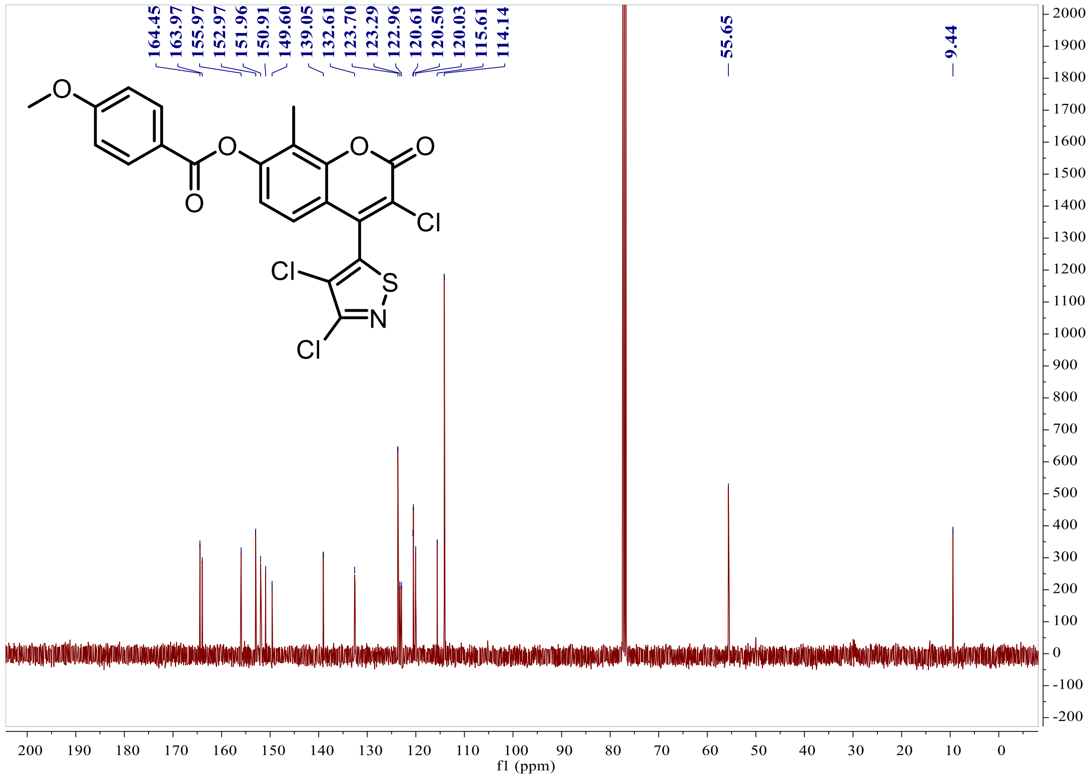

Figure S8. **The ^{13}C NMR (101MHz, Chloroform-*d*) of compound 2ac.**

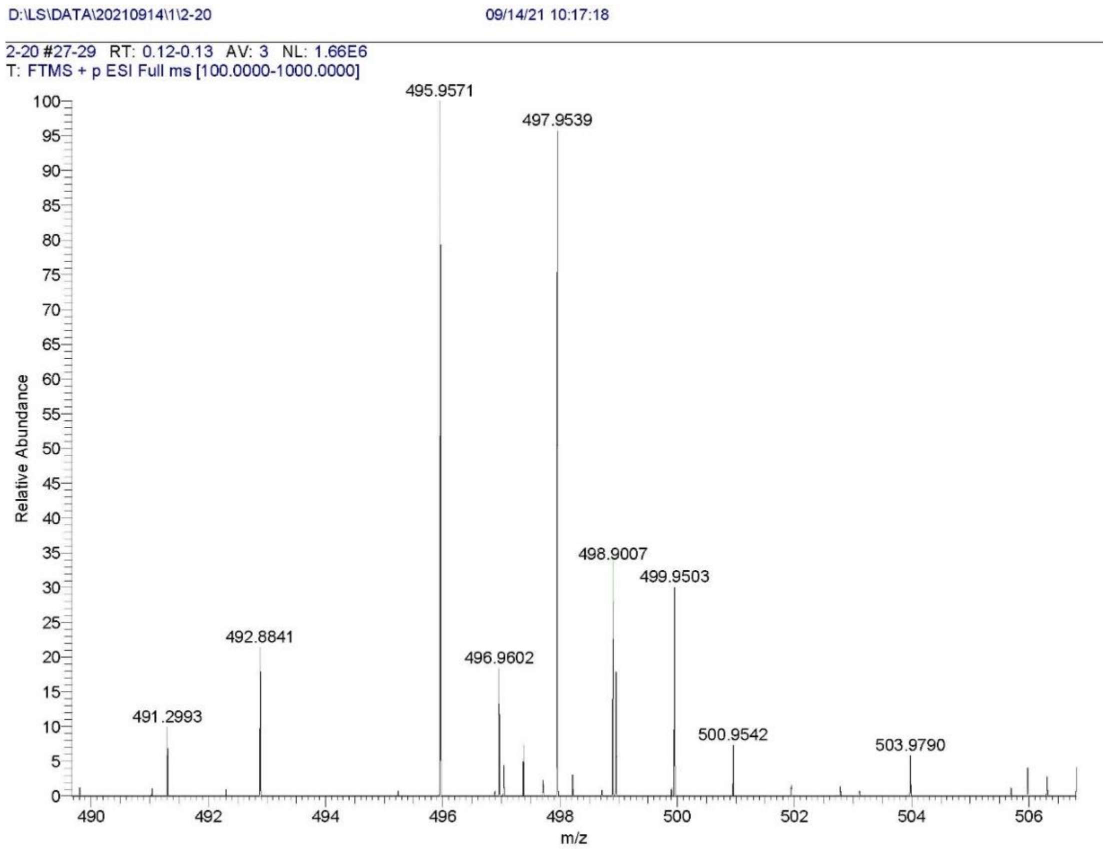

Figure S9. **The HRMS of compound 2ac.**

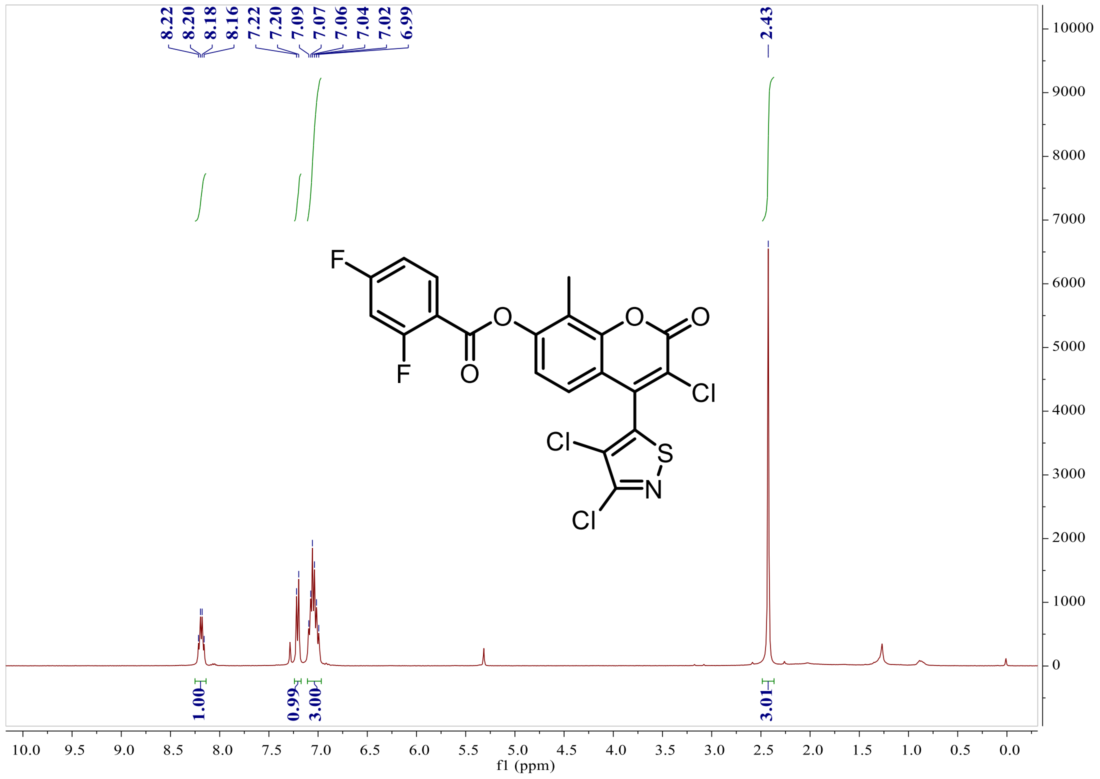

Figure S10. **The ^{1}H NMR (400MHz, Chloroform-*d*) of compound 2ad**

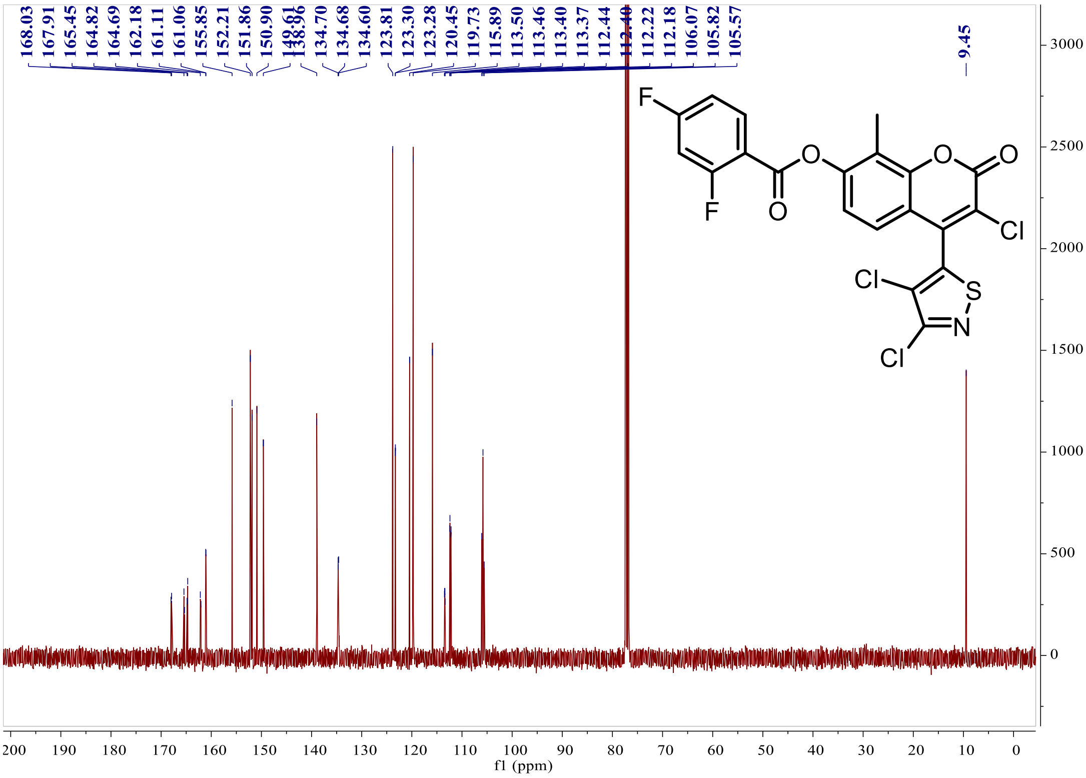

Figure S11. **The ^{13}C NMR (101MHz, Chloroform-*d*) of compound 2ad**

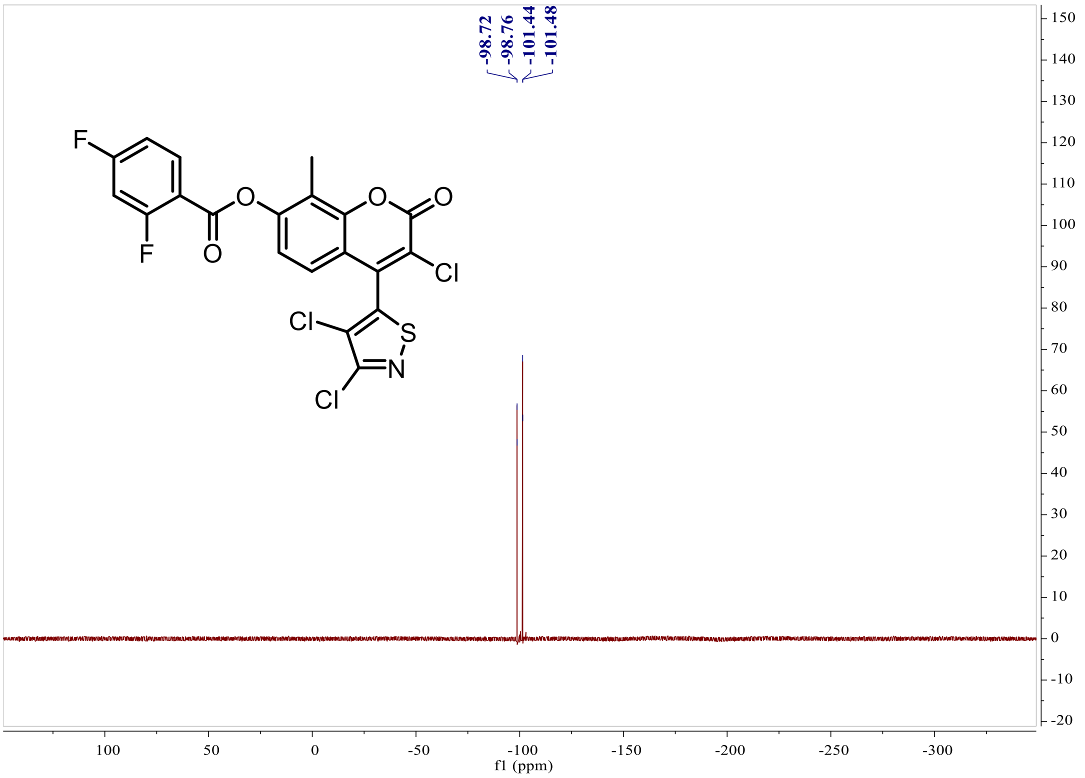

Figure S12. **The ^{19}F NMR (376MHz, Chloroform-*d*) of compound 2ad**

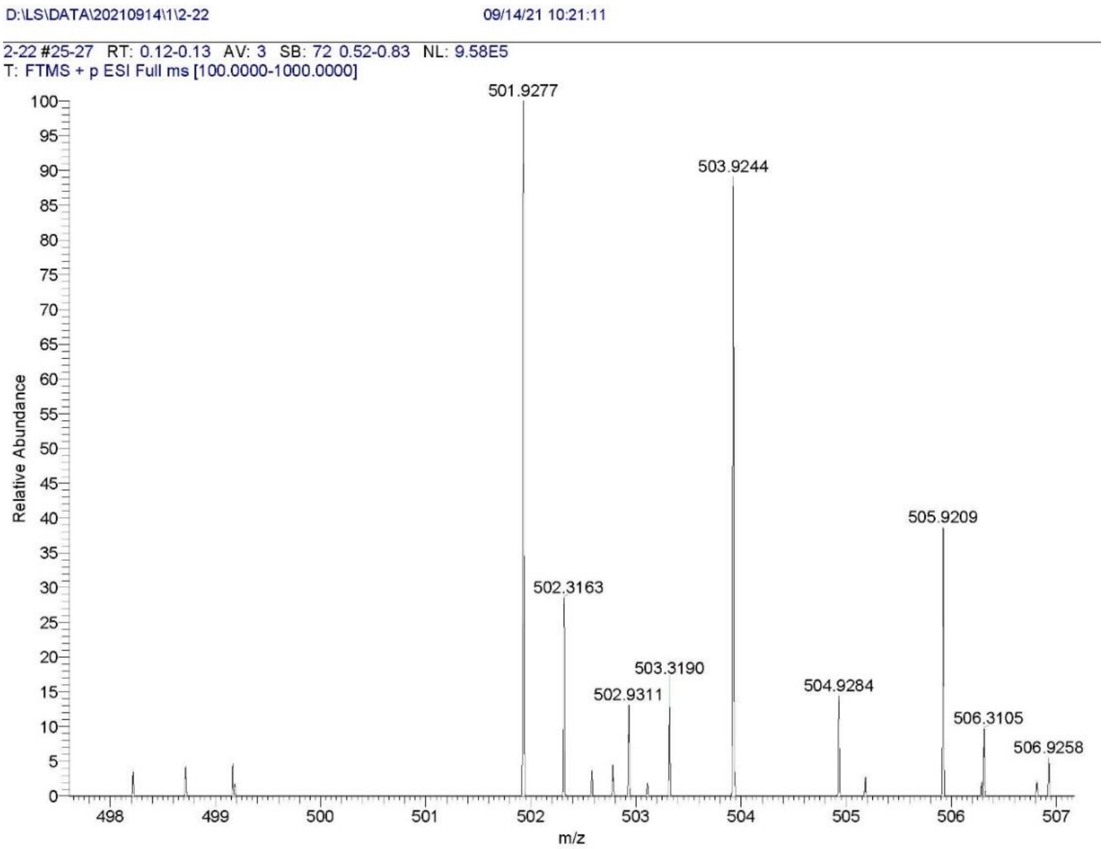

Figure S13. **The HRMS of compound 2ad.**

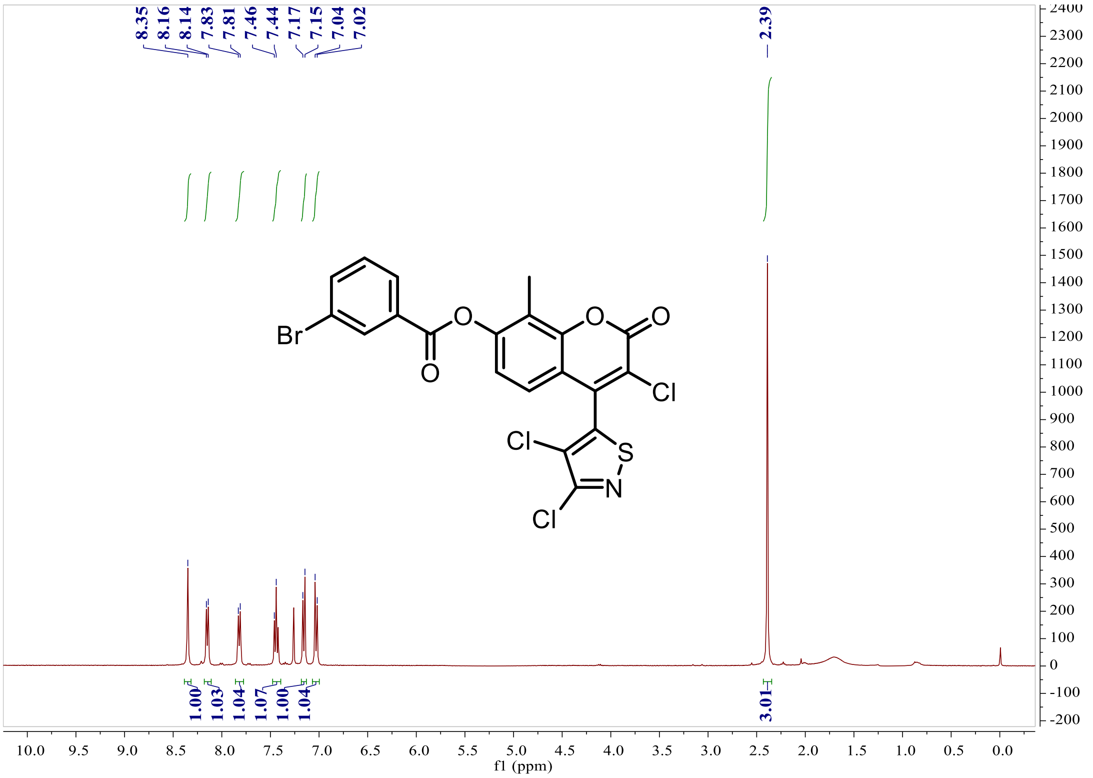

Figure S14. **The ^{1}H NMR (400MHz, Chloroform-*d*) of compound 2ae**

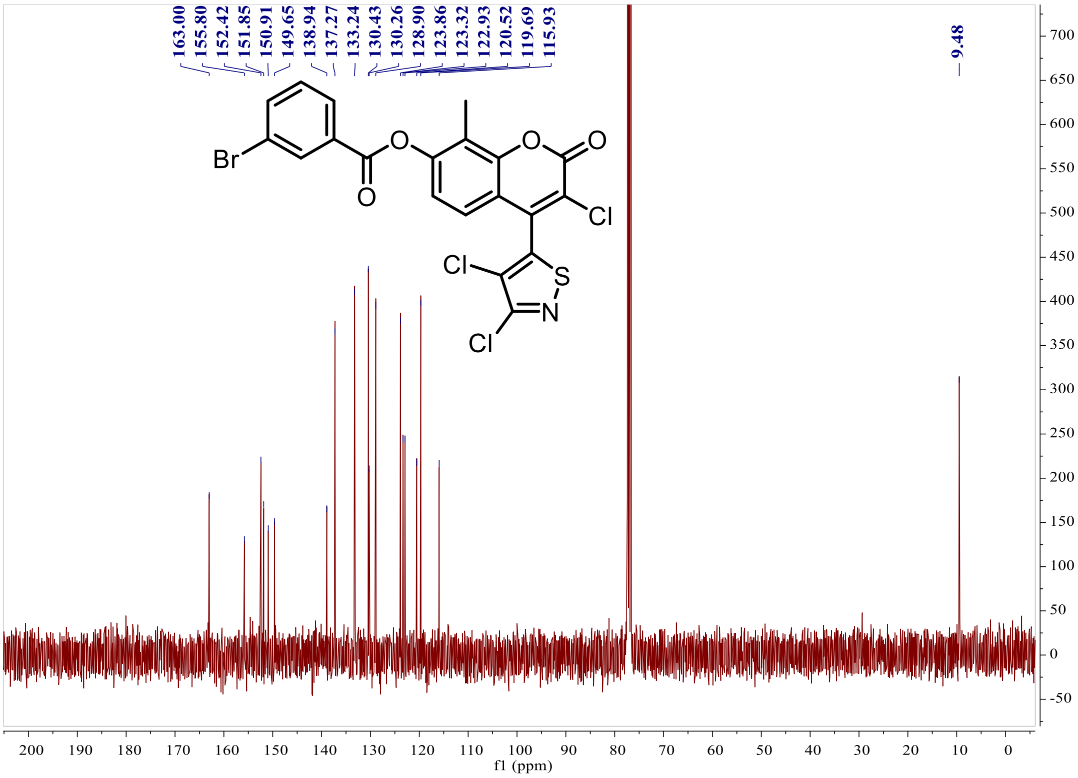

Figure S15. **The ^{13}C NMR (101MHz, Chloroform-*d*) of compound 2ae**

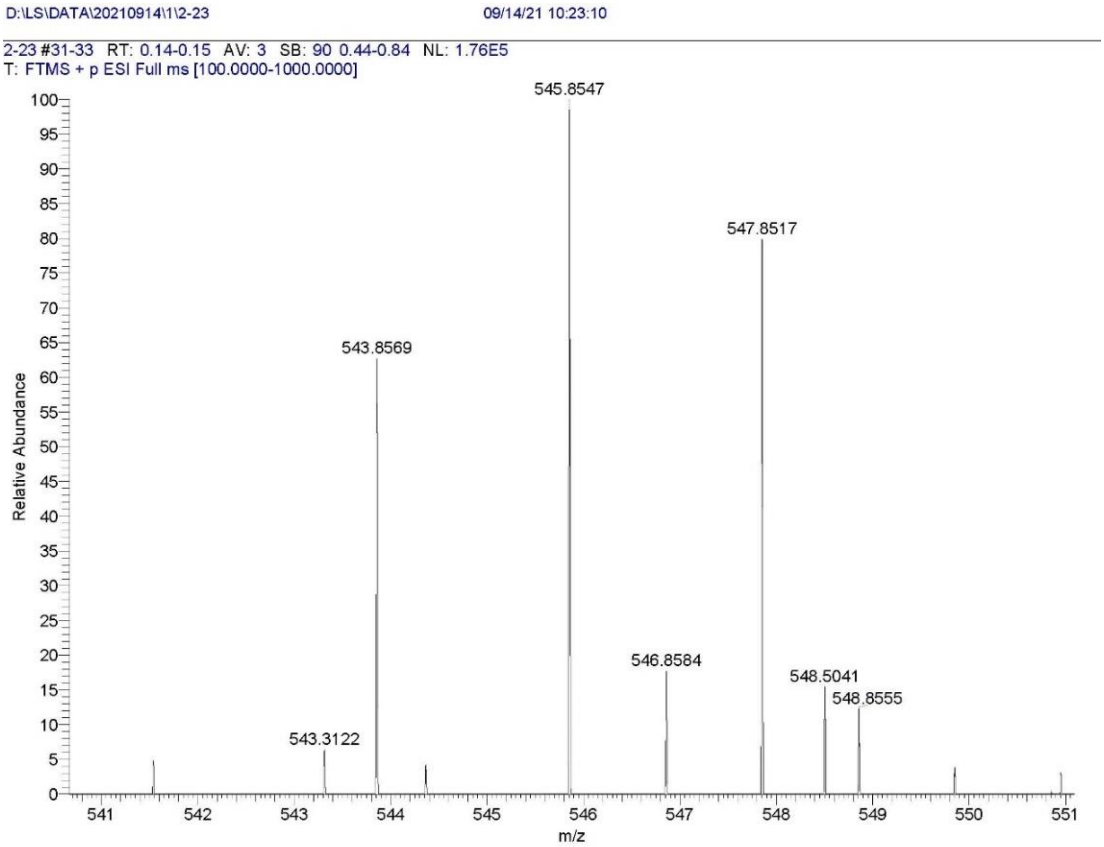

Figure S16. **The HRMS of compound 2ae.**

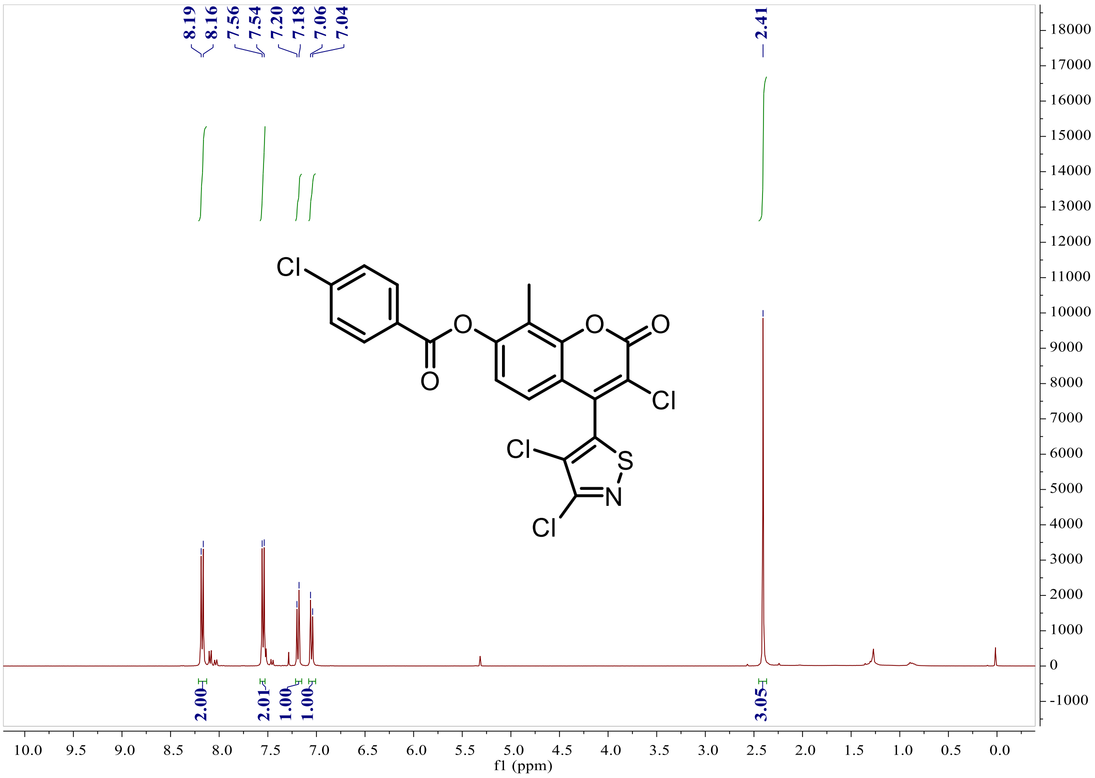

Figure S17. **The ^{1}H NMR (400MHz, Chloroform-*d*) of compound 2af**

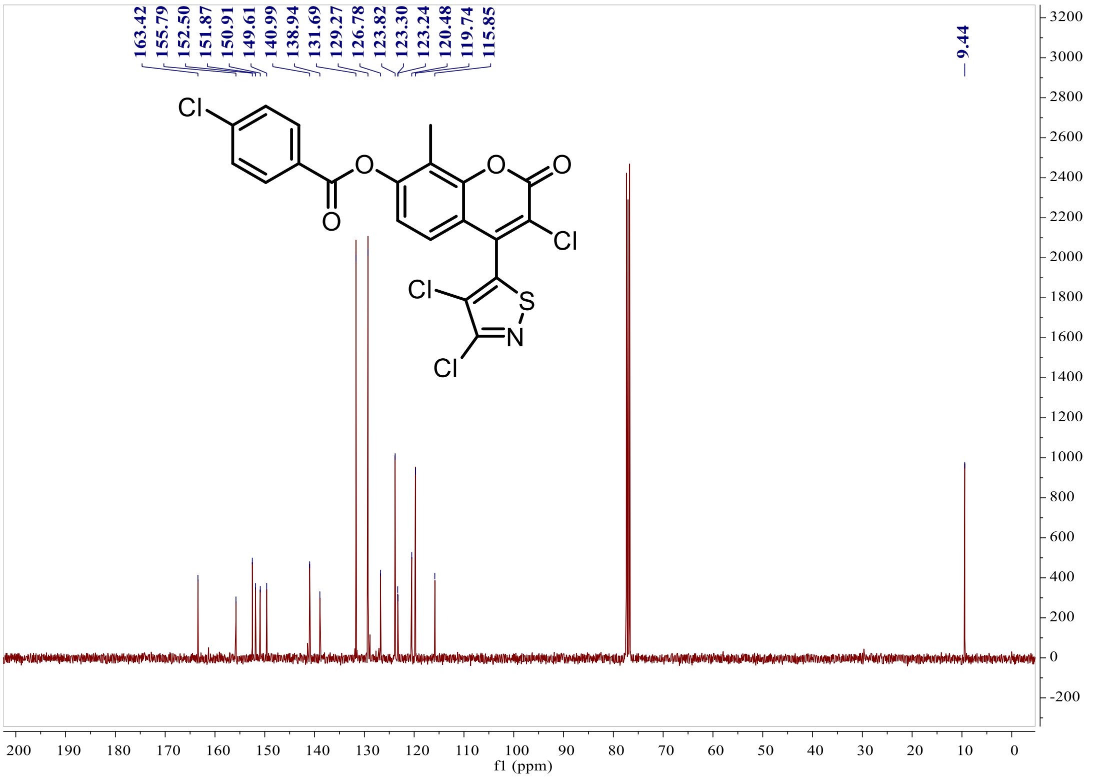

Figure S18. **The ^{13}C NMR (101MHz, Chloroform-*d*) of compound 2af**

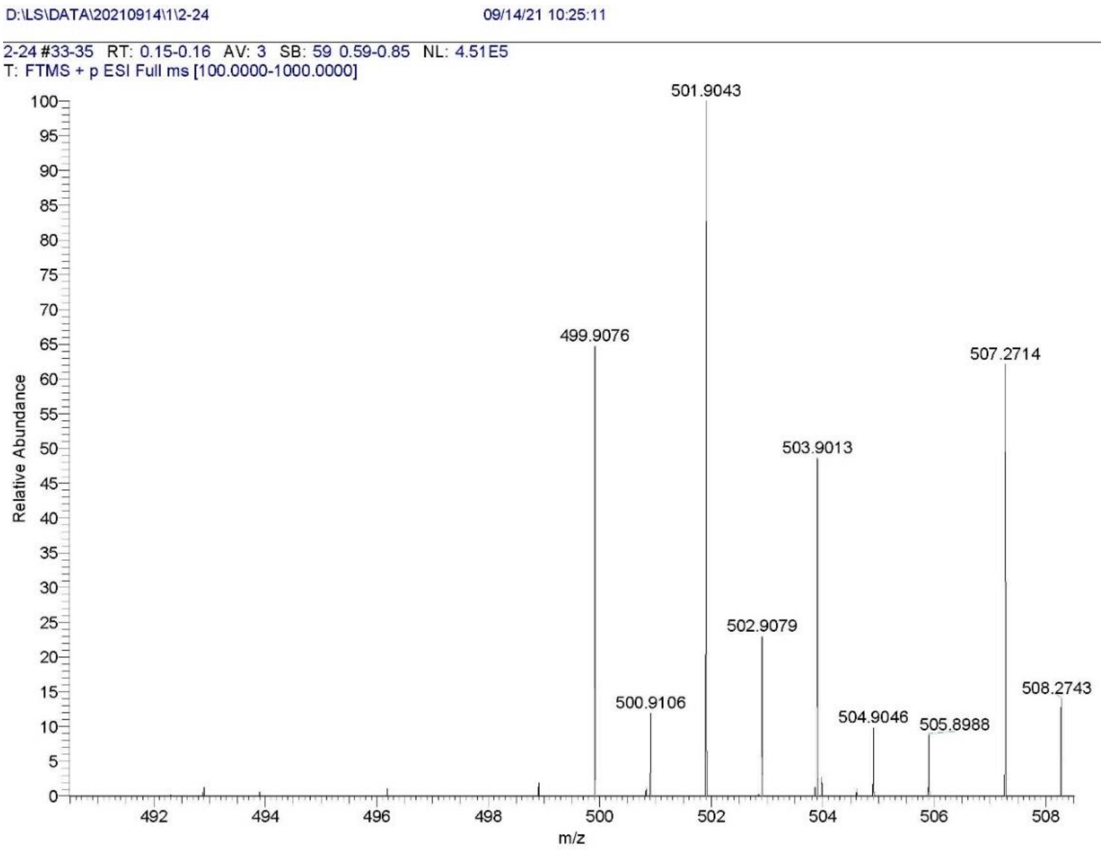

Figure S19. **The HRMS of compound 2af.**

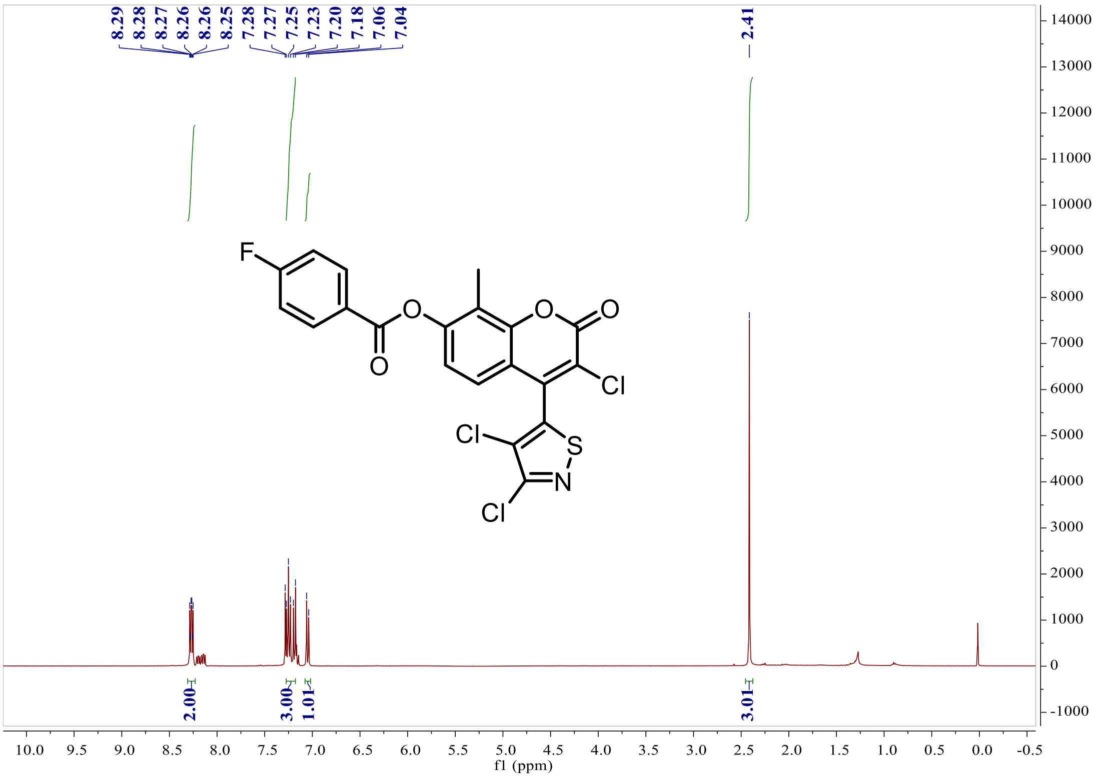

Figure S20. **The ^{1}H NMR (400MHz, Chloroform-*d*) of compound 2ag.**

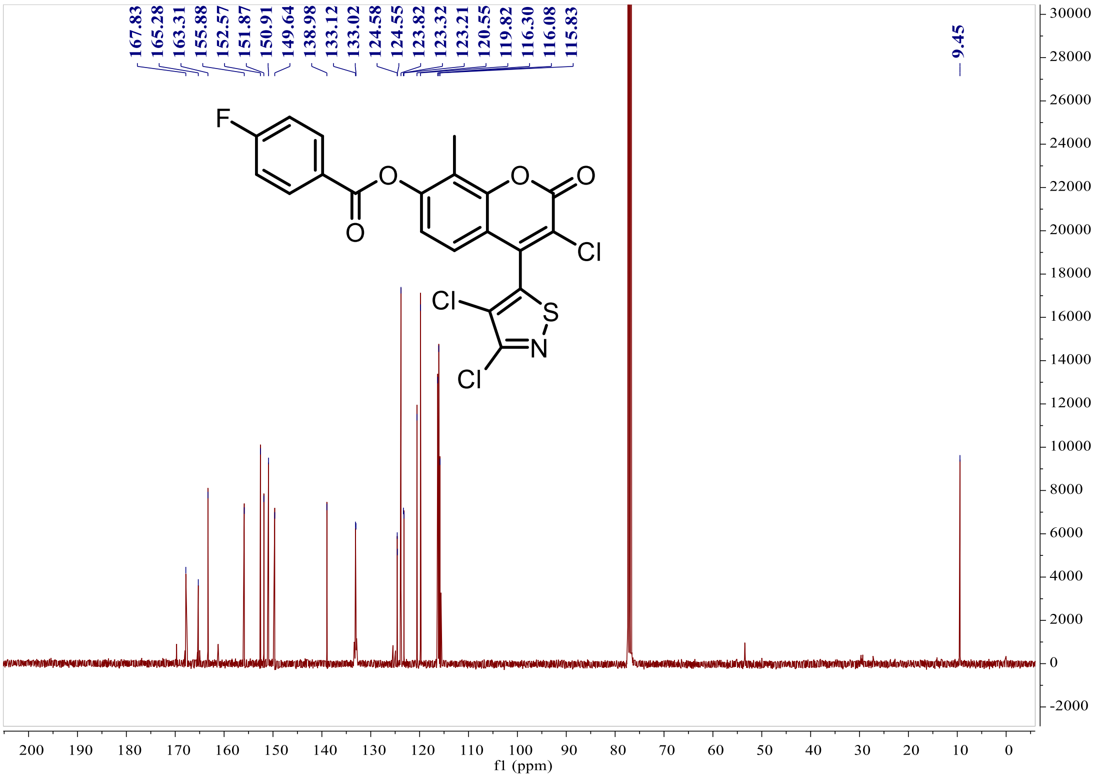

Figure S21. **The ^{13}C NMR (101MHz, Chloroform-*d*) of compound 2ag.**

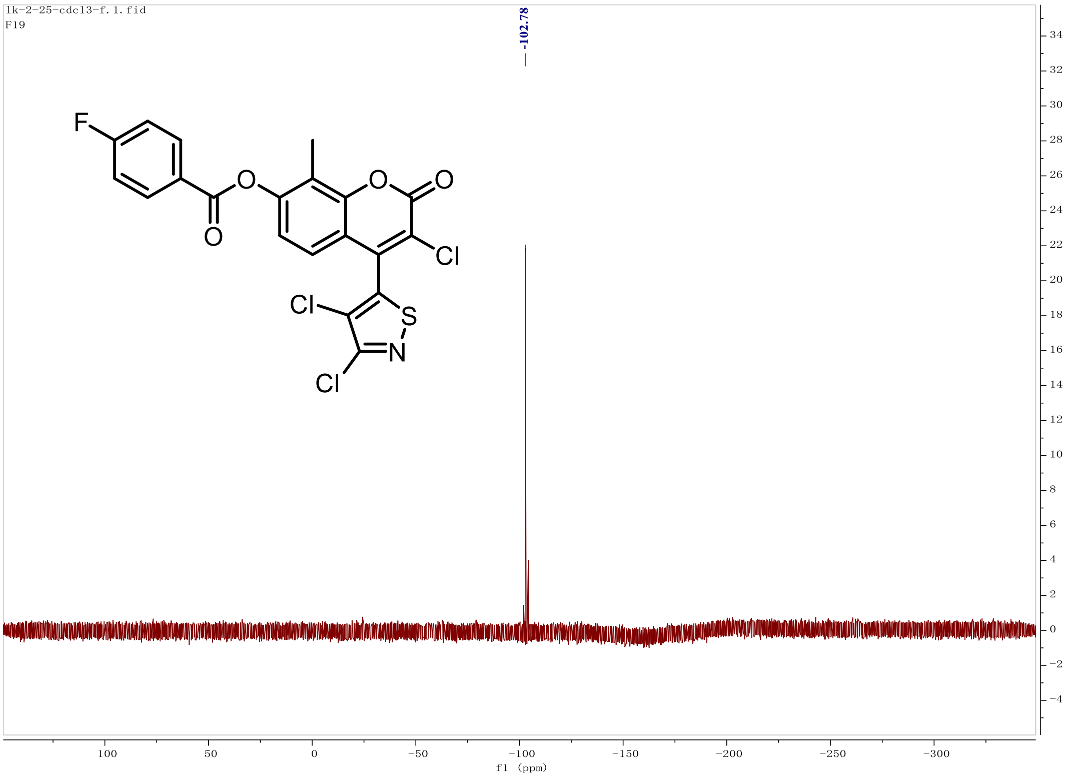

Figure S22. **The ^{19}F NMR (376MHz, Chloroform-*d*) of compound 2ag.**

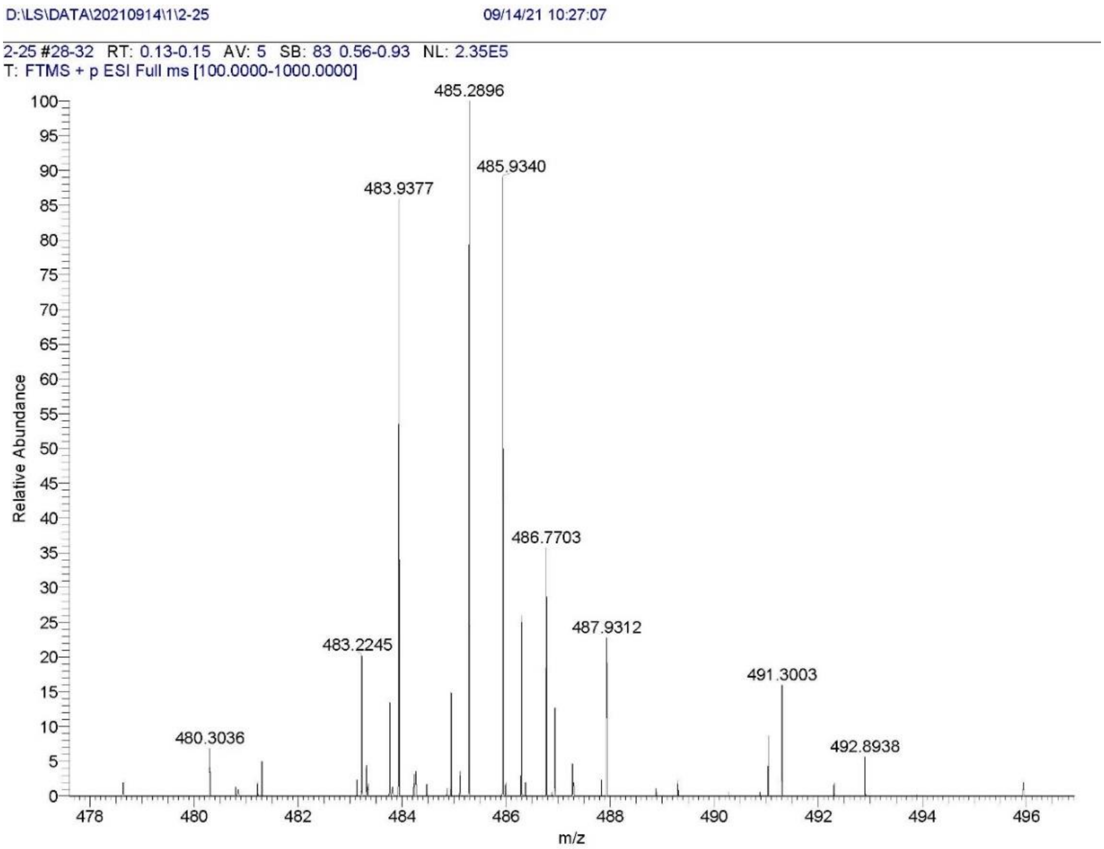

Figure S23. **The HRMS of compound 2ag.**

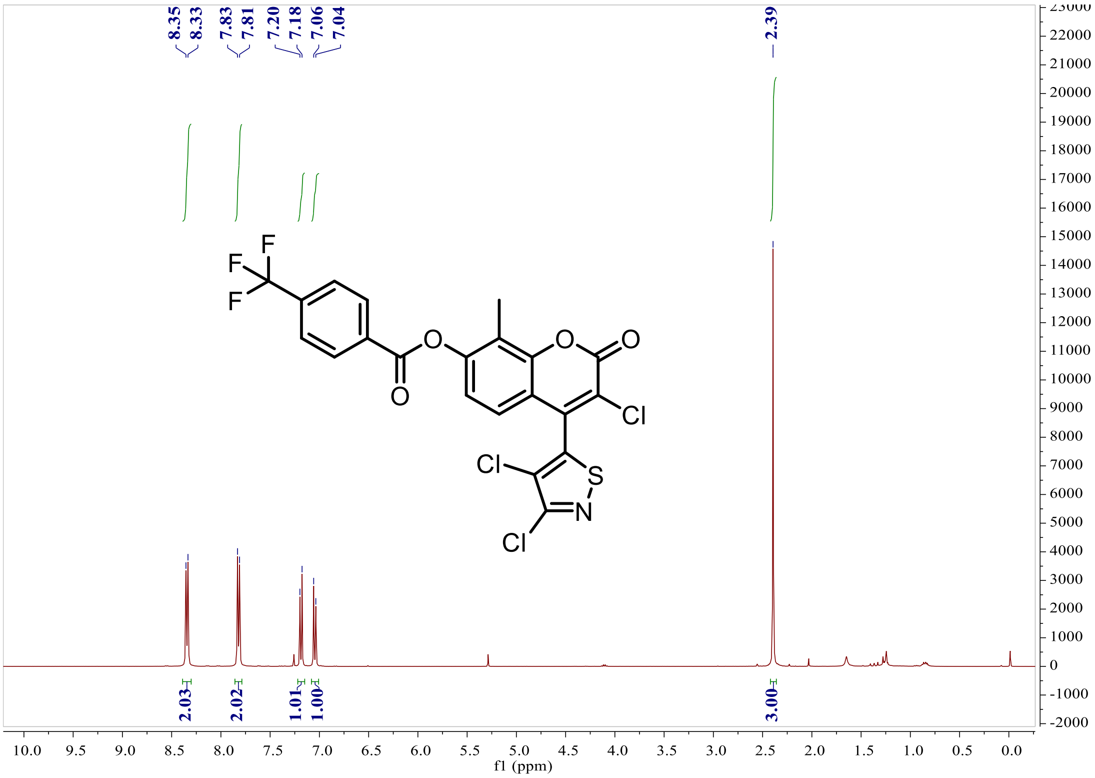

Figure S24. **The ^{1}H NMR (400MHz, Chloroform-*d*) of compound 2ah.**

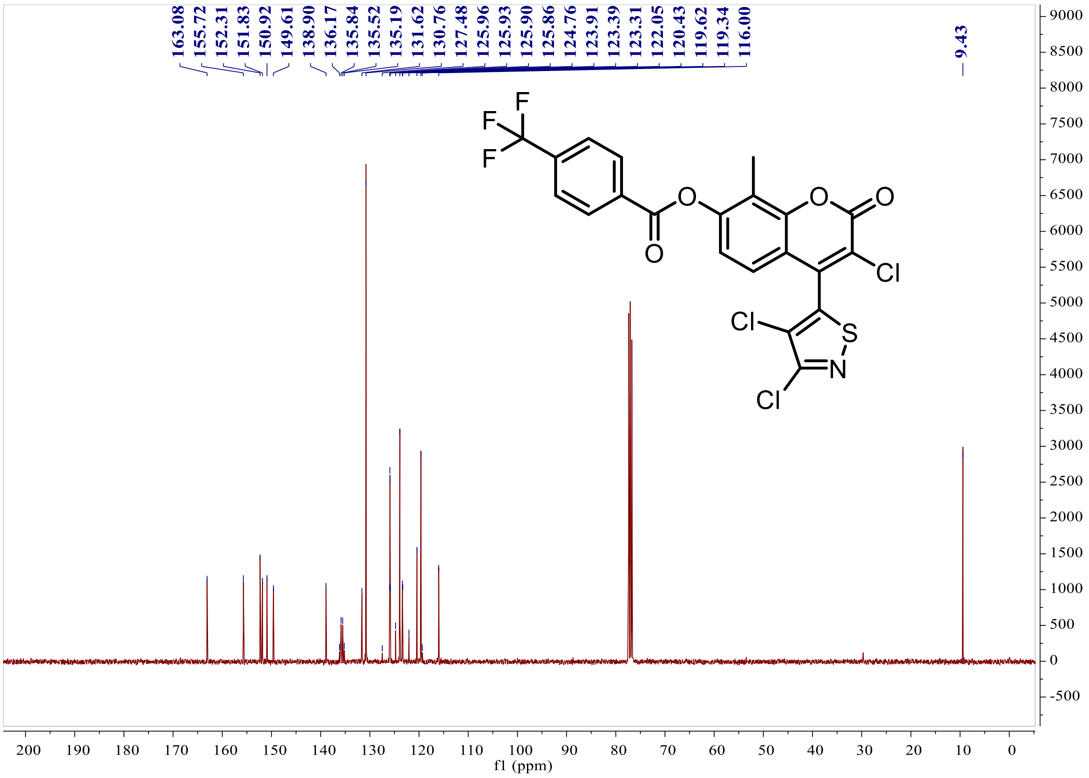

Figure S25. **The ^{13}C NMR (101MHz, Chloroform-*d*) of compound 2ah.**

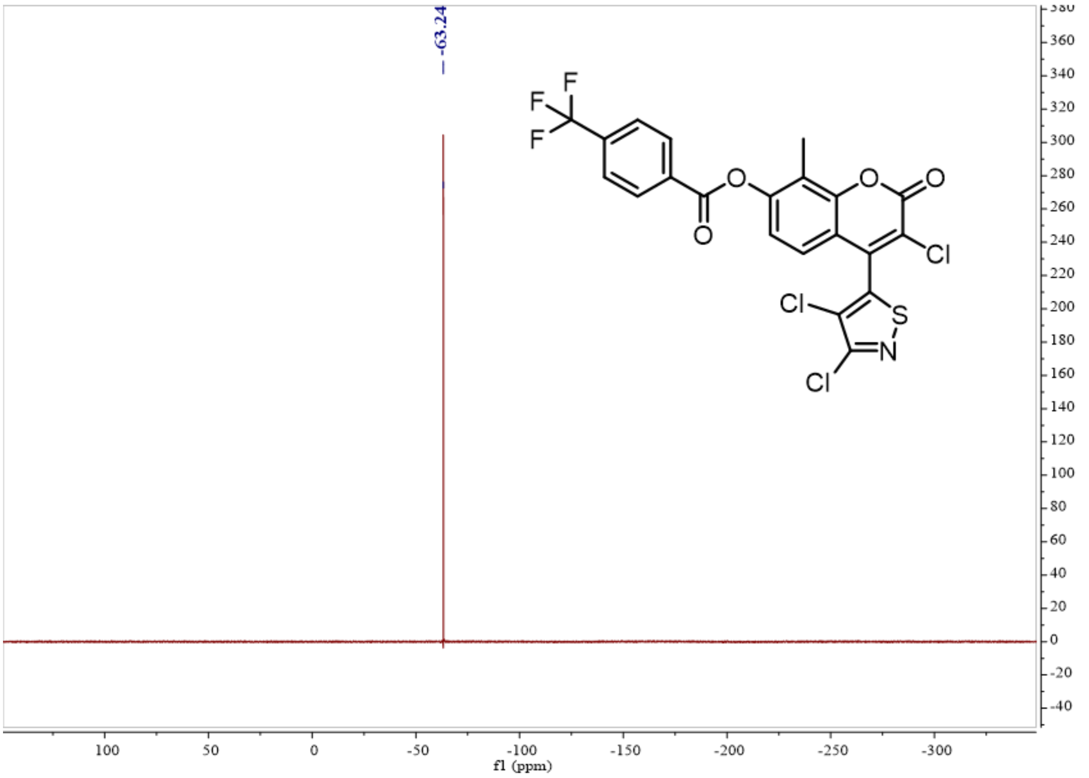

Figure S26. **The ^{19}F NMR (376MHz, Chloroform-*d*) of compound 2ah.**

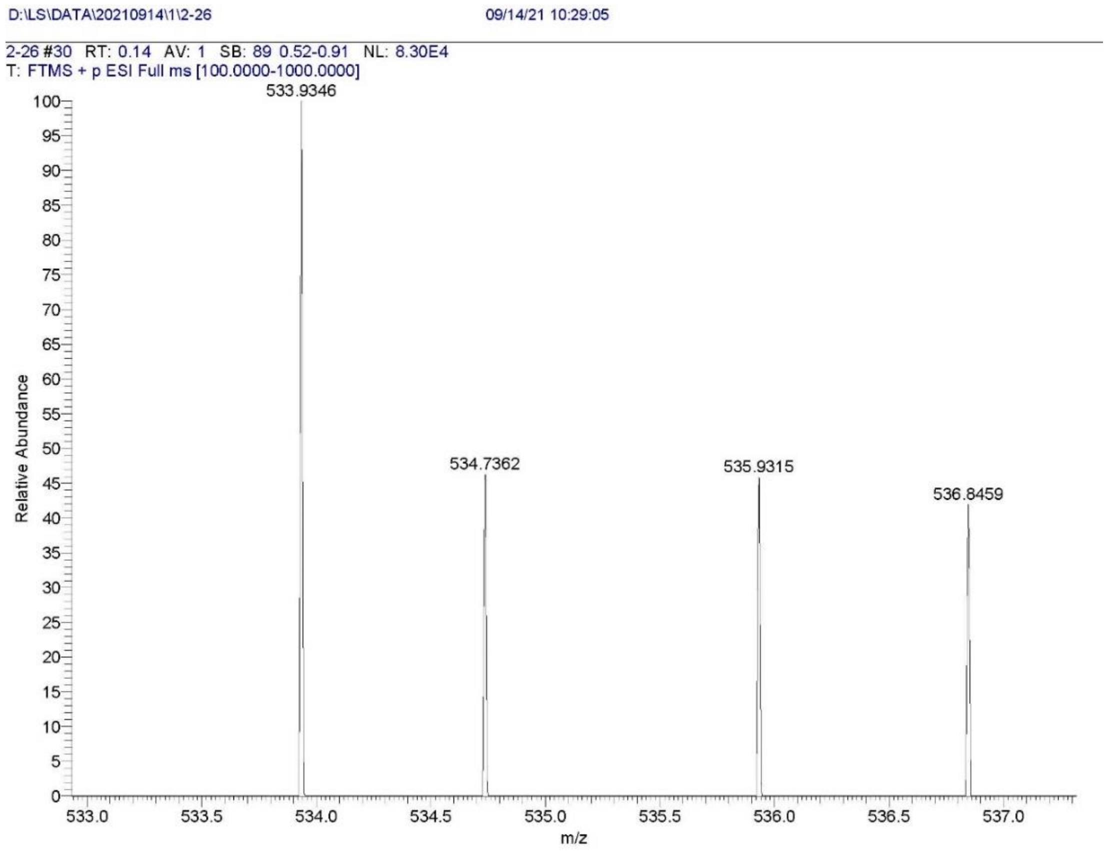

Figure S27. **The HRMS of compound 2ah.**

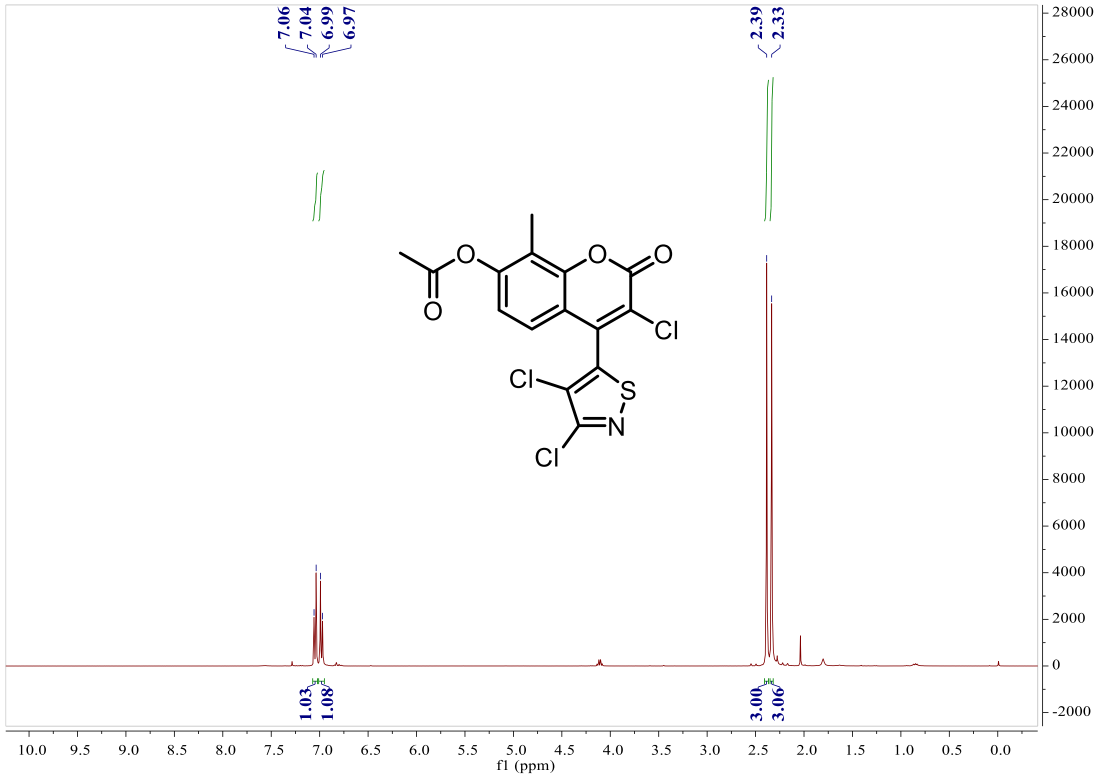

Figure S28. **The ^{1}H NMR (400MHz, Chloroform-*d*) of compound 2ai.**

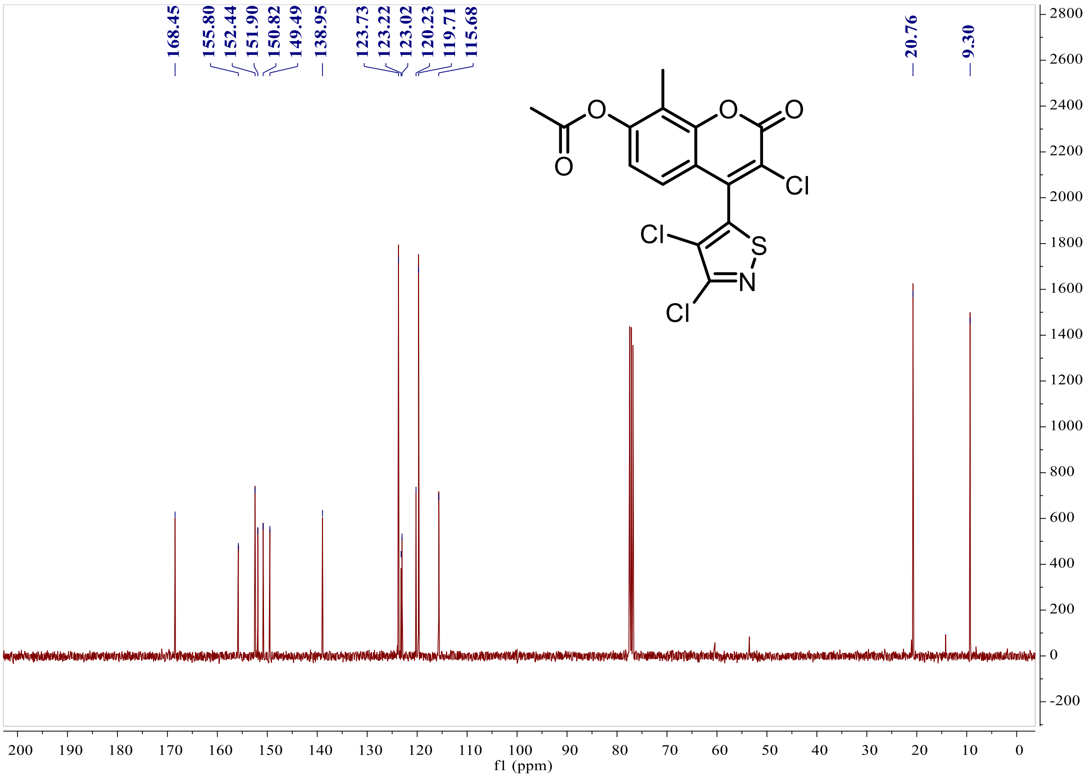

Figure S29. **The ^{13}C NMR (101MHz, Chloroform-*d*) of compound 2ai.**

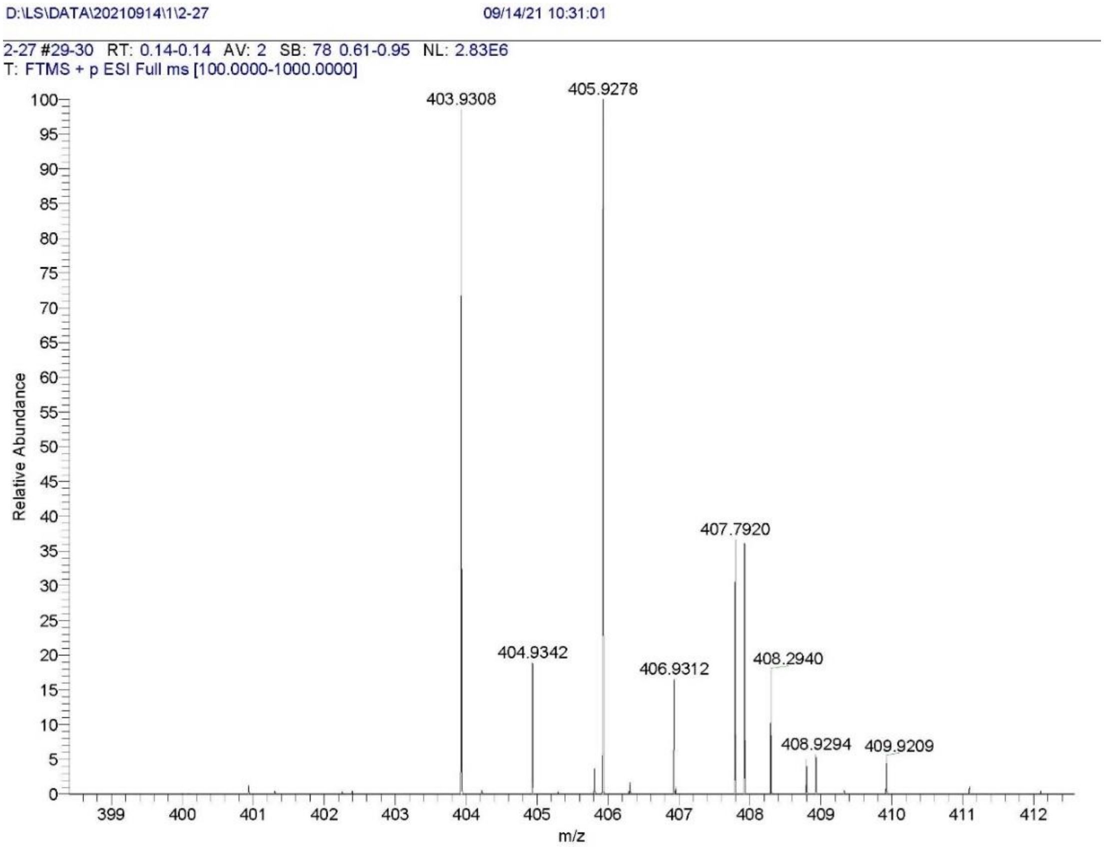

Figure S30. **The HRMS of compound 2ai.**

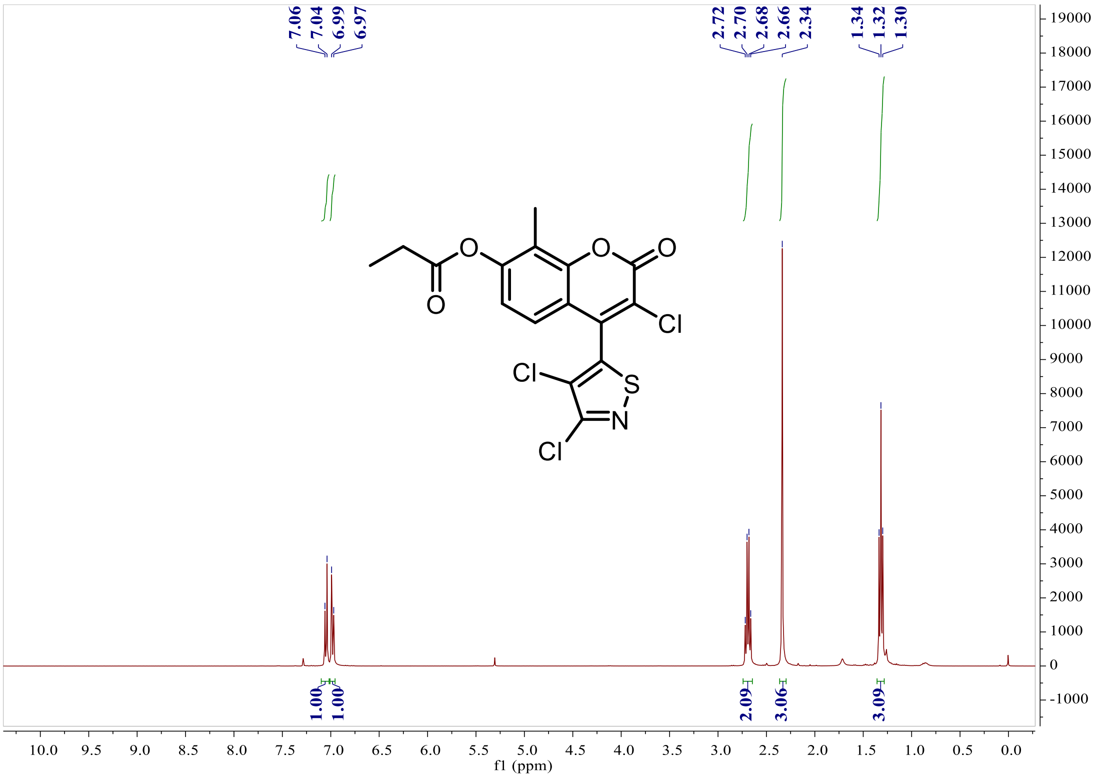

Figure S31. **The** [1]**H NMR (400MHz, Chloroform-*d*) of compound 2aj.**

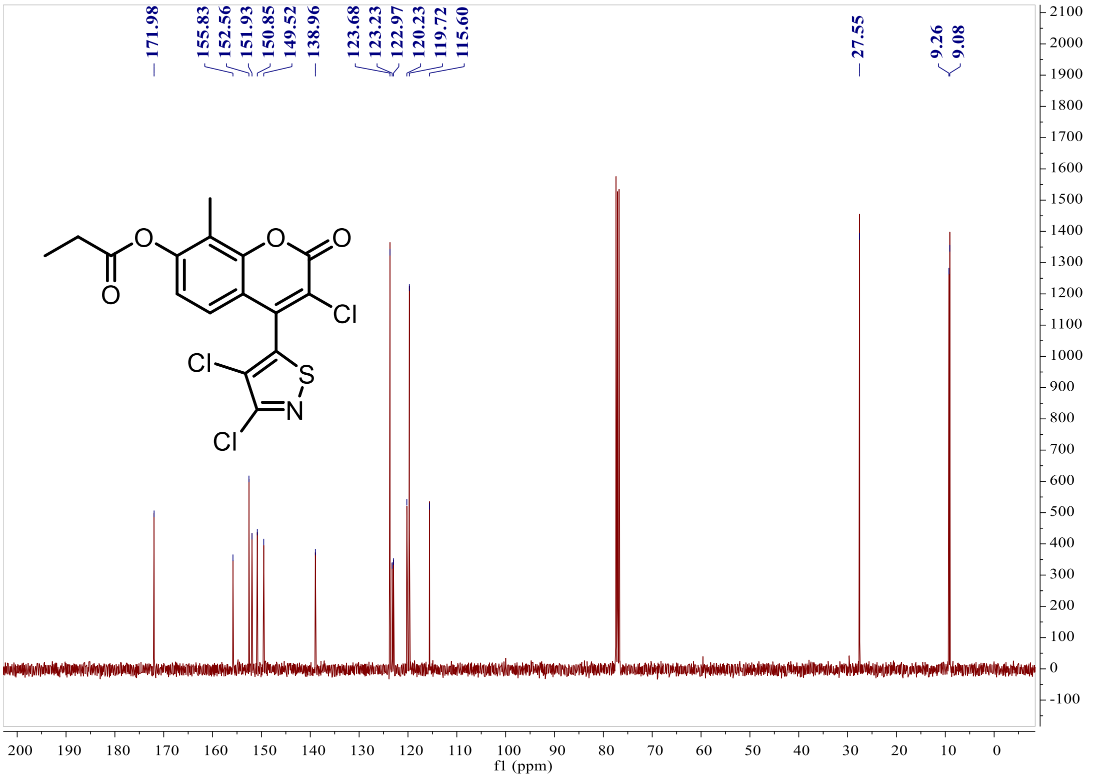

Figure S32. **The** [13]**C NMR (101MHz, Chloroform-*d*) of compound 2aj.**

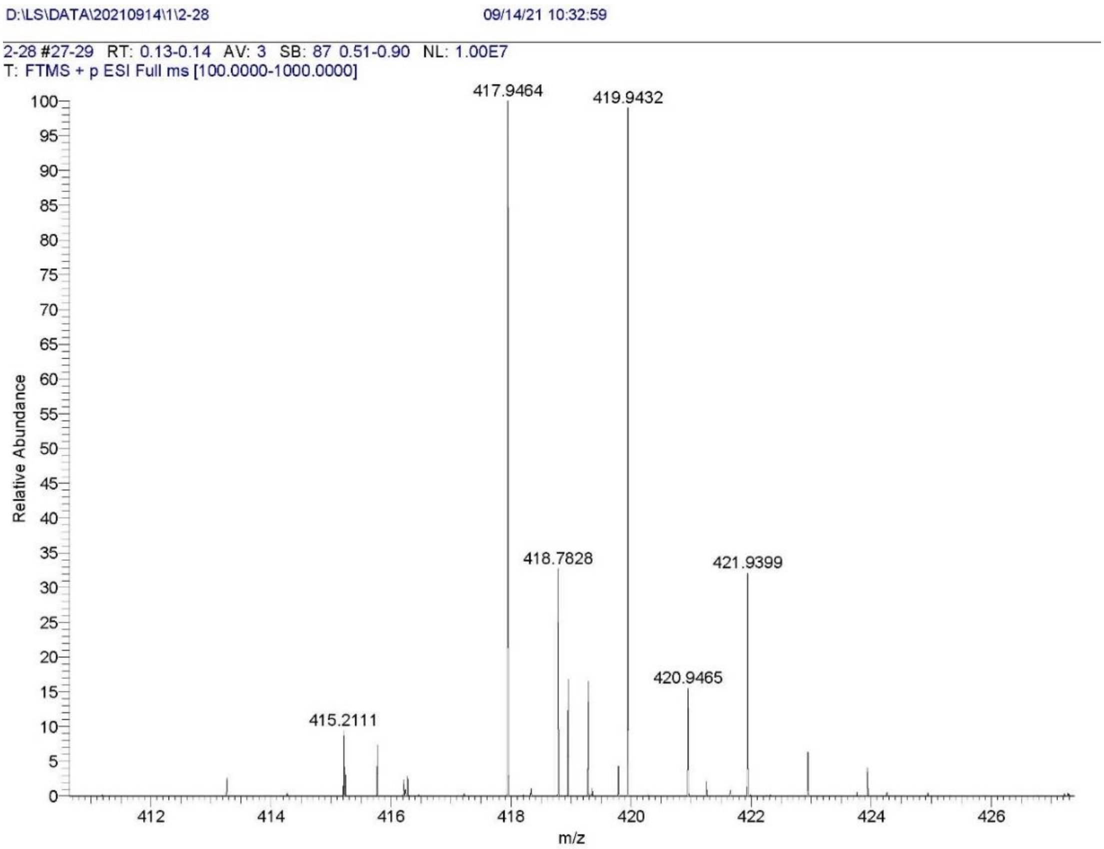

Figure S33. **The HRMS of compound 2aj.**

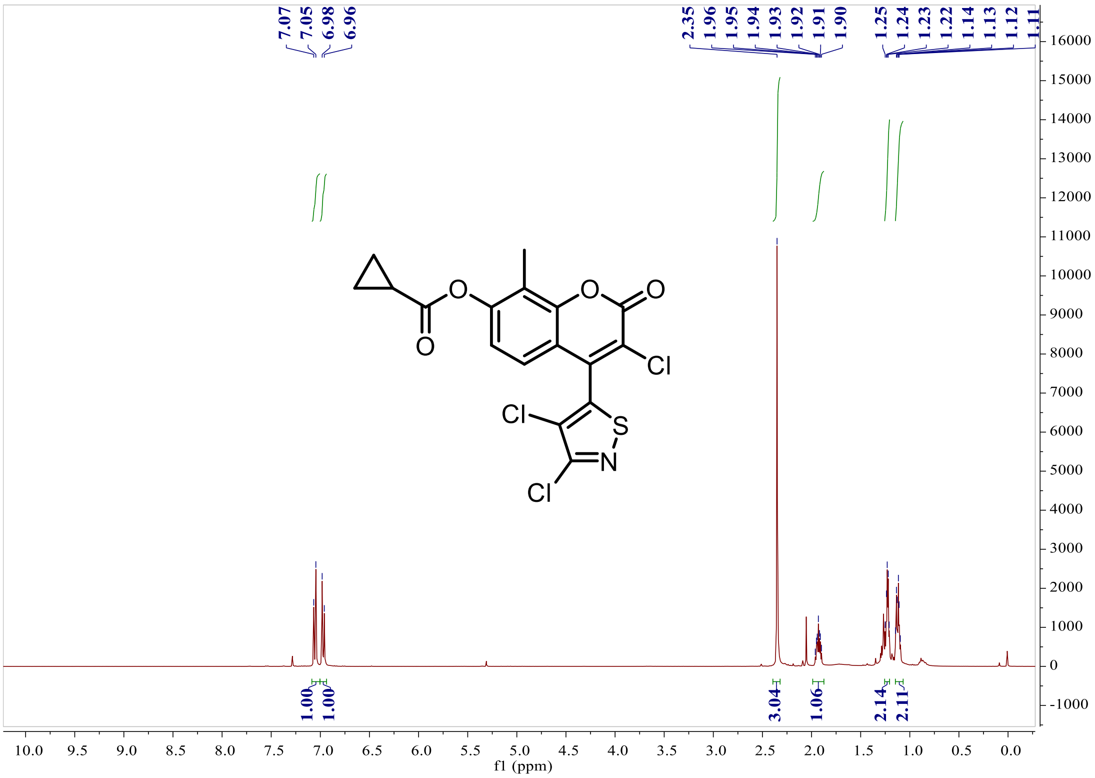

Figure S34. **The ^{1}H NMR (400MHz, Chloroform-*d*) of compound 2ak.**

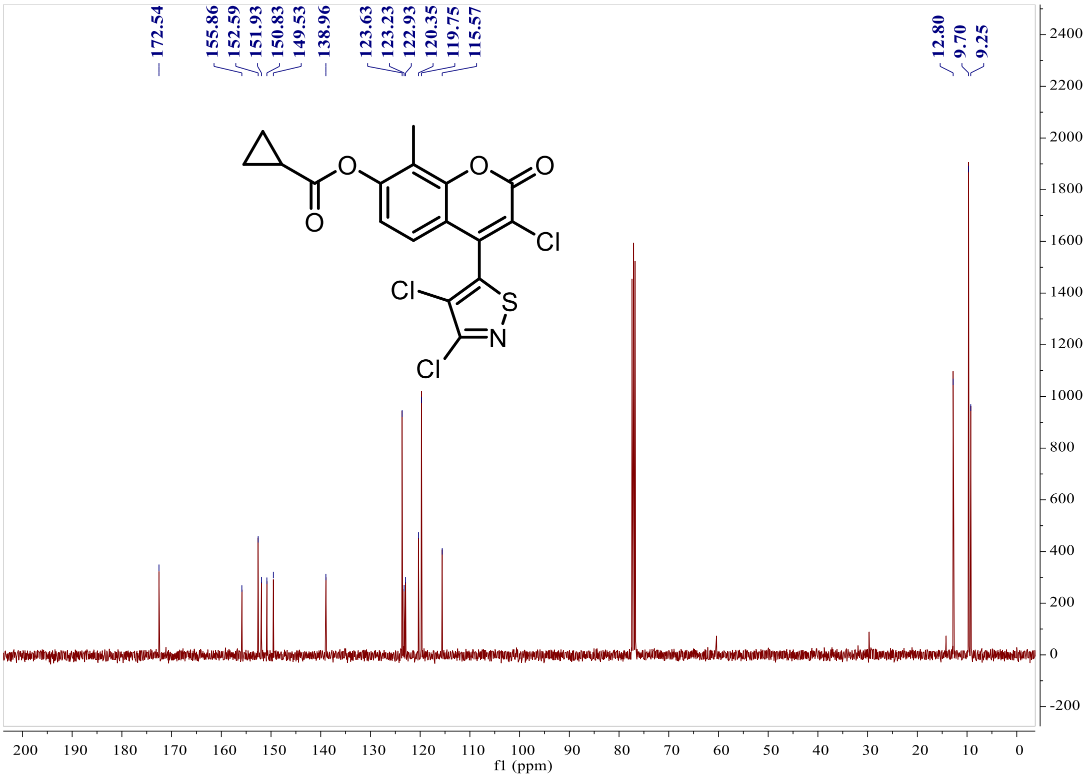

Figure S35. **The ^{13}C NMR (101MHz, Chloroform-*d*) of compound 2ak.**

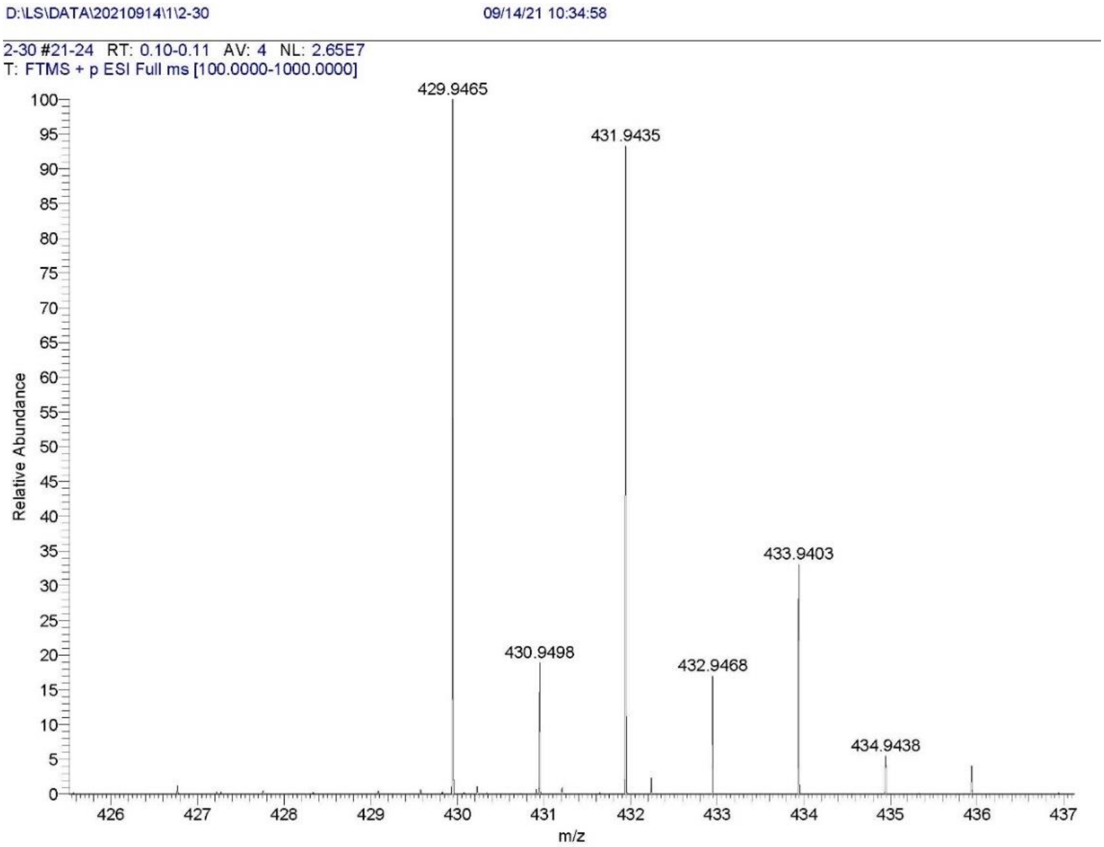

Figure S36. **The HRMS of compound 2ak.**

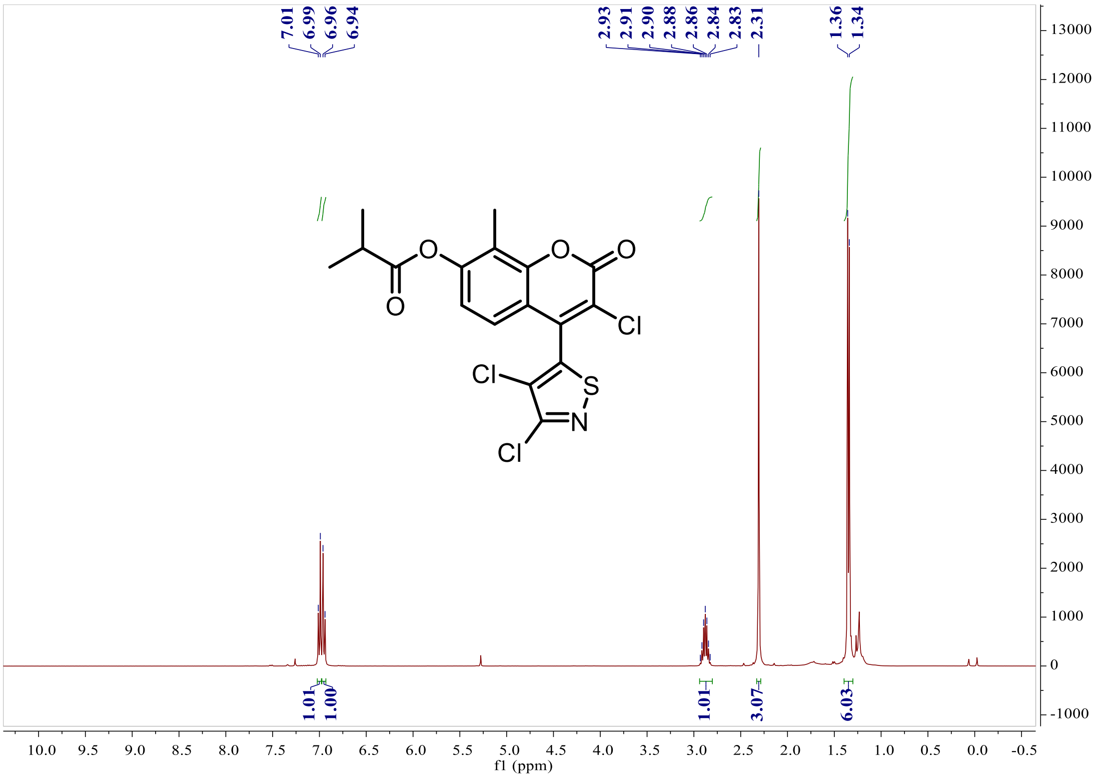

Figure S37. **The ^{1}H NMR (400MHz, Chloroform-*d*) of compound 2al.**

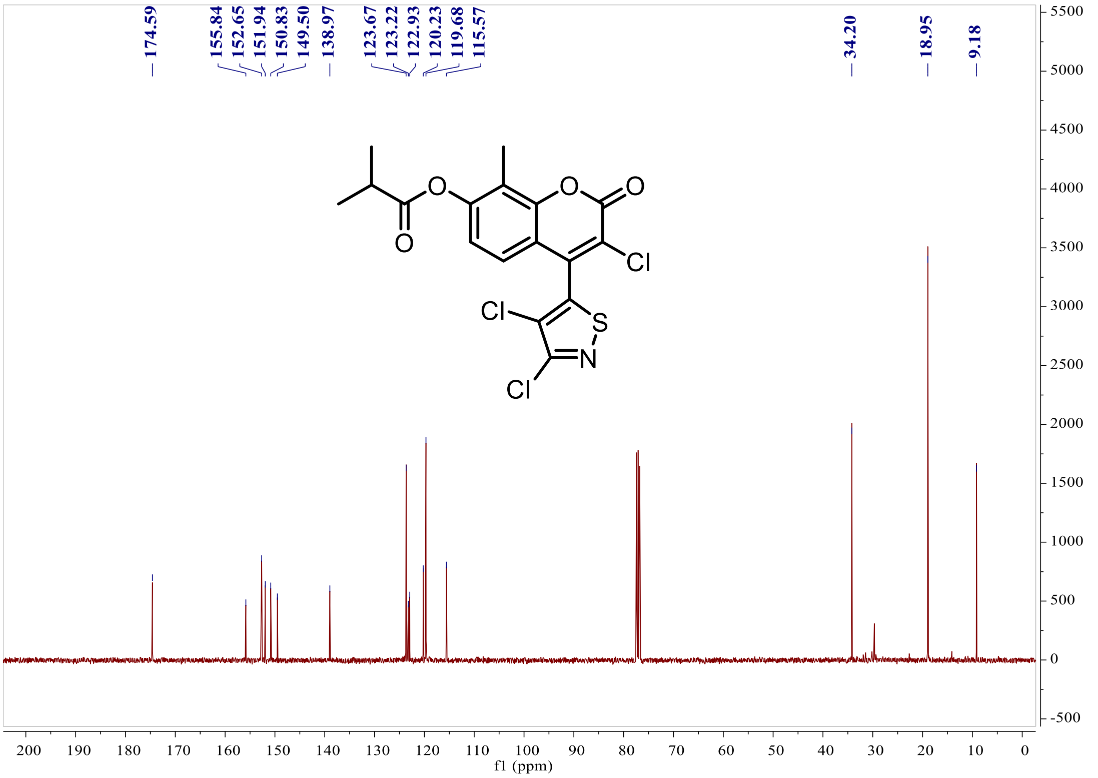

Figure S38. **The ^{13}C NMR (101MHz, Chloroform-*d*) of compound 2al.**

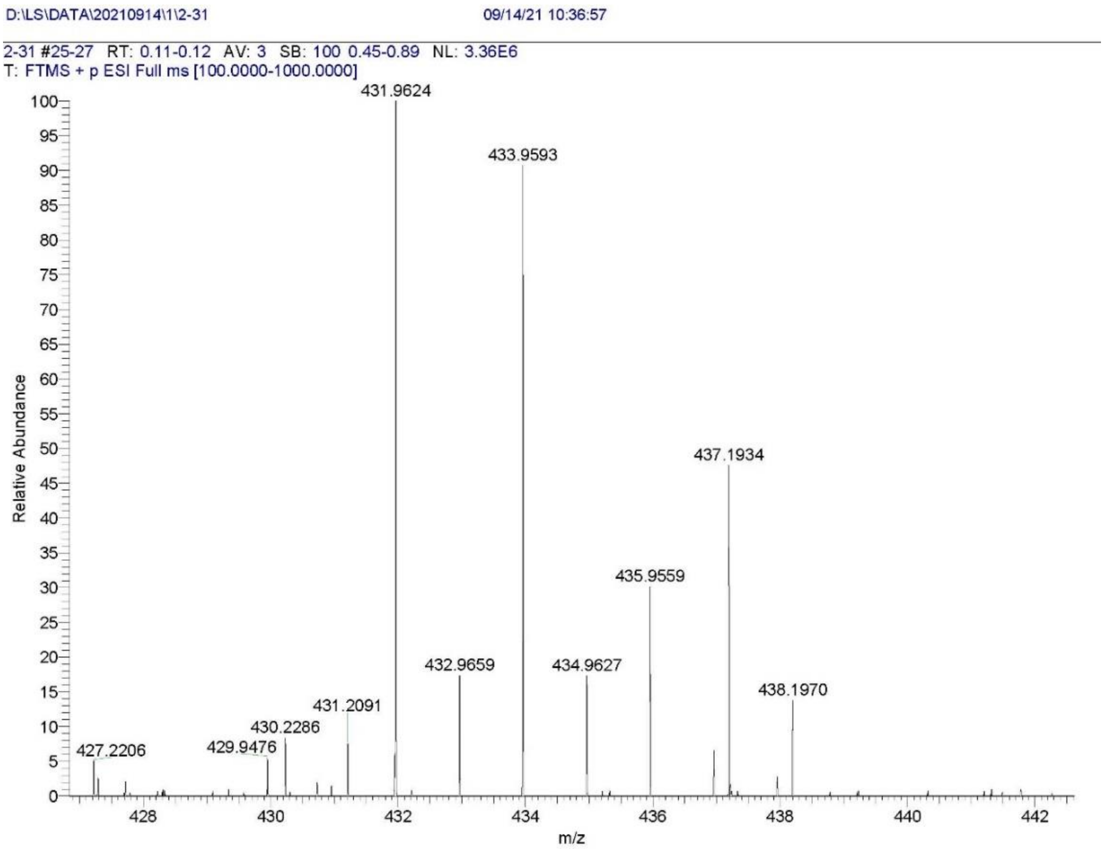

Figure S39. **The HRMS of compound 2al.**

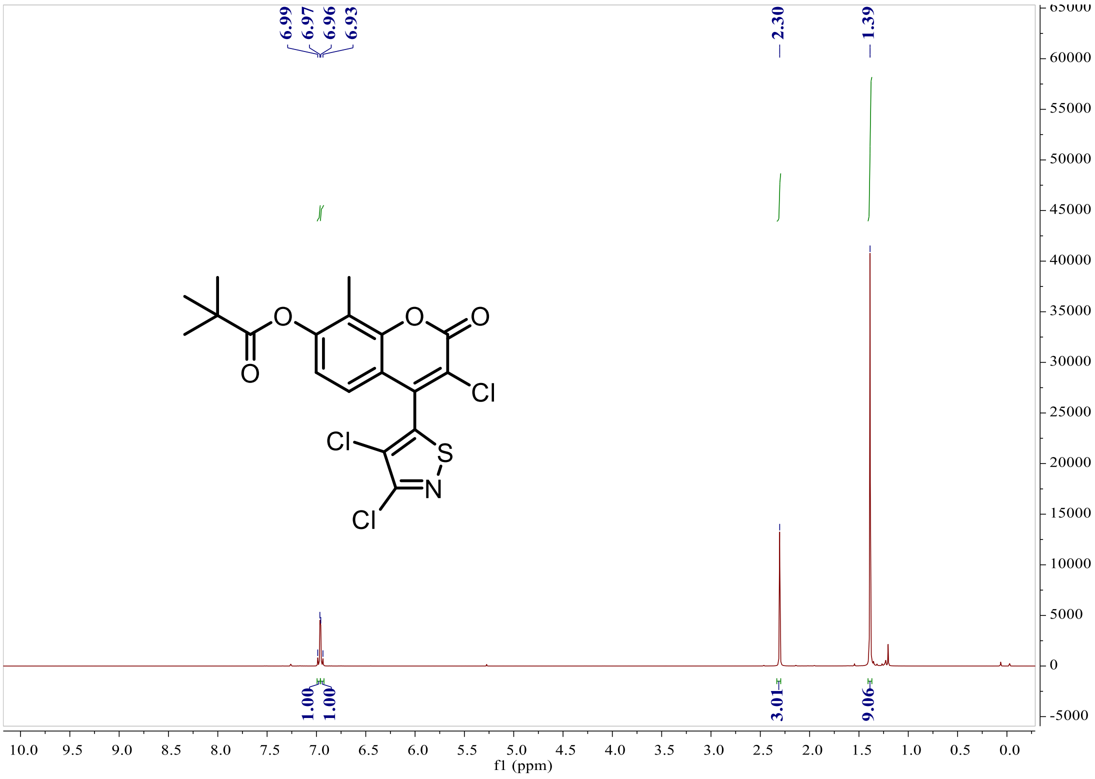

Figure S40. **The ^{1}H NMR (400MHz, Chloroform-*d*) of compound 2am.**

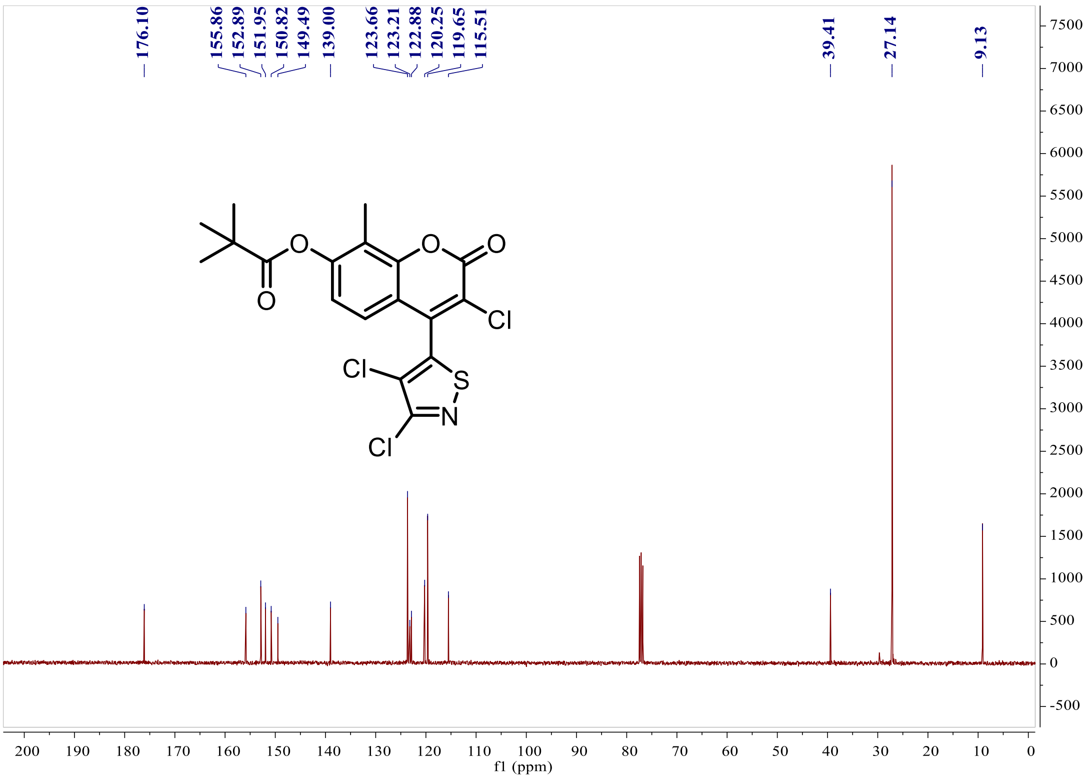

Figure S41. **The ^{13}C NMR (101MHz, Chloroform-*d*) of compound 2am.**

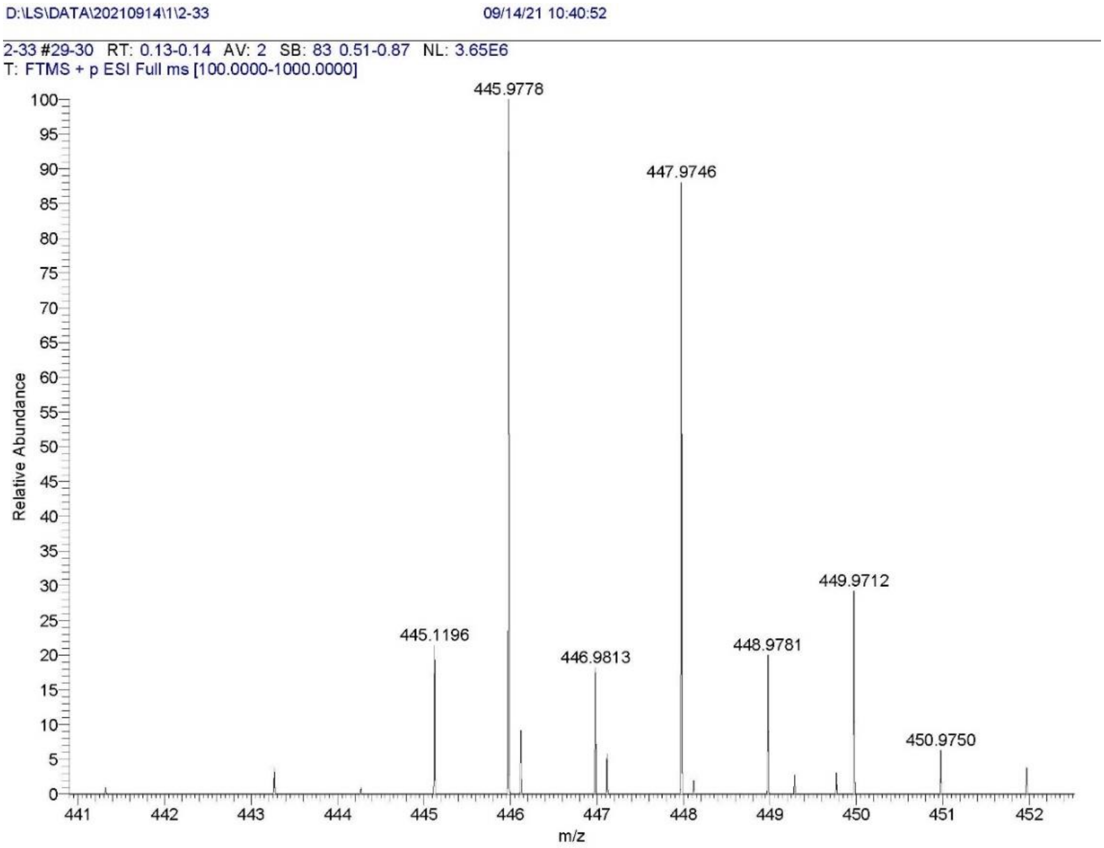

Figure S42. **The HRMS of compound 2am.**

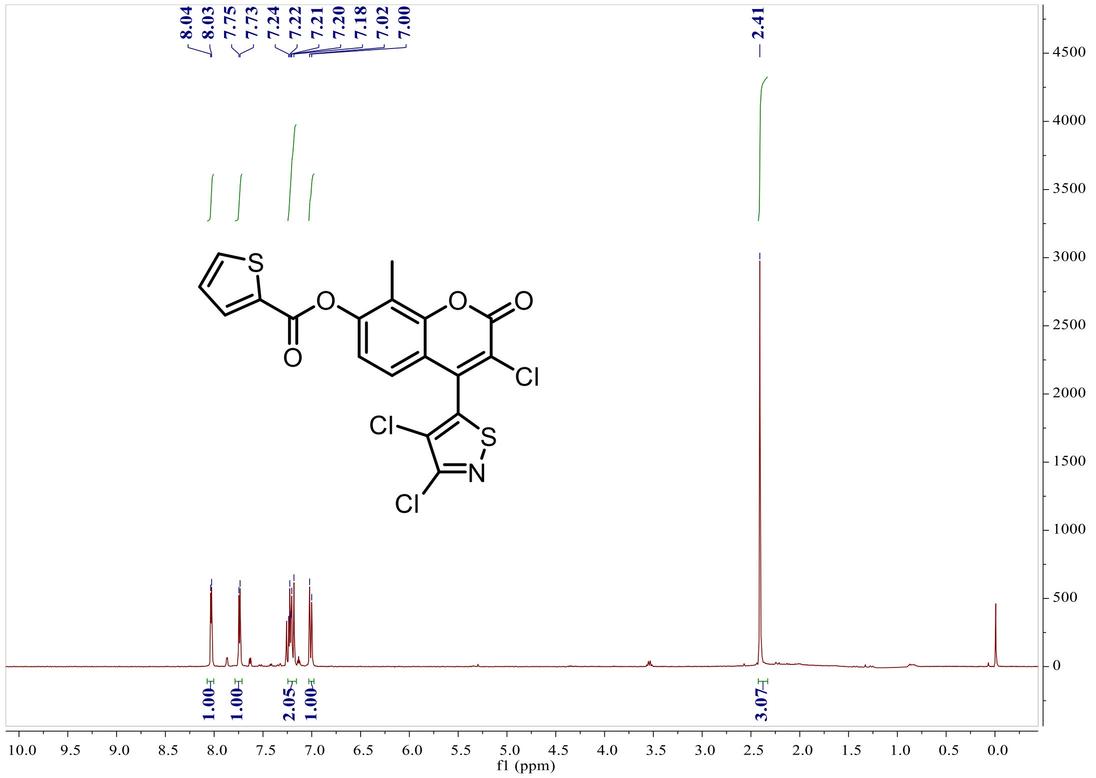

Figure S43. **The ^{1}H NMR (400MHz, Chloroform-*d*) of compound 2an.**

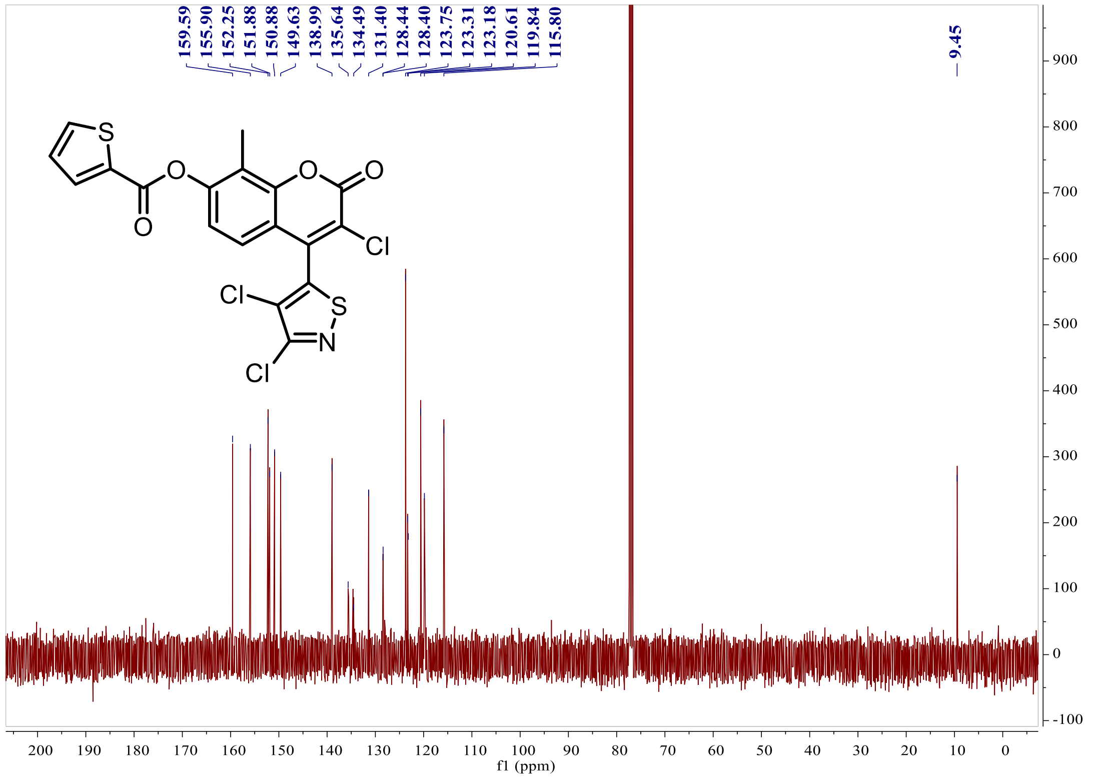

Figure S44. **The ^{13}C NMR (101MHz, Chloroform-*d*) of compound 2an.**

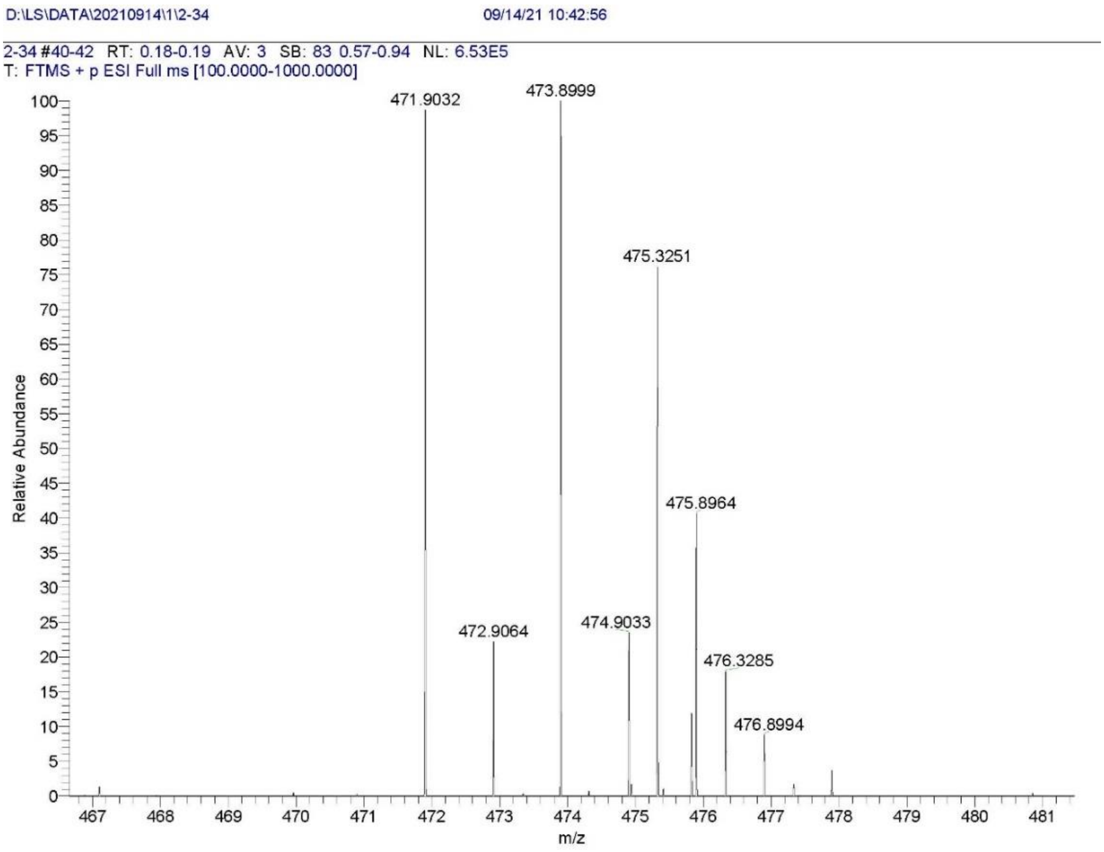

Figure S45. **The HRMS of compound 2an.**

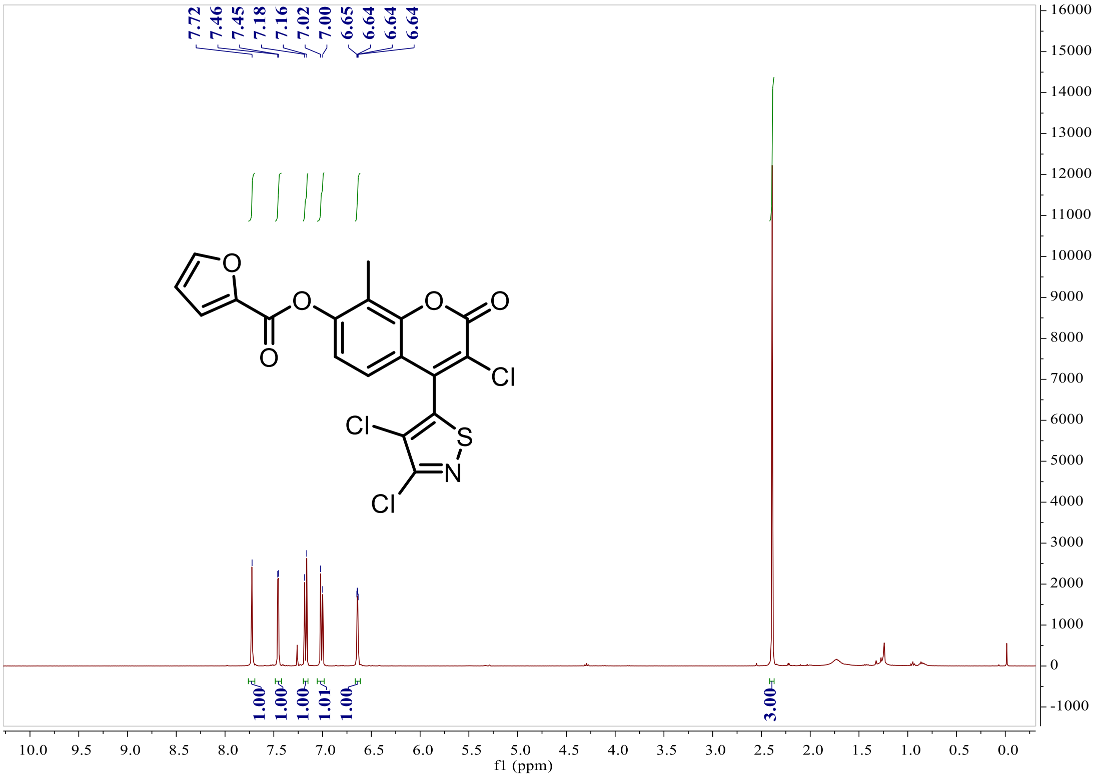

Figure S46. **The ^{1}H NMR (400MHz, Chloroform-*d*) of compound 2ao.**

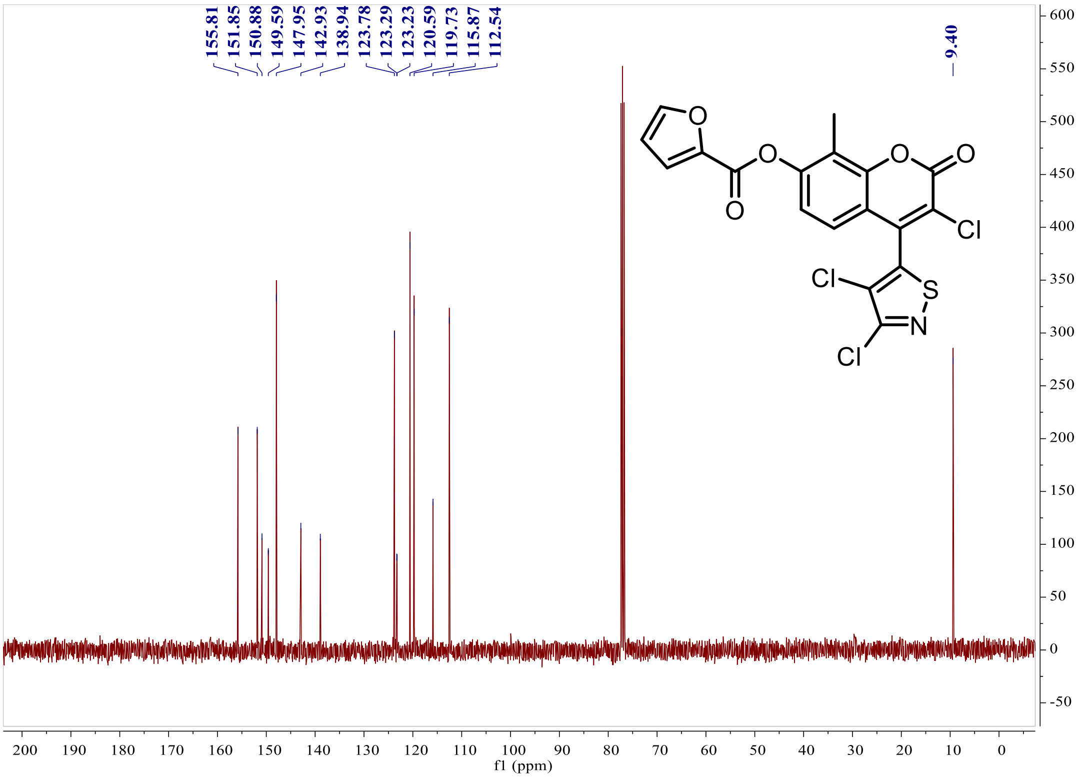

Figure S47. **The ^{13}C NMR (101MHz, Chloroform-*d*) of compound 2ao.**

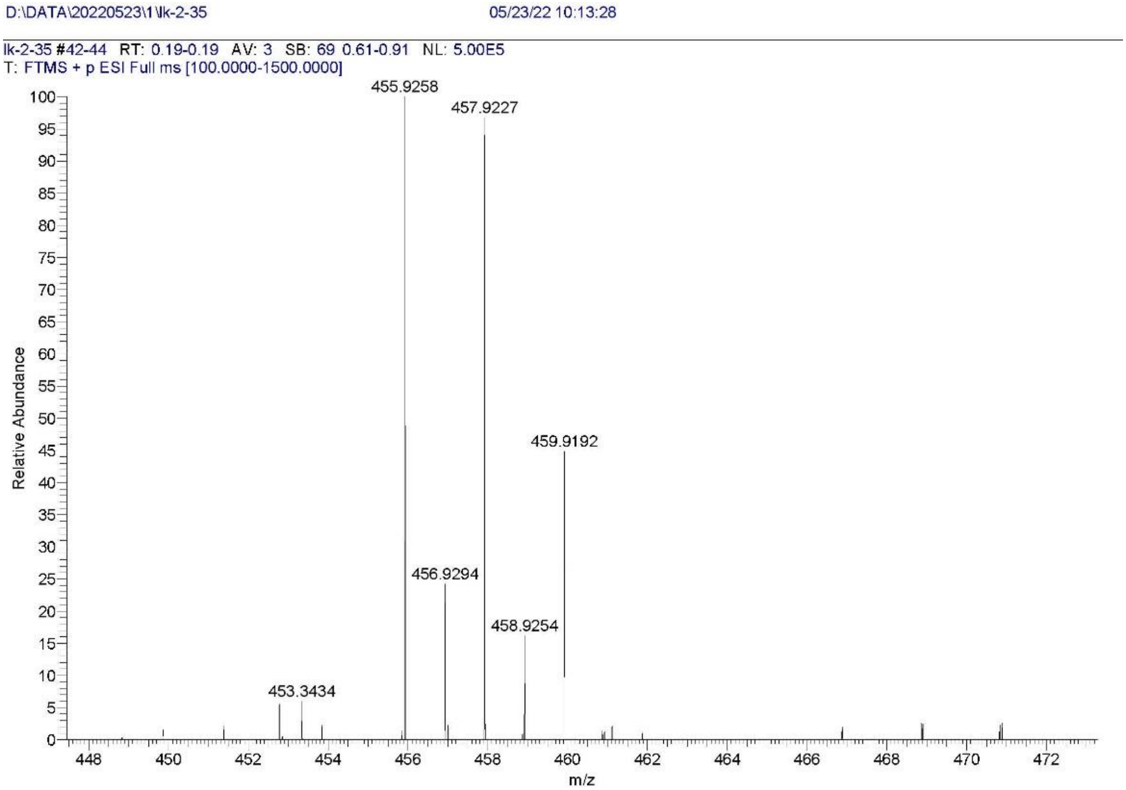

Figure S48. **The HRMS of compound 2ao.**

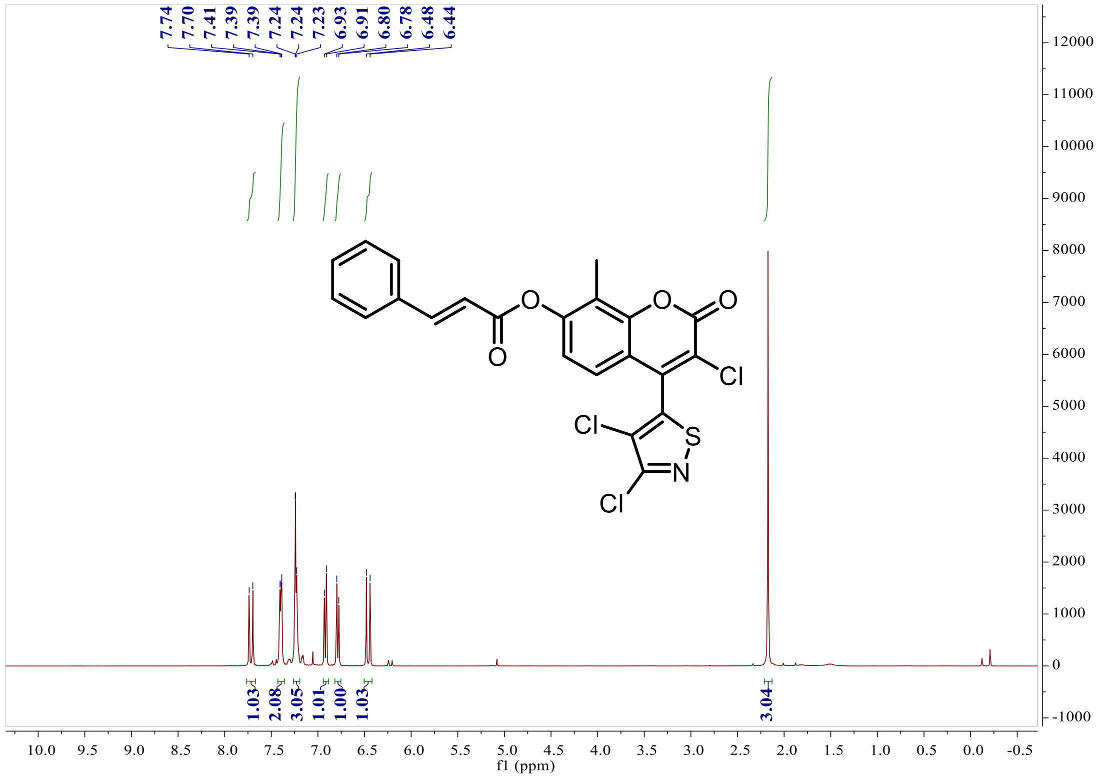

Figure S49. **The ^{1}H NMR (400MHz, Chloroform-*d*) of compound 2ap.**

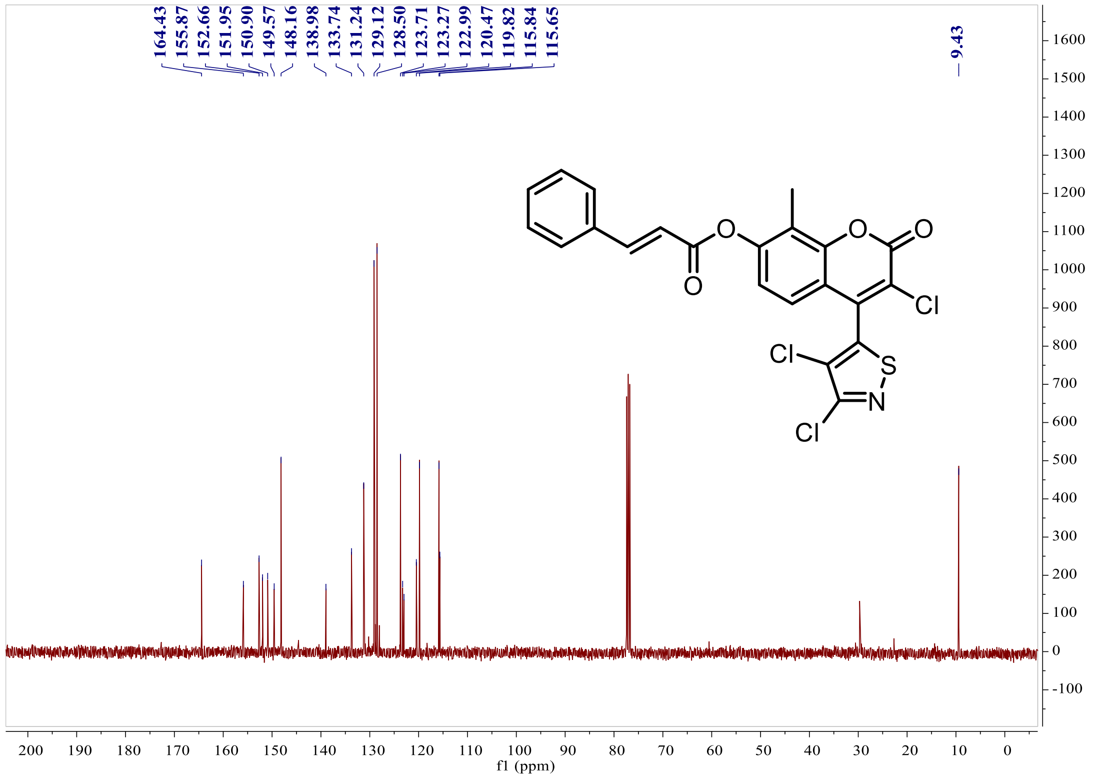

Figure S50. **The ^{13}C NMR (101MHz, Chloroform-*d*) of compound 2ap.**

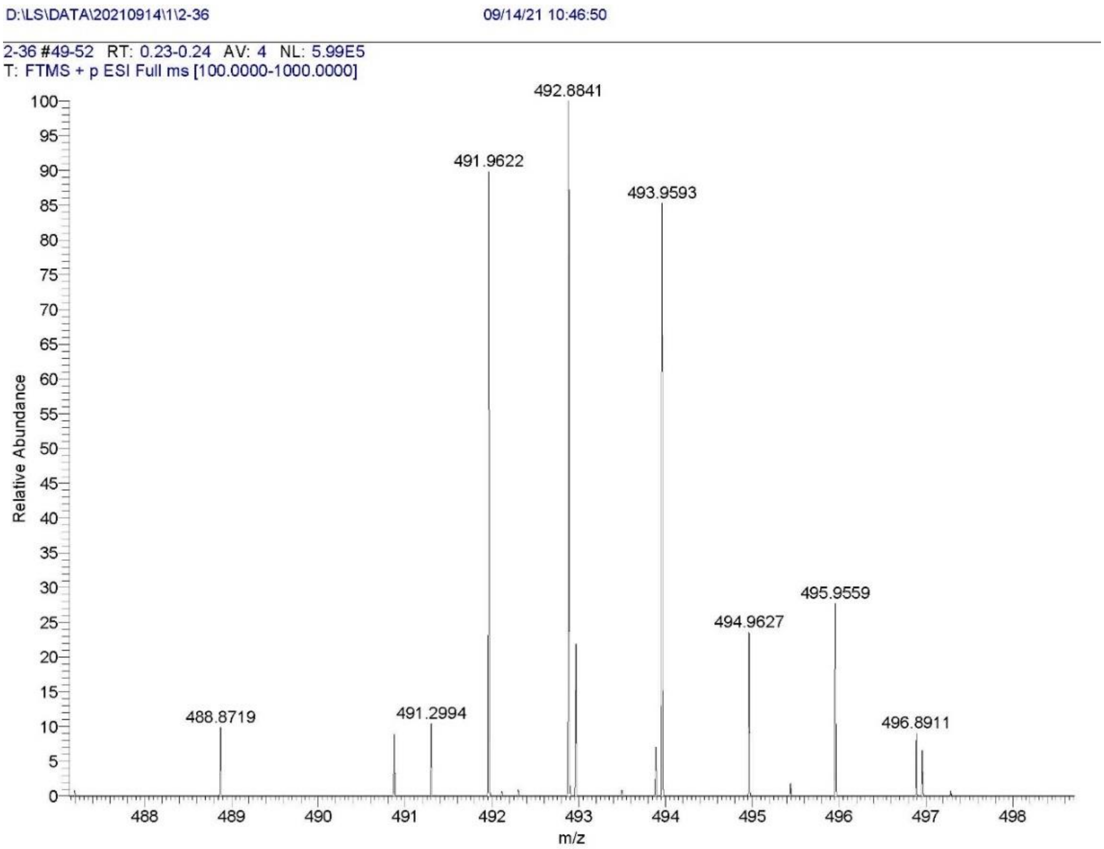

Figure S51. **The HRMS of compound 2ap.**

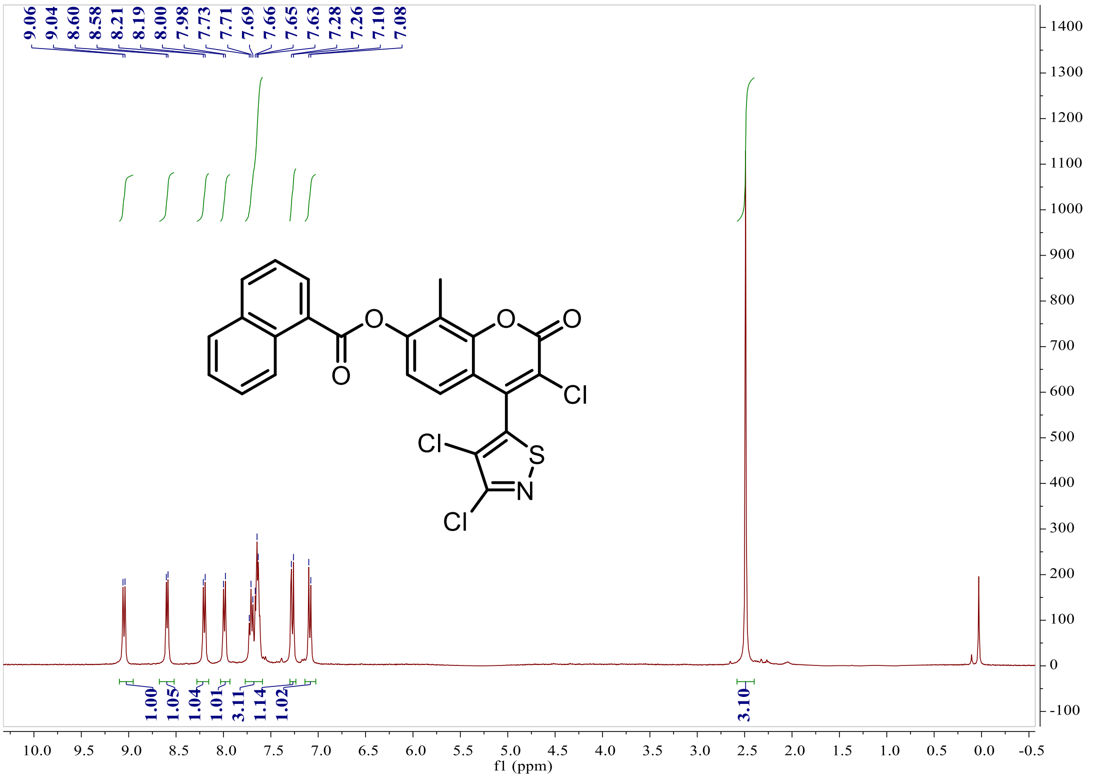

Figure S52. **The ^{1}H NMR (400MHz, Chloroform-*d*) of compound 2aq.**

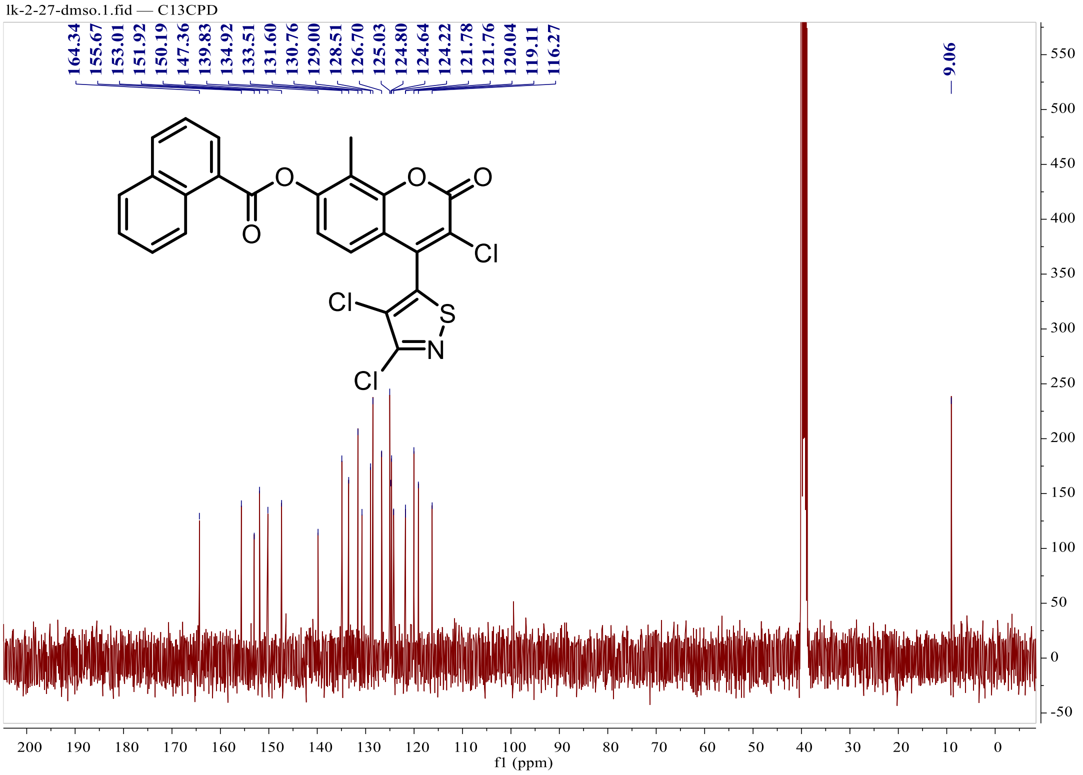

Figure S53. **The ^{13}C NMR (101MHz, DMSO) of compound 2aq.**

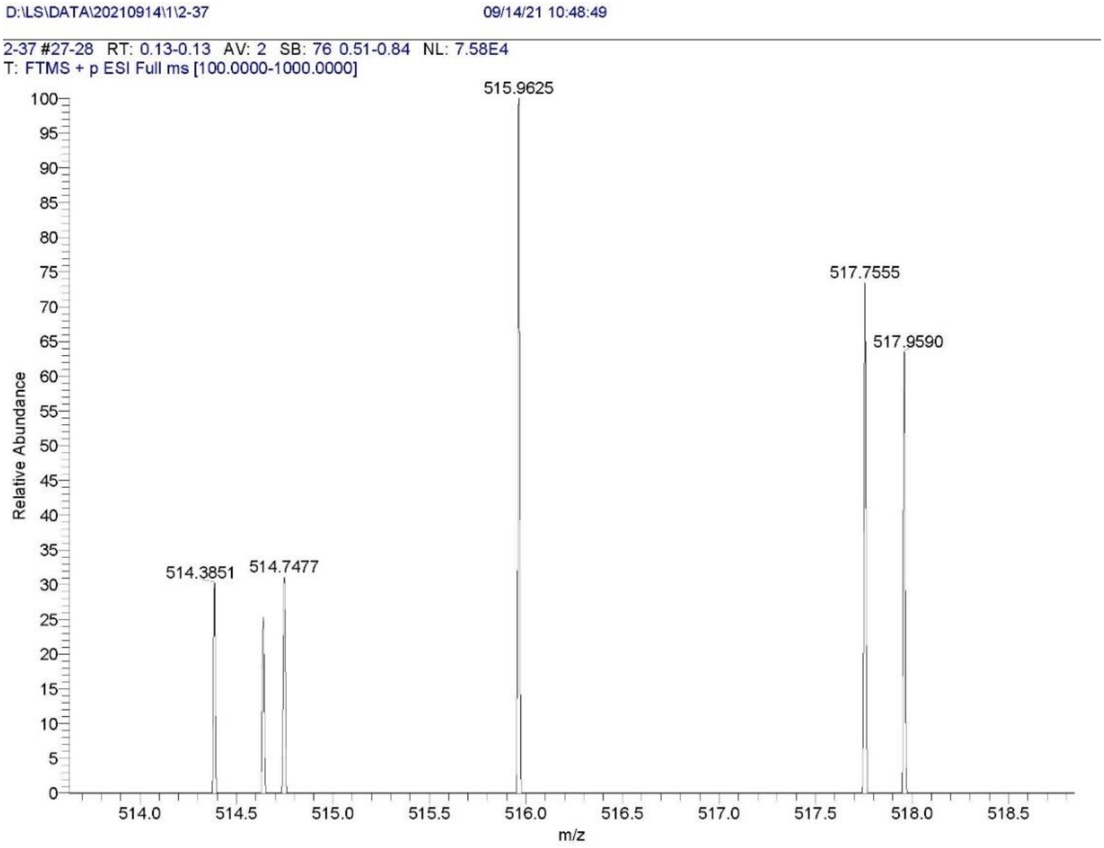

Figure S54. **The HRMS of compound 2aq.**

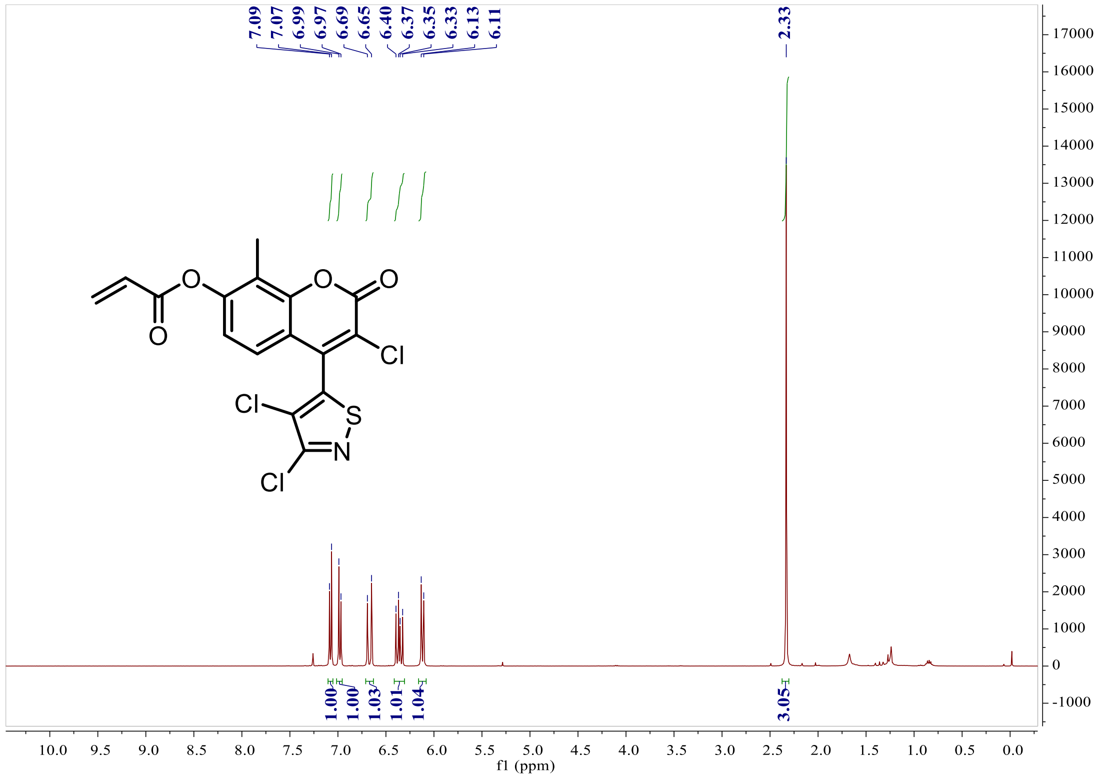

Figure S55. **The ^{1}H NMR (400MHz, Chloroform-*d*) of compound 2ar.**

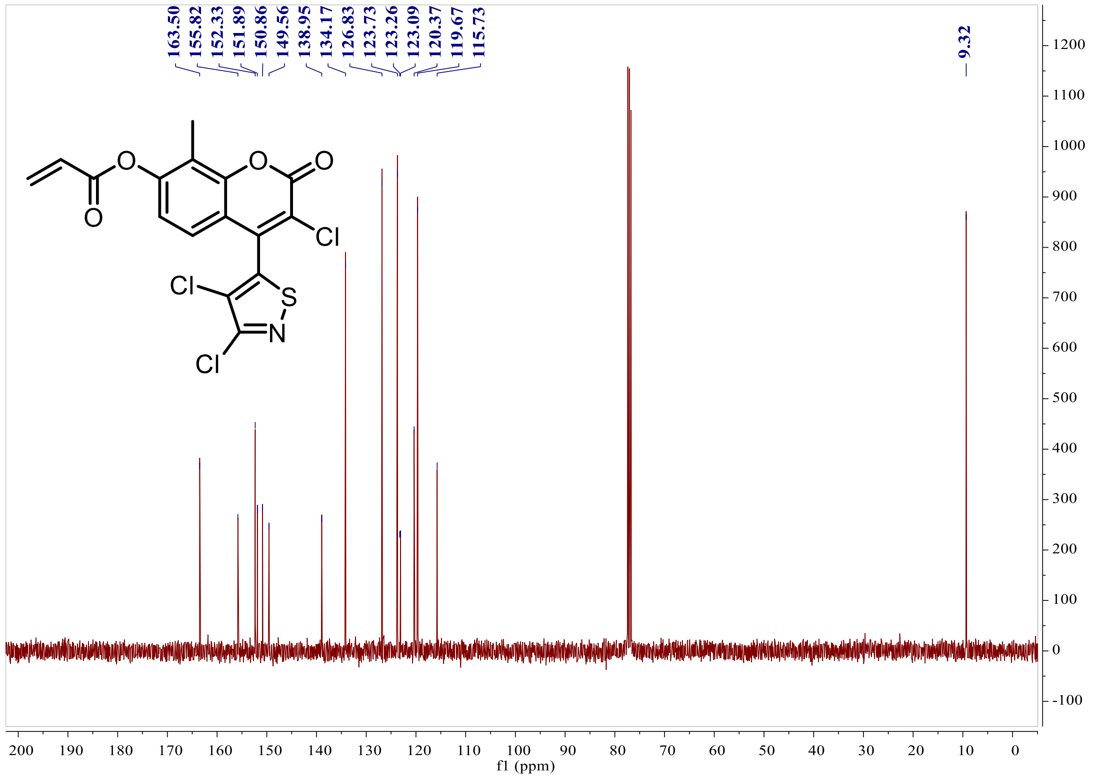

Figure S56. **The ^{13}C NMR (101MHz, Chloroform-*d*) of compound 2ar.**

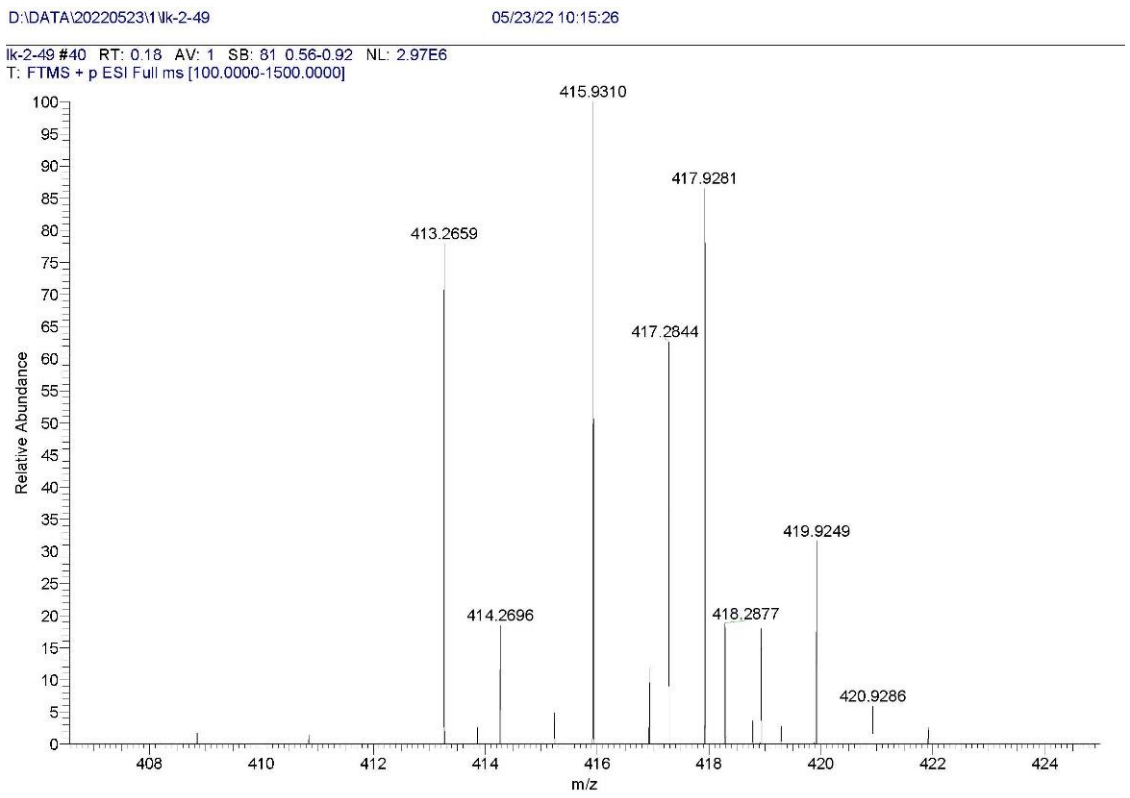

Figure S57. **The HRMS of compound 2ar.**

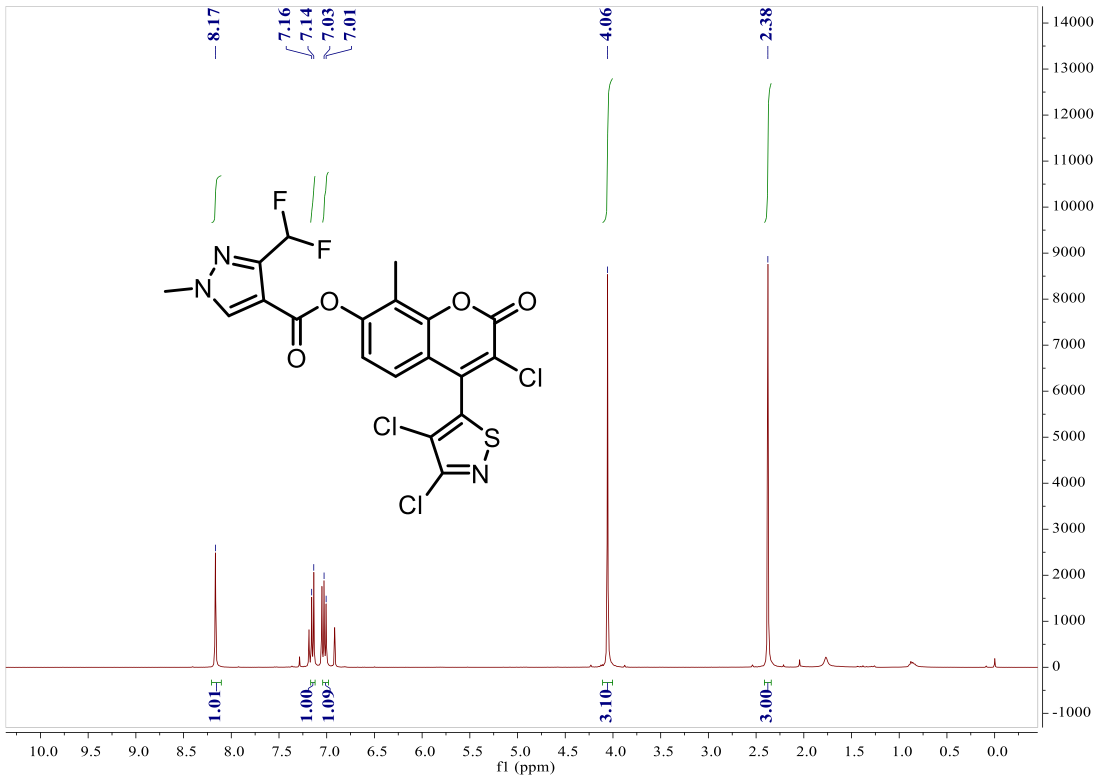

Figure S58. **The ^{1}H NMR (400MHz, Chloroform-*d*) of compound 2as.**

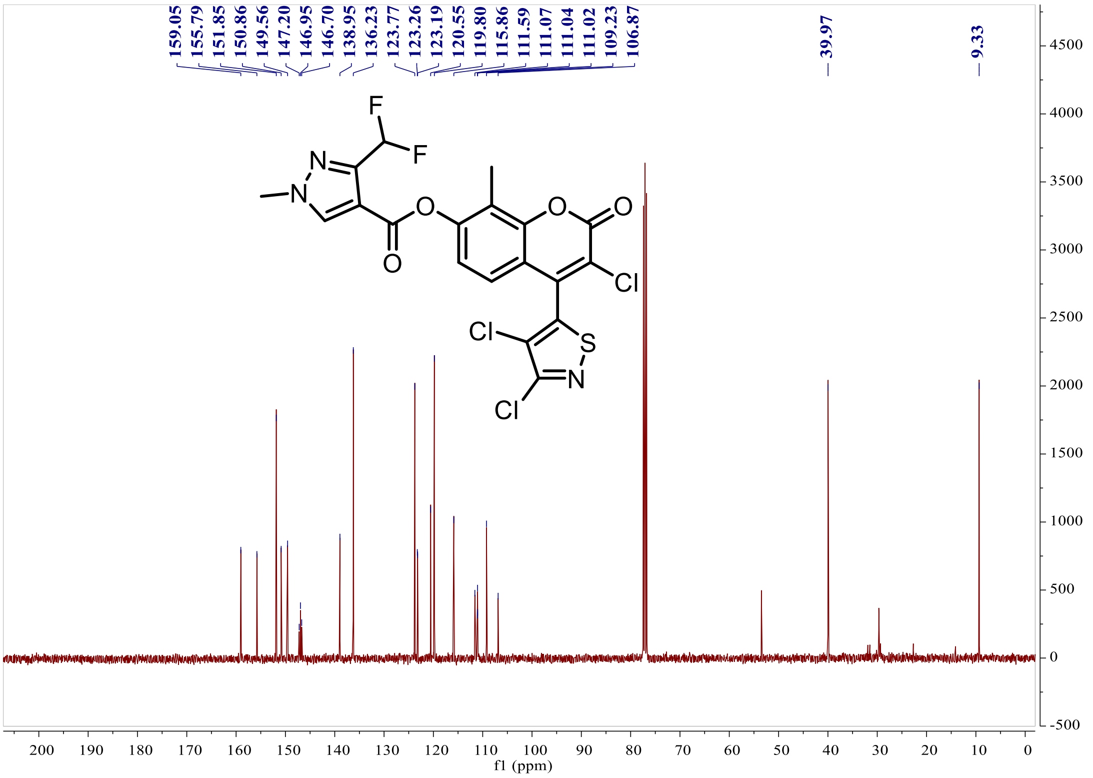

Figure S59. **The ^{13}C NMR (101MHz, Chloroform-*d*) of compound 2as.**

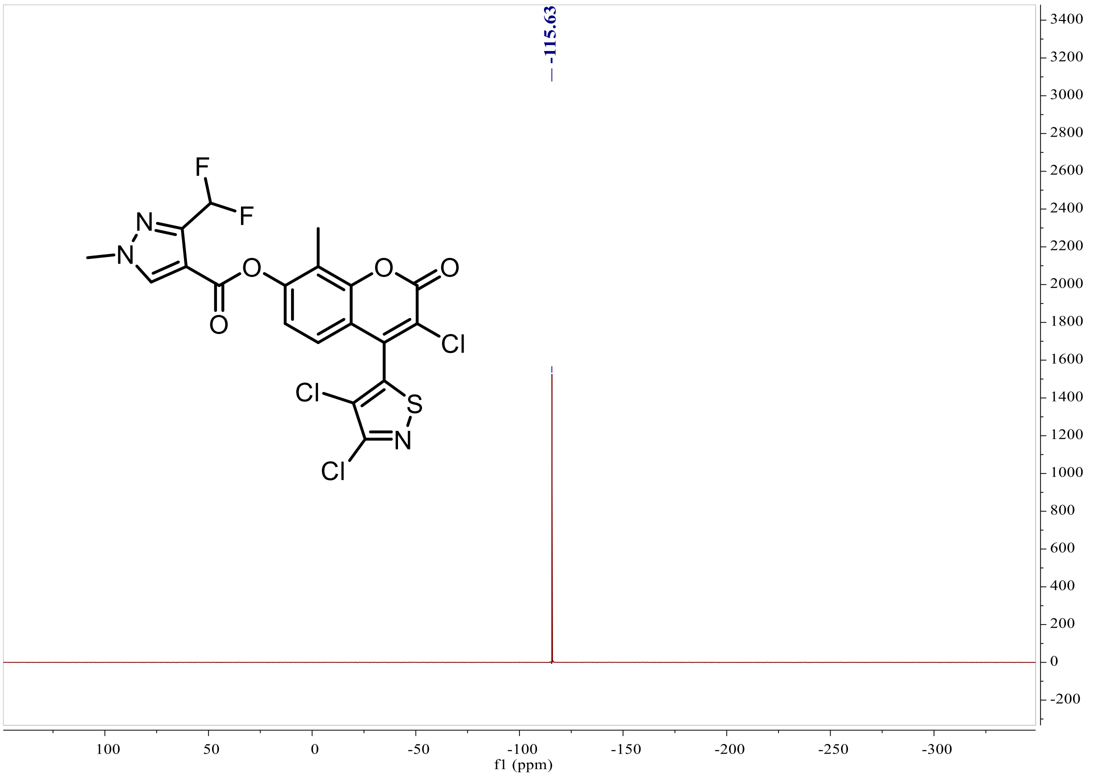

Figure S60. **The ^{19}F NMR (376MHz, Chloroform-*d*) of compound 2as.**

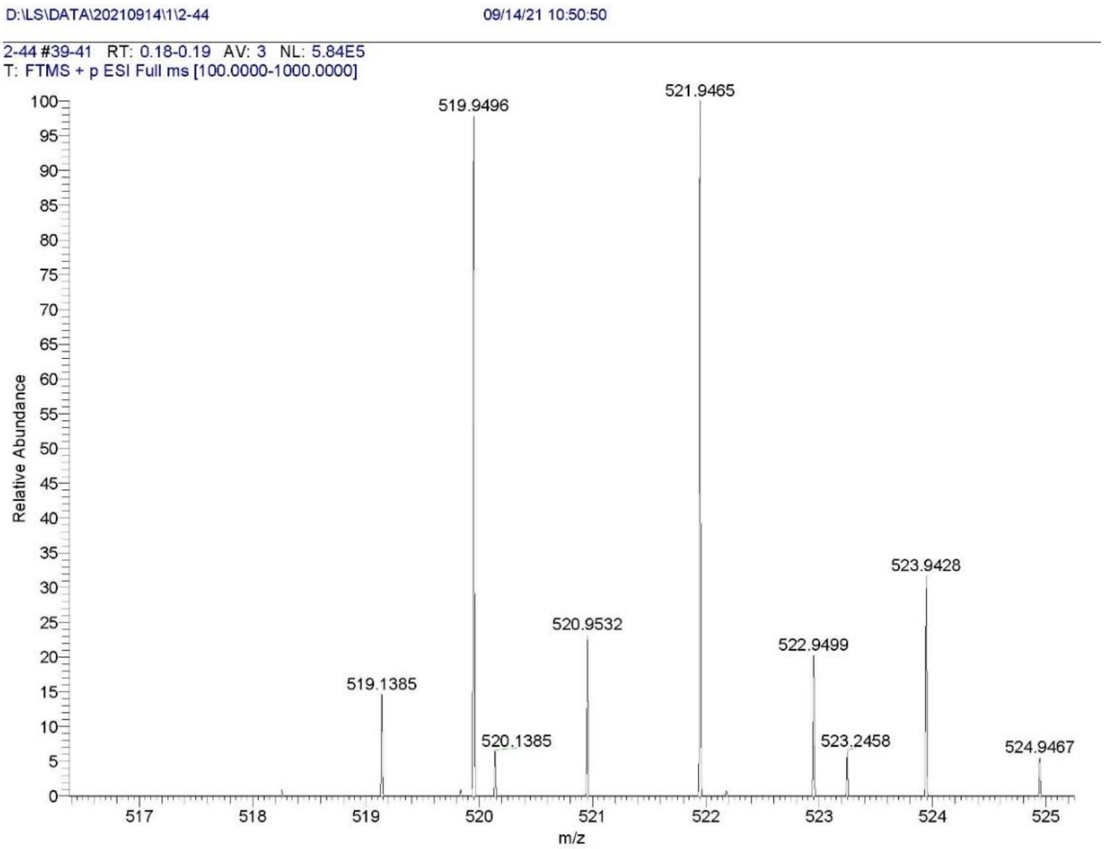

Figure S61. **The HRMS of compound 2as.**

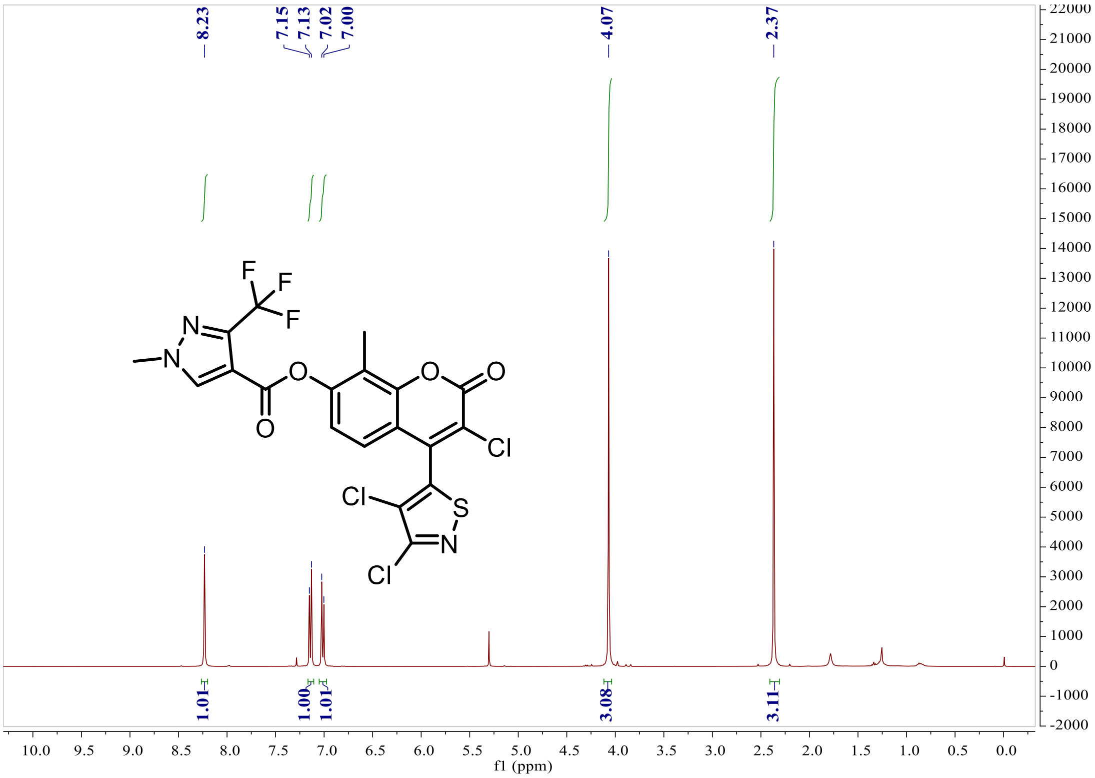

Figure S62. **The ^{13}H NMR (400MHz, Chloroform-*d*) of compound 2at.**

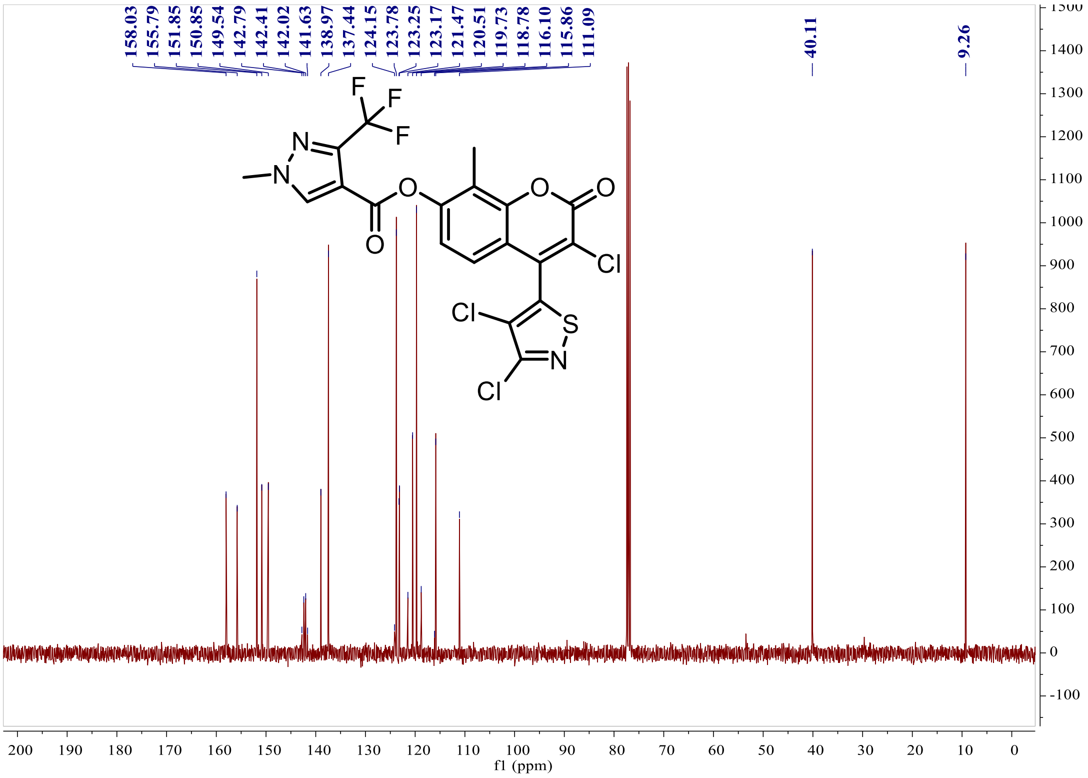

Figure S63. **The ^{13}C NMR (101MHz, Chloroform-*d*) of compound 2at.**

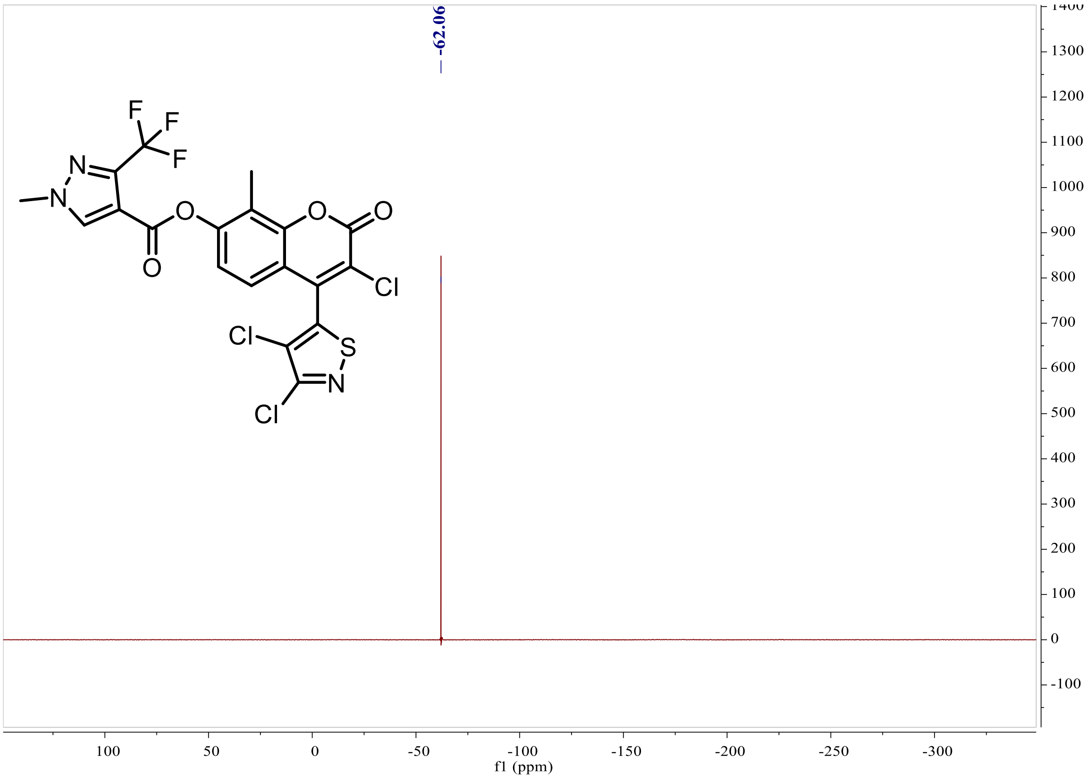

Figure S64. **The ^{19}F NMR (376MHz, Chloroform-*d*) of compound 2at.**

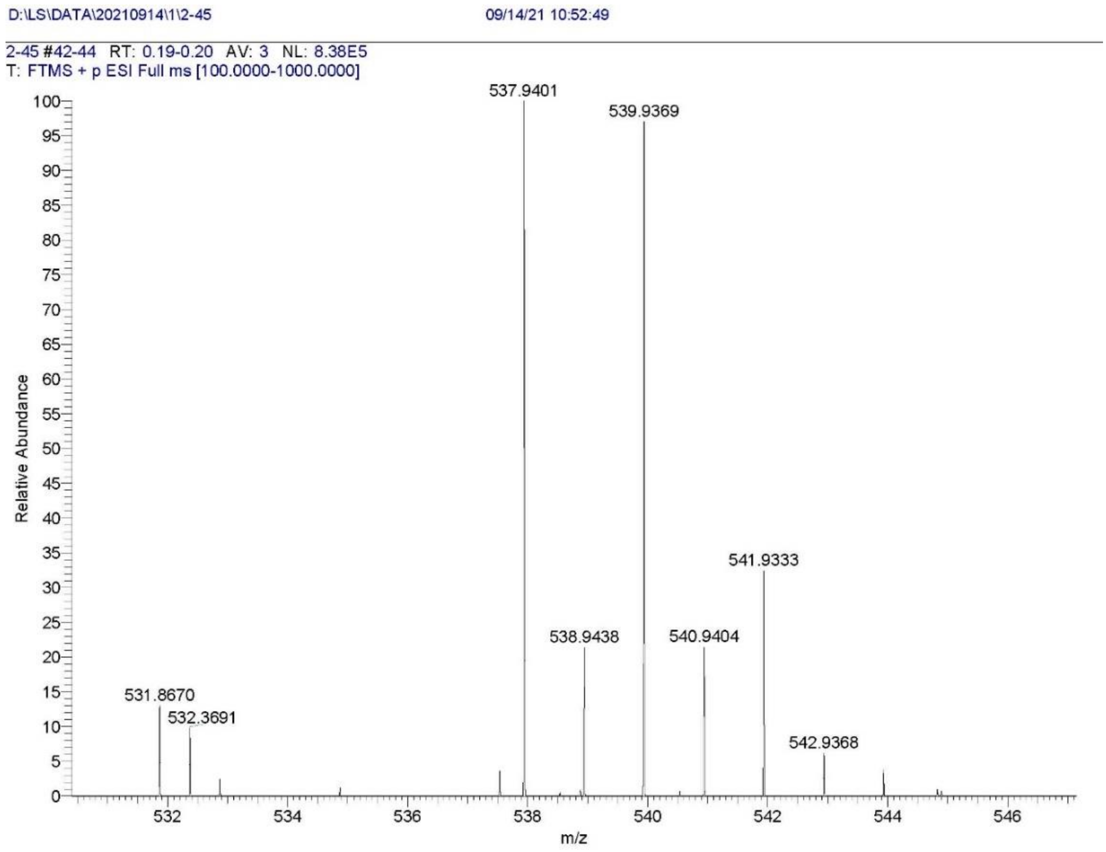

Figure S65. **The HRMS of compound 2at.**

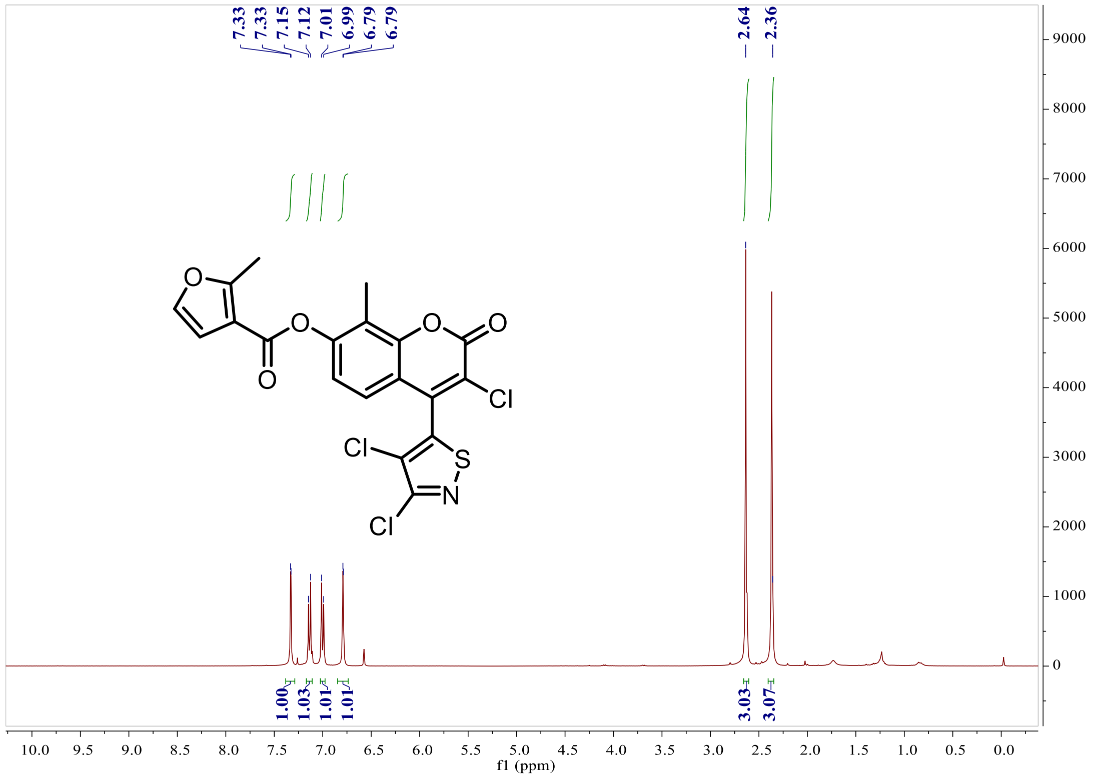

Figure S66. **The ^{1}H NMR (400MHz, Chloroform-*d*) of compound 2au.**

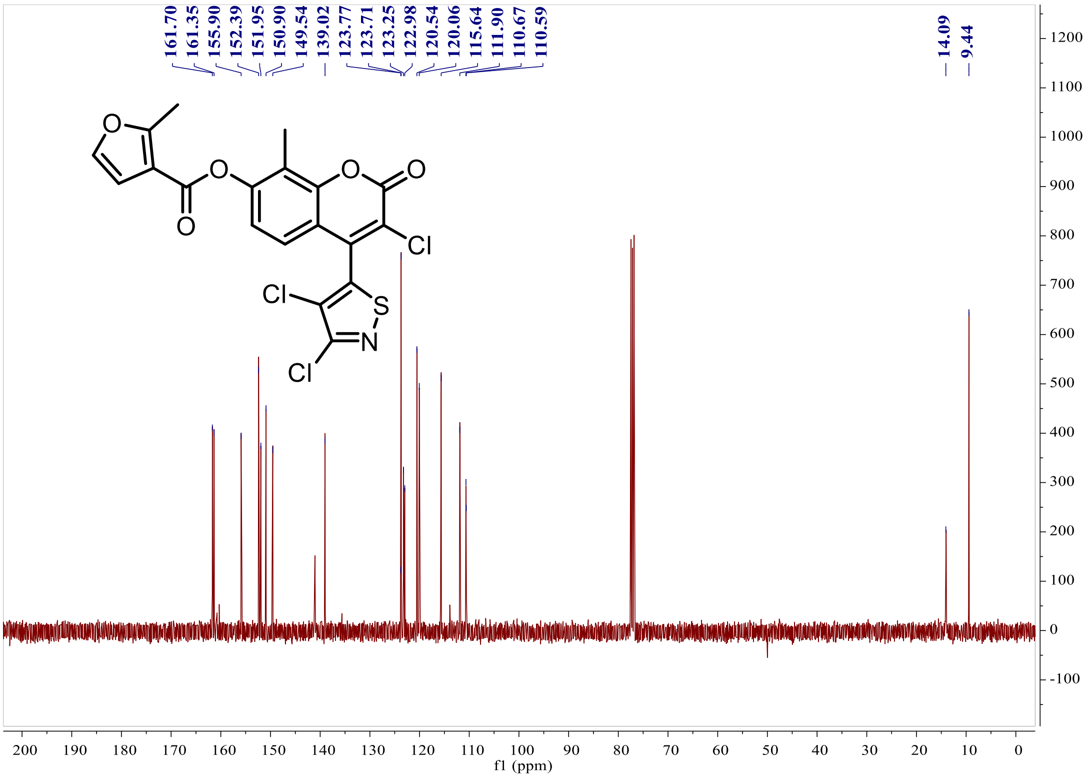

Figure S67. **The ^{13}C NMR (101MHz, Chloroform-*d*) of compound 2au.**

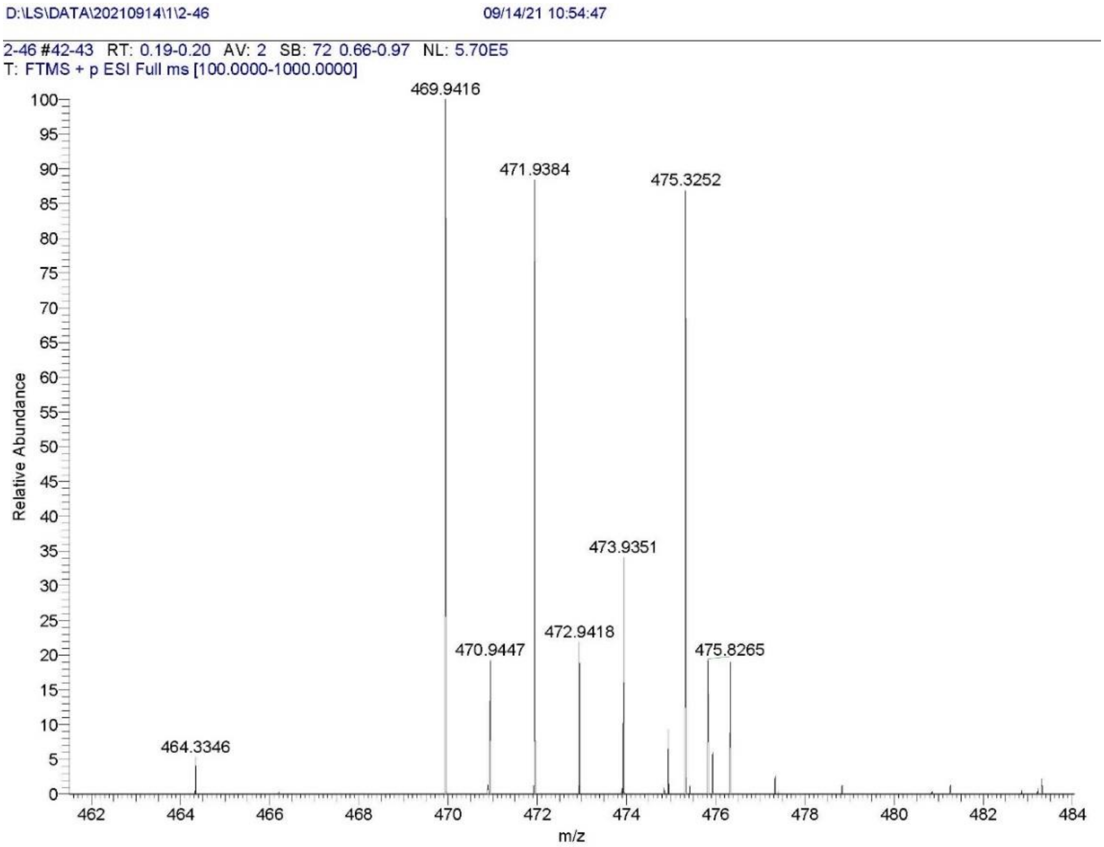

Figure S68. **The HRMS of compound 2au.**

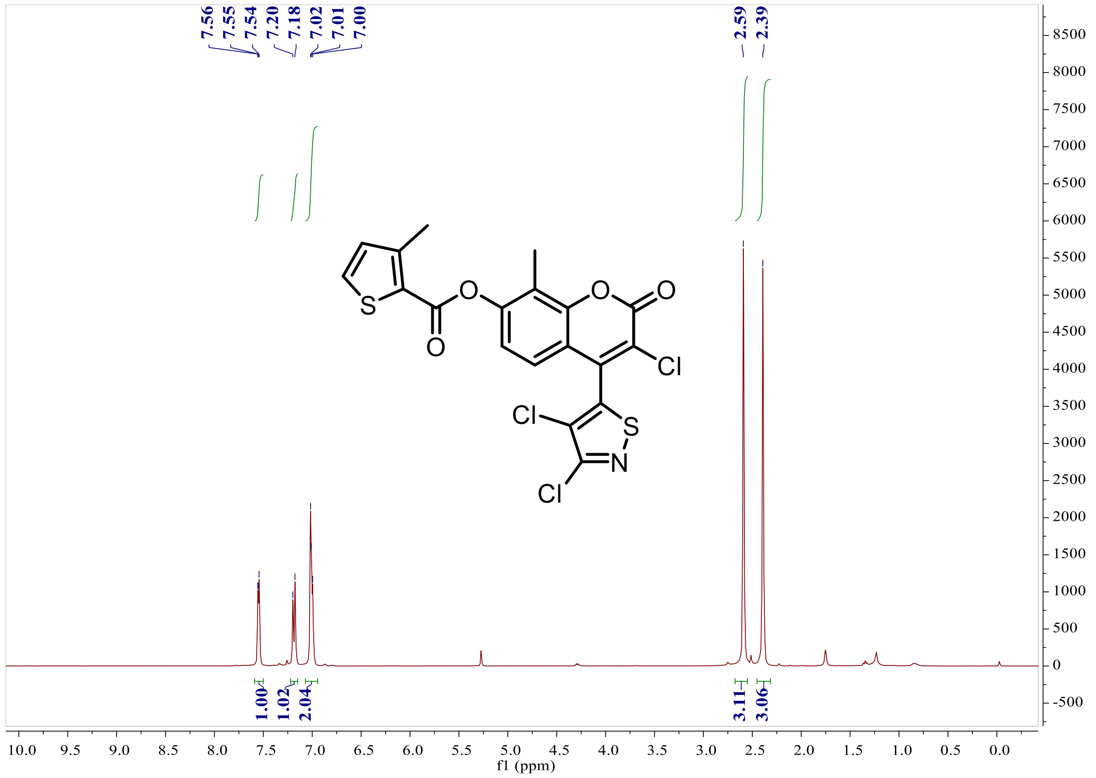

Figure S69. **The ^{1}H NMR (400MHz, Chloroform-*d*) of compound 2av.**

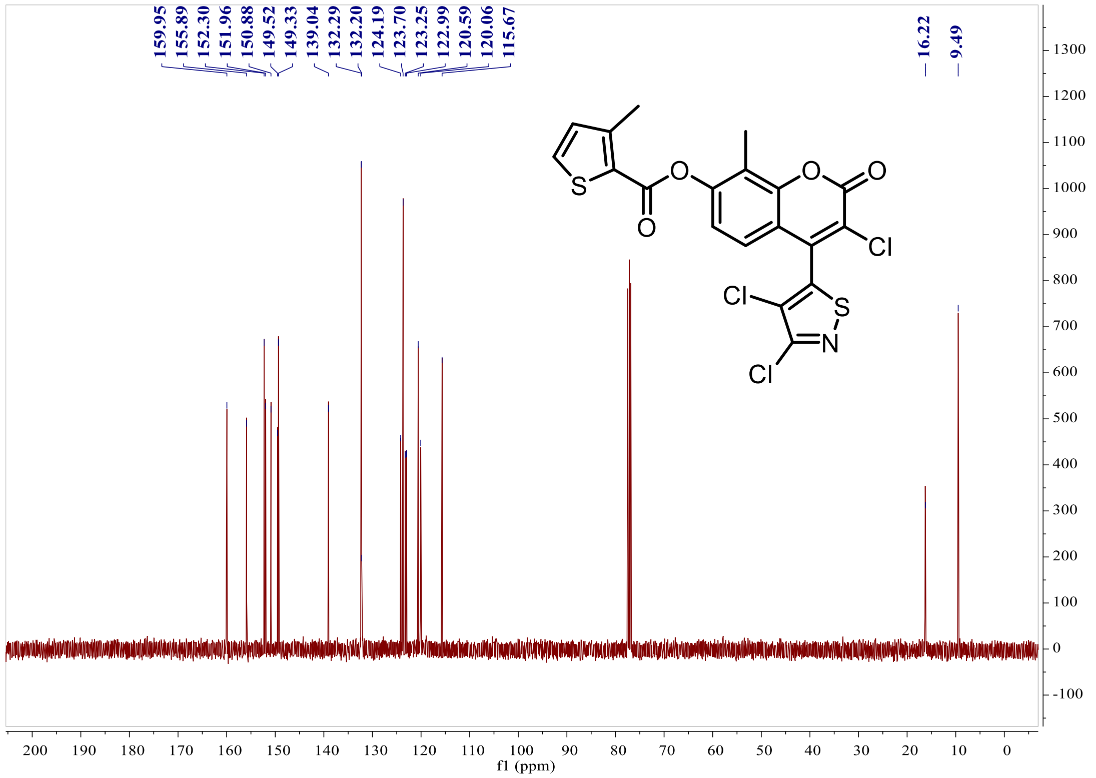

Figure S70. **The ^{13}C NMR (101MHz, Chloroform-*d*) of compound 2av.**

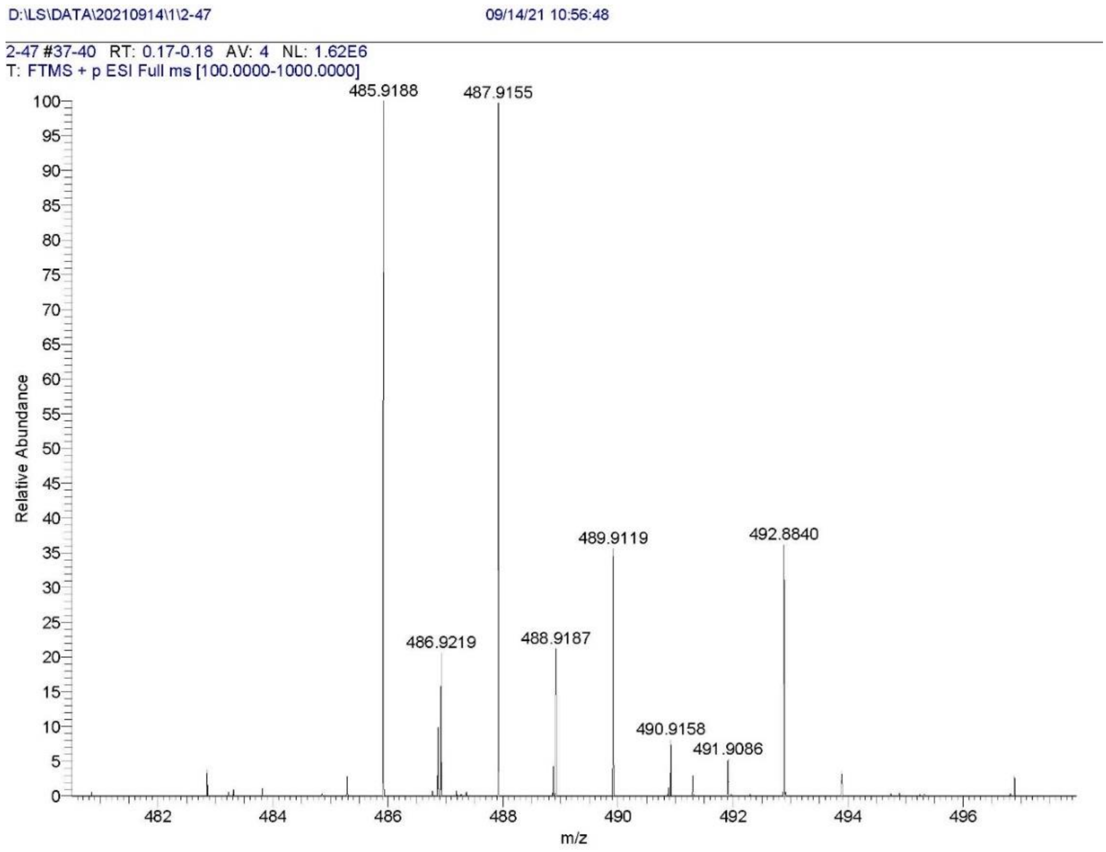

Figure S71. **The HRMS of compound 2av.**

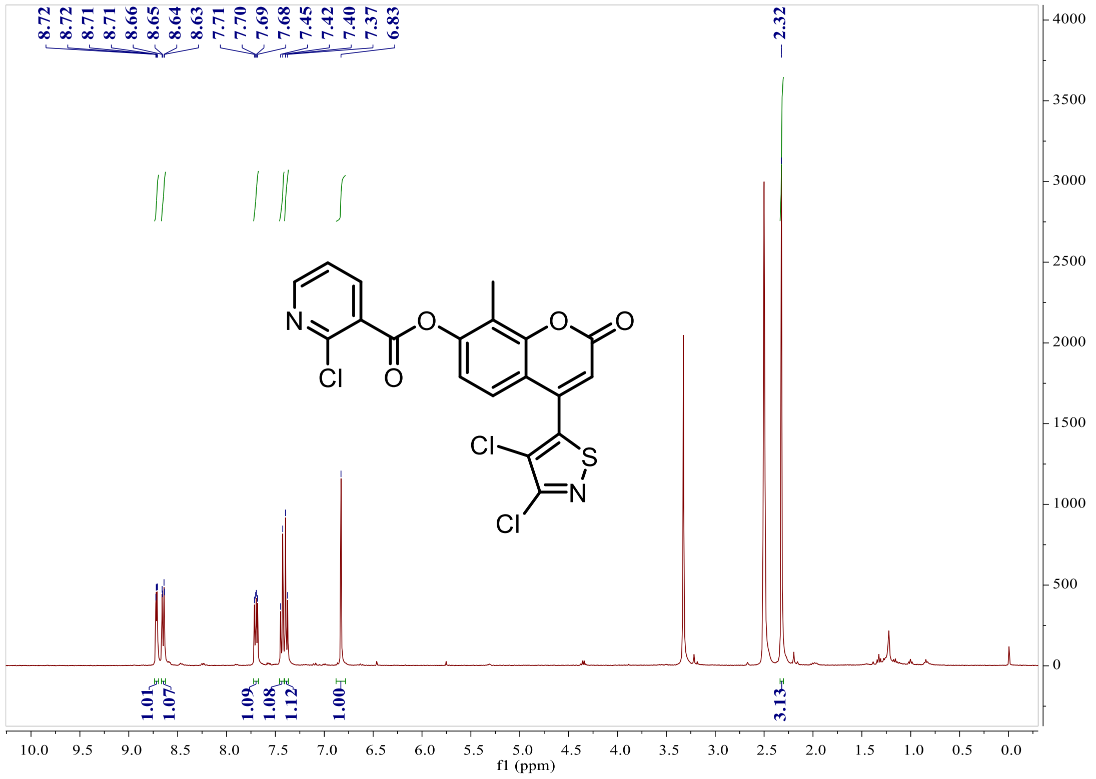

Figure S72. **The ^{1}H NMR (400MHz, Chloroform-*d*) of compound 2ba.**

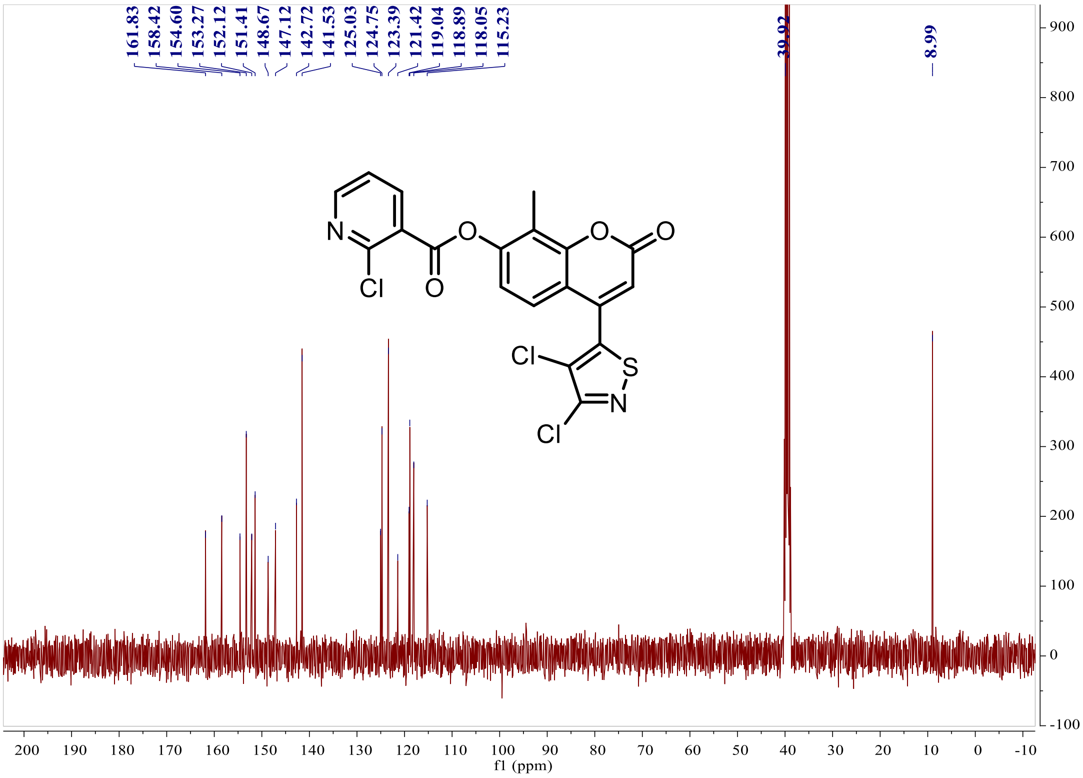

Figure S73. **The ^{13}C NMR (101MHz, Chloroform-*d*) of compound 2ba.**

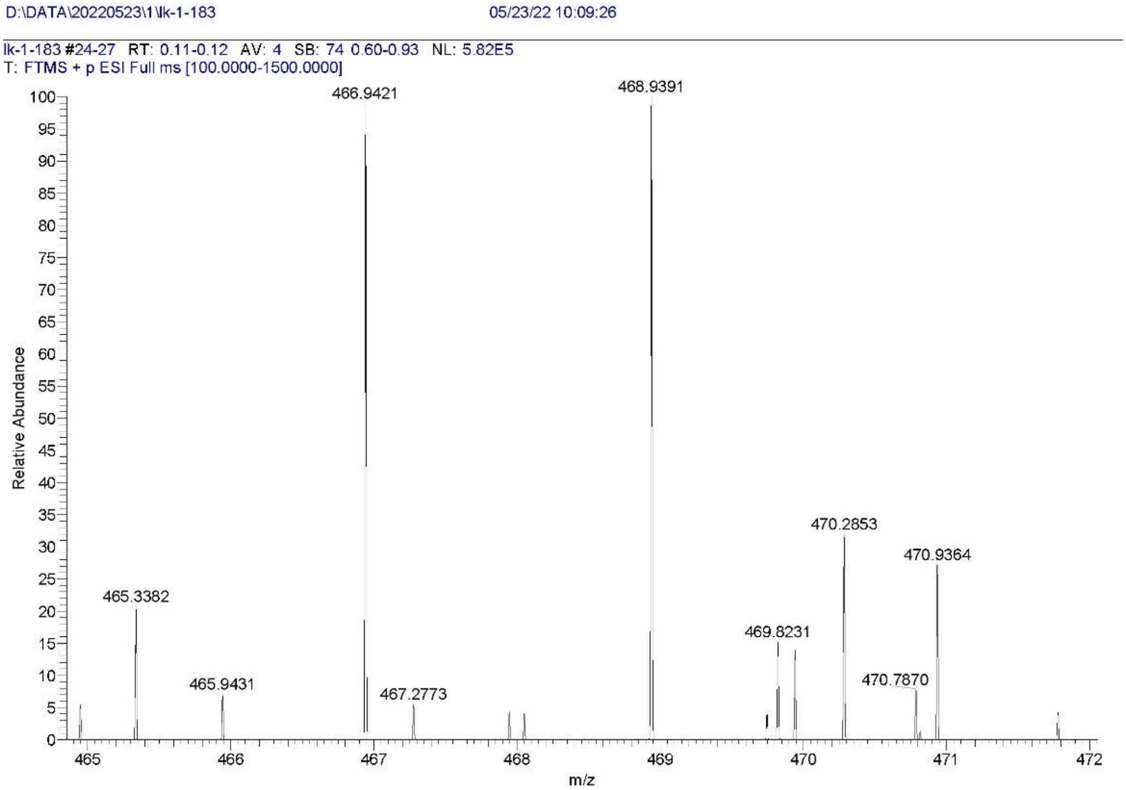

Figure S74. **The HRMS of compound 2ba.**

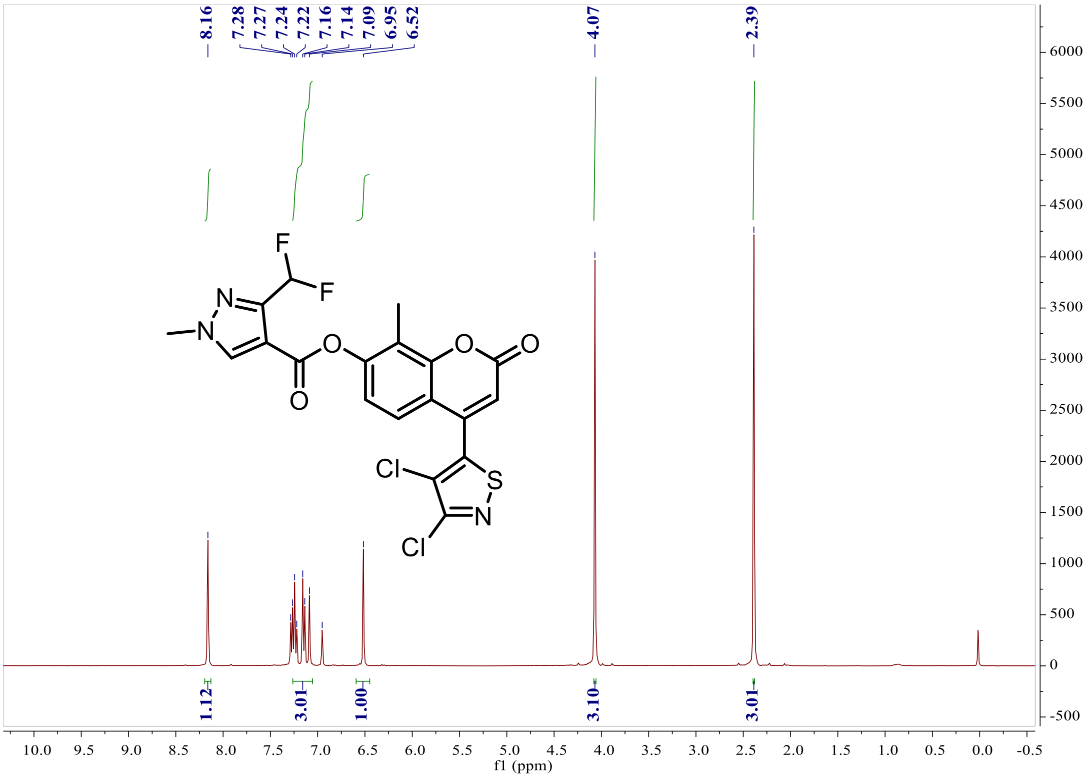

Figure S75. **The ^{1}H NMR (400MHz, Chloroform-*d*) of compound 2bb.**

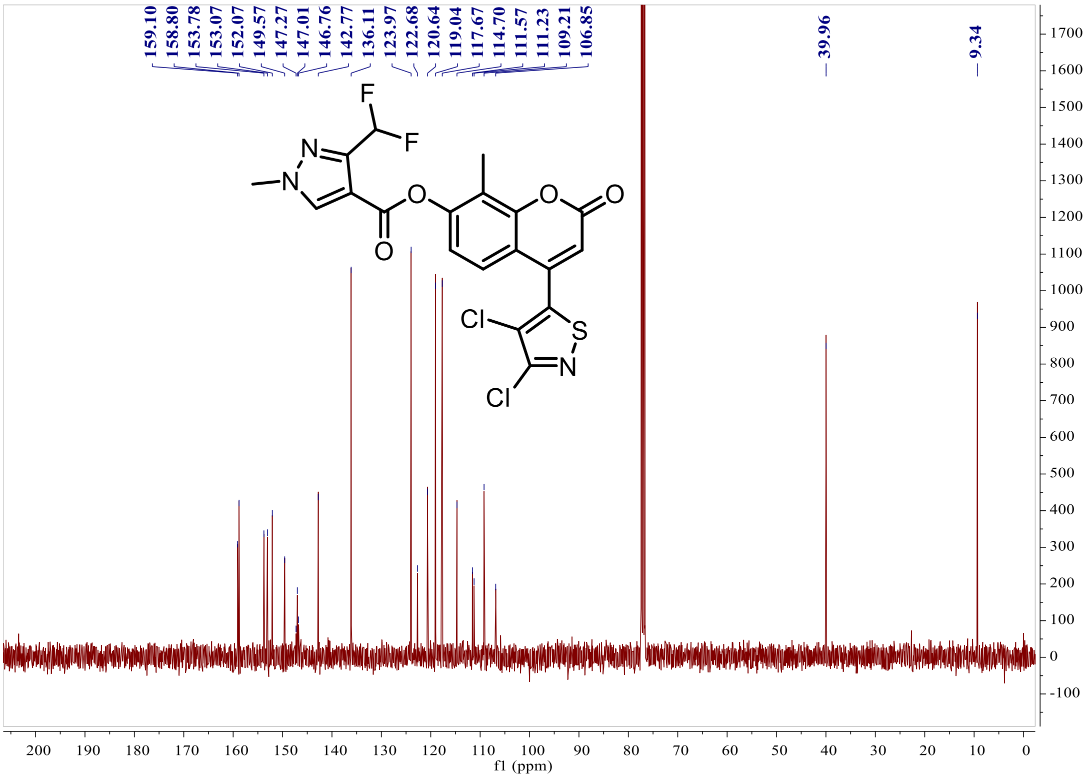

Figure S76. **The ^{13}C NMR (101MHz, Chloroform-*d*) of compound 2bb.**

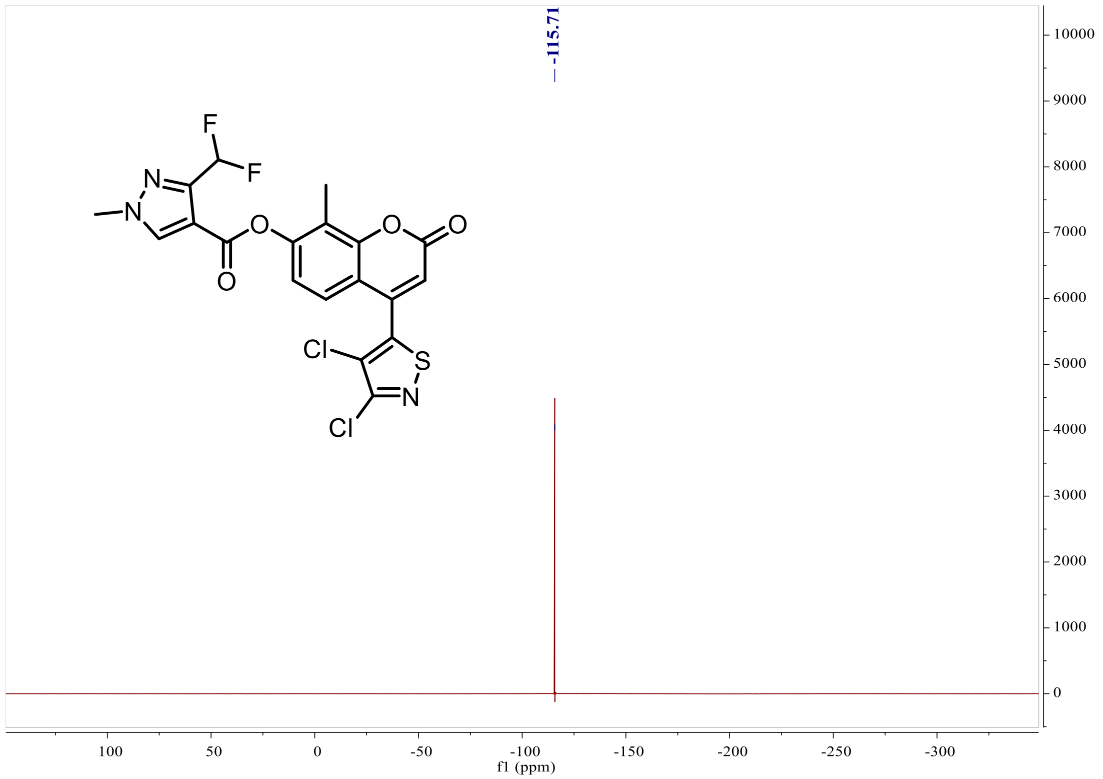

Figure S77. **The ^{19}F NMR (376MHz, Chloroform-*d*) of compound 2bb.**

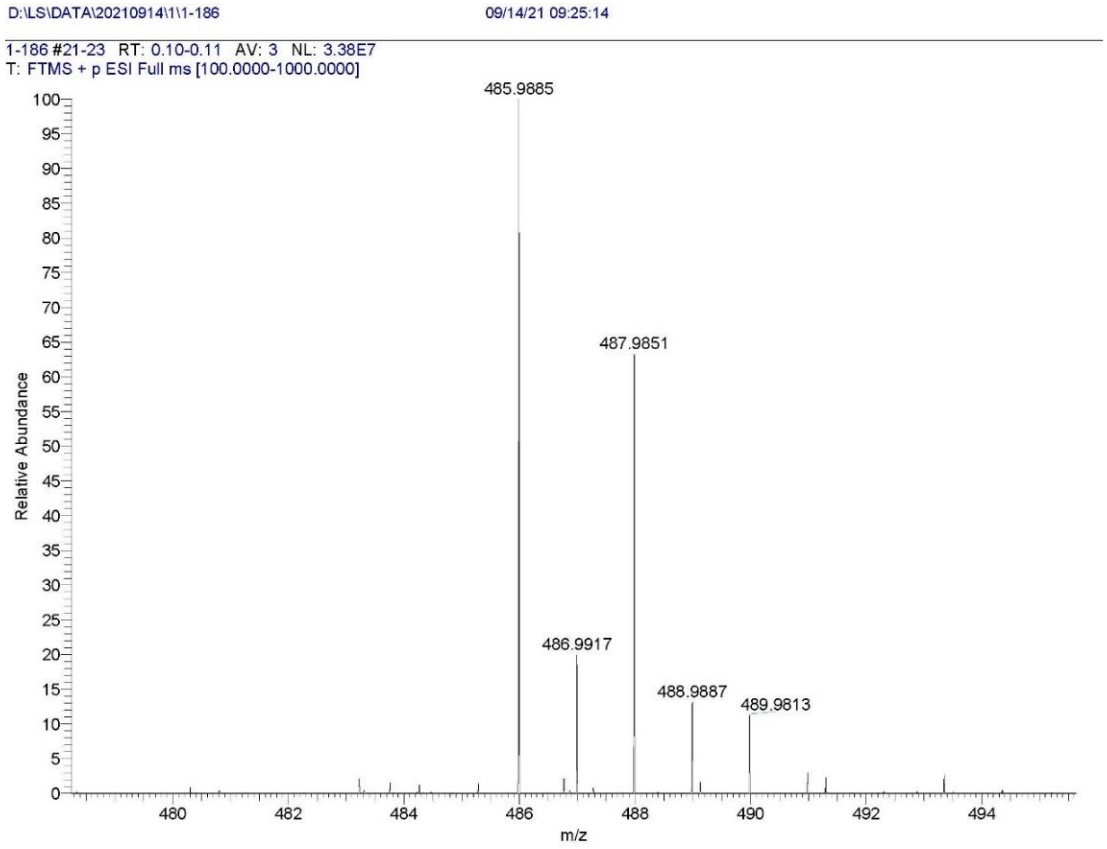

Figure S78. **The HRMS of compound 2bb.**

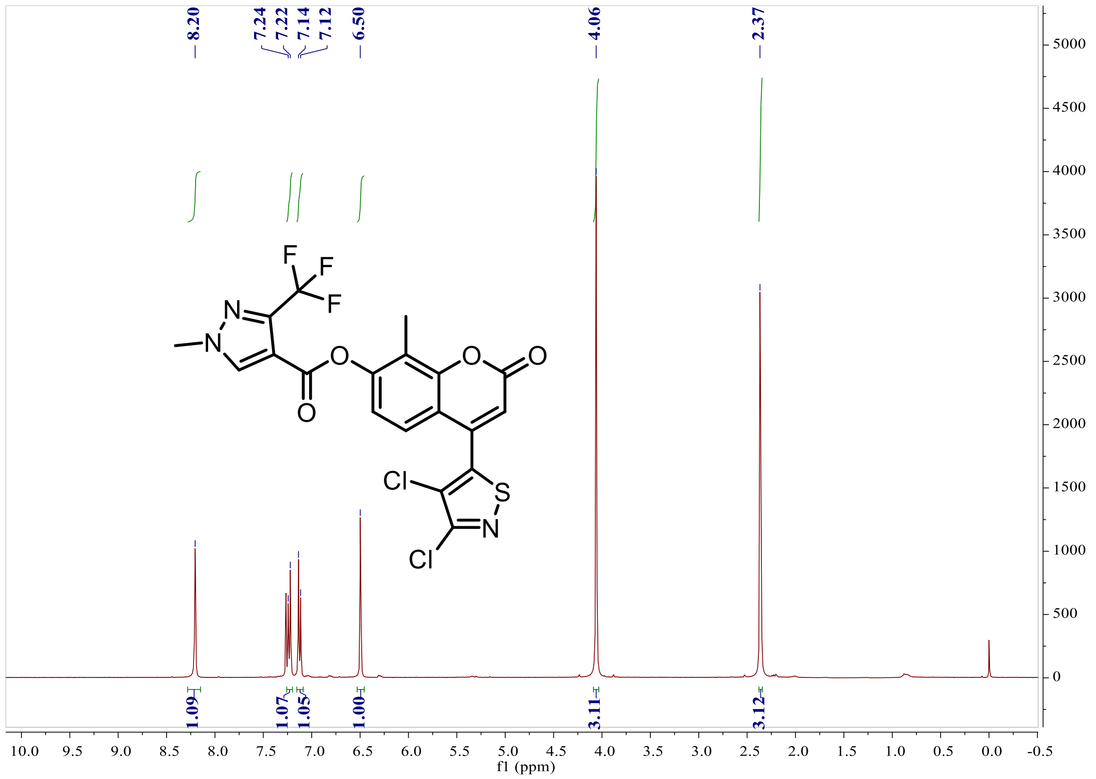

Figure S79. **The ^{1}H NMR (400MHz, Chloroform-*d*) of compound 2bc.**

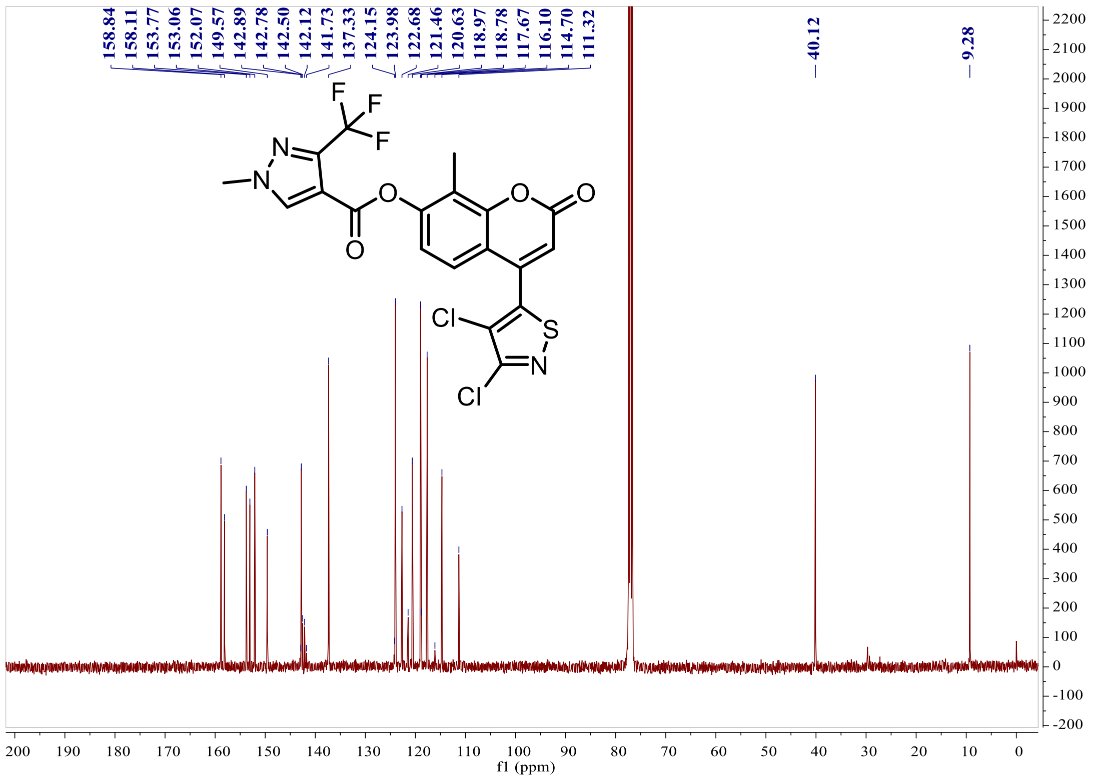

Figure S80. **The ^{13}C NMR (101MHz, Chloroform-*d*) of compound 2bc.**

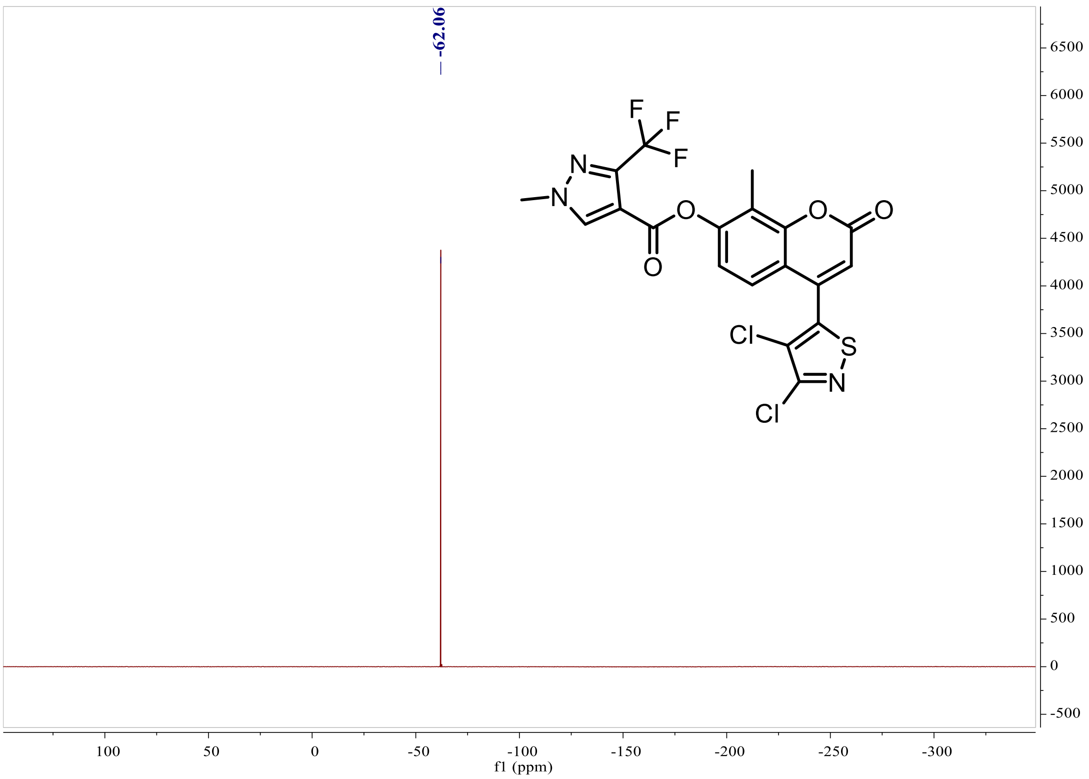

Figure S81. **The ^{19}F NMR (376MHz, Chloroform-*d*) of compound 2bc.**

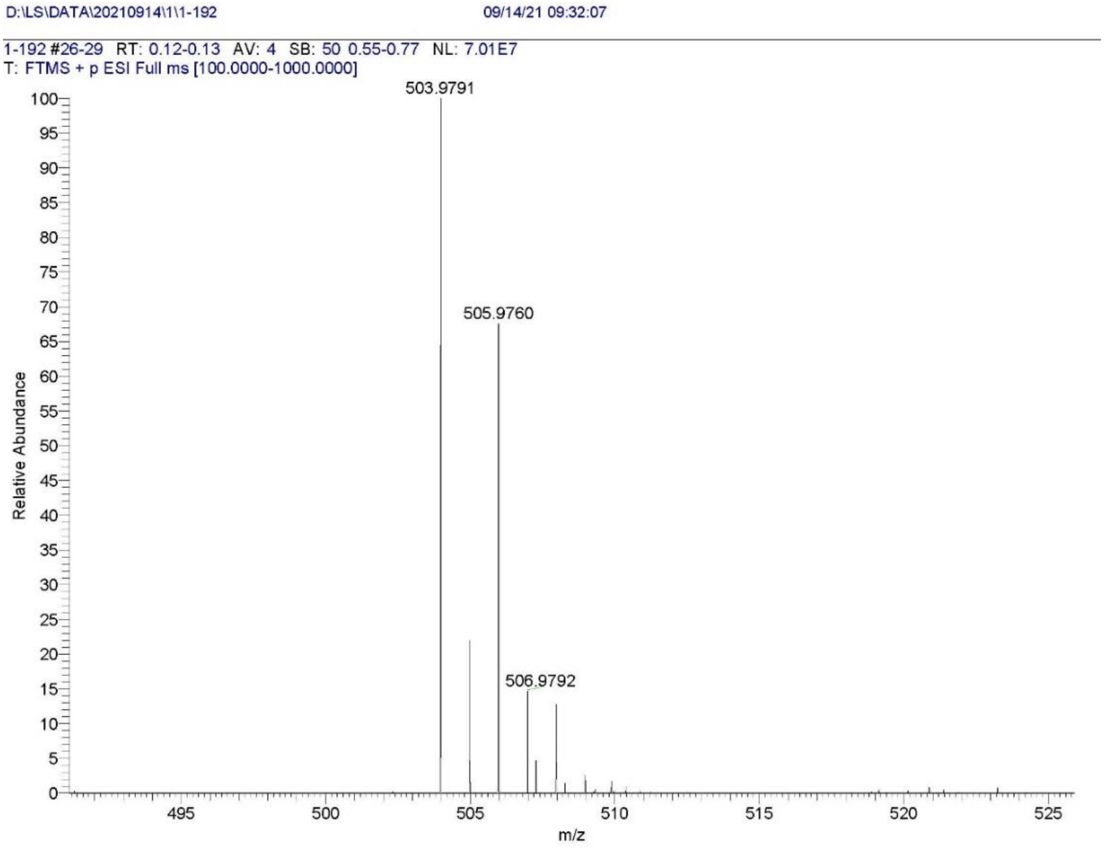

Figure S82. **The HRMS of compound 2bc.**

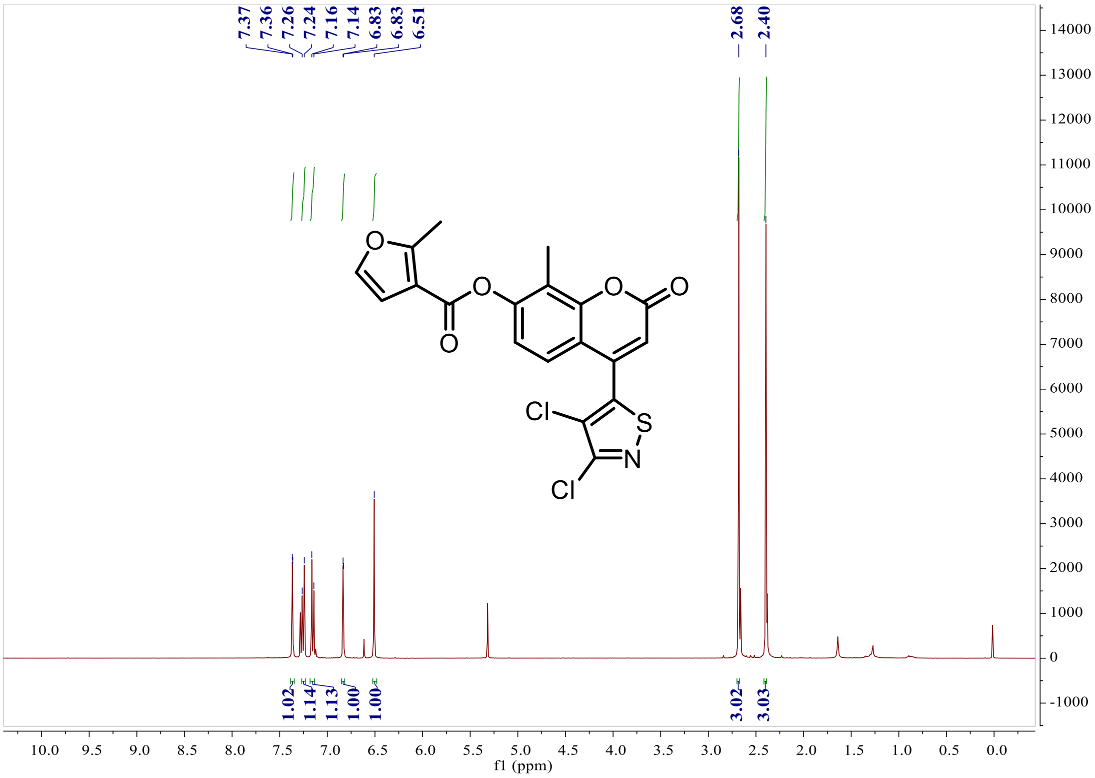

Figure S83. **The ^{1}H NMR (400MHz, Chloroform-*d*) of compound 2bd.**

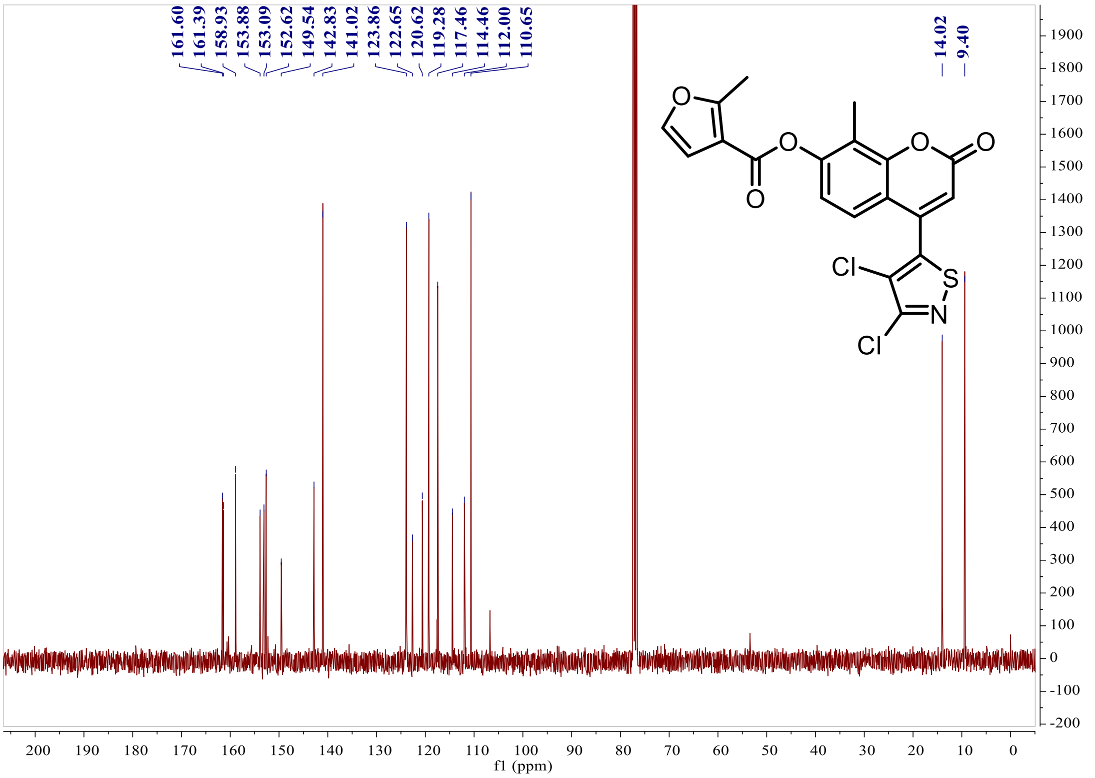

Figure S84. **The ^{13}C NMR (101MHz, Chloroform-*d*) of compound 2bd.**

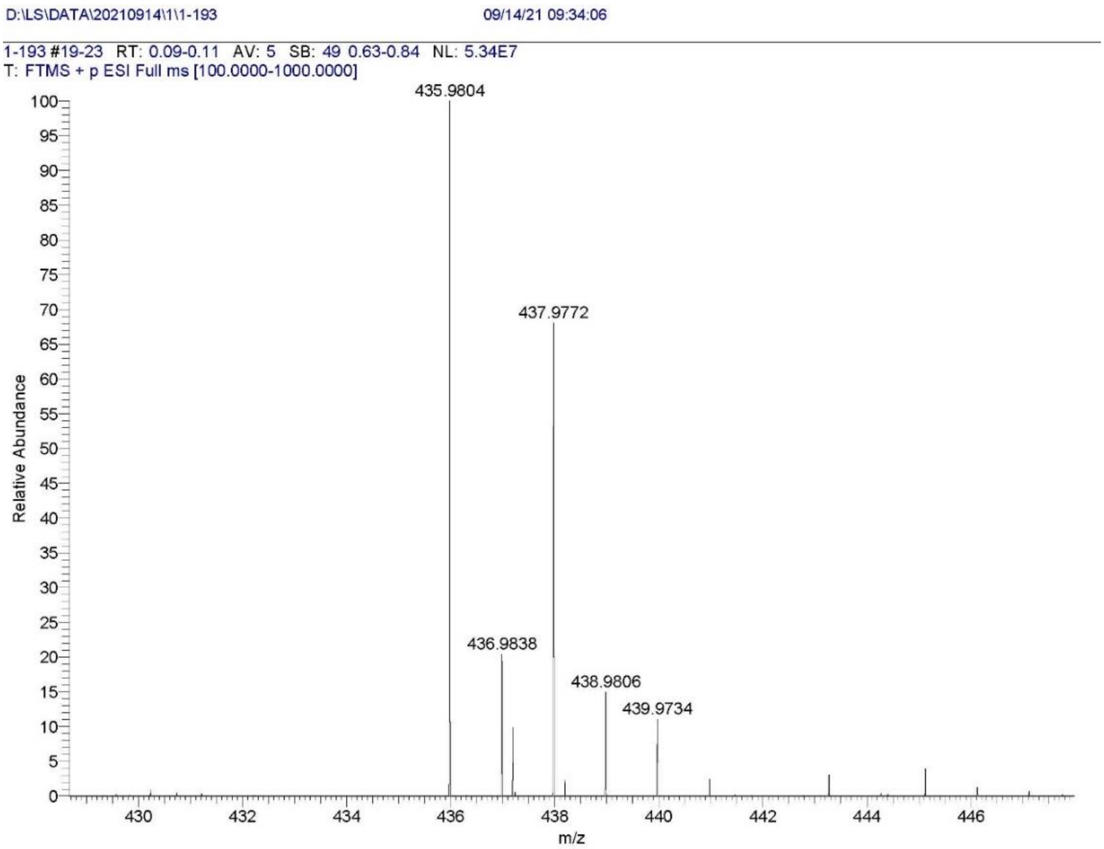

Figure S85. **The HRMS of compound 2bd.**

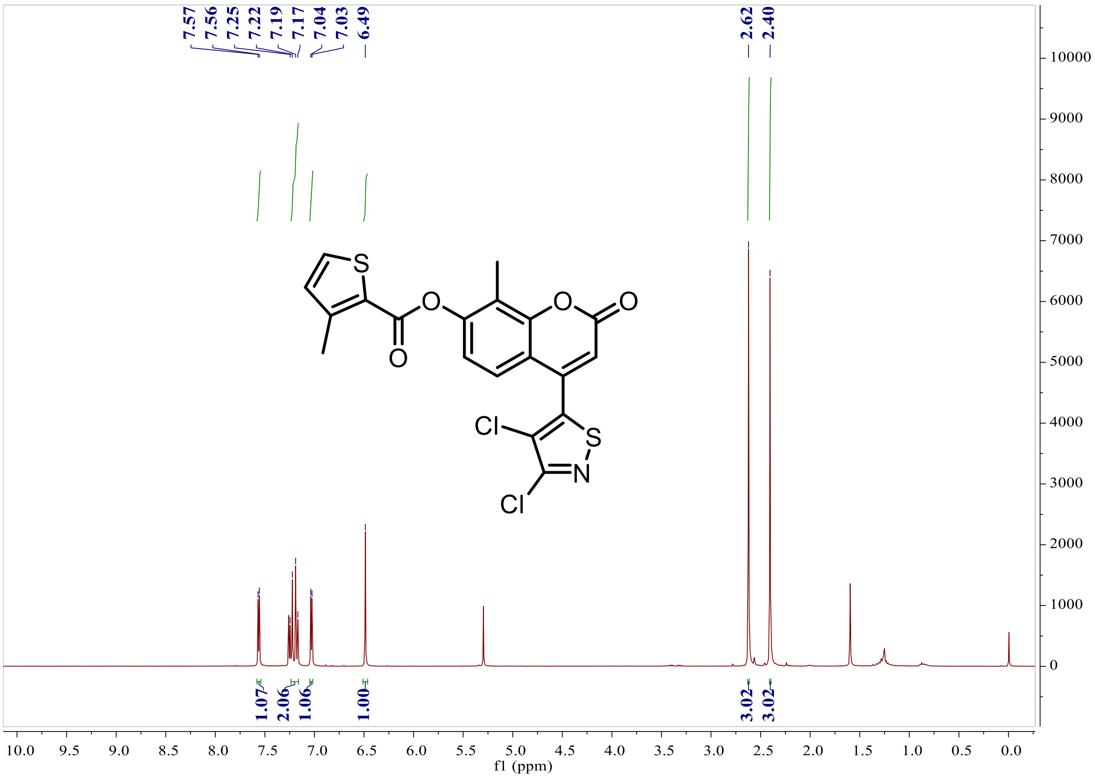

Figure S86. **The ^{1}H NMR (400MHz, Chloroform-*d*) of compound 2be.**

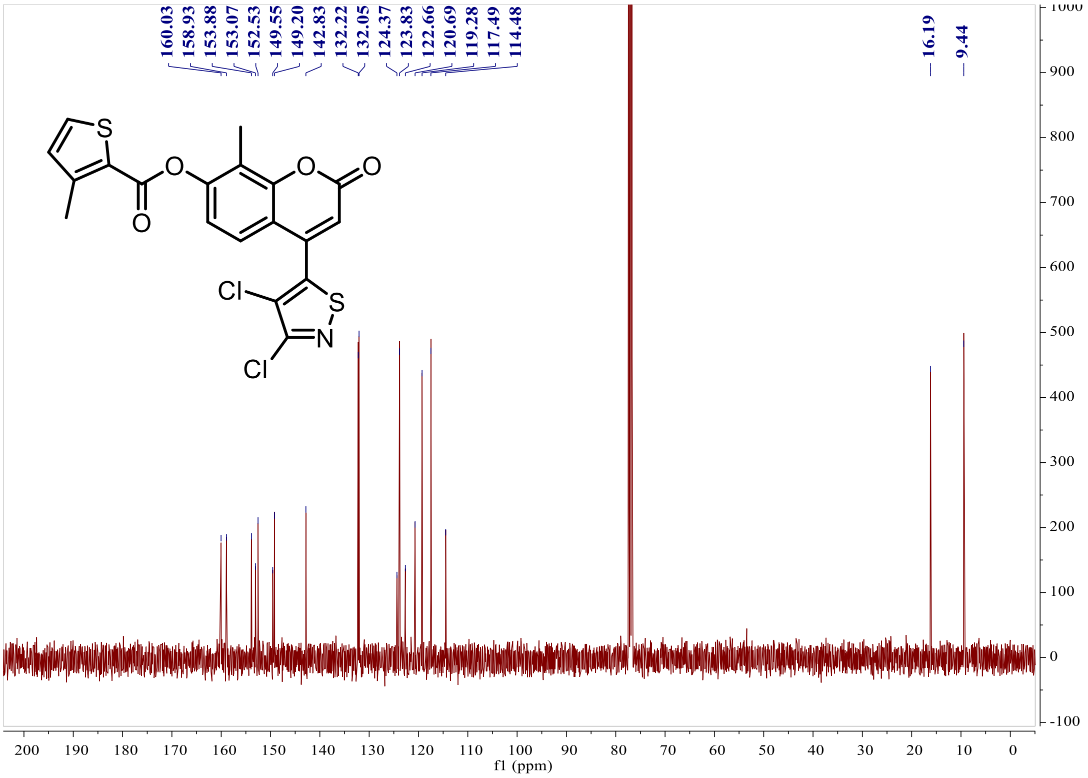

Figure S87. **The ^{13}C NMR (101MHz, Chloroform-*d*) of compound 2be.**

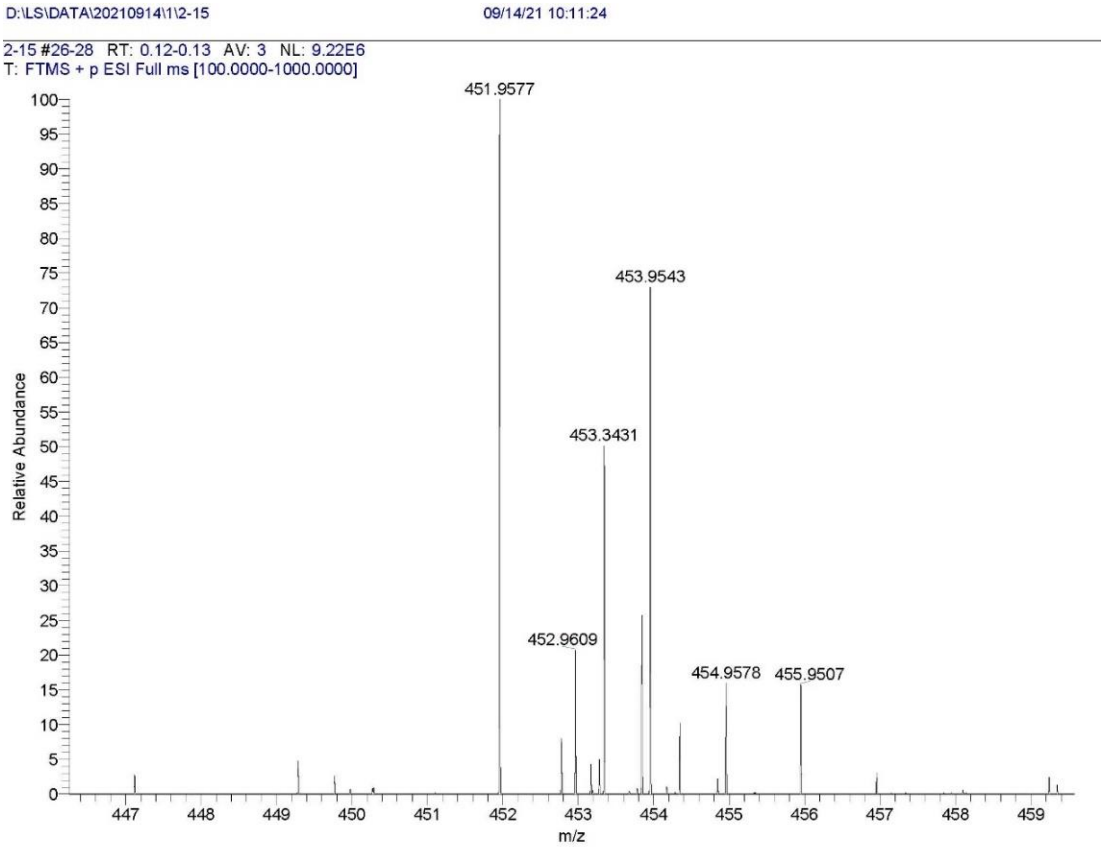

Figure S88. **The HRMS of compound 2be.**

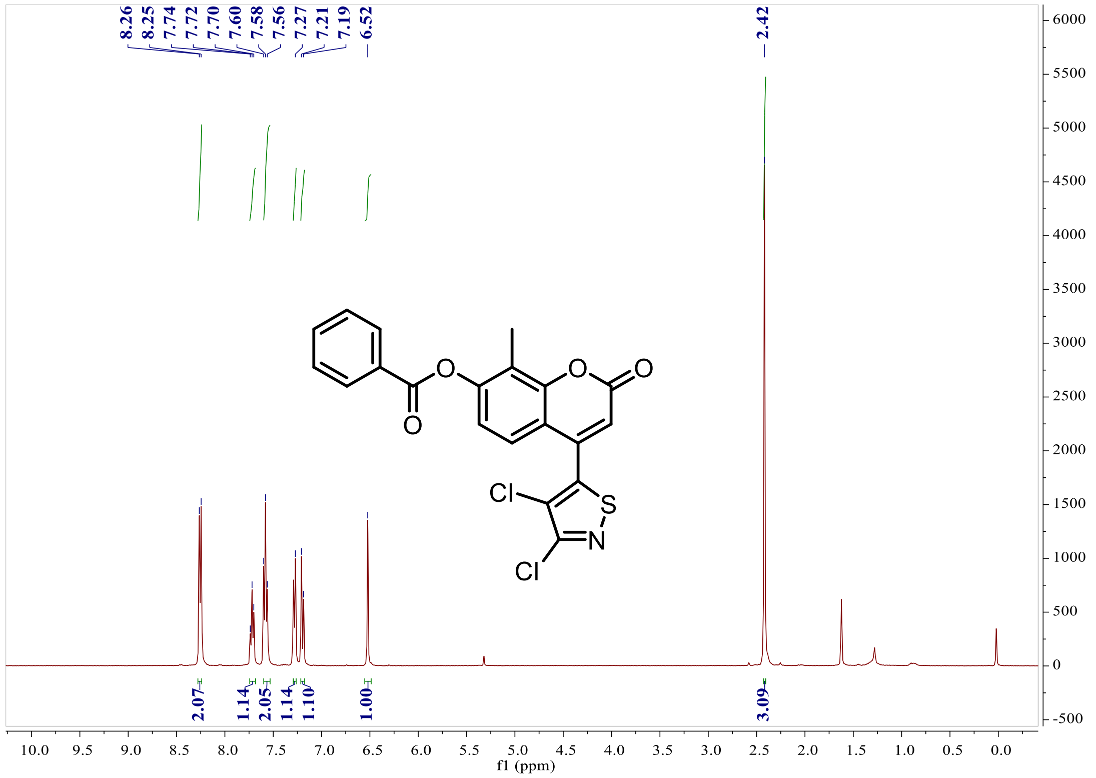

Figure S89. **The ^{1}H NMR (400MHz, Chloroform-*d*) of compound 2bf.**

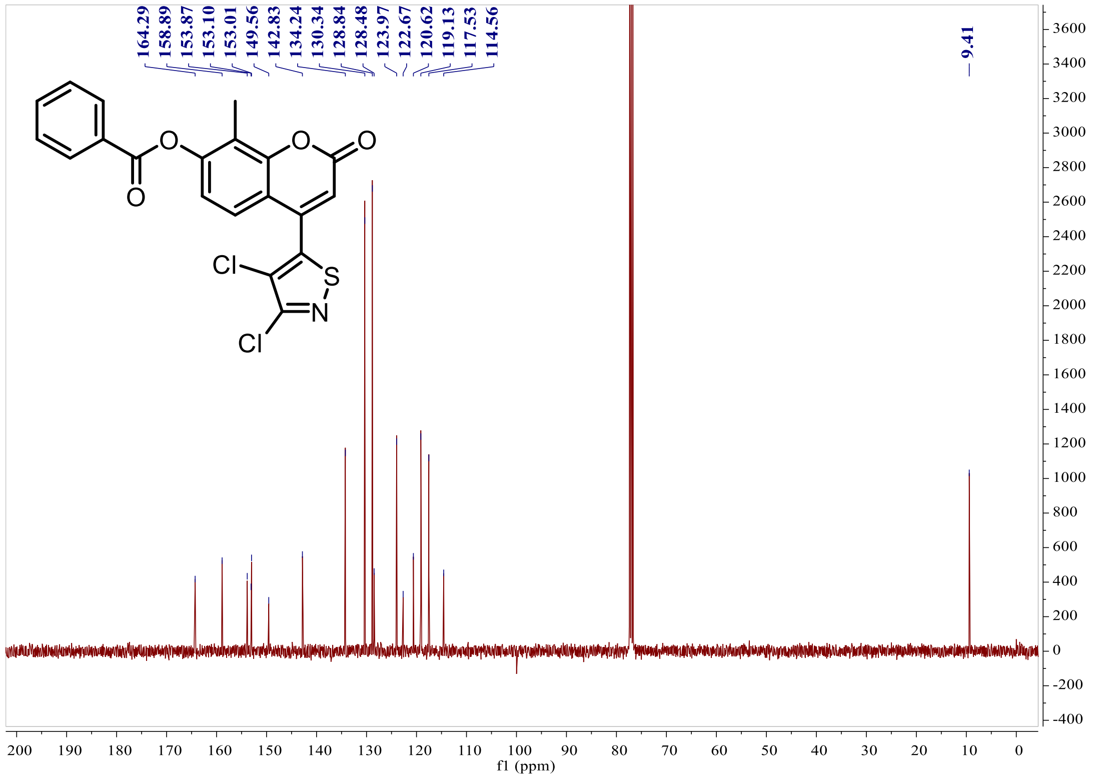

Figure S90. **The ^{13}C NMR (101MHz, Chloroform-*d*) of compound 2bf.**

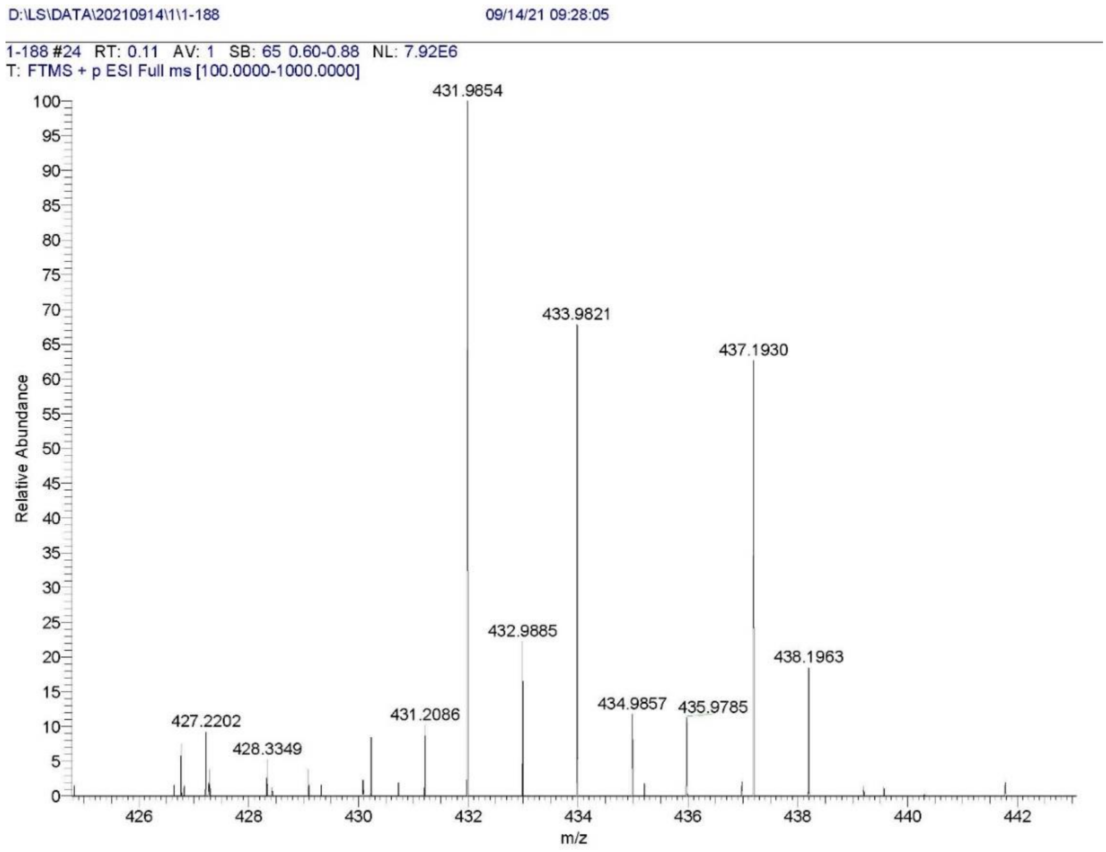

Figure S91. **The HRMS of compound 2bf.**

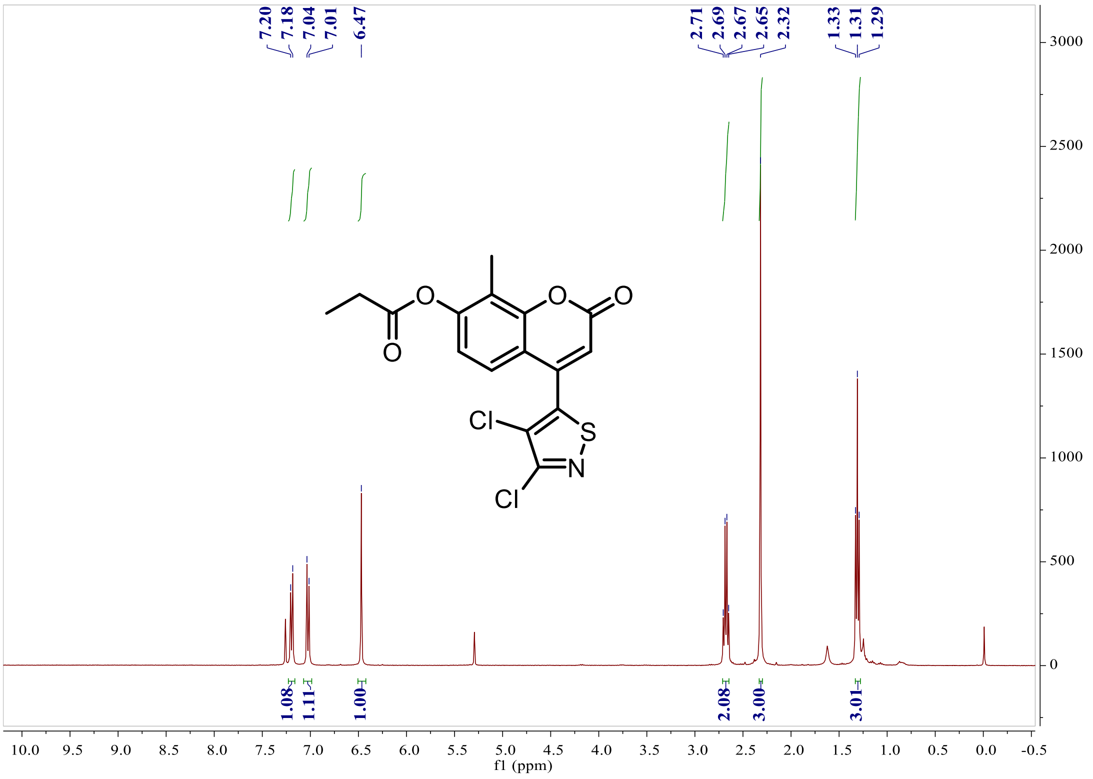

Figure S92. **The ^{1}H NMR (400MHz, Chloroform-*d*) of compound 2bg.**

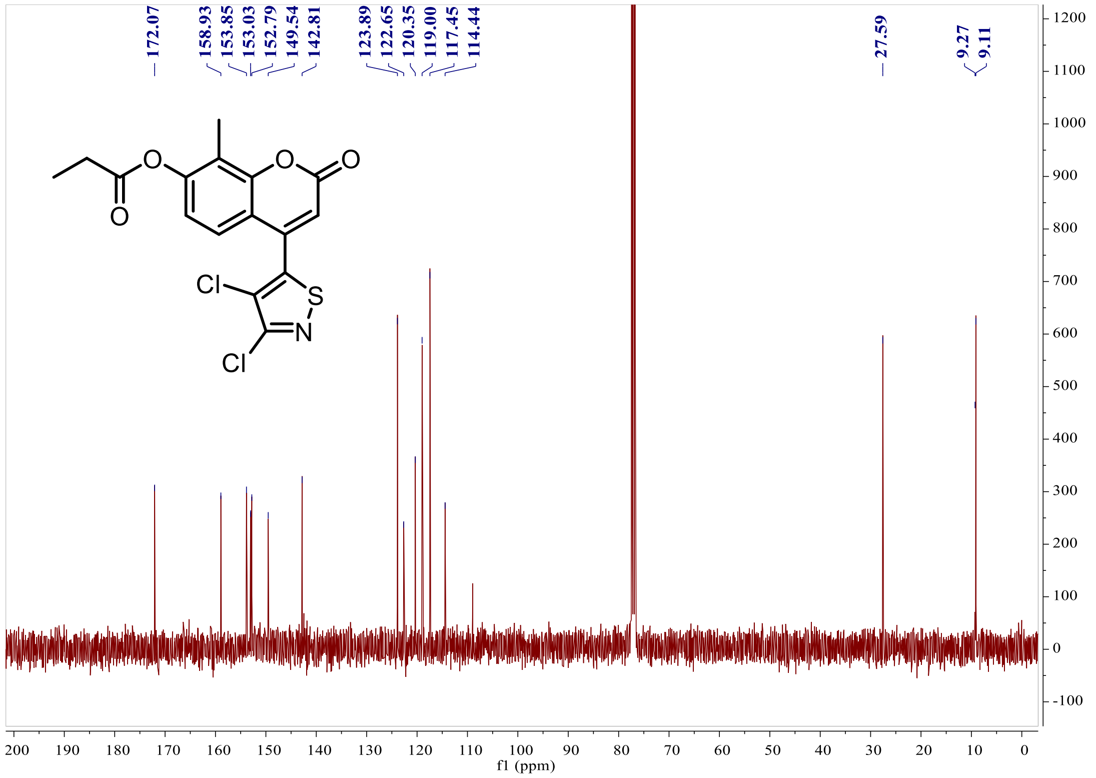

Figure S93. **The ^{13}C NMR (101MHz, Chloroform-*d*) of compound 2bg.**

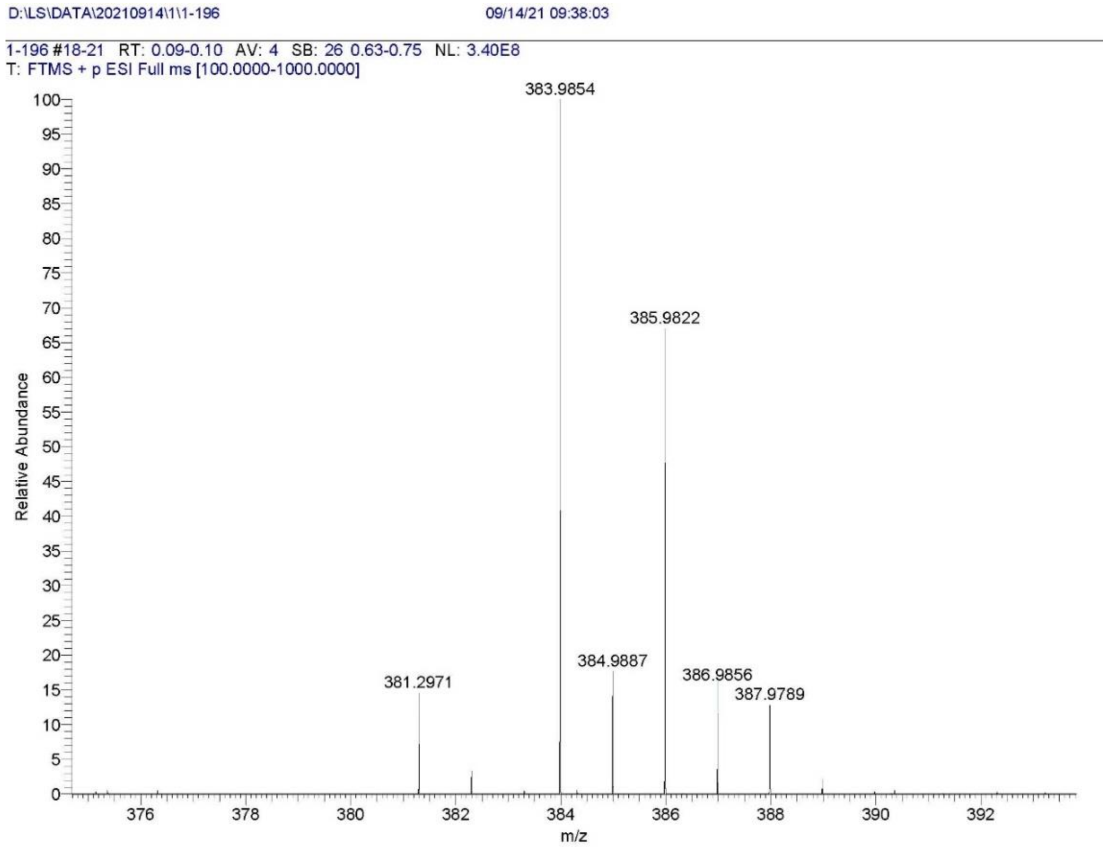

Figure S94. **The HRMS of compound 2bg.**

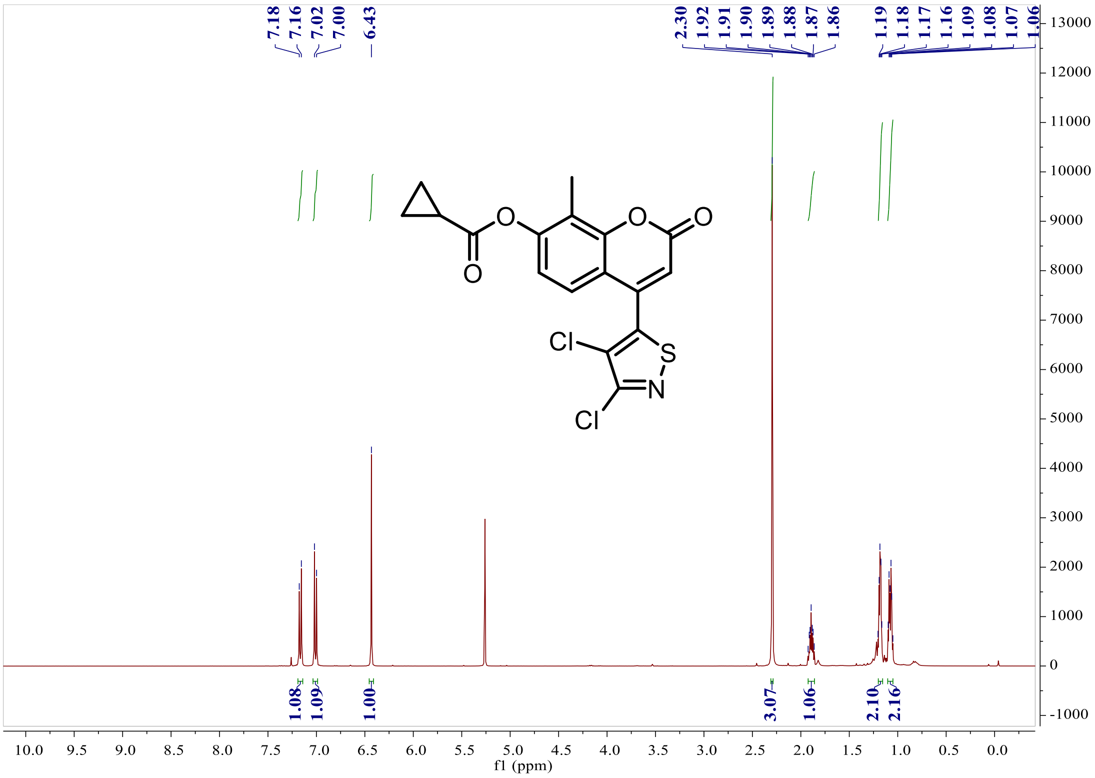

Figure S95. **The ^{1}H NMR (400MHz, Chloroform-*d*) of compound 2bh.**

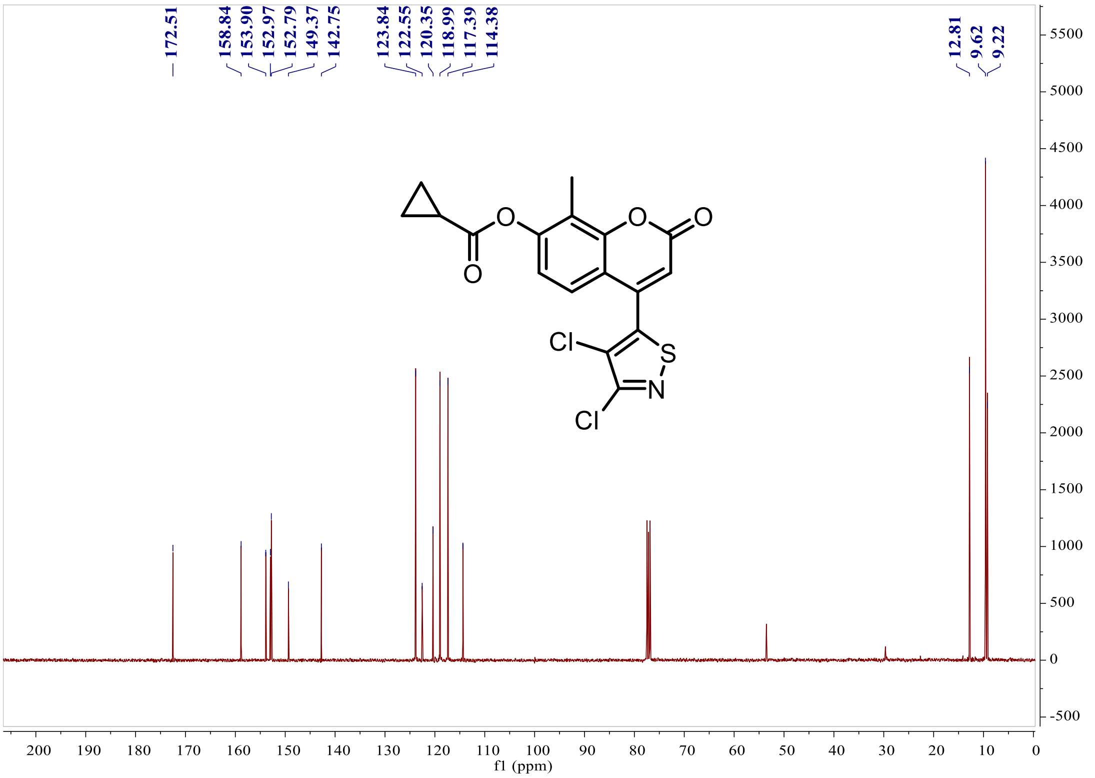

Figure S96. **The ^{13}C NMR (101MHz, Chloroform-*d*) of compound 2bh.**

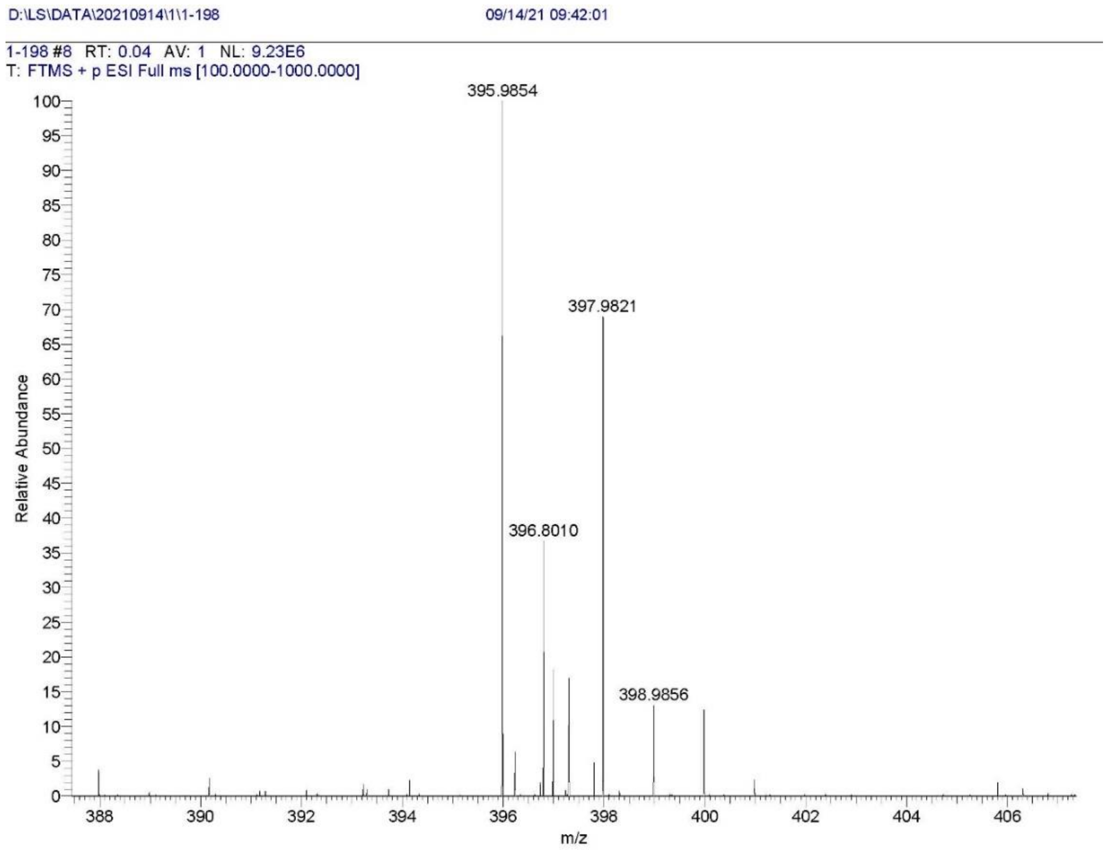

Figure S97. **The HRMS of compound 2bh.**

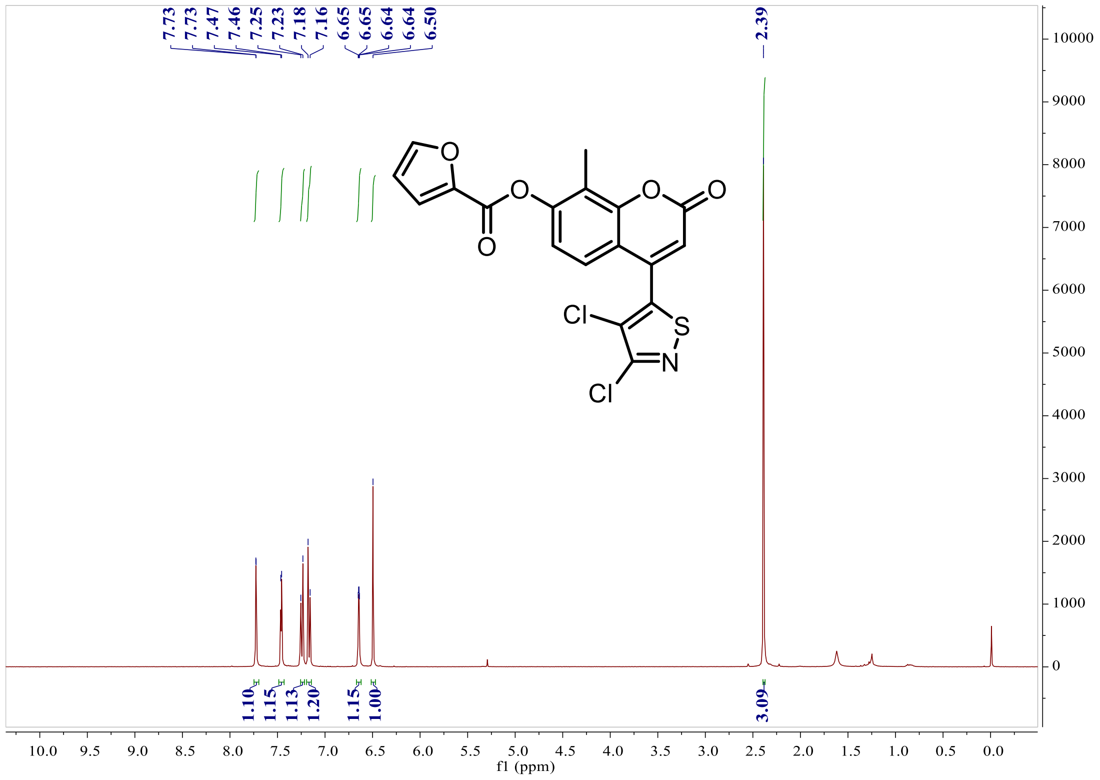

Figure S98. **The ^{1}H NMR (400MHz, Chloroform-*d*) of compound 2bi.**

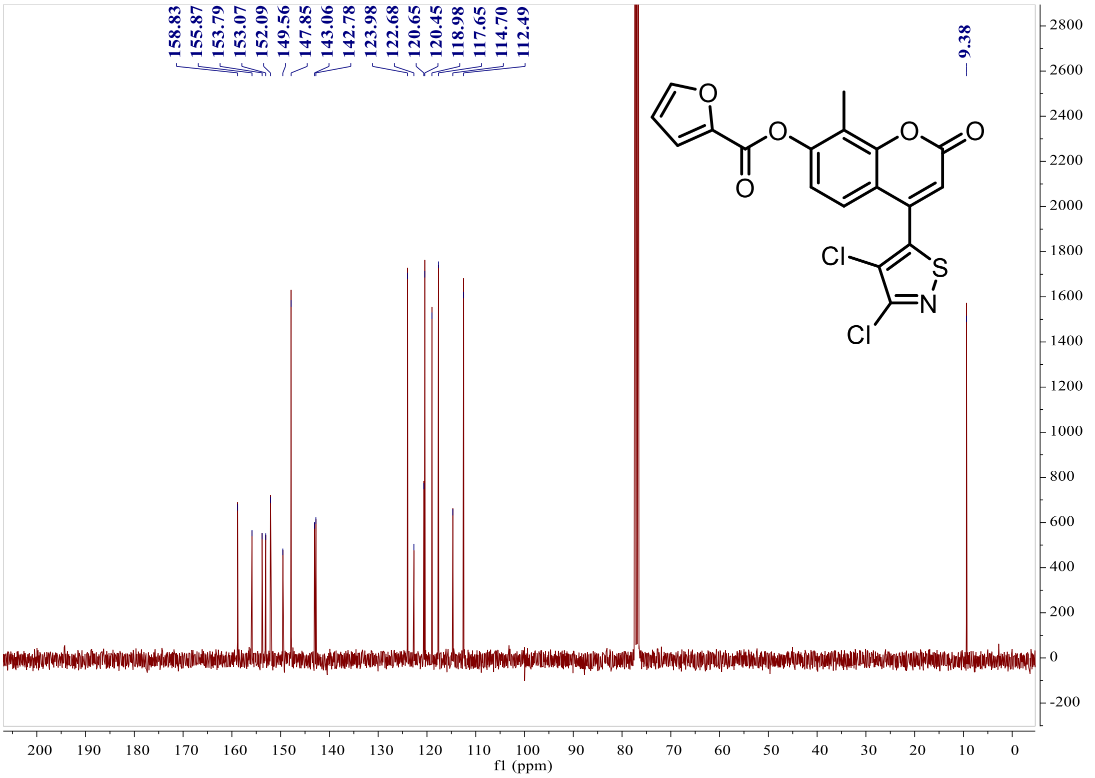

Figure S99. **The ^{13}C NMR (101MHz, Chloroform-*d*) of compound 2bi.**

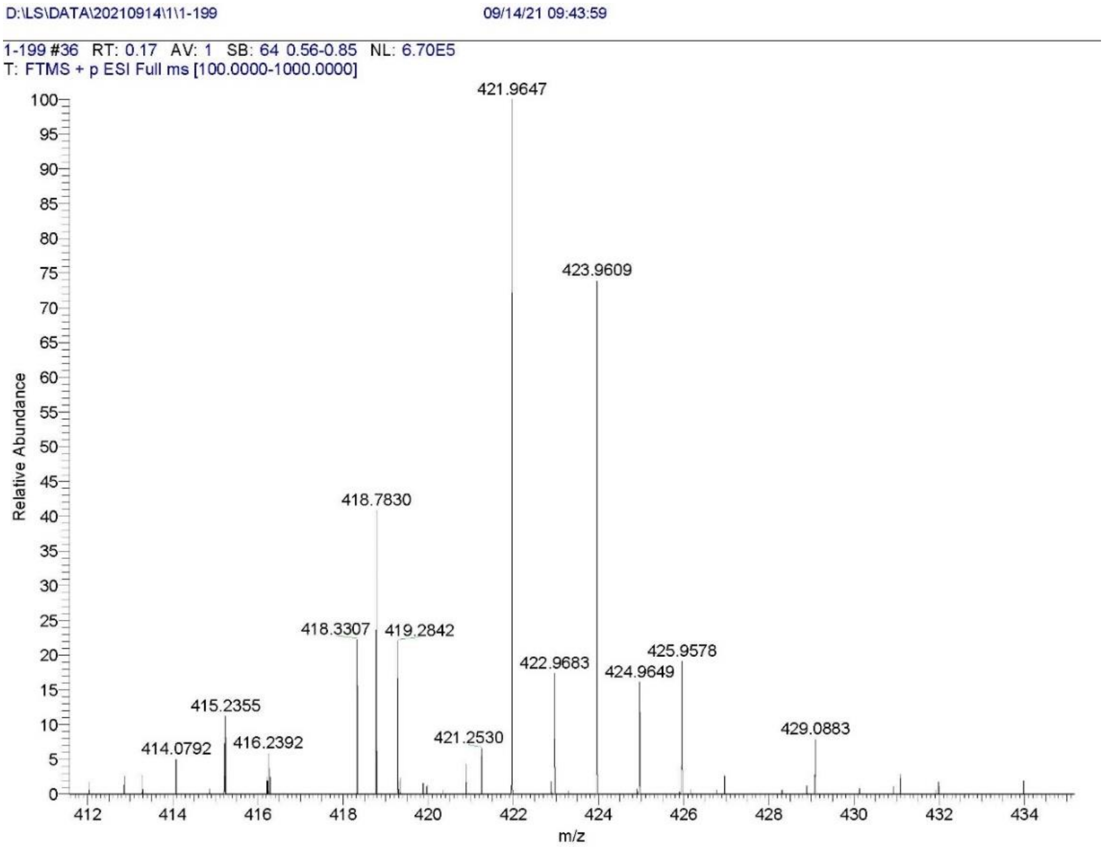

Figure S100. **The HRMS of compound 2bi.**

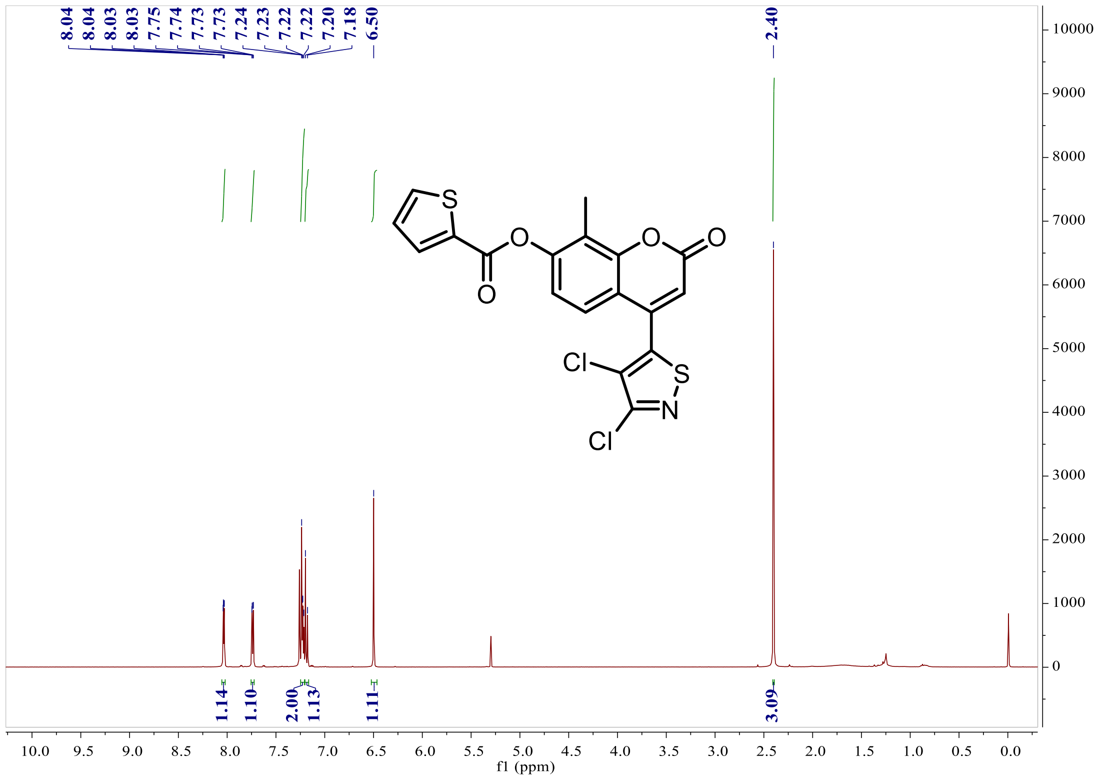

Figure S101. **The ^{1}H NMR (400MHz, Chloroform-*d*) of compound 2bj.**

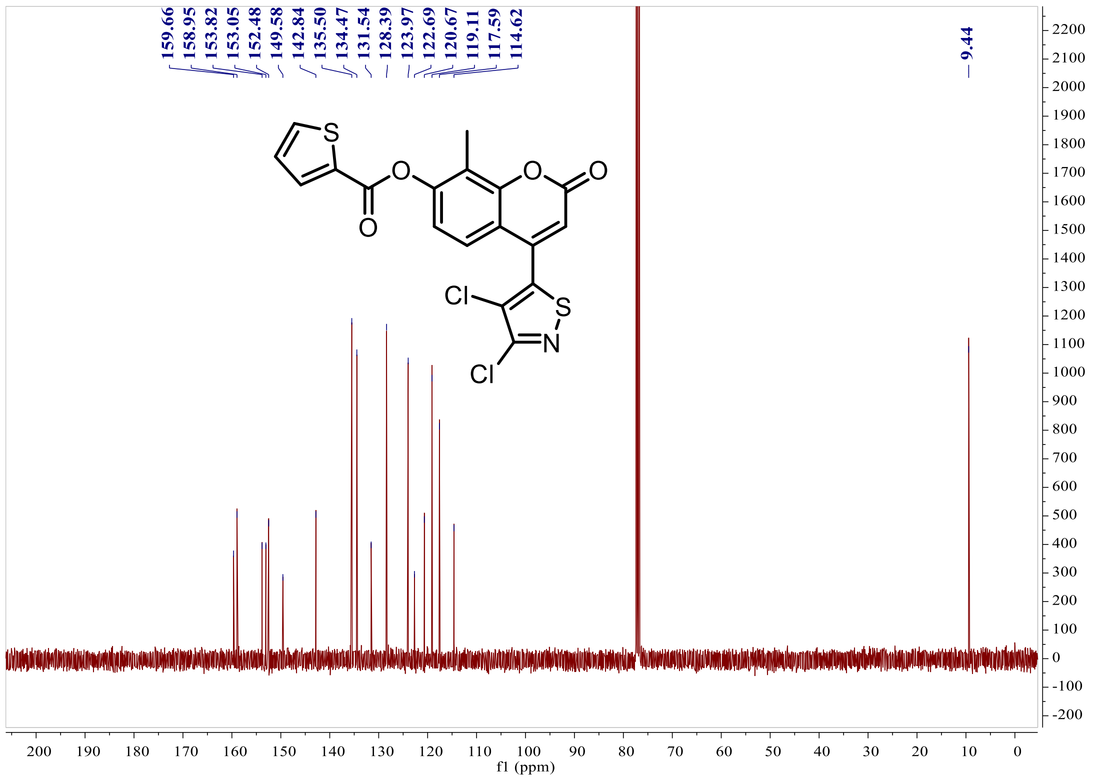

Figure S102. **The ^{13}C NMR (101MHz, Chloroform-*d*) of compound 2bj.**

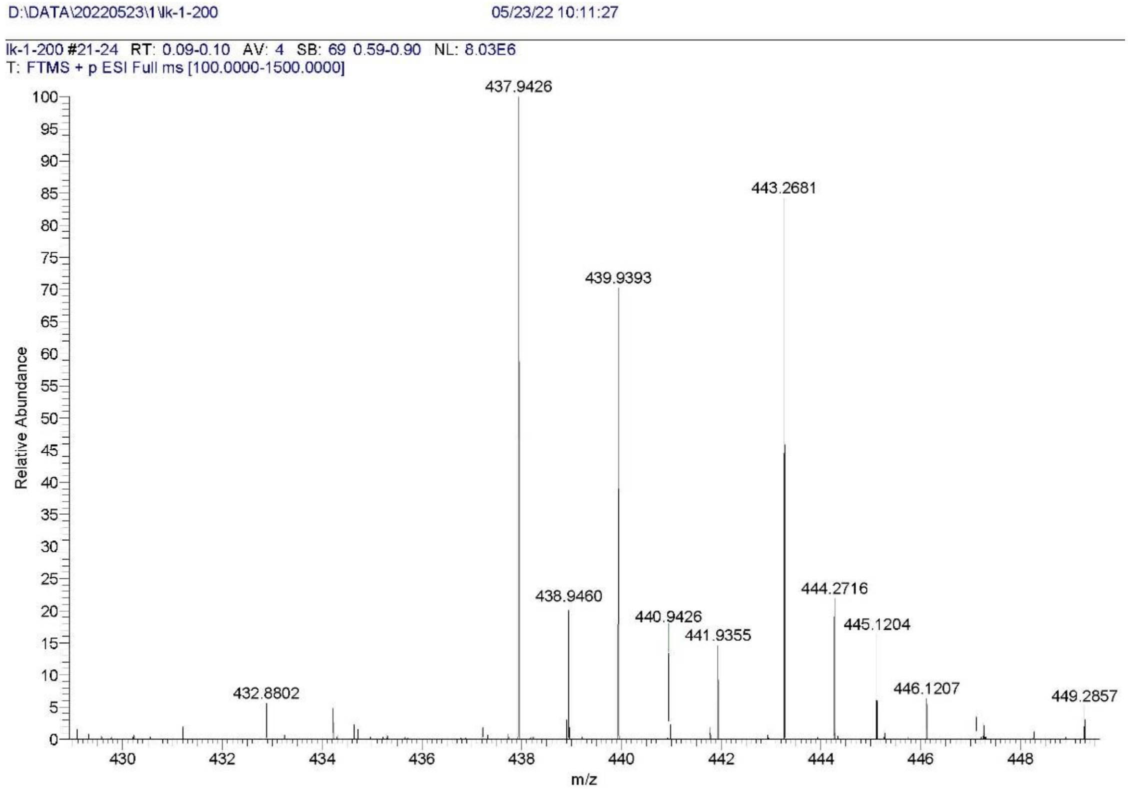

Figure S103. **The HRMS of compound 2bj.**

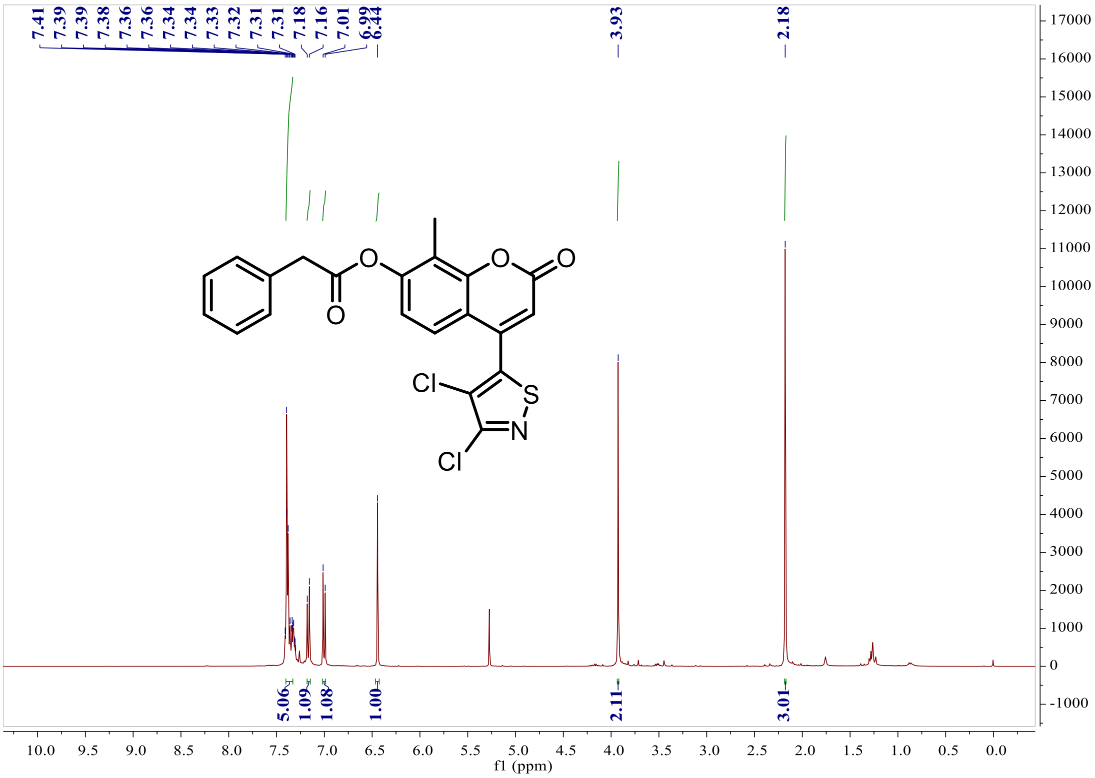

Figure S104. **The ^{1}H NMR (400MHz, Chloroform-*d*) of compound 2bk.**

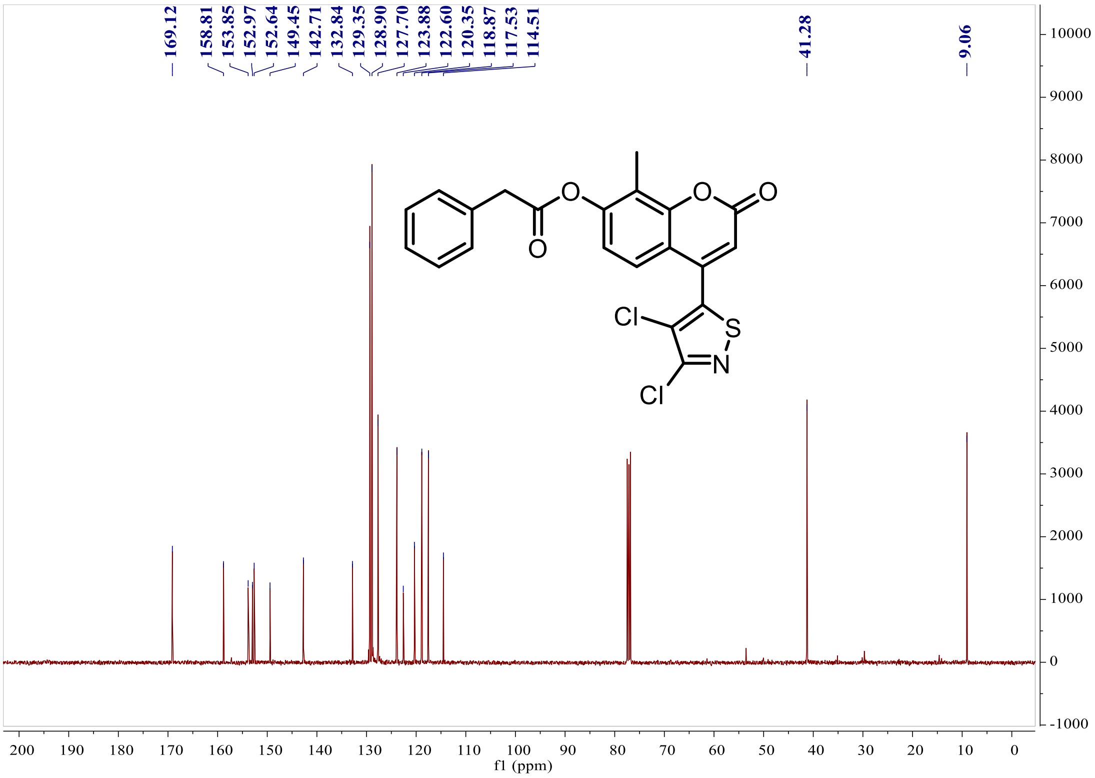

Figure S105. **The ^{13}C NMR (101MHz, Chloroform-*d*) of compound 2bk.**

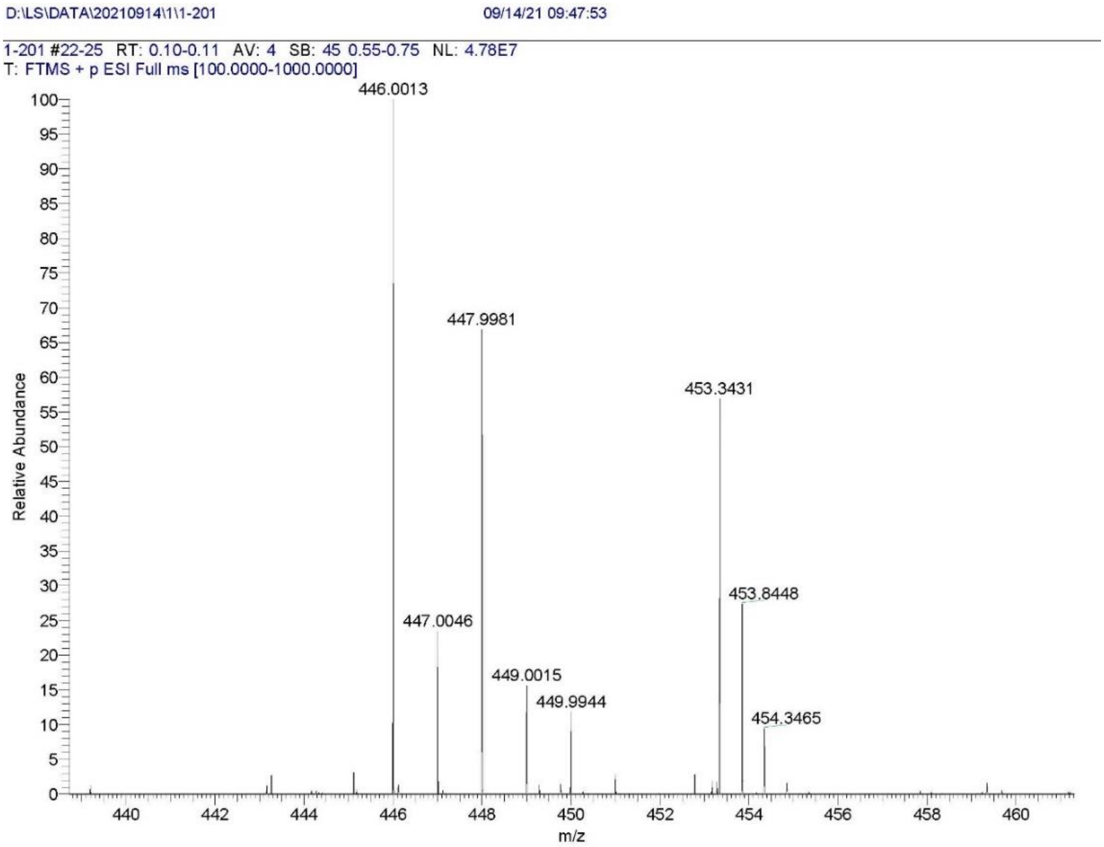

Figure S106. **The HRMS of compound 2bk.**

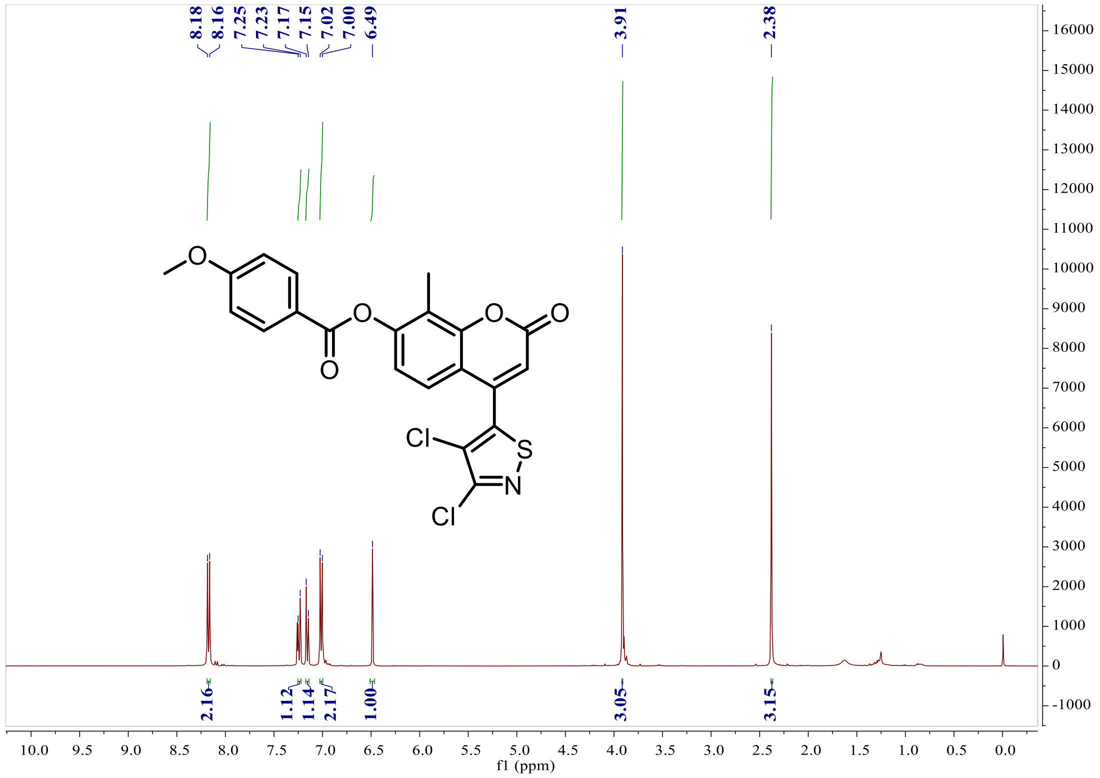

Figure S107. **The ^{1}H NMR (400MHz, Chloroform-*d*) of compound 2bl.**

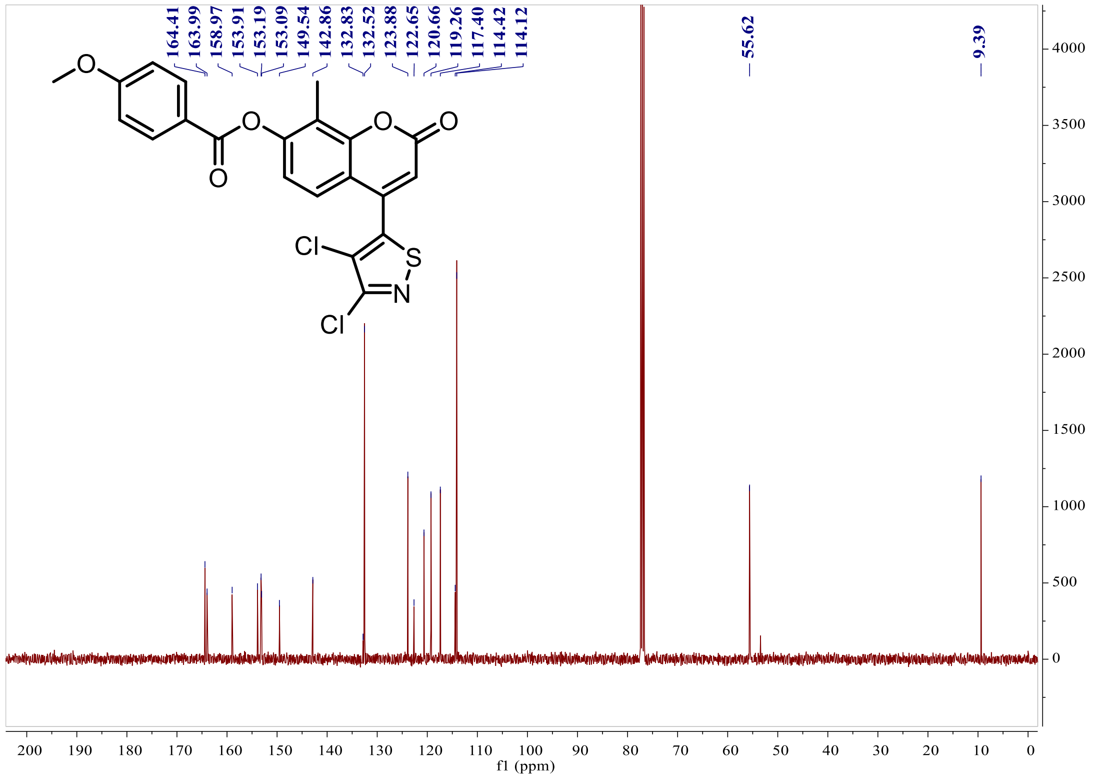

Figure S108. **The ^{13}C NMR (101MHz, Chloroform-*d*) of compound 2bl.**

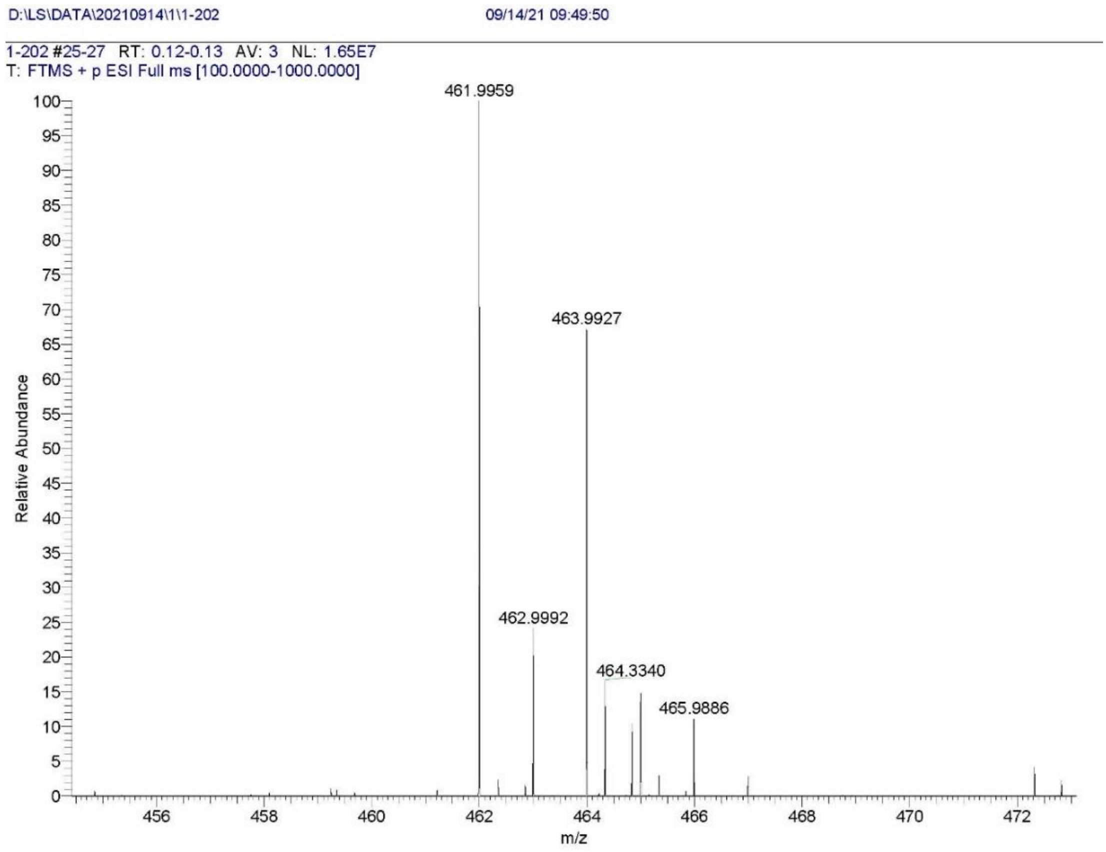

Figure S109. **The HRMS of compound 2bl.**

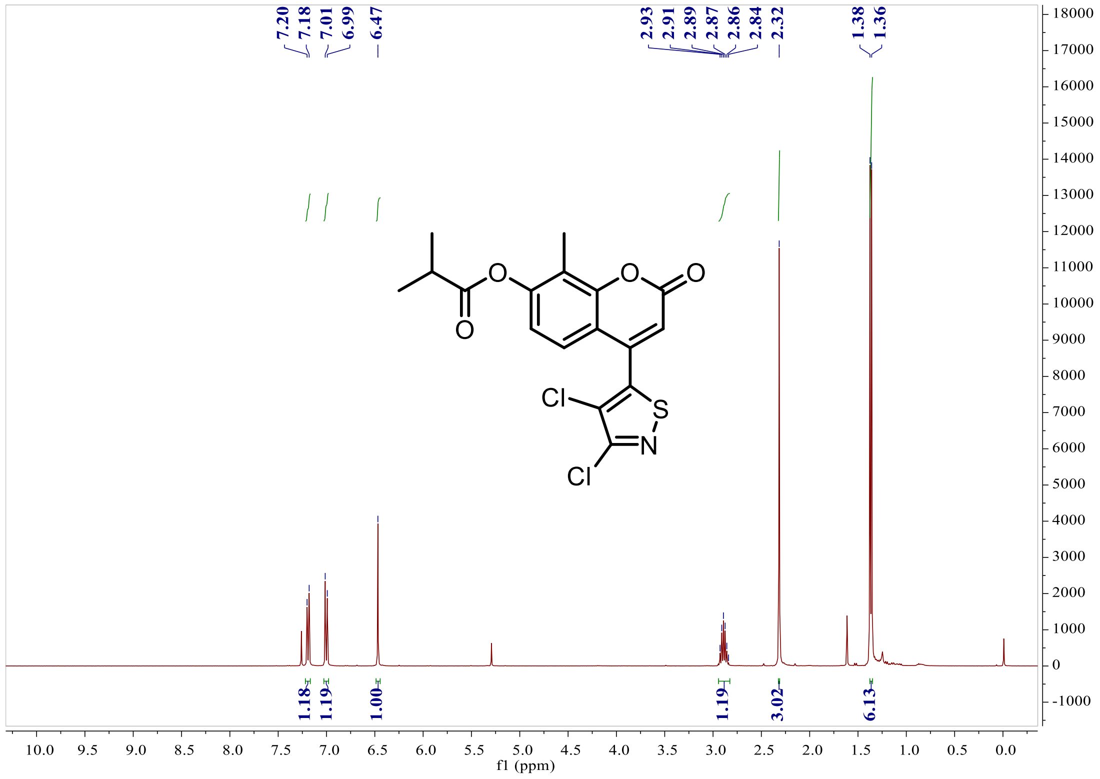

Figure S110. **The ^{1}H NMR (400MHz, Chloroform-*d*) of compound 2bm.**

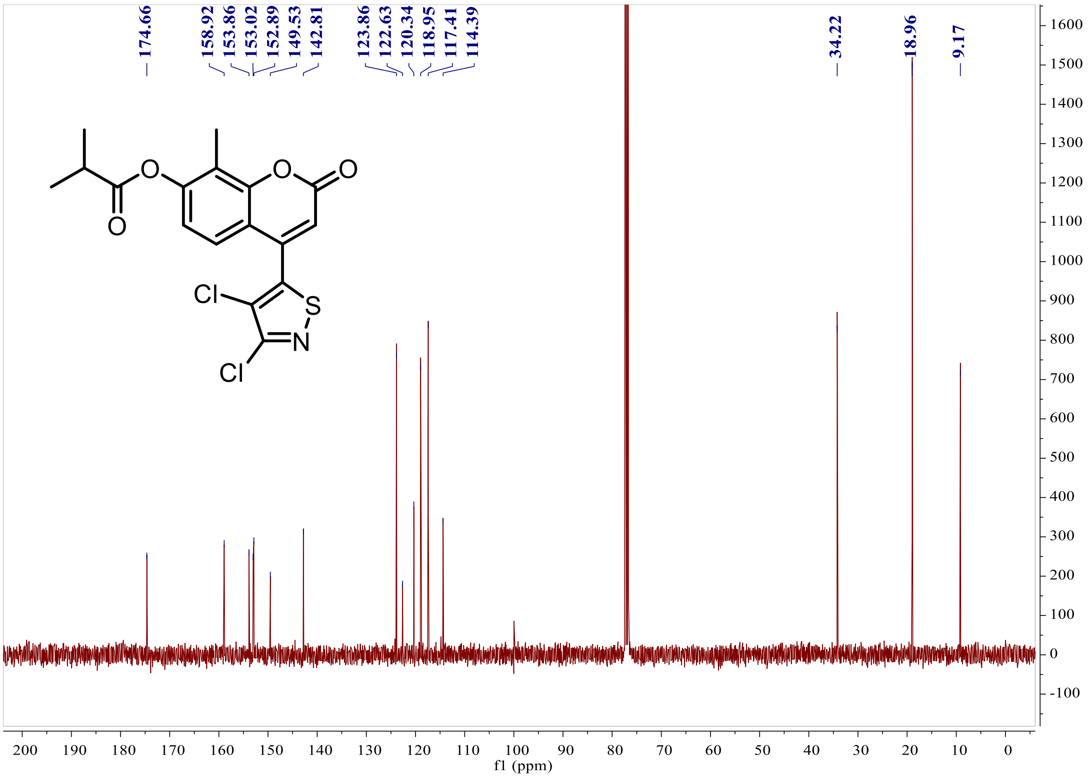

Figure S111. **The ^{13}C NMR (101MHz, Chloroform-*d*) of compound 2bm.**

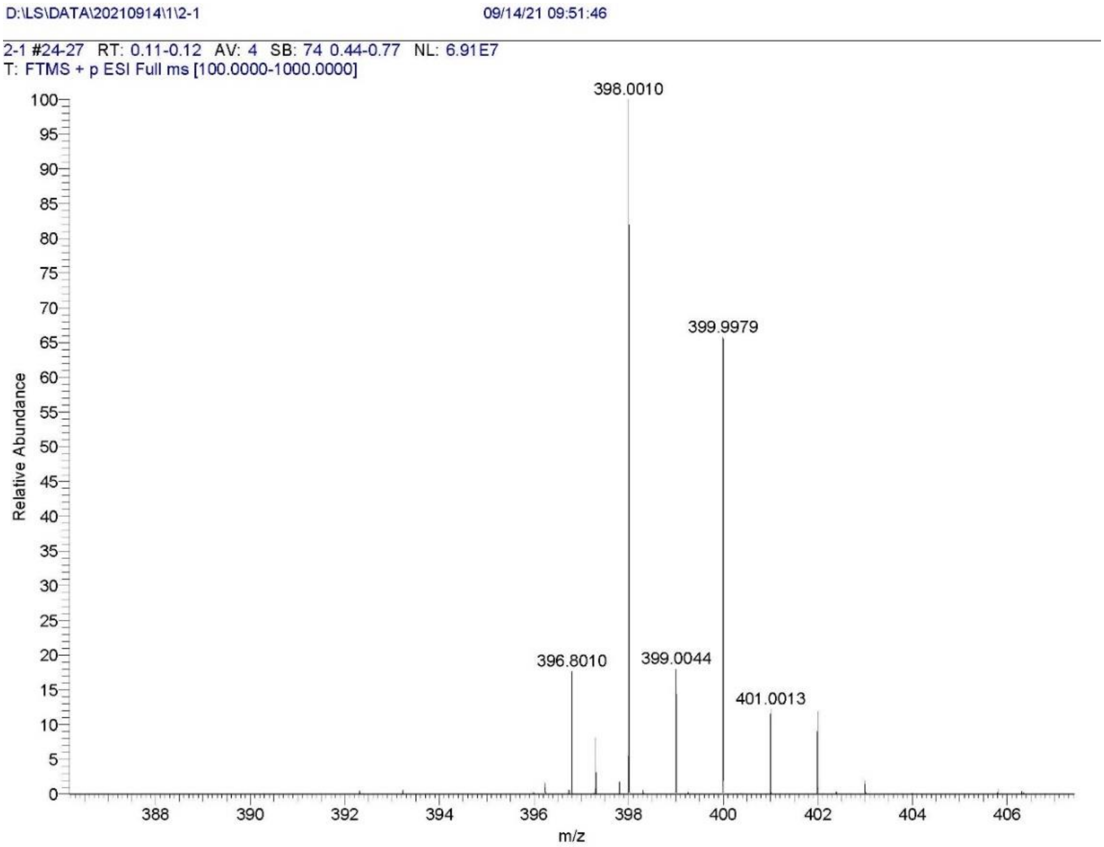

Figure S112. **The HRMS of compound 2bm.**

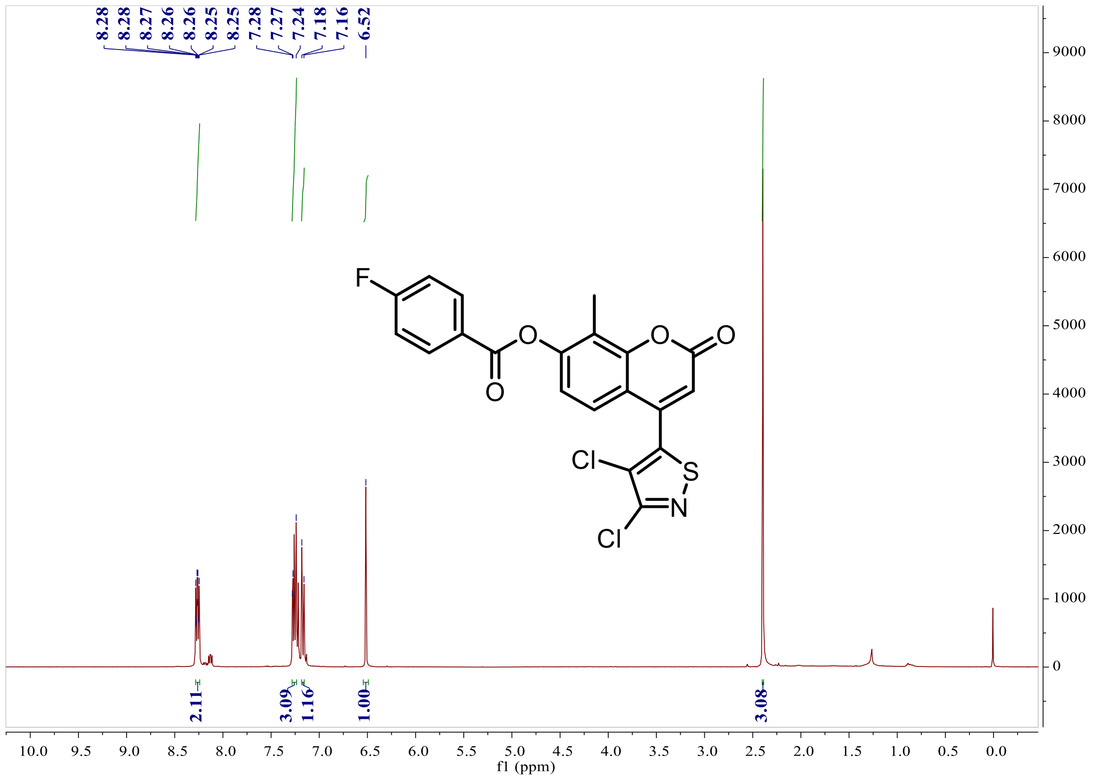

Figure S113. **The ^{1}H NMR (400MHz, Chloroform-*d*) of compound 2bn.**

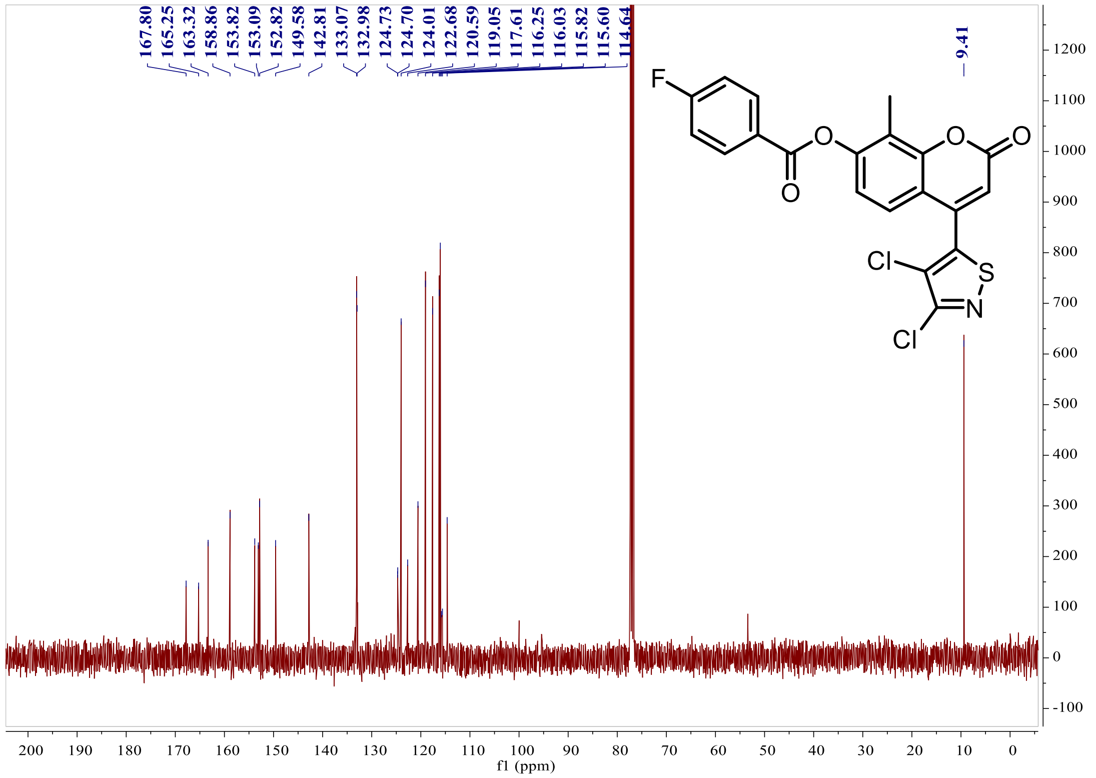

Figure S114. **The ^{13}C NMR (101MHz, Chloroform-*d*) of compound 2bn.**

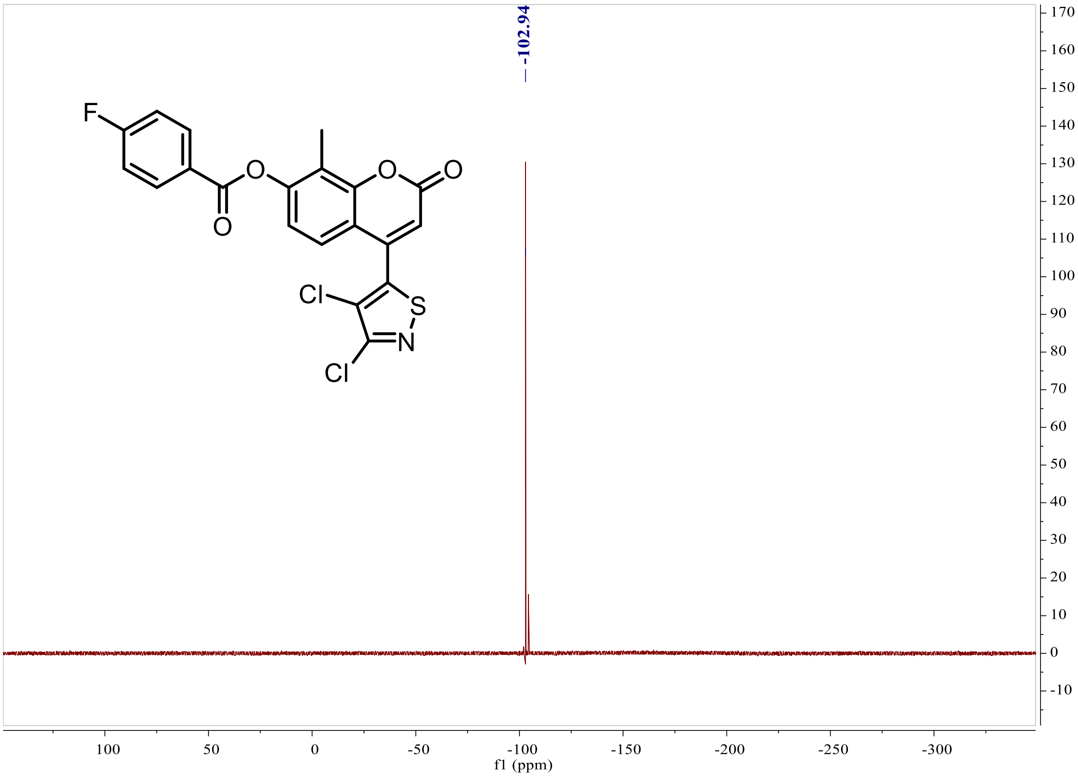

Figure S115. **The ^{19}F NMR (376MHz, Chloroform-*d*) of compound 2bn.**

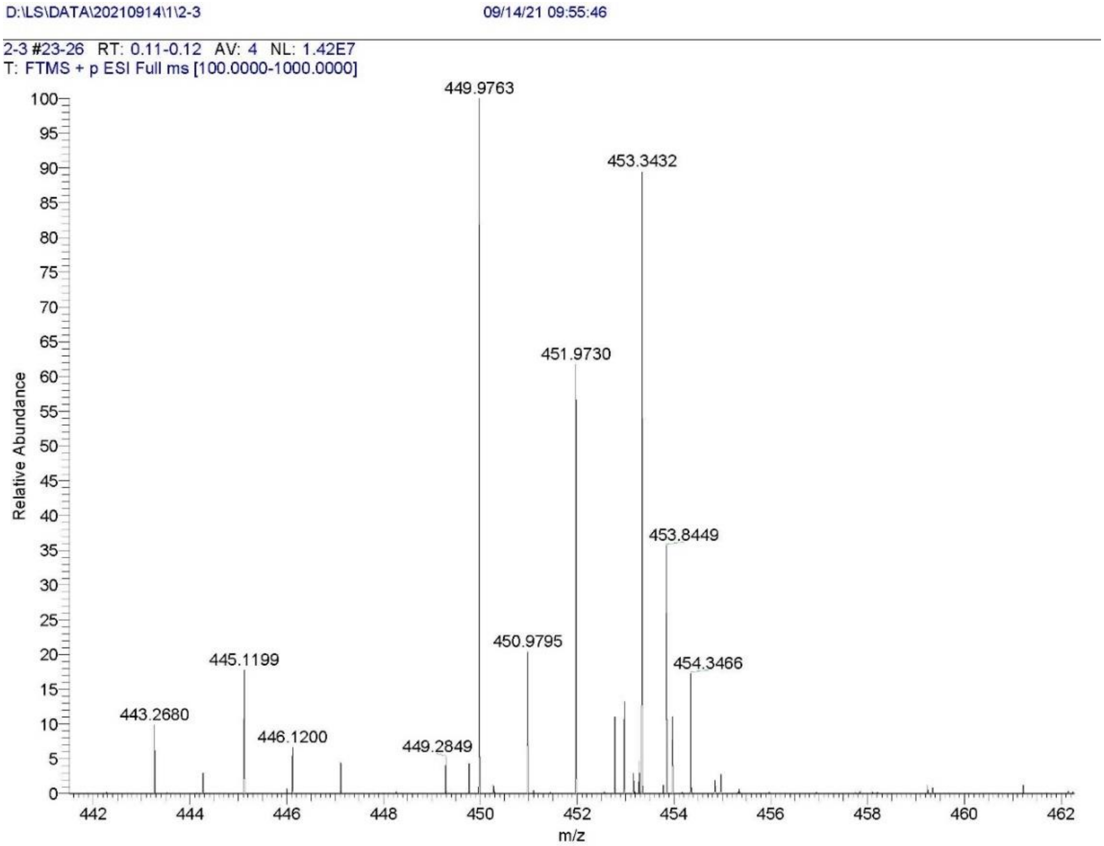

Figure S116. **The HRMS of compound 2bn.**

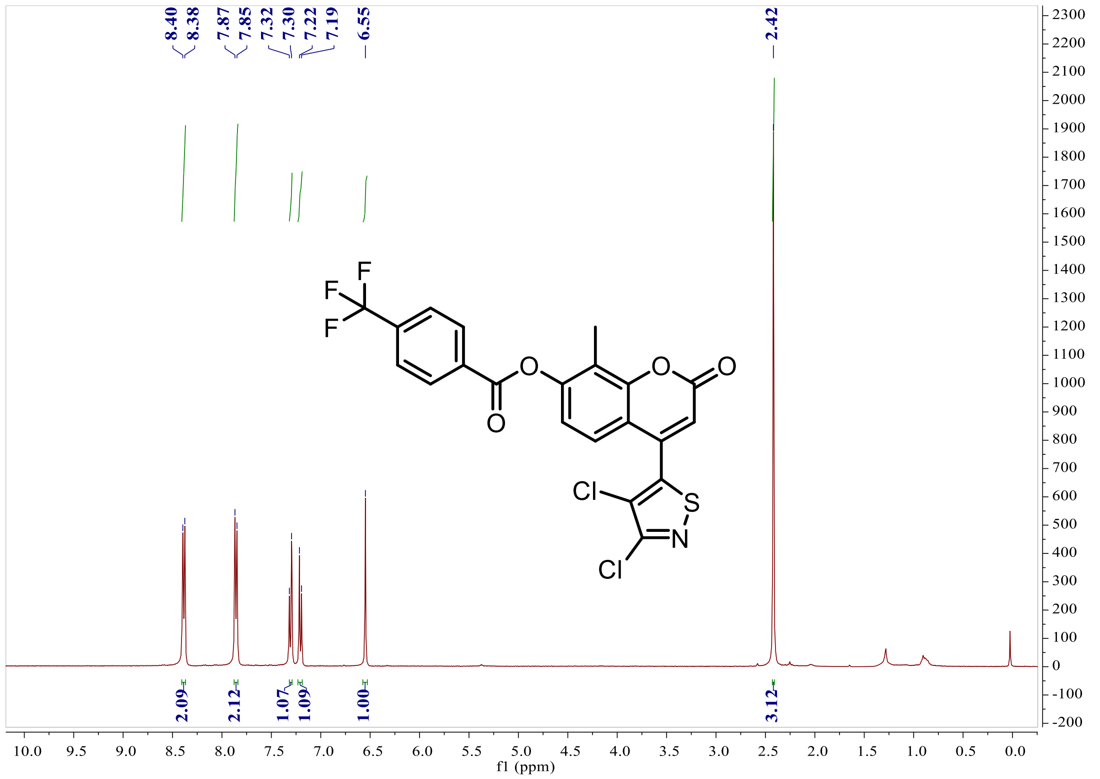

***Figure S117*. The ^{1}H NMR (400MHz, Chloroform-*d*) of compound 2bo.**

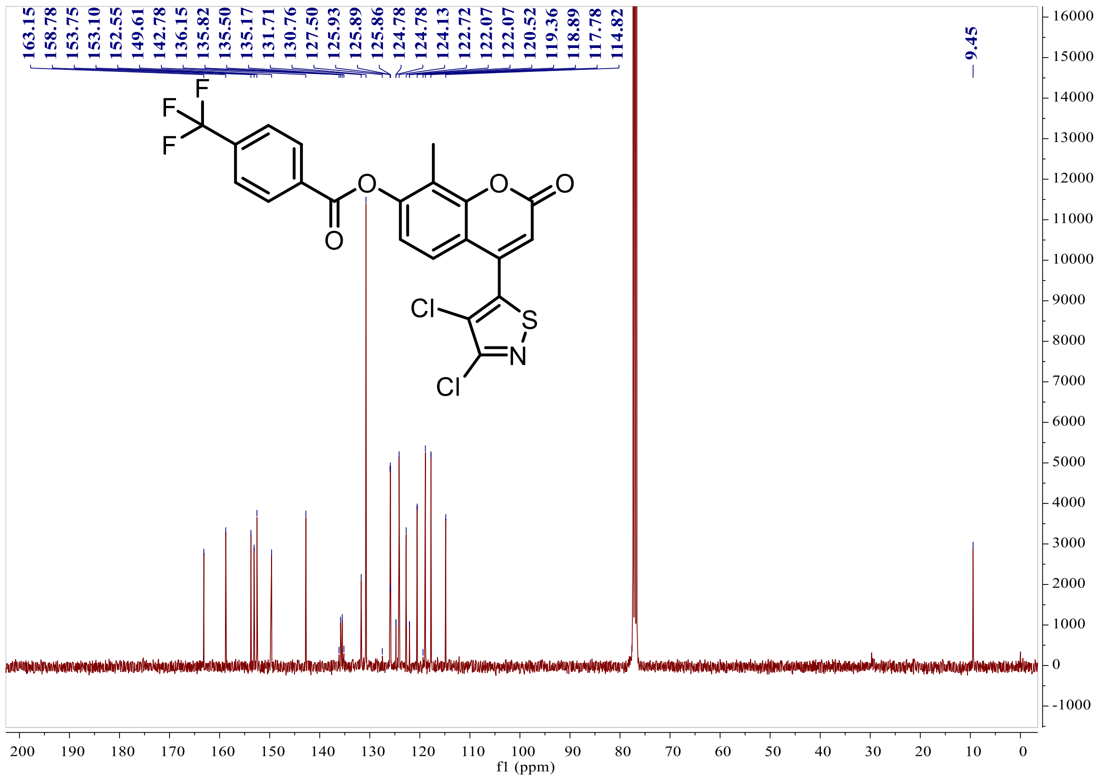

***Figure S118*. The ^{13}C NMR (101MHz, Chloroform-*d*) of compound 2bo.**

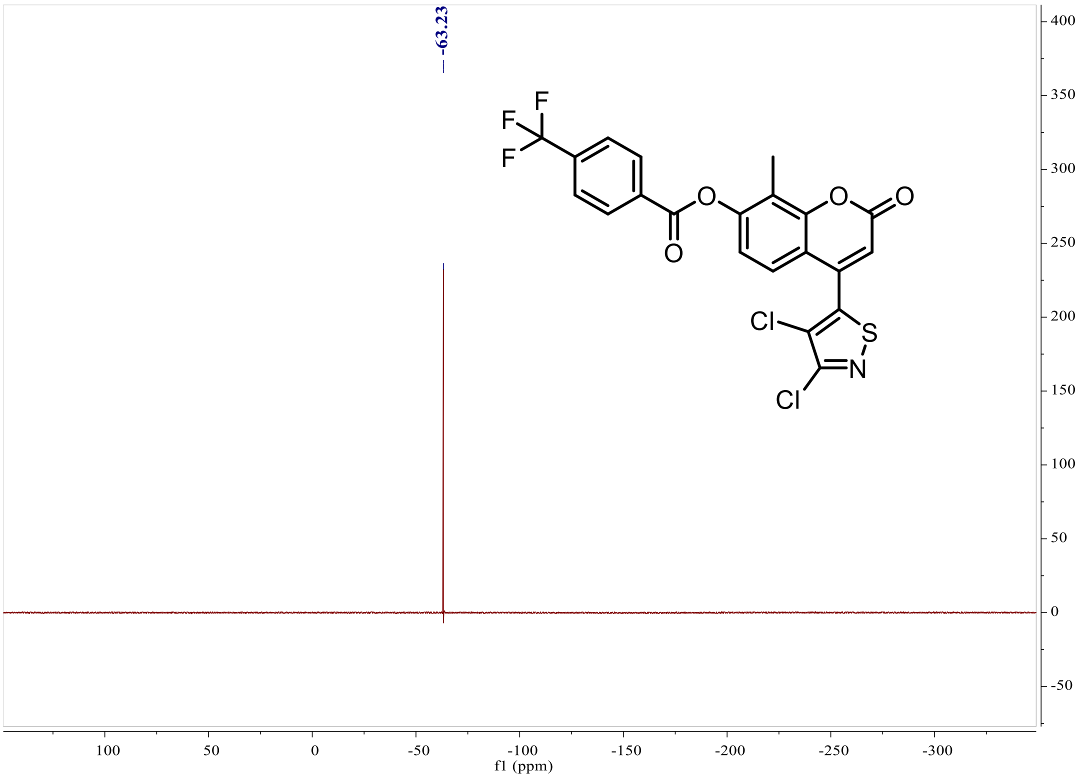

Figure S119. **The ^{19}F NMR (376MHz, Chloroform-*d*) of compound 2bo.**

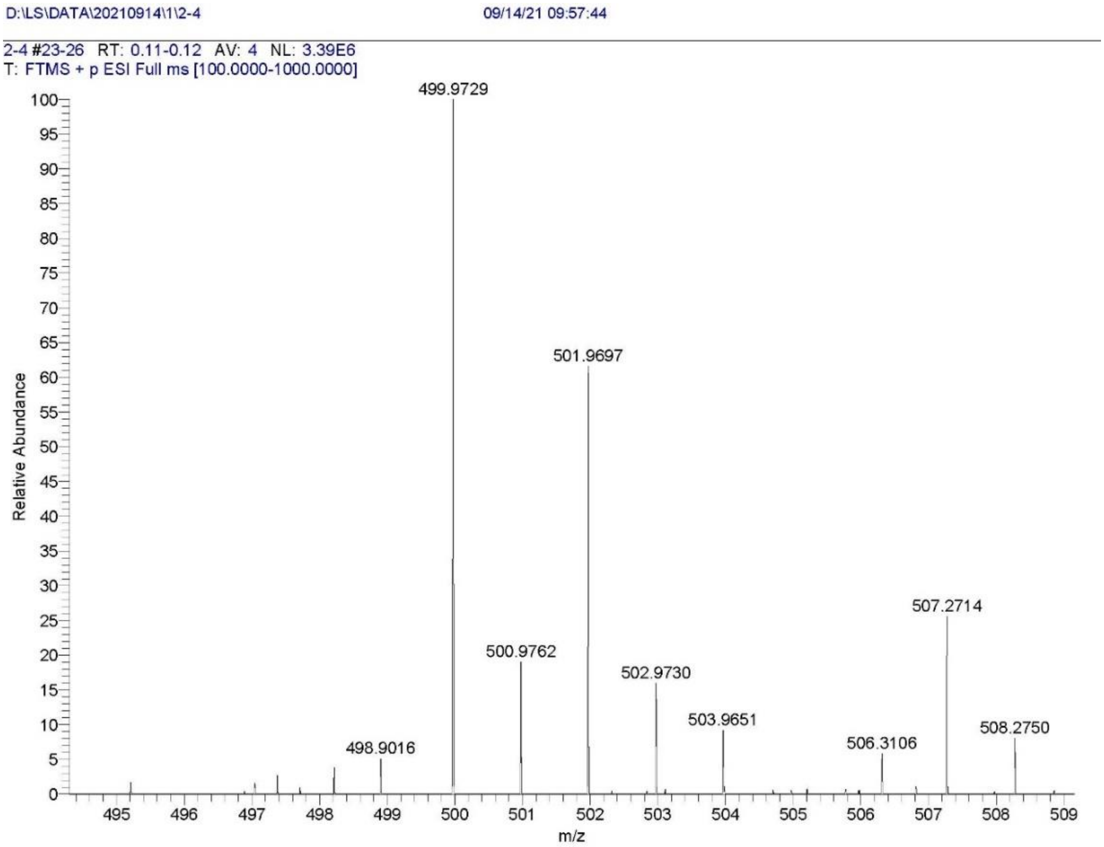

Figure S120. **The HRMS of compound 2bo.**

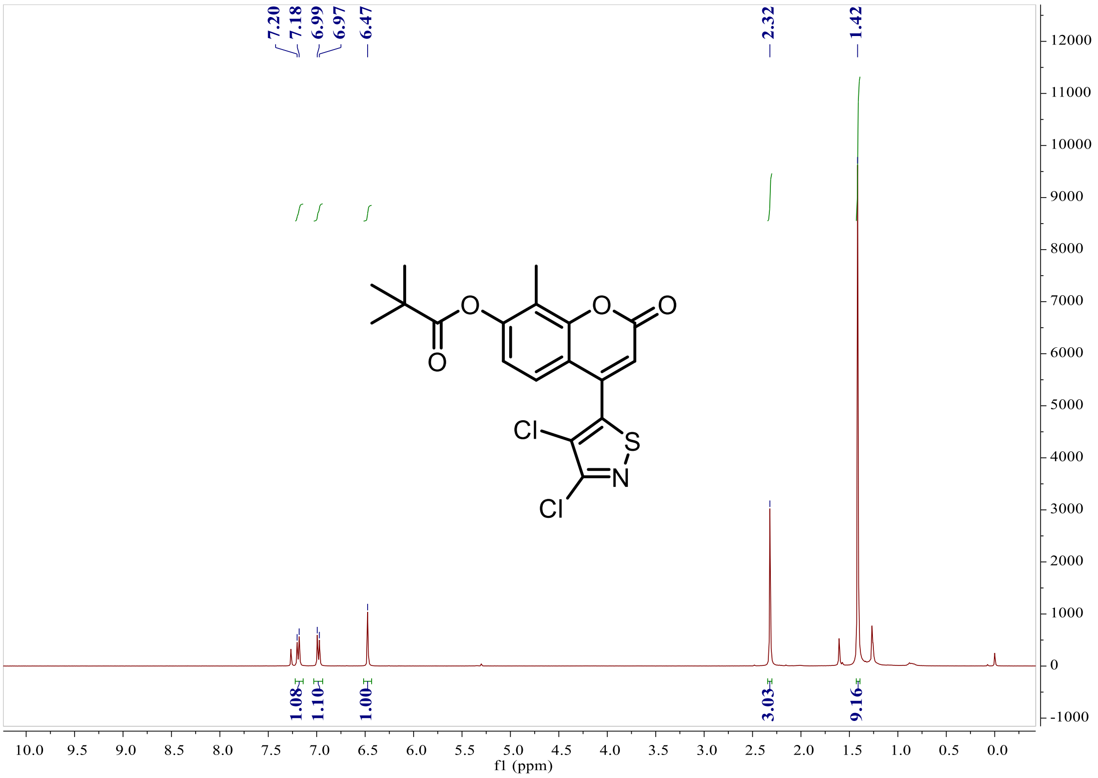

Figure S121. **The ^{1}H NMR (400MHz, Chloroform-*d*) of compound 2bp.**

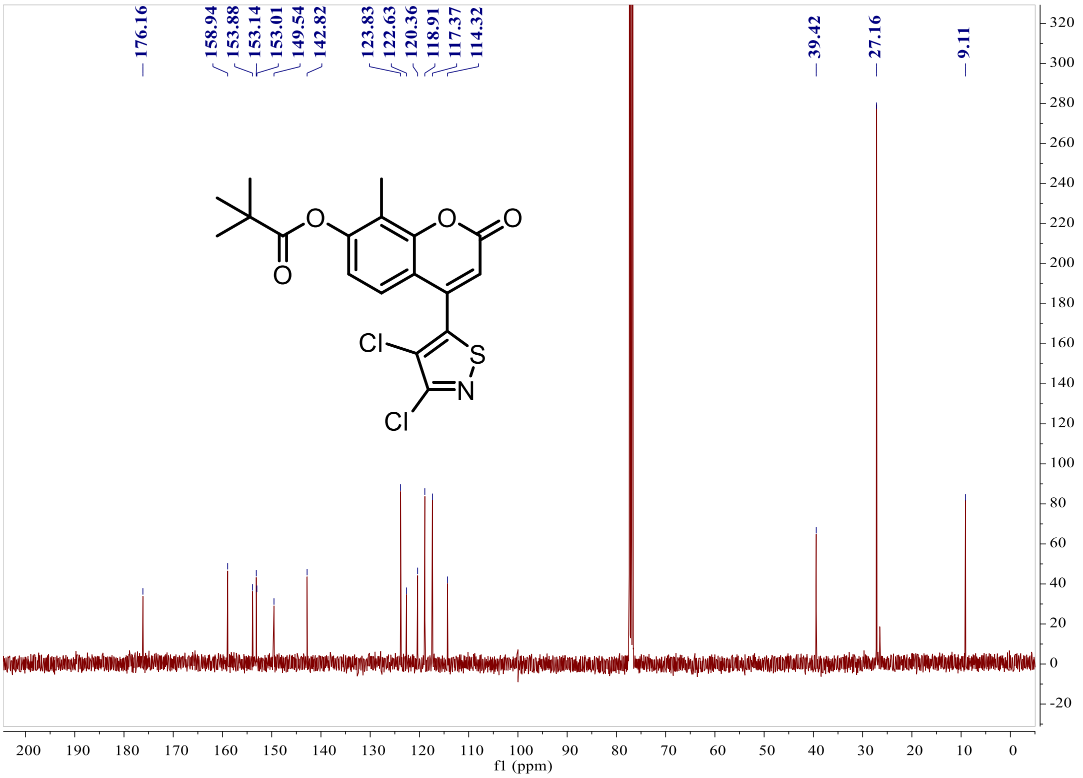

Figure S122. **The ^{13}C NMR (101MHz, Chloroform-*d*) of compound 2bp.**

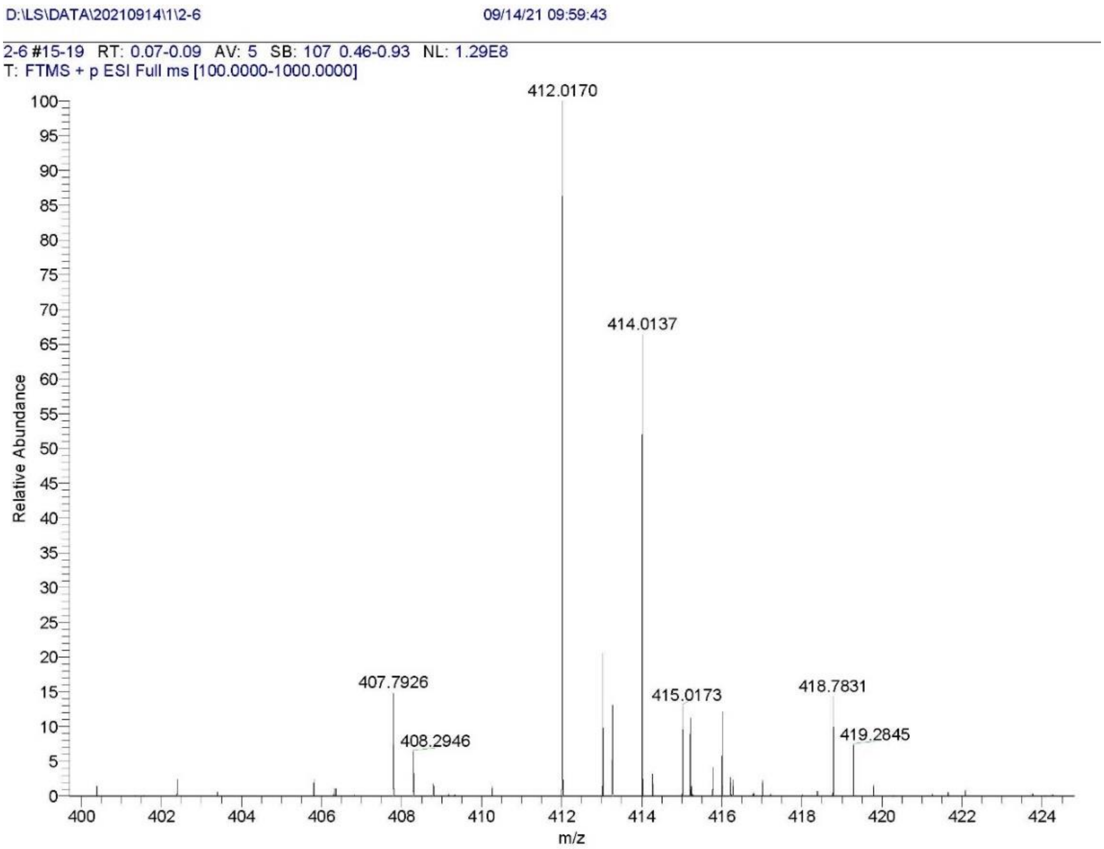

Figure S123. **The HRMS of compound 2bp.**

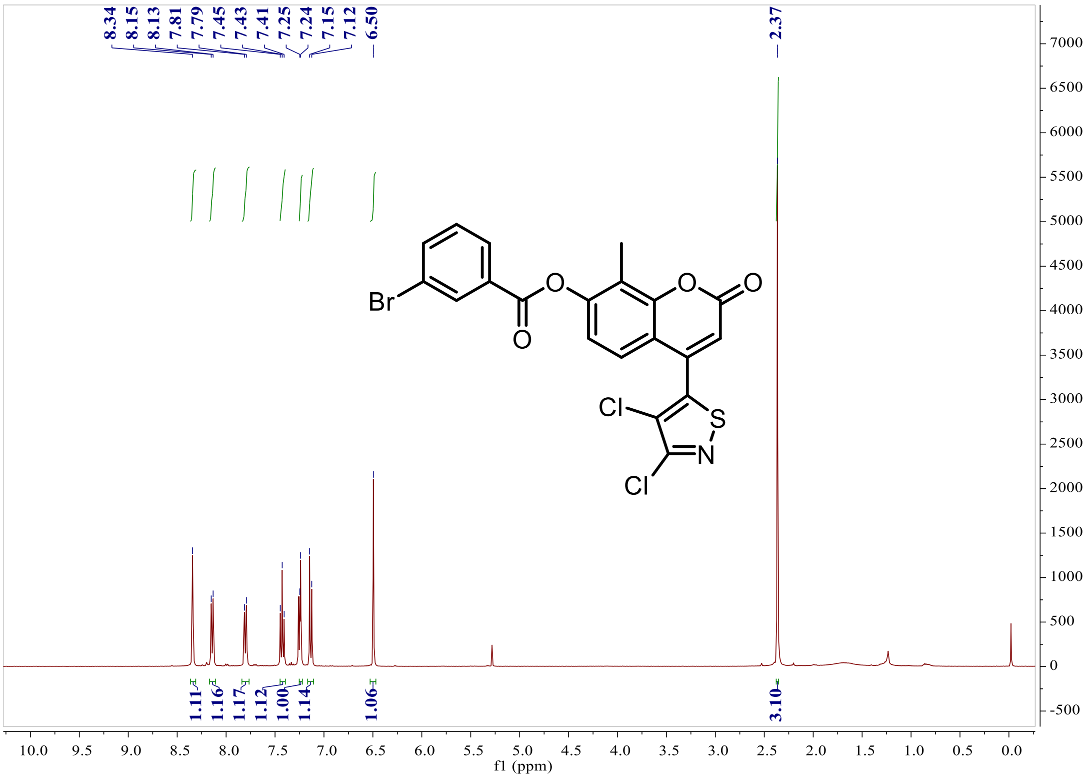

Figure S124. **The ^{1}H NMR (400MHz, Chloroform-*d*) of compound 2bq.**

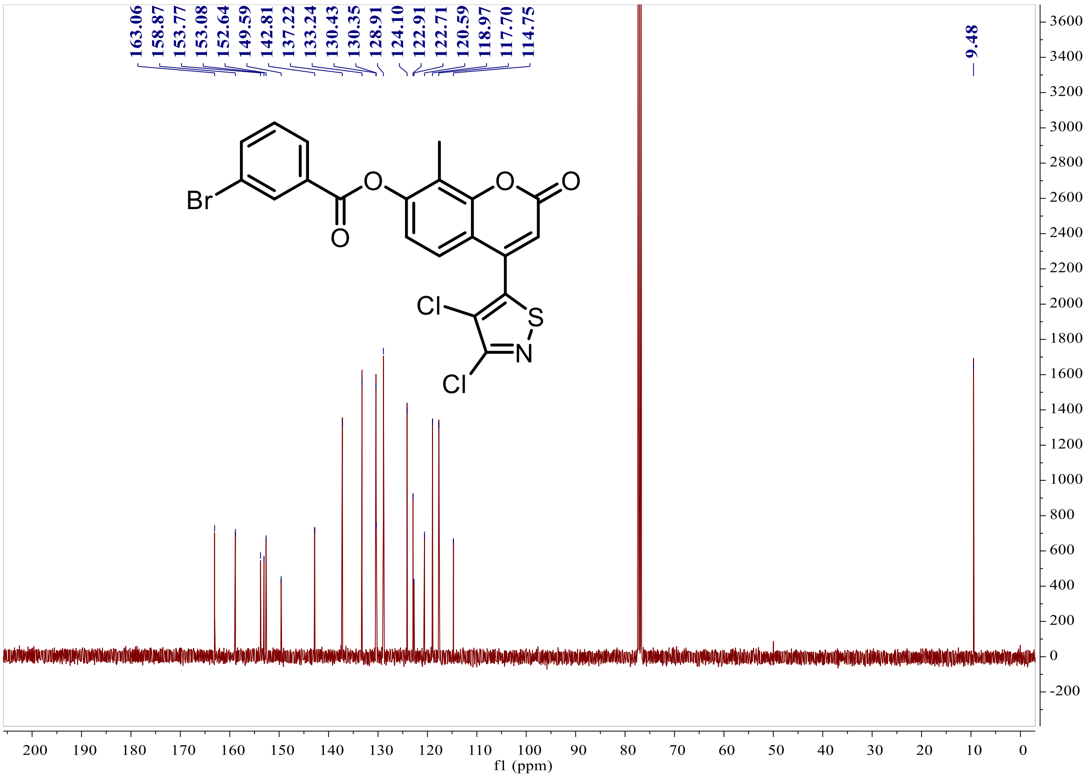

Figure S125. **The ^{13}C NMR (101MHz, Chloroform-*d*) of compound 2bq.**

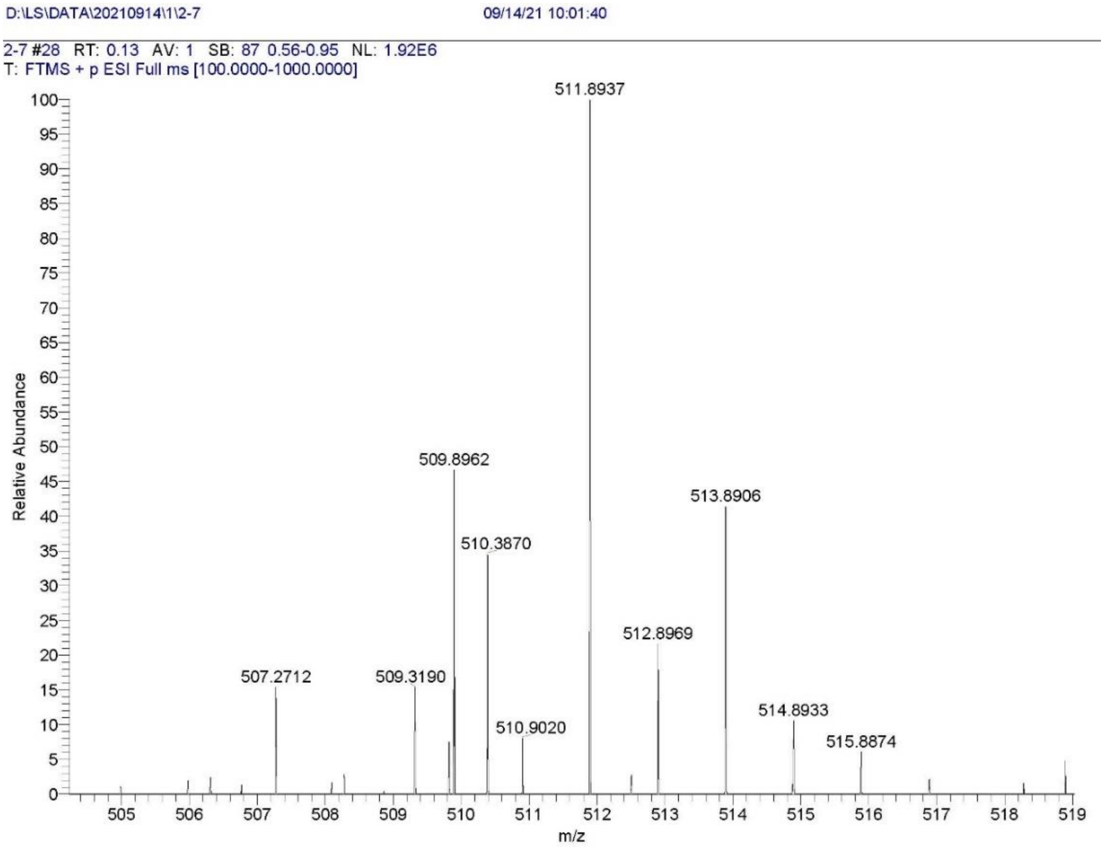

Figure S126. **The HRMS of compound 2bq.**

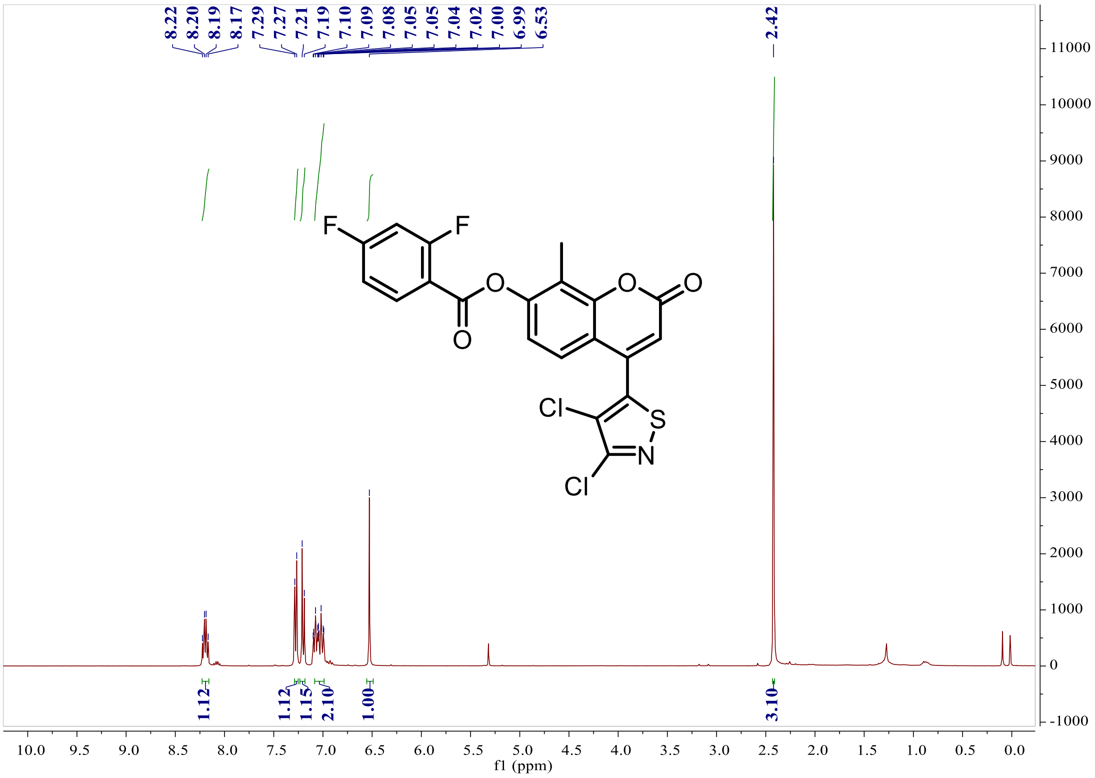

Figure S127. **The ^{1}H NMR (400MHz, Chloroform-*d*) of compound 2br.**

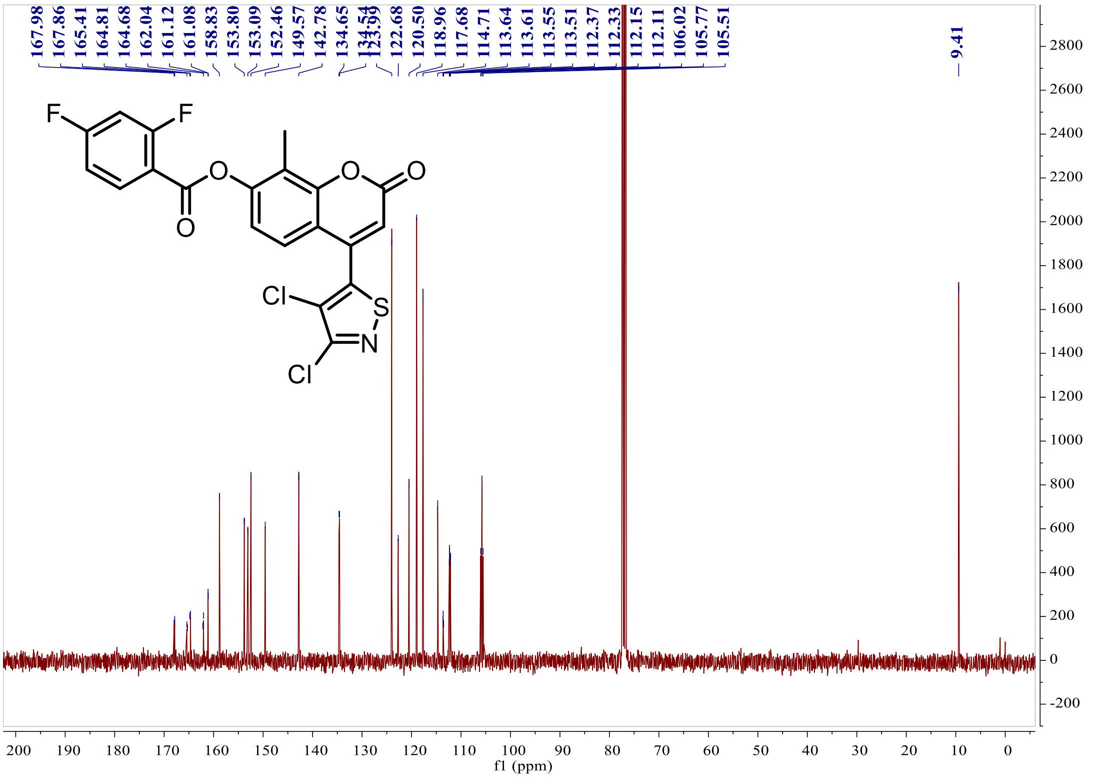

Figure S128. **The ^{13}C NMR (101MHz, Chloroform-*d*) of compound 2br.**

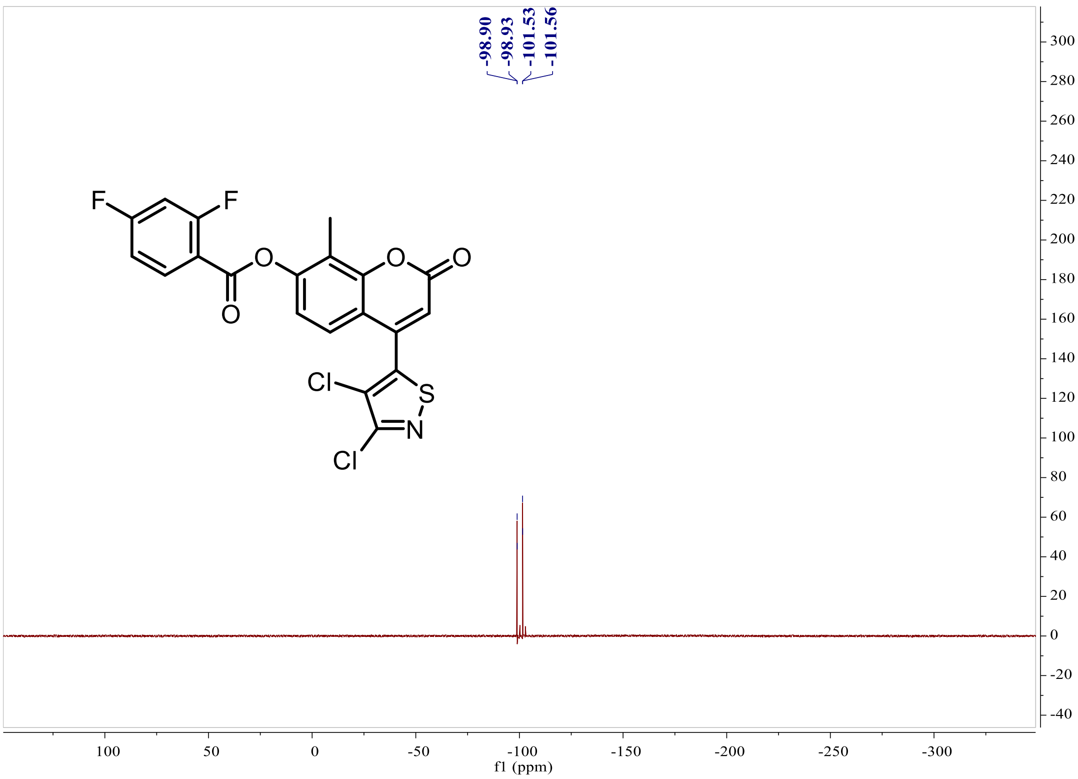

Figure S129. **The** **^{19}F NMR (376MHz, Chloroform-*d*) of compound 2br.**

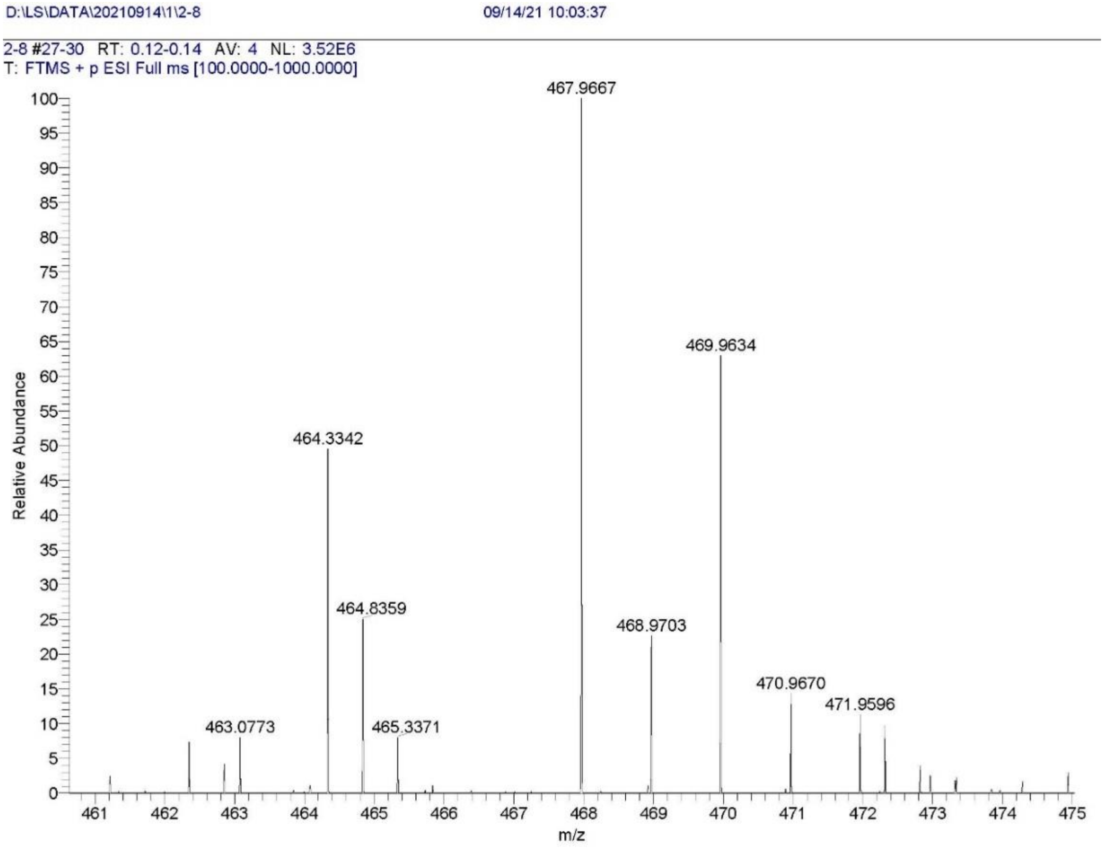

Figure S130. **The HRMS of compound 2br.**

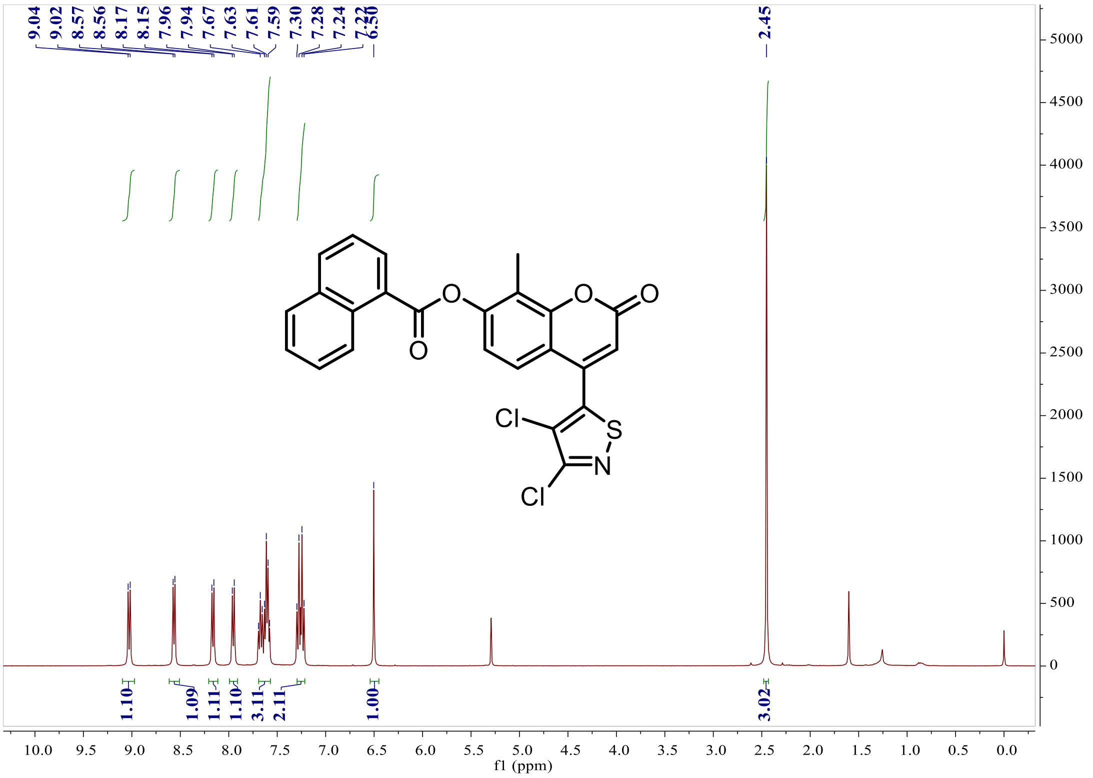

Figure S131. **The** ^{1}**H NMR (400MHz, Chloroform-*d*) of compound 2bs.**

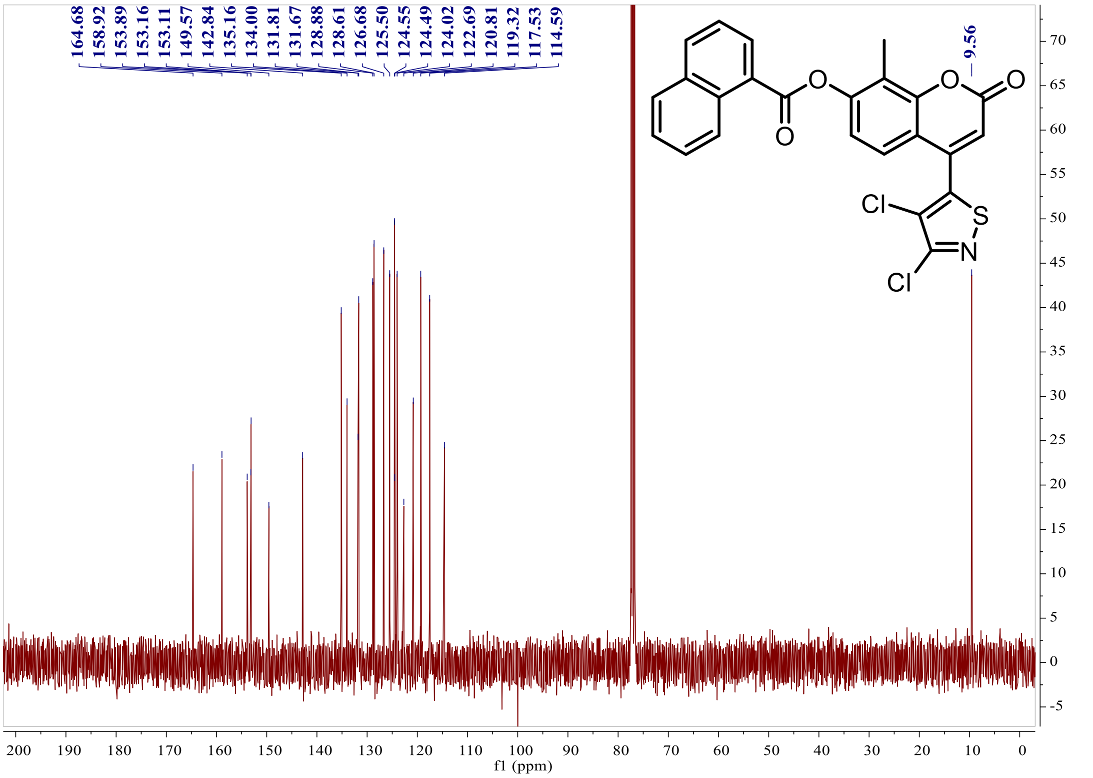

Figure S132. **The** ^{13}**C NMR (101MHz, Chloroform-*d*) of compound 2bs.**

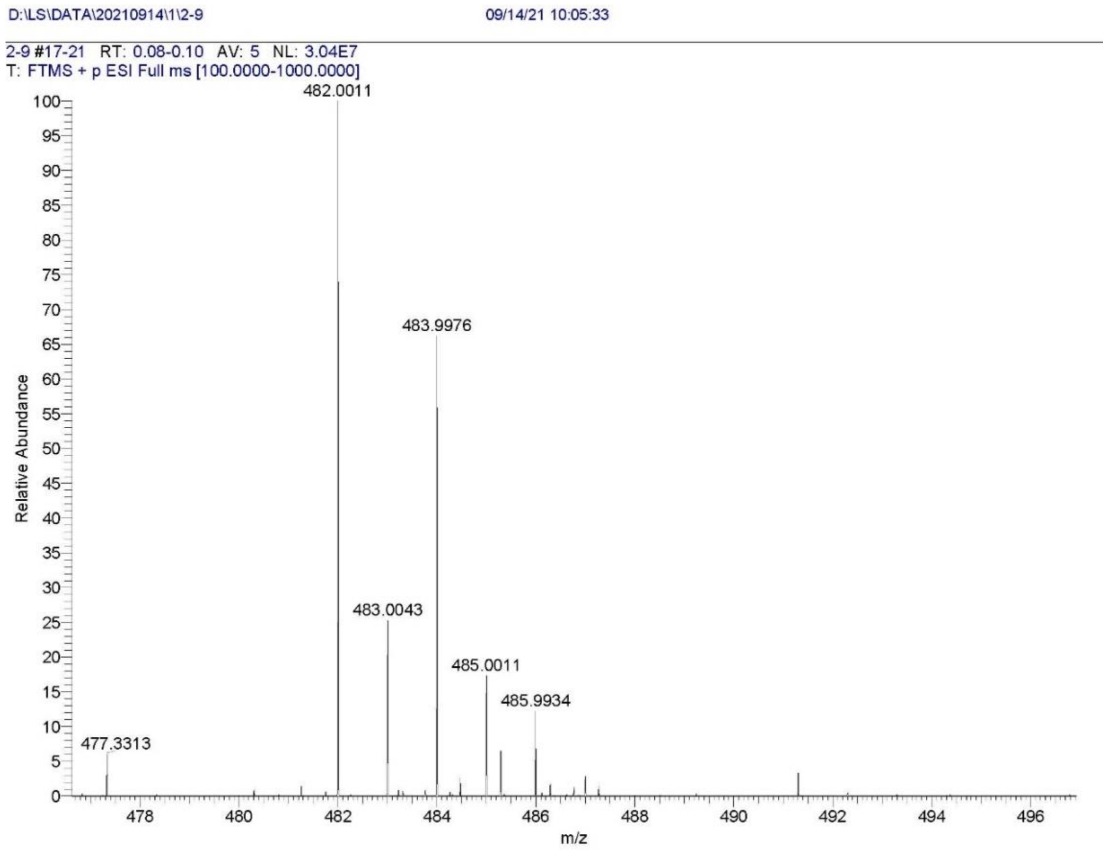

Figure S133. **The HRMS of compound 2bs.**

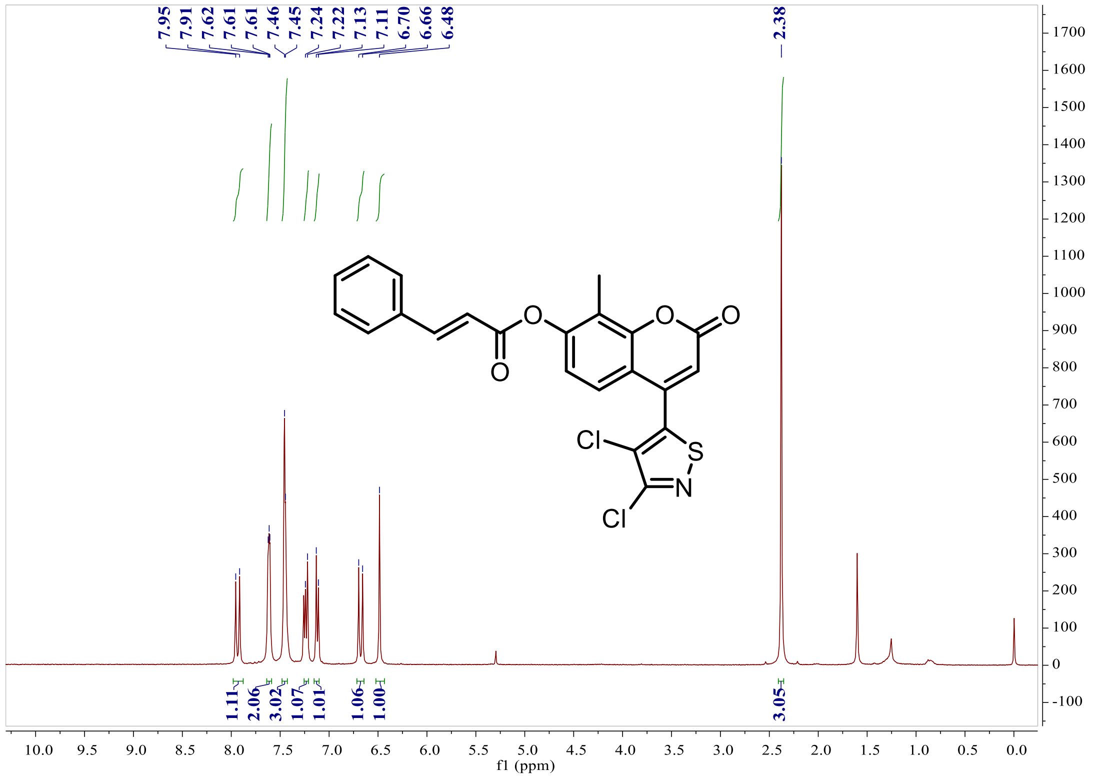

Figure S134. **The ^{1}H NMR (400MHz, Chloroform-*d*) of compound 2bt.**

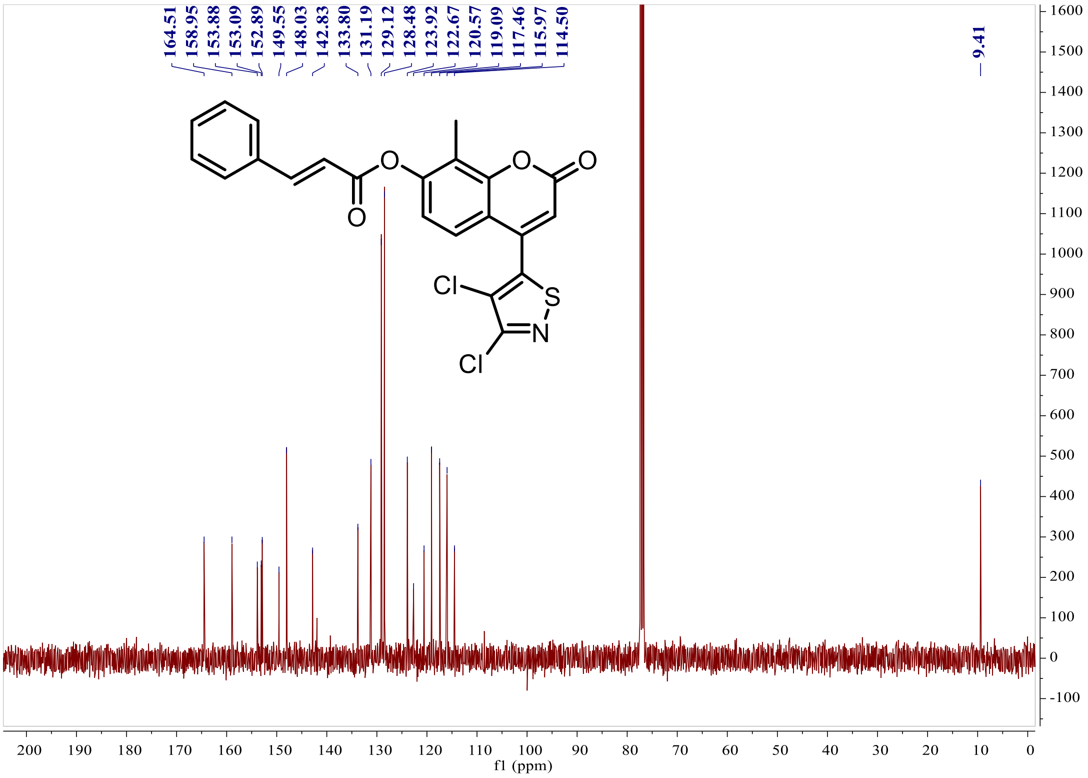

Figure S135. **The** ^{13}**C NMR (101MHz, Chloroform-*d*) of compound 2bt.**

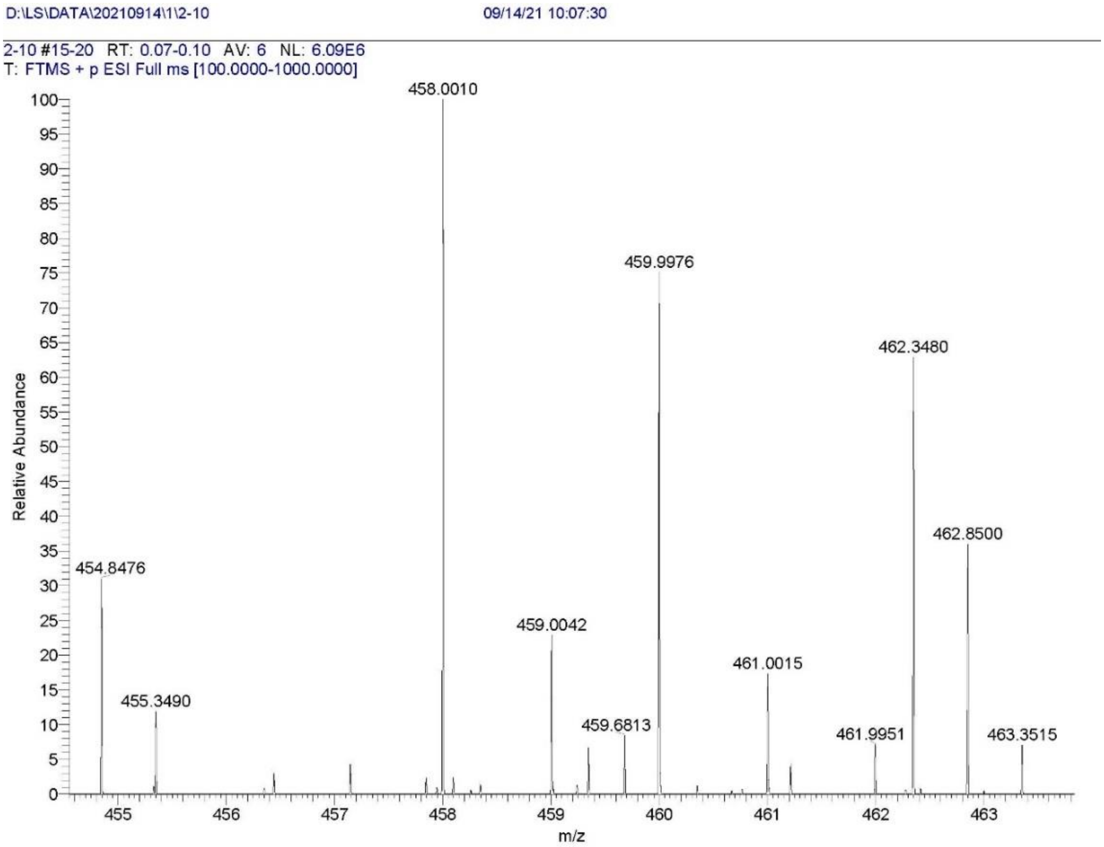

Figure S136. **The HRMS of compound 2bt.**

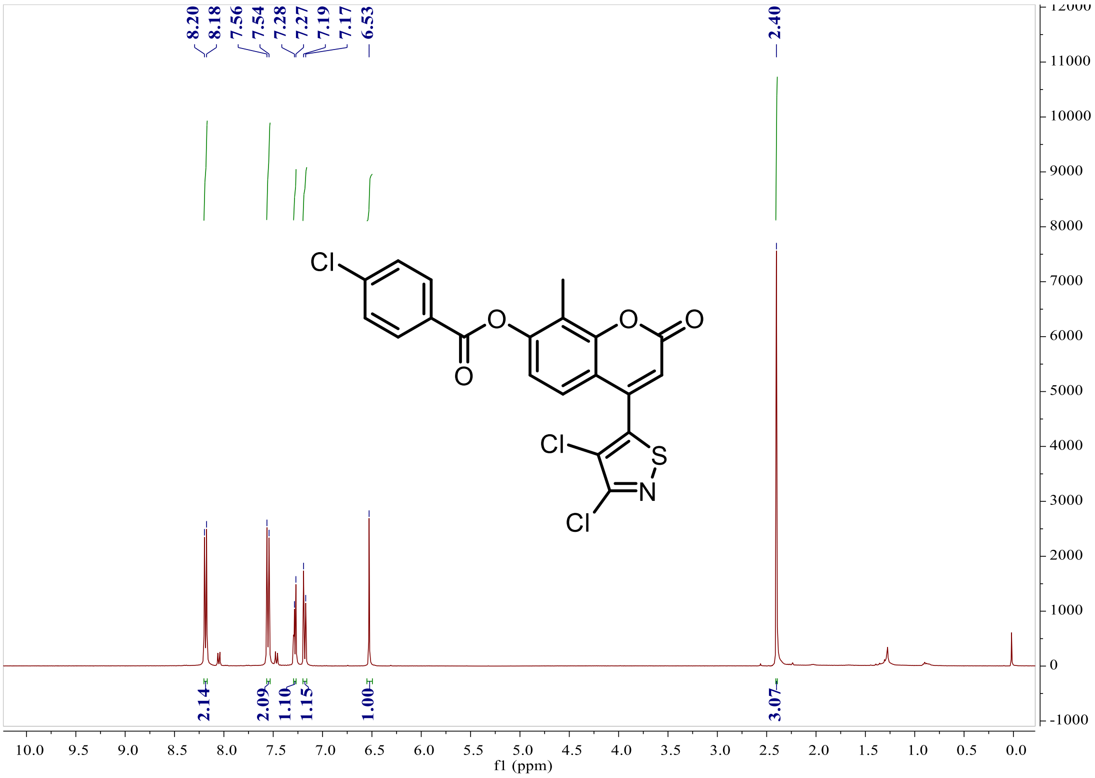

Figure S137**. The ^{1}H NMR (400MHz, Chloroform-*d*) of compound 2bu.**

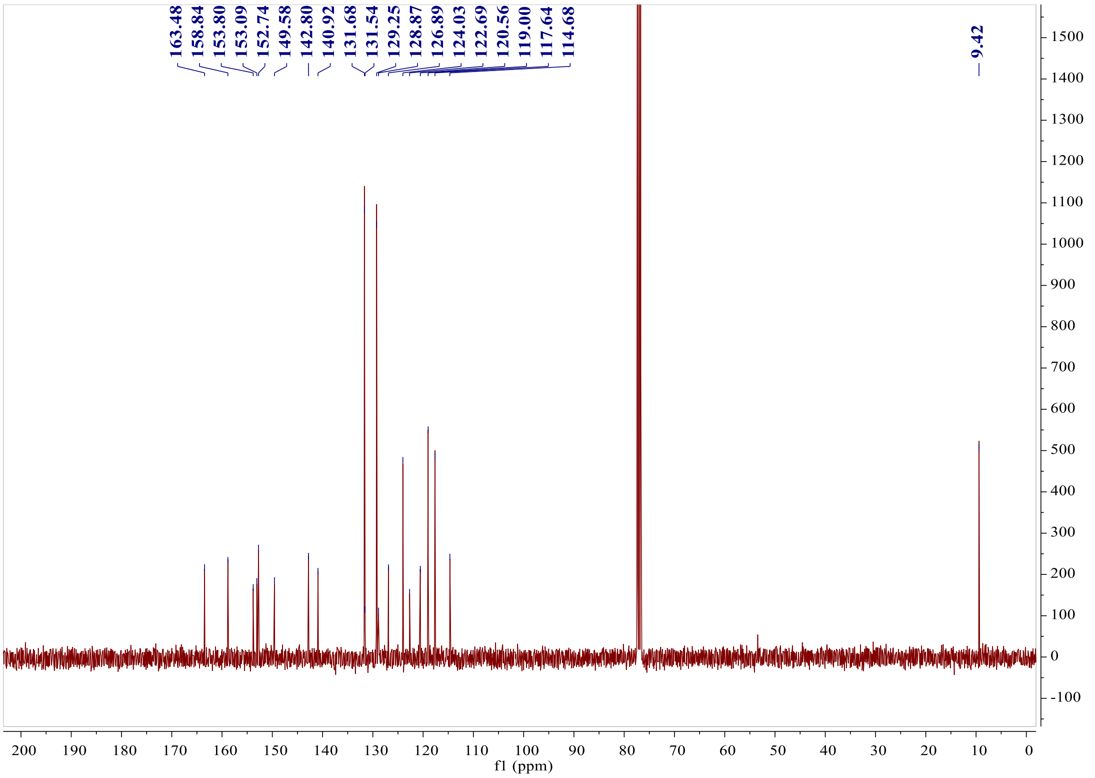

Figure S138**. The ^{13}C NMR (101MHz, Chloroform-*d*) of compound 2bu.**

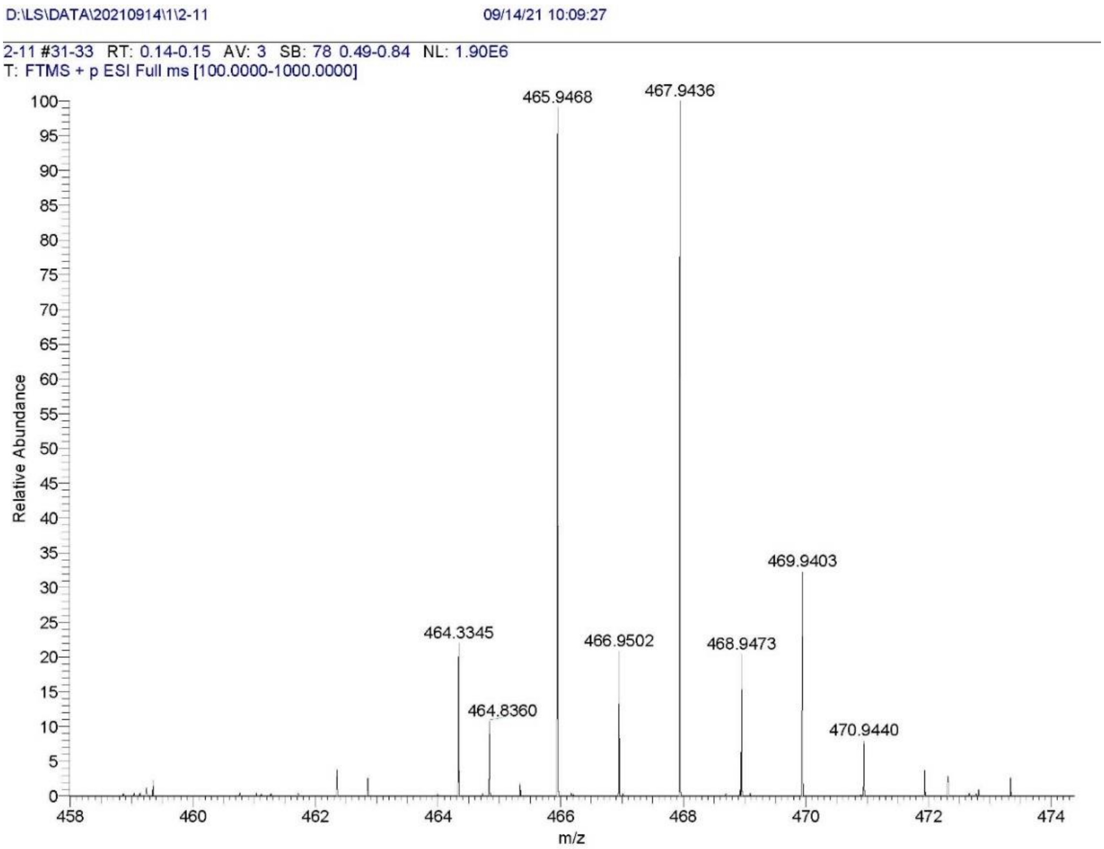

Figure S139. **The HRMS of compound 2bu.**

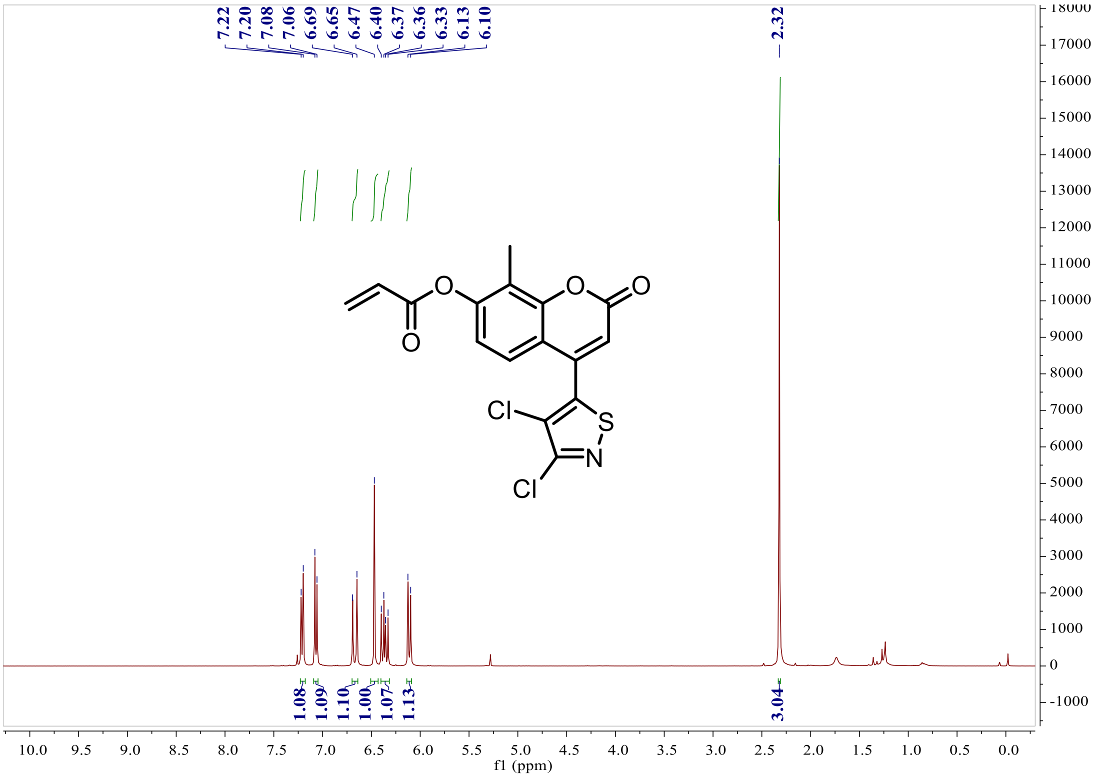

Figure S140. **The ^{1}H NMR (400MHz, Chloroform-*d*) of compound 2bv.**

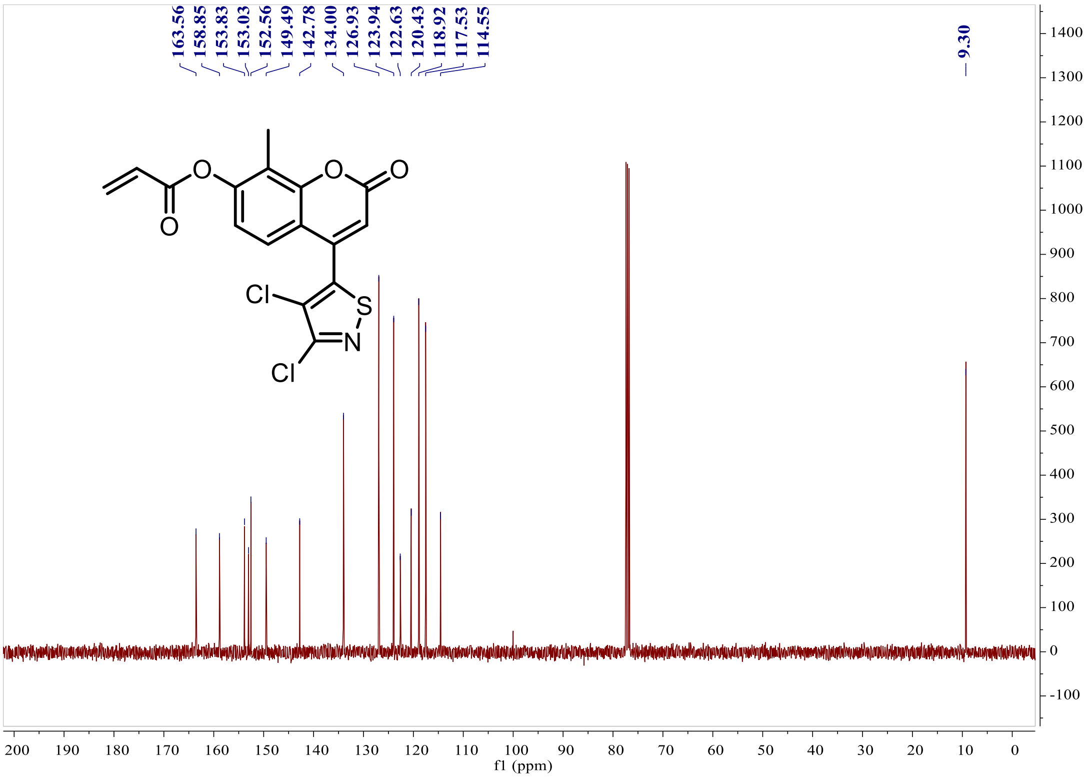

Figure S141. **The ^{13}C NMR (101MHz, Chloroform-*d*) of compound 2bv.**

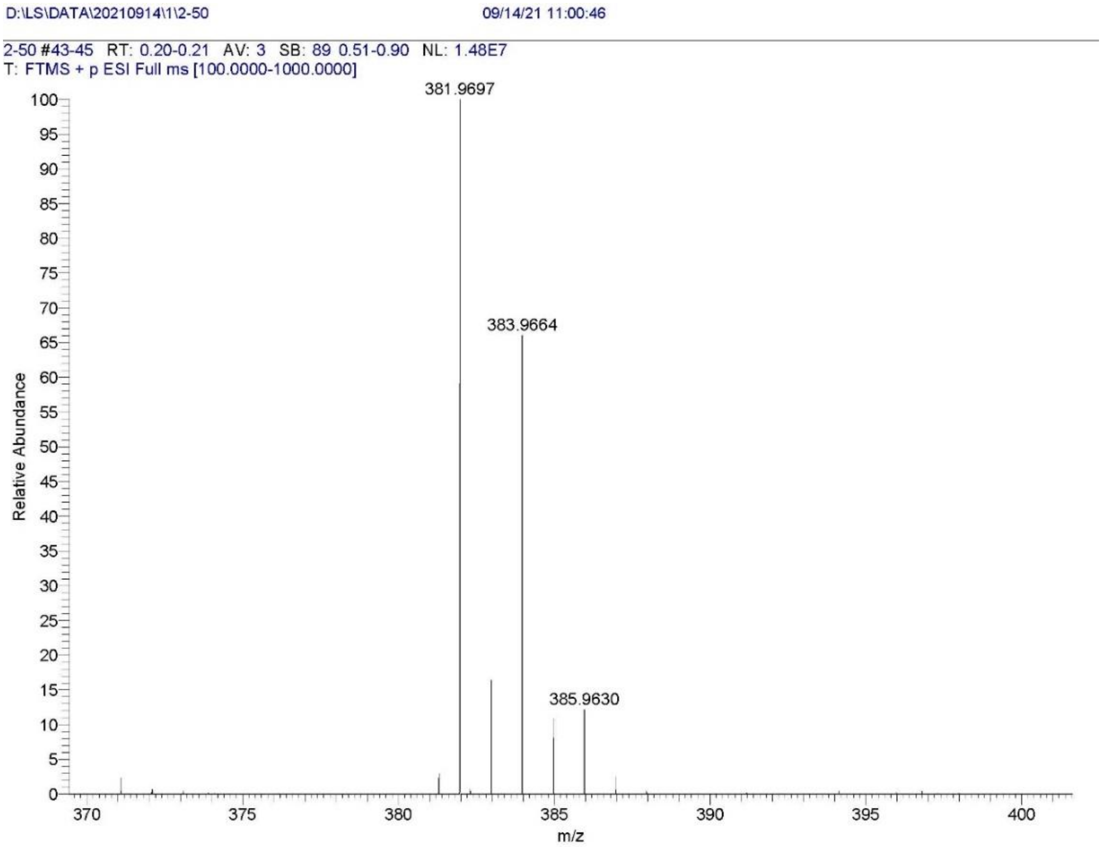

Figure S142. **The HRMS of compound 2bv.**

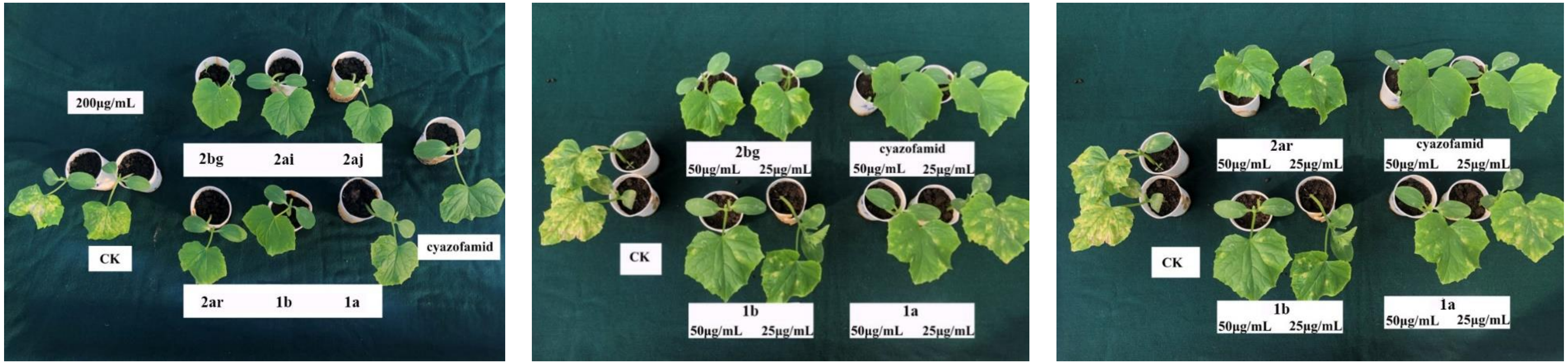

Figure S143. *In vivo* fungicidal activities of the compounds **2ai**, **2aj**, **2ar** and **2bg**.
